# *Haemonchus contortus* Acetylcholine Receptors of the DEG-3 Subfamily and Their Role in Sensitivity to Monepantel

**DOI:** 10.1371/journal.ppat.1000380

**Published:** 2009-04-10

**Authors:** Lucien Rufener, Pascal Mäser, Isabel Roditi, Ronald Kaminsky

**Affiliations:** 1 Novartis Centre de Recherche Santé Animale, St. Aubin (FR), Switzerland; 2 Institute of Cell Biology, University of Bern, Switzerland; NIAID/NIH, United States of America

## Abstract

Gastro-intestinal nematodes in ruminants, especially *Haemonchus contortus*, are a global threat to sheep and cattle farming. The emergence of drug resistance, and even multi-drug resistance to the currently available classes of broad spectrum anthelmintics, further stresses the need for new drugs active against gastro-intestinal nematodes. A novel chemical class of synthetic anthelmintics, the Amino-Acetonitrile Derivatives (AADs), was recently discovered and the drug candidate AAD-1566 (monepantel) was chosen for further development. Studies with *Caenorhabditis elegans* suggested that the AADs act via nicotinic acetylcholine receptors (nAChR) of the nematode-specific DEG-3 subfamily. Here we identify nAChR genes of the DEG-3 subfamily from *H. contortus* and investigate their role in AAD sensitivity. Using a novel in vitro selection procedure, mutant *H. contortus* populations of reduced sensitivity to AAD-1566 were obtained. Sequencing of full-length nAChR coding sequences from AAD-susceptible *H. contortus* and their AAD-1566-mutant progeny revealed 2 genes to be affected. In the gene *monepantel-1* (*Hco-mptl-1*, formerly named *Hc*-acr-23H), a panel of mutations was observed exclusively in the AAD-mutant nematodes, including deletions at intron-exon boundaries that result in mis-spliced transcripts and premature stop codons. In the gene *Hco-des-2H*, the same 135 bp insertion in the 5′ UTR created additional, out of frame start codons in 2 independent *H. contortus* AAD-mutants. Furthermore, the AAD mutants exhibited altered expression levels of the DEG-3 subfamily nAChR genes *Hco-mptl-1*, *Hco-des-2H* and *Hco-deg-3H* as quantified by real-time PCR. These results indicate that *Hco*-MPTL-1 and other nAChR subunits of the DEG-3 subfamily constitute a target for AAD action against *H. contortus* and that loss-of-function mutations in the corresponding genes may reduce the sensitivity to AADs.

## Author Summary

Worldwide, sheep and cattle farming are threatened by anthelmintic-resistant gastro-intestinal nematodes. A novel chemical class of synthetic anthelmintics was recently discovered, the Amino-Acetonitrile Derivatives (AADs), which exhibit excellent efficacy against various species of livestock-pathogenic nematodes and, more importantly, overcome existing resistances to the currently available anthelmintics. *Haemonchus contortus*, the largest nematode found in the abomasum of sheep and cattle, is a blood-feeding parasite that causes severe anemia that can lead to the sudden death of the infected animal; *H. contortus* is highly susceptible to AADs. In order to elucidate the mode of action of the AADs, we have developed 2 independent *H. contortus* mutants with reduced sensitivity to monepantel (AAD-1566). Both mutants were affected in their acetylcholine receptor (ACR) genes of the DEG-3 subfamily. In particular, we discovered a panel of mutations in the gene *monepantel-1* (*Hco-mptl-1*) including deletions leading to mis-splicing, insertions and point mutations leading to premature termination of translation of the protein. These findings support the notion that *Hco*-MPTL-1 and other nAChR subunits of the DEG-3 subfamily are targets of the AADs. The fact that the DEG-3 subfamily of acetylcholine receptors is nematode-specific may explain the good therapeutic index of AADs in mammals.

## Introduction

Throughout the world, successful livestock production of ruminants is hampered by gastro-intestinal nematodes. *Haemonchus contortus* in particular is responsible for substantial losses to the global sheep industry [1]. *Haemonchus contortus* is a blood-feeding nematode that inhabits the abomasum of sheep, producing in acute infections, severe anemia that can lead to the death of infected animals.

Broad spectrum chemotherapy against gastro-intestinal nematodes is restricted to 3 anthelmintic classes: the benzimidazoles, such as albendazole and oxfendazole, the imidazothiazoles, including levamisole and tetramisole and the macrocyclic lactones (e.g. ivermectin, moxidectin, abamectin and doramectin). The increased usage of anthelmintics has contributed to the spread of resistant nematodes with increasing reports of nematodes insensitive to most if not all of the available classes of anthelmintics [2]–[10]. In some countries in the southern hemisphere, sheep farming is severely endangered by such populations [4], further increasing the need for a new class of anthelmintic [11].

Recently, a new class of compounds, the Amino-Acetonitrile Derivatives (AADs) was discovered [12] with good tolerability in mammals and promising activity against drug-resistant nematodes. The AADs are low molecular mass compounds bearing different aryloxy and aroyl moieties on an amino-acetonitrile core [13]. Further studies [14] have allowed the selection of a drug candidate, AAD-1566 (monepantel). In order to investigate the mode of action of this new class of compounds, AAD-resistant *Caenorhabditis elegans* mutants were generated by EMS mutagenesis. Classical forward genetics revealed that the majority of recuperated AAD-resistant mutants carried mutations in the gene *acr*-*23*, a member of the nematode-specific DEG-3 subfamily of nicotinic acetylcholine receptor (nAChR) alpha subunits [12]. Preliminary data had already indicated an involvement of similar acetylcholine receptors in AAD action against *H. contortus* [12]. Here we report the identification of the gene *monepantel-1* (*Hco-mptl-1*, formerly named *Hc*-acr-23H) and other members of the DEG-3 subfamily of ACR genes from *H. contortus*. A panel of different mutations, mis-splicing in particular, in *Hco-mptl-1* transcripts from AAD-resistant worms indicates that *Hco*-MPTL-1 is a target for monepantel action against *H. contortus*.

## Materials and Methods

### *Haemonchus contortus* isolates

The drug-susceptible *H. contortus* CRA (*Hc*-CRA) was received in 1984 from the Veterinary Institute of Onderstepoort, Republic of South Africa and has since been passaged in sheep 75 times. The *H. contortus* Howick isolate (*Hc*-Howick) was received from the same institute in 2001. This is a multidrug-resistant isolate that is completely resistant to albendazole, rafoxanide, morantel, ivermectin and trichlorfon [6],[15]. The isolate has been passaged in sheep 9 times since being received. The mutant lines *Hc*-CRA AAD^M^ and *Hc*-Howick AAD^M^ were selected from *Hc*-CRA and *Hc*-Howick, respectively, by in vitro exposure to increasing doses of AAD-1566 alternatively with propagation in sheep [12].

### Collection of nematode eggs

*Haemonchus contortus* isolates were propagated in 3–6 month old sheep (‘Blanc des Alpes’), which had been experimentally infected with the nematode. The sheep were kept in groups of 4 and housed indoors off pasture to prevent natural infection. After 14 days, they were transferred to individual cages. Starting on day 21 after infection, eggs were collected from homogenized feces and filtered several times through a 32 µm sieve. Eggs were further purified by floating on 50% sucrose solution, rinsed with water and counted microscopically.

### In vivo determination of drug sensitivity

Sheep studies were performed with approval of a Cantonal animal welfare committee (permit number FR 25A/05). Anthelmintic efficacy tests in sheep were performed according to the guidelines of the World Association for the Advancement of Veterinary Parasitology [16]. Each animal was infected intraruminally on study day −21 with 3000 L_3_-larvae of *H. contortus* (cultivated in coprocultures). On study day 0, the sheep were treated with single anthelmintics or combinations thereof as an oral drench at the recommended dose. A sheep was classified as ‘cured’ when no more eggs were counted in the feces and no adults were found in the abomasum at necropsy.

### Recovery of adult *Haemonchus contortus* and isolation of nucleic acids

Adult worms were recovered from the abomasum of freshly euthanized sheep, washed in Hank's Buffered Salt Solution (HBSS; Invitrogen) and immediately shock-frozen in liquid nitrogen. While frozen, the worms were crushed with a Kontes pellet pestle (Fisher Scientific). The powder was resuspended in 600 µl of lysis buffer (10 mM Tris pH 7.5, 1 mM EDTA, 100 mM NaCl, 0.5% SDS, 100 µg/ml RNase A) and incubated at 37°C for 1 hour. Pronase (100 µg/ml) was added to the mixture and the tubes were incubated at 37°C until the solution became clear. The samples were extracted with equal volumes of phenol∶chloroform (1∶1) and chloroform. The DNA was ethanol precipitated, washed and resuspended in 50 µl of Tris-Cl (pH 7.5). For RNA extraction, worms were homogenized in TRIzol and processed according to the instructions of the supplier (Invitrogen). To remove DNA contamination, the RNA samples were treated with a TURBO DNA-free kit (Ambion). To generate cDNA, 1 µg of total RNA was reverse transcribed to cDNA using a d(T)_30_ primer and a Moloney Murine Leukemia Virus Reverse Transcriptase (MMLV RT; SMART cDNA library construction kit from Clontech).

### Construction and screening of a *Haemonchus contortus* cDNA library

A total of 4 µg of mRNA was isolated from a mixture of male and female *Hc*-CRA using a Oligotex kit from Qiagen. A cDNA library was constructed with the ZAP-cDNA Cloning kit and Gigapack III Gold packaging kit. The library was screened at high stringency (hybridization at 65°C in 5×SSC, 5× Denhardt's solution, 0.1% SDS, 0.1% sodium pyrophosphate, 100 µg/ml salmon sperm DNA; final wash at 60°C in 0.2×SSC, 0.1% SDS) with a ^32^P-labeled 456 bp fragment of *Hco-mptl-1*. This fragment had been amplified from cDNA with the primers *Hco-mptl-1*_frw3 and *Hco-mptl-1*_rev1 and cloned into pCR®2.1-TOPO® (Invitrogen). Positive phages were taken through 3 rounds of plaque purification with this probe and the phagemid (pBluescript SK+) was excised using the ExAssist helper phage in the *E. coli* SOLR strain. Inserts were sequenced in both directions with standard M13 forward and reverse primers and the internal primers *Hco-mptl-1*_frw4 and *Hco-mptl-1*_rev3. The sequences were read and assembled using 4Peaks (by A. Griekspoor and T. Groothuis; http://mekentosj.com).

### PCR

The primers used for PCR-amplification, real-time PCR or for cDNA first strand synthesis of *H. contortus* nAChR genes are summarized in Table S1. For nested PCR on cDNA with spliced leader (SL) primers, the primary products were diluted 50-fold and 2 µl were used for the second PCR with nested primers. The annealing temperature was fixed at 55°C for cDNA and 58°C for genomic DNA template. PCR products were gel purified using the NucleoSpin® ExtactII kit (Macherey-Nagel) and cloned into either pGEM-T easy (Promega) or pCR®2.1-TOPO® (Invitrogen). Plasmid DNA was purified using the QIAprep Spin Miniprep Kit (Qiagen) and sequenced using the standard primers M13 forward and reverse and, if necessary, an additional internal primer to cover long products. For rapid amplification of cDNA ends by PCR (RACE-PCR), an internal reverse primer (Table S1) was combined with splice leader sequence (1 or 2) to obtain the 5′ UTR, or an internal forward primer combined with a poly-dT primer for the 3′ UTR of the transcript.

For real-time PCR, 1 µg of total RNA from adult *H. contortus* was used to synthesize first-strand cDNA by random priming using Superscript II reverse transcriptase (Invitrogen) in a final volume of 20 µl following the manufacturer's instructions. Reverse-transcribed material corresponding to 40 ng RNA was amplified in 25 µl MESA GREEN qPCR MasterMix Plus for SYBR Assay (Eurogentec) by using the ABI SDS7000 Sequence Detection System under the following conditions: 1 cycle of 95°C for 15 minutes followed by 40 cycles of 95°C for 15 seconds and 60°C for 1 minute. The primer pairs used for the amplification are listed in Table S1 and target the following genes: β-tubulin, *Hco-mptl-1*, *Hco-des-2H* and *Hco-deg-3H*. Three independent total RNA extractions were performed and each was tested in duplicate. Relative expression values were calculated according to Livak and Schmittgen [17]; a 136 bp region within the phosphoglucose isomerase gene was used for normalization, a 122 bp region within the β-tubulin gene was used as a (presumably) non-affected control, and no reverse transcriptase and no template reactions as negative controls. The specificity and identity of individual amplicons were verified by melt curve analysis and visualized on a 2% agarose gel.

## Results

### In vivo sensitivity of *Haemonchus contortus* AAD mutants

In order to study the mode of action of the AADs, we used 2 mutant isolates, *Hc*-CRA AAD^M^ and *Hc*-Howick AAD^M^ selected from parent *Hc*-CRA and *Hc*-Howick isolates, respectively. Both mutant isolates showed reduced sensitivity to AAD-1566 (monepantel) in vitro [12]. To test whether the observed loss of susceptibility to AAD-1566 in vitro was relevant for the situation in vivo, *Hc*-CRA, *Hc*-Howick and their AAD^M^ derivatives were challenged in vivo with single compounds or combinations thereof; AAD-1566 and the commercial compounds were applied at their recommended doses to sheep. Sheep were infected intraruminally with *Hc*-CRA AAD^M^. Following treatment with AAD-1566 at the proposed minimum dose rate of 2.5 mg/kg body weight [18] eggs were found in the feces and adults seen at necropsy (Table 1). Likewise, nematode eggs and adults were also found in sheep infected with *Hc*-Howick AAD^M^ larvae when treated either with AAD-1566 or albendazole or a combination of AAD-1566 and ivermectin (Table 1). The offspring from the *Hc*-Howick AAD^M^ isolate that survived the AAD-1566 and ivermectin treatment were cultured and challenged with albendazole and levamisole over the following generations (data not shown). Finally, *Hc*-Howick AAD^M^ was able to survive a full simultaneous in vivo treatment with albendazole, levamisole, ivermectin and AAD-1566, administered at their recommended doses (Table 1). Thus the reduction of sensitivity to AAD-1566 induced in vitro was also relevant in vivo for the mutant lines. The AAD-mutant *H. contortus* apparently did not show any alterations in motility, infectivity to sheep (determined by the numbers of adult *H. contortus* recovered at necropsy) or egg production, and did not exhibit any phenotype with respect to the ultrastructure (by electron microscopy) of the cuticle, head or tail.

**Table 1:** In vivo sensitivity of adult stages of *H. contortus* CRA, CRA AAD^M^, Howick and Howick AAD^M^.

Isolates	Drug	Dose (oral drench)	Number of animals effectively treated/number of animals treated
*H. contortus* CRA	Albendazole	3.8 mg kg^−1^	4/4
	Ivermectin	0.2 mg kg^−1^	2/2
	AAD-1566	2.5 mg kg^−1^	3/3
*H. contortus* CRA AAD^M^	AAD-1566	2.5 mg kg^−1^	0/3
	Albendazole	3.8 mg kg^−1^	3/3
*H. contortus* Howick	AAD-1566	2.5 mg kg^−1^	3/3
	Combination of:		
	Albendazole	3.8 mg kg^−1^	0/3
	+Levamisole	7.5 mg kg^−1^	
	+Ivermectin	0.2 mg kg^−1^	
*H. contortus* Howick AAD^M^	AAD-1566	2.5 mg kg^−1^	0/3
	Albendazole	3.8 mg kg^−1^	0/2
	Combination of:		
	AAD-1566	2.5 mg kg^−1^	0/2
	+Ivermectin	0.2 mg kg^−1^	
	Combination of:		
	AAD-1566	2.5 mg kg^−1^	0/1
	+Levamisole	7.5 mg kg^−1^	
	+Ivermectin	0.2 mg kg^−1^	
	+Albendazole	3.8 mg kg^−1^	

Sheep were treated orally with commercial compounds at the recommended doses. An animal was considered to have been effectively treated when no more eggs were counted in the feces and no adults were found in the abomasum at necropsy.

### The *Haemonchus contortus* DEG-3 subfamily compared to related nematodes species

**Figure 1:**
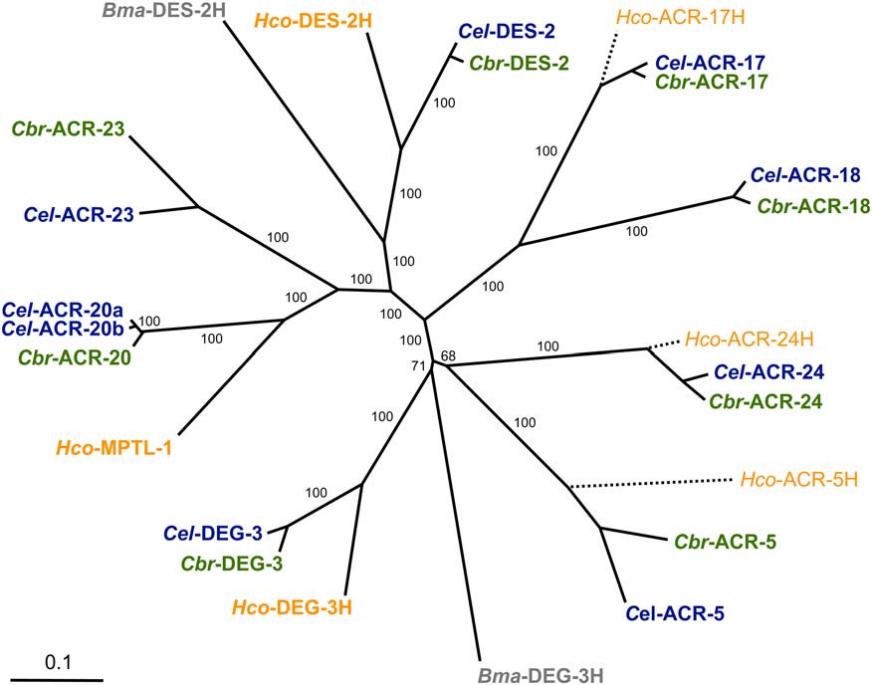
Phylogenetic analysis of the DEG-3 subfamily of nAChR. ClustalW dendrogram [49] of nAChRs subunits of the DEG-3 subfamily (amino acid sequences) from *B. malayi* (*Bma*; grey), *C. briggsae* (*Cbr*; green), *C. elegans* (*Cel*; blue), and *H. contortus* (*Hco*; orange). Two isoforms (a and b) of *Cel*-ACR-20 are shown. The scale bar indicates the number of amino acid substitutions per site, bootstrapping values are shown in percent positives of 1000 rounds. Tree construction and bootstrapping was initially performed on full-length sequences only; the partial sequences (dashed lines, thin characters) were added subsequently based on a second ClustalW guide tree.

The putative target of the AADs in *C. elegans*, ACR-23, is a member of the nematode-specific DEG-3 family of nAChR alpha subunits. A tblastn search [19] with DEG-3 members against the (incomplete) *H. contortus* genome database (http://www.sanger.ac.uk/Projects/H_contortus) returned strong hits from different contigs, coding for a total of 6 different DEG-3 subfamily nAChR subunit homologues. However, the lack of overlap between the different contigs precluded the assembly of full length coding sequences. The predicted *H. contortus* proteins were named *Hco*-MPTL-1 (accession number: contig_0024907; contig_0033952; contig_0079482; haem-240m02.q1k; contig_0053297; contig_069357), *Hco*-DES-2H (contig_0064641), *Hco*-DEG-3H (contig_0075200; contig_0075201), *Hco*-ACR-24H (contig_0003482; contig_0064300), *Hco*-ACR-5H (contig_0106281; contig_0023143) and *Hco*-ACR-17H (contig_0101516; contig_0101514). For *Hco*-MPTL-1, *Hco*-DES-2H and *Hco*-DEG-3H, full-length coding sequences were obtained by cDNA library screening or RACE-PCR, respectively (see below). Figure 1 shows the position of the *H. contortus* sequences in a phylogenetic tree of the DEG-3 subfamily nAChR from *C. elegans*, *C. briggsae and Brugia malayi*. Note that an incomplete sequence of *Hco*-MPTL-1 was previously named *Hc*-ACR-23H [12].

### Cloning of *Haemonchus contortus Hco-mptl-1*

To obtain the full length coding sequence of the *Hco-mptl-1* gene, a lambda phage cDNA library from mRNA of adult *H. contortus* was constructed and screened at high stringency with a radioactive probe from a partial *Hco-mptl-1* sequence. After 3 rounds of selection, a clone with the full-length coding sequence, *Hco-mptl-1*, was isolated and sequenced. The *Hco-mptl-1* mRNA is composed of at least 17 exons and 16 introns (1992 bp) with a short 5′ UTR and 3′ UTR (21 bases and 42 bases, respectively). The transcript is trans-spliced as the splice leader 1 (SL1) is present at its 5′ end. Interestingly, a start codon (AUG) is present in exon 1 but is followed after 8 amino acids by a stop codon in frame (UGA). This is a feature found in many other organisms [20]–[22] and it is assumed to play a role in the regulation of translation efficiency. In most cases, upstream AUGs decrease mRNA translation efficiency and have a strong, negative regulatory effect [23]. The longest open reading frame (ORF) in the *Hco-mptl-1* gene is obtained when the translation is initiated at the second AUG codon in exon 3 and extends over 1695 bases. Overlapping long range PCR was performed in order to estimate the total size of *Hco-mptl-1*. The gene was found to be approximately 18.5 kb long with a large intron (about 7 kb) between exons 3 and 4 (see below). The predicted *Hco*-MPTL-1 protein consists of 564 amino acids and possesses motifs typical for Cys-loop ligand-gated ion channels, including an N-terminal signal peptide of 18 amino acids [24], 4 transmembrane domains and the Cys-loop (2 cysteines separated by 13 amino acids). Loops A to F, which are involved in ligand binding [25] are also present in the protein (Figure S1). In loop C, there are 2 adjacent cysteines, defining *Hco*-MPTL-1 as a nAChR alpha subunit.

**Figure 2:**
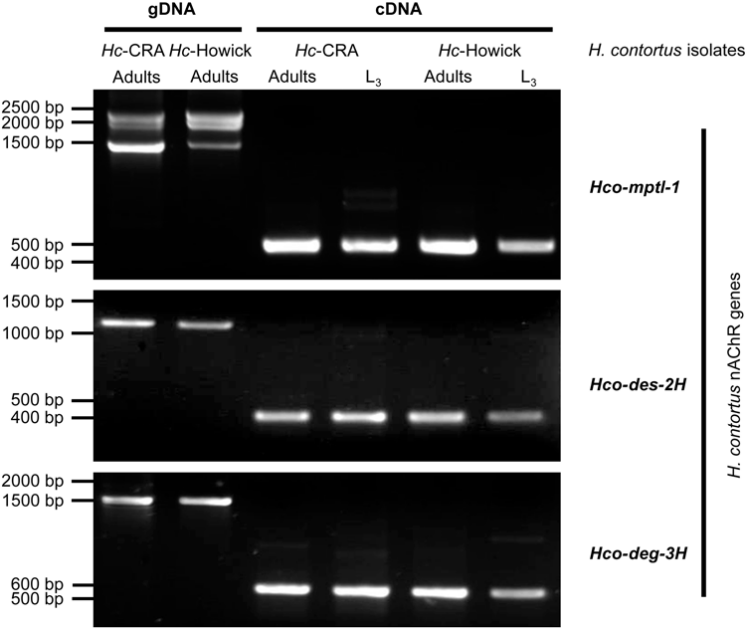
Expression of DEG-3 subfamily members in *Haemonchus contortus*. *Hco-mptl-1*, *Hco-des-2H* and *Hco-deg-3H* are expressed in adult as well as L_3_-larvae stages of both the *Hc*-CRA and *Hc*-Howick parental reference isolates (AAD naïve) as determined by reverse transcriptase PCR. Genomic DNA (gDNA) was included as a control.

As determined by PCR with gene-specific primers on genomic DNA, *Hco-mptl-1* (*Hco-mptl-1*_frw6 and *Hco-mptl-1*_rev6), *Hco-des-2H* (*Hco*-des2_frw8 and *Hco*-des2_rev8) and *Hco-deg-3H* (*Hco*-deg3_frw1 and *Hco*-deg3_rev1) are present in the *Hc*-CRA and *Hc*-Howick parental isolates (Figure 2). Of the 3 products obtained for the *Hco-mptl-1* gene, the smallest one (1478 bp) corresponded to *Hco-mptl-1*. The same primers were used for reverse transcriptase PCR on total RNA, showing that all 3 genes were expressed and spliced in L_3_-larvae as well as in adult nematodes (Figure 2).

The predicted *Hco*-MPTL-1 protein shares 48.5% identity and 66.8% similarity with *C. elegans* ACR-23 and 60.2% identity and 70.7% similarity with *C. elegans* ACR-20. The novel *H. contortus* nAChR was originally named *Hc*-ACR-23H based on a partial sequence that was most closely related to *C. elegans* ACR-23 [12]. In the light of the full-length sequence, this nomenclature seems to have been premature since the *Haemonchus* nAChR turned out to be more closely related to *C. elegans* ACR-20 (Figure 1). In the absence of a complete record of ACR paralogues from *H. contortus*, and in analogy to levamisole-insensitive (*lev*-) mutants in *C. elegans* [26], we propose to name the gene *monepantel-1* (*Hco-mptl-1*) due to its apparent involvement in monepantel sensitivity.

### *Hco-mptl-1* mutations associated with the AAD-mutant phenotype

**Figure 3:**
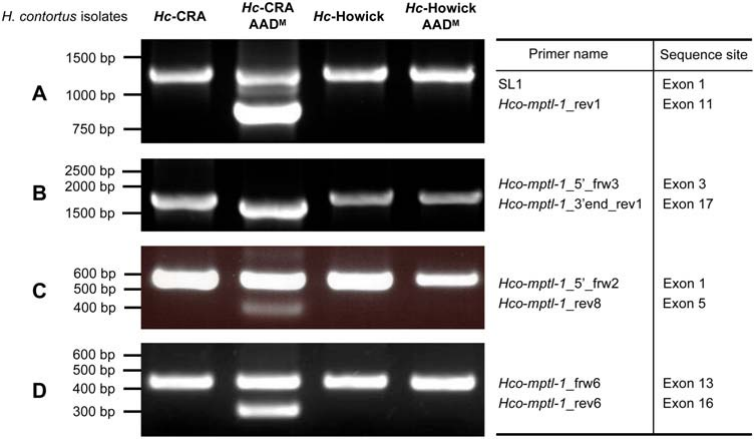
Deletions in the *Hco-mptl-1* coding sequence. PCR products were amplified from cDNA of the mutant (AAD^M^) and sensitive parental isolates. Different pairs of primers were tested in order to map the region where the deletions occurred. No apparent deletions were observed in *Hc*-Howick AAD^M^ mutants. Note the apparent absence of a full-length product for Hc-CRA AAD^M^ in panel B, where the primers encompass both critical exons 4 and 15 (Figure 4), indicating the absence of wild-type *Hco-mptl-1* transcripts in this mutant.

**Figure 4:**
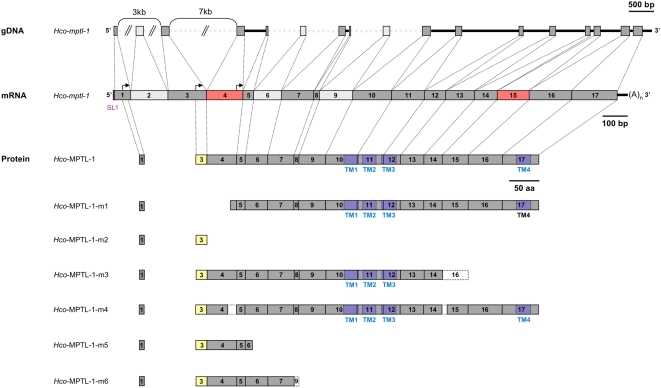
The *Hco-mptl-1* locus, mRNA and protein (top) and mis-splicing mutations in the AAD mutants (bottom). Exons are represented by boxes, start codons by arrows. The 5′ region of the genomic DNA is not drawn to scale (double parallel bars). No hits were found in the *H. contortus* genome project for the exons and introns shown in clear grey. The spliced leader is shown in violet and mis-spliced exons in red. The signal peptide is shown in yellow and the predicted transmembrane domains (TM) in blue.

In order to compare the *Hco-mptl-1* sequences from the AAD-susceptible isolates and their AAD-mutant progeny, primers were designed at each extremity of the ORF (*Hco-mptl-1*_5′_frw3 and *Hco-mptl-1*_3′end_rev1) and the full length *Hco-mptl-1* coding sequences amplified from cDNA from adults. A product of about 1800 bp was obtained for all isolates apart from the *Hc*-CRA AAD^M^, which produced a shorter product of 1650 bp (Figure 3B). Sequencing clones of the latter revealed that they lacked either exon 4 or exon 15 (Figure 4, *Hco*-MPTL-1-m2 and m3). This was confirmed with primers flanking either exon 4 (*Hco-mptl-1*_5′_frw2 and *Hco-mptl-1*_rev8; Figure 3C) or exon 15 (*Hco-mptl-1*_frw6 and *Hco-mptl-1*_rev6; Figure 3D). PCR with a SL1 forward primer and a reverse primer in the *Hco-mptl-1* coding sequence (*Hco-mptl-1*_rev1, product of about 1200 bp; Figure 3A) also produced shorter products (1000 bp and 850 bp; Figure 3A) from *Hc*-CRA AAD^M^. The 850 bp product turned out to lack both exon 2 and exon 3 while the 1 kb product lacked exon 4 (Figure 4, *Hco*-MPTL-1-m1 and m2). The 1200 bp product was cloned from *Hc*-CRA AAD^M^ but contained only silent mutations compared to *Hc*-CRA. Loss of exon 4 caused a frame-shift leading to a premature stop of translation and a predicted *Hco*-MPTL-1 protein truncated at amino acid 19 (Figure 4). Loss of exon 15 also led to a premature stop codon that truncated the *Hco*-MPTL-1 protein at amino acid 448 (Figure 4). The mutation *Hco*-MPTL-1-m1 (loss of exon 2 and 3) did not cause a frame-shift but the loss of the signal peptide and the first 39 amino acids of the extracellular loop.

### Mutation cause mis-splicing of the *Hco-mptl-1* transcript in *Hc*-CRA AAD^M^ mutants

**Figure 5:**
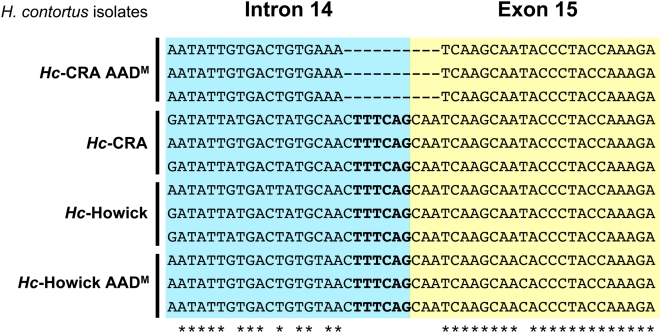
*Hc*-CRA AAD^M^ mutants lack the splice acceptor site of intron 14. Sequencing of PCR products amplified from genomic DNA revealed a 10 bp deletion in the *Hc*-CRA AAD^M^ mutant that encompasses the predicted splice acceptor site (bold). The blue box corresponds to the end of intron 14 and the yellow box to the start of exon 15. Asterisks denote bases identical throughout all 12 sequenced clones.

To understand the molecular basis of exon loss in the *Hc*-CRA AAD^M^ isolate, PCR primers *Hco-mptl-1*_frw8 and *Hco-mptl-1*_rev6 (Table S1) were designed to flank the mis-spliced exon 15. PCR was performed using genomic DNA as a template. Sequencing of cloned PCR products revealed a 10 bp deletion upstream of exon 15 in the *Hc*-CRA AAD^M^ mutant that encompasses the predicted splice acceptor site (UUUCAG; Figure 5). Presumably, the splicing machinery is not able to identify the end of intron 14 and uses the next splice acceptor site (intron 15). This would explain why exon 15 is skipped (Figure 4, *Hco*-MPTL-1-m3). Joining of exon 14 to exon 16 causes a frame-shift leading to a premature stop codon. With primers flanking exon 4 (*Hco-mptl-1*_frw10/gDNA and *Hco-mptl-1*_rev8; Table S1), a 323 bp deletion was detected consisting of the end of intron 3 (206 bp) and most of exon 4 (117 bp). Again, loss of the predicted splice acceptor site at the end of intron 3 may explain the observed loss of exon 4 (Figure 4, *Hco*-MPTL-1-m2), since the splicing machinery will use the next available splice acceptor site (intron 4), joining exon 3 and exon 5. The resulting frame-shift causes a premature stop at codon 19 (TGA), terminating translation after the signal peptide (Figure 4, *Hco*-MPTL-1-m2).

### Detection of the *Hco-mptl-1* E93* point mutation in the *Hc*-Howick AAD^M^ nematodes

**Figure 6:**
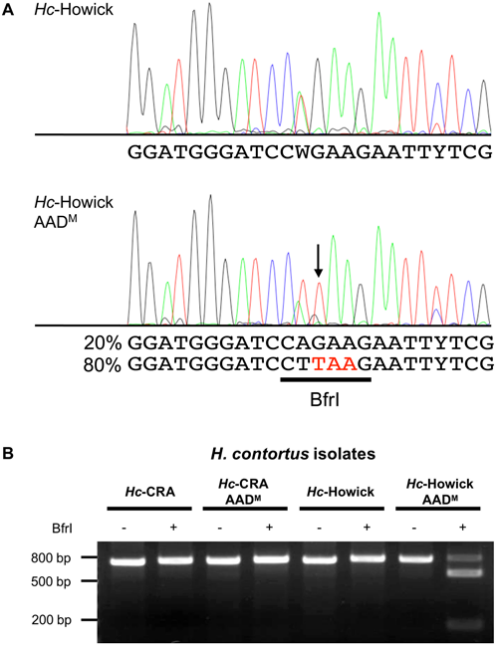
Detection of a nonsense mutation in *Hc*-Howick AAD^M^ worms. (A) Direct sequencing of RT-PCR products revealed a transversion in exon 6 from G to T (arrow) in the *Hco-mptl-1* gene that leads to a premature stop at codon 93 (TAA; shown in red) in about 80% of *Hc*-Howick AAD^M^ mutants as estimated from the electropherogram. (B) The point mutation creates a restriction site for the endonuclease BfrI (CTTAAG; underlined). Only the product amplified from cDNA of the *Hc*-Howick AAD^M^ mutant could be digested.

No obvious mutations such as mis-spliced exons were detected in the *Hc*-Howick AAD^M^ isolates. When sequencing the *Hco-mptl-1* coding regions (SL1 and *Hco-mptl-1*_rev6) from both susceptible and AAD-1566-mutant Howick isolates, a transversion from G_277_ to T in exon 6 of the *Hco-mptl-1* gene was observed that led to a premature stop codon (E93*; Figure 6). Direct sequencing of RT-PCR products (using *Hco-mptl-1*_frw4 and *Hco-mptl-1*_rev1 primers) revealed that about 80% of the *Hc*-Howick AAD^M^ cDNAs, as estimated from the electropherogram [27], carried a T at position 277 (Figure 6A). The point mutation underlying E93* creates a restriction site for the endonuclease BfrI (recognition site: CTTAAG) that lent itself for RFLP analysis. Only the PCR product amplified from cDNA of *Hc*-Howick AAD^M^ was digested by BfrI (Figure 6B). As expected from the sequencing, a small proportion (about 20%) of the product was not cut, indicating that not all of the *Hco-mptl-1* genes from *Hc*-Howick AAD^M^ population carried the G277T mutation. When this BfrI-unrestricted product from *Hc*-Howick AAD^M^ was excised from an agarose gel, cloned and sequenced, a further polymorphism was detected that led to skipping of exon 8 (Figure 4, *Hco*-MPTL-1-m6). As this exon is very short (22 bases), it was impossible to discriminate between mutant and parental wild type PCR products (Figure 3). Loss of exon 8 causes a frame-shift leading to a premature stop codon and a predicted *Hco*-MPTL-1 protein truncated at amino acid 166 (Figure 4). A minority of the *Hco-mptl-1* PCR products obtained from *Hc*-Howick AAD^M^ did not contain any major mutations. These sequences could come from AAD-susceptible individuals within the *H. contortus* Howick AAD^M^ populations or from AAD-mutant individuals that carry other, yet to be identified mutations.

### An insertion in the 5′ UTR of the *des-2* homologue of *Haemonchus contortus* AAD mutants

As the DEG-3 subfamily gene *Hco-des-2H* has also been implicated in AAD action in *H. contortus* [12], we cloned and sequenced the full-length *Hco-des-2H* coding sequence from *H. contortus* cDNA by RACE-PCR. Using primers NheI_des2_frw1 and XhoI_des2_rev1 (Table S1), 2 products were obtained from the four *H. contortus* isolates. Cloning and sequencing revealed the smaller transcript to lack 168 bases coding for part of the internal loop between TM3 and TM4, possibly indicating alternative splicing of the *Hco-des-2H* gene. The predicted protein (full version) consists of 534 amino acids and shows 69% identity and 80% similarity with *C. elegans* DES-2. *Hco*-DES-2H possesses motifs typical for Cys-loop ligand-gated ion channels (4 transmembrane domains, a Cys-loop and loops A to F) and the 2 adjacent cysteines in the C-loop, defining *Hco*-DES-2H as a nAChR alpha subunit (Figure S2).

When comparing *Hco-des-2H* coding sequences (Table 2) obtained from *Hc*-CRA and *Hc*-CRA-AAD^M^, respectively *Hc*-Howick and *Hc*-Howick-AAD^M^, no mutation was found to correlate perfectly with AAD-susceptibility. Nevertheless, using the SL1 primer and 2 internal reverse primers (*Hco*-AcRa_rev3 and *Hco*-AcRa_rev2) in a nested PCR experiment, an insertion of 135 bp was detected in the 5′ UTR of the *Hco-des-2H* gene from the *Hc*-CRA AAD^M^ and *Hc*-Howick AAD^M^ isolates, creating 2 additional start codons. Both start codons are followed by an early stop codon in frame.

**Table 2:** Summary of *H. contortus* ACR genes and mutations occurring in the AAD mutant lines.

Gene	Source	Nature of mutation	gDNA (GenBank accessions)	cDNA (GenBank accessions)
*Hco-mptl-1*	*H. contortus* CRA	n.a.	FJ807291–293	FJ807280–282
			FJ807298–300	FJ807287–288
*Hco-mptl-1*	*H. contortus* Howick	n.a.	FJ807304–309	FJ807283–286
				FJ807314
*Hco-mptl-1*-m1	*H. contortus* CRA AAD^M^	Unknown mutation leads to loss of exons 2 and 3 in transript.	n.d.	FJ807289–290
*Hco-mptl-1*-m2	*H. contortus* CRA AAD^M^	Deletion of 323 bp (end of intron 3 and most of exon 4) leads to loss of exon 4 in transcript.	FJ807294–296	FJ807297
*Hco-mptl-1*-m3	*H. contortus* CRA AAD^M^	Loss of splice acceptor site in intron 14 (10 bp deletion, Figure 5) leads to loss of exon 15 in transcript.	FJ807301–303	FJ807310–312
*Hco-mptl-1*-m4	*H. contortus* CRA AAD^M^	Unknown mutation(s) leading to partial loss of exons 4 and 15 in transcript.	n.d.	FJ807313
*Hco-mptl-1*-m5	*H. contortus* Howick AAD^M^	Transversion in exon 6 from G to T that leads to a premature stop codon in transcript (Figure 6).	n.d.	FJ807315
*Hco-mptl-1*-m6	*H. contortus* Howick AAD^M^	Unknown mutation leads to loss of exon 8 in transcript.	n.d.	FJ807316
*Hco-des-2H*	*H. contortus* CRA	n.a.	n.d.	FJ807317–331
				FJ807336–340
*Hco-des-2H*	*H. contortus* Howick	n.a.	n.d.	FJ807332–335
				FJ807346–347
*Hco-des-2H*	*H. contortus* CRA AAD^M^	Insertion of 135 bp in the 5′ UTR creating 2 additional start codons.	n.d.	FJ807341–345
*Hco-des-2H*	*H. contortus* Howick AAD^M^	Insertion of 135 bp in the 5′ UTR creating 2 additional start codons.	n.d.	FJ807348–349
*Hco-deg-3H*	*H. contortus* CRA	n.a.	n.d.	FJ807350–356
*Hco-deg-3H*	*H. contortus* Howick	n.a.	n.d.	FJ807357–360

n.a. = not applicable; n.d. = not determined. The sequences are included in the file Text S1.

In the *C. elegans* genome, DES-2 and DEG-3 are encoded on the same operon and both subunits are co-expressed to form a functional channel [28],[29]. Performing RACE-PCR on *H. contortus* (adults) cDNA we identified *Hco-deg-3H* encoding a protein of 569 amino acids that shows 68.4% identity and 78% similarity to *C. elegans* DEG-3. Again, *Hco*-DEG-3H carried all the hallmarks of nAChR alpha subunits (Figure S3). No mutations were detected for *Hco-deg-3H* in the AAD-mutant *H. contortus* isolates compared to the parental isolates. The *Hco-deg-3H* mRNA carries a spliced leader type 2 (SL2) sequence at its 5′ end. To test whether *Hco-des-2H* and *Hco-deg-3H* are also on an operon in *H. contortus*, a long range PCR was performed using a forward primer designed at the end of *Hco-des-2H* (*Hco*-des2_frw11) and a reverse primer at the beginning of *Hco-deg-3H* (*Hco*-deg3_2r). A band of approximately 6 kb was obtained for the 4 isolates confirming that *Hco-des-2H* and *Hco-deg-3H* are encoded on a single operon. However, the distance between the 2 genes is 10 times larger in *H. contortus* than in *C. elegans*.

### Relative expression levels of *acr* genes in AAD mutant *Haemonchus contortus*

**Figure 7:**
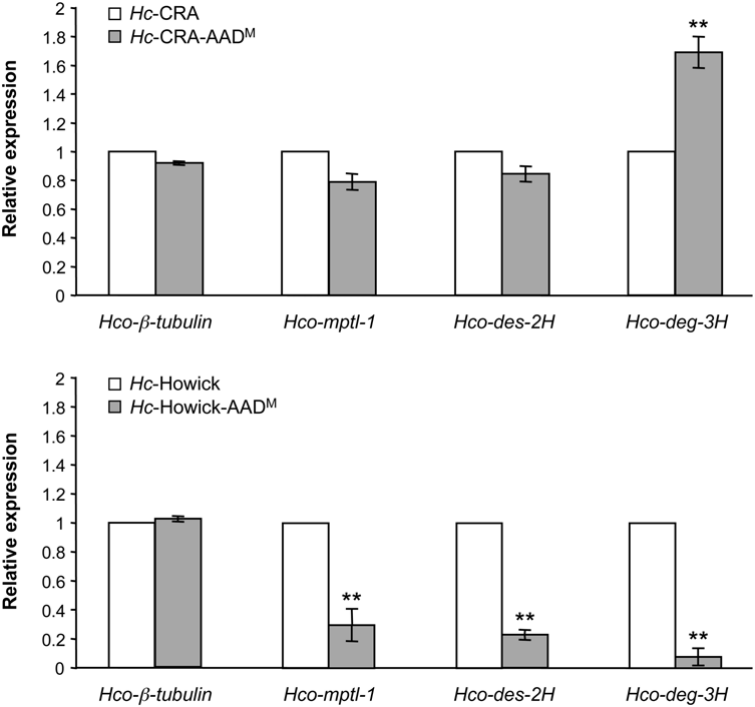
Relative mRNA levels of DEG-3 subfamily genes in AAD mutant *H. contortus*. Relative expression levels of the DEG-3 subfamily nAChR genes *Hco-mptl-1*, *Hco-des-2H*, and *Hco-deg-3H* quantified by RT-qPCR for *Hc*-CRA and *Hc*-CRA AAD^M^ (top), or *Hc*-Howick and *Hc*-Howick AAD^M^ isolates (bottom). Relative expression values were normalized to those of glucose-phosphate isomerase (GPI); ß-tubulin served as a non-affected control. P-values (<0.01 are indicated by **) were calculated with repeated measures Anova, followed by Dunnett's test against the parental control (which had been set to 1). Average mRNA levels and SD were derived from 3 independent experiments, each in duplicate with 1 qPCR run each.

The steady-state mRNA levels of the DEG-3 subfamily acetylcholine receptor genes *Hco-mptl-1*, *Hco-des-2H* and *Hco-deg-3H* were quantified by real-time PCR (Figure 7). For the *Hc*-CRA AAD^M^ isolate, a small, statistically not significant (p>0.05) decrease in the mRNA level was observed for *Hco-mptl-1* (−21%) and *Hco-des-2H* (−16%). In contrast, the relative mRNA level of the *Hco-deg-3H* gene was higher (69%; p<0.01) in this mutant. For *Hc*-Howick AAD^M^, a significant (p<0.01) down-regulation of the 3 measured DEG-3 subfamily members was observed: −70% for *Hco-mptl-1*, −77% for *Hco-des-2H* and −92% for *Hco-deg-3H*. The relative expression level of the β-tubulin gene was measured in both mutant isolates as a (presumably) non-affected control. No statistically significant changes were observed.

## Discussion

A new chemical class of synthetic anthelmintics, the AADs, was recently discovered [12]. The AADs exhibit excellent efficacy against various species of livestock-pathogenic nematodes and more importantly, can control nematodes resistant to the currently available anthelmintics [30],[31]. To get insights into the mode of action of the new AADs, a classical ‘forward genetic’ screen for AAD-resistant *C. elegans* mutants was performed previously [12]. As a result, AADs were proposed to act through the nAChR ACR-23, a member of the nematode-specific DEG-3 subfamily [32]. By screening the currently available (but incomplete) *H. contortus* genome sequence for DEG-3 nAChR homologues, it was found that this subfamily is conserved between *C. elegans* and *H. contortus*. Six paralogous proteins out of 8 in *C. elegans* or *C. briggsae* were identified (Figure 1), in contrast to only 2 in the genome of *B. malayi* [33]. The AADs possess a unique mode of action: the nAChR subunits involved in AAD action are different from those targeted by imidazothiazoles [34],[35] and there is no cross-resistance between the 2 chemical classes [12].

Two independent AAD-mutant *H. contortus* lines were used to screen for mutations in ACR genes of the DEG-3 subfamily. Two genes were found to be affected: The *H. contortus* des-2 homologue *Hco-des-2H*, where all AAD-mutant *H. contortus* carried an insertion in the 5′ UTR introducing 2 additional, out-of-frame start codons, and the gene *monepantel-1* (*Hco-mptl-1*), for which a panel of different mutations were detected in AAD-mutant (AAD^M^) *H. contortus*. Apart from 1 nonsense mutation discovered in *Hc*-Howick AAD^M^ nematodes (*Hco*-MPTL-1-m5; Figure 4), the detected mutations all involved mis-splicing resulting in loss of exon(s) from the mRNA as indicated by shortened reverse transcriptase-PCR products (Figure 3). This unusual mechanism has not been described before in *H. contortus*. In the genetic screen performed on AAD-resistant *C. elegans* [12], 2 mutants bearing a G-to-A transition of the conserved G nucleotide in the 3′ splice acceptor sites of either the second or third introns were described; these mutations are predicted to cause an increase in the size of the mRNA due to the lack of splicing of the affected intron. In another study [36], a single base pair change in the first intron of the lev-8 subunit gene was identified in a partially levamisole-resistant *C. elegans* mutant. This mutation leads to alternative splicing and introduction of a premature stop codon. In the case of mutations *Hco*-MPTL-1-m2 (loss of exon 4), *Hco*-MPTL-1-m3 (loss of exon 15) or *Hco*-MPTL-1-m6 (loss of exon 8), exon skipping creates a frame-shift that leads to a premature stop codon (Figure 4). These mutations, including the *Hco*-MPTL-1-m5 (stop codon) are predicted to result in a truncated, non-functional *Hco*-MPTL-1 protein and/or, if the mutant mRNA is recognized by the nonsense-mediated mRNA decay (NMD) machinery [37], degradation of the mRNA (some known genes of the NMD machinery in *C. elegans* have orthologues in the *H. contortus* genome; Rufener and Mäser, unpublished). Measuring the expression levels of the 3 DEG-3 subfamily genes *Hco-mptl-1*, *Hco-des-2H* and *Hco-deg-3H* in adult *H. contortus*, we found statistically significant differences in the steady state level of mRNA in AAD mutant worms. In the *Hc*-CRA AAD^M^ isolate, a significant increase of the *Hco-deg-3H* transcript was observed. A possible explanation may be that this compensates for the loss of the *Hco*-MPTL-1 subunit since no more full-length *Hco-mptl-1* transcript was detectable in Hc-CRA AAD^M^. In the case of *Hc*-Howick AAD^M^, all 3 nAchR genes were down-regulated compared to *Hc*-Howick. Although we cannot give a result-based explanation, we interpret the finding that the expression of DEG-3 subfamily nAChR genes is affected in *H. contortus* as further evidence for the involvement of these genes in AAD susceptibility.

The mutations *Hco*-MPTL-1-m1 (loss of exon 2 and 3) and Hco-MPTL-1-m4 (partial loss of exon 4 and 15) did not cause a frame-shift, but the loss of the signal peptide and the first 39 amino acids of the extracellular loop for the first mutation, and a truncated protein for the second mutation. Interestingly, 1 of the previously identified AAD-resistant *C. elegans* mutants also carried a mutation in the signal peptide of the Cel-ACR-23 protein [12]. Receptors are assembled in the endoplasmic reticulum (ER) [38] and interference with the signal peptide could result in mis-localization of the protein or in improper interactions with ER-resident, ACR-specific chaperones [25], [39]–[41]. Furthermore, it is known that the expression, assembly and transport to the surface of ACR subunits is subject to stringent quality controls that guarantee the functional competence of the final product [42]–[44]. Truncated nAChR proteins are likely to be targeted to the lysozyme and degraded.

In summary, we have detected a large number of different mutations affecting the *Hco-mptl-1* gene and transcript, respectively, from AAD mutant *H. contortus* (Table 2). For the benzimidazoles, a variety of different mutations in the target protein ß-tubulin are associated with drug resistance, 3 so far from *H. contortus* [15],[45],[46] and many more from phytopathogenic fungi [47]. These are point mutations, that are thought to interfere with benzimidazole binding while preserving microtubular function. The mutations have less drastic effects on the predicted protein than those described here for *Hco-mptl-1* of *H. contortus*. At present, we do not know whether *Hco-mptl-1* is an essential gene in *H. contortus*, but our findings indicate that it may not be. There were no mutations in common between *H. contortus* CRA-AAD^M^ and Howick-AAD^M^, indicating that the genetic screen was not saturated. However, for *Hco-des-2H*, an insertion of 135 bp creating 2 additional start codons was present in the 5′ UTR from both AAD^M^ isolates. While *Hco-des-2H* mRNA levels were significantly lower in *Hc*-Howick AAD^M^ (compared to *Hc*-Howick), no effect was observed on *Hco-des-2H* expression in *Hc*-CRA AAD^M^. It is interesting to note that in *C. elegans*, mutant worms lacking a functional DES-2 did not exhibit any AAD resistance [12]. The in vitro protocol used to breed AAD-mutant *H. contortus* is very focused using a large number of individuals and a border line subcurative exposure concentrations over extended time period. This protocol is different from the situation in the field, e.g. after multiple generations exposed to subcurative treatment in sheep, we have so far not been able to obtain AAD-resistant *H. contortus* (Pradervand and Kaminsky, unpublished data).

In conclusion, several independent mutations were found in the *Hco-mptl-1* gene from *H. contortus* mutants with reduced sensitivity for monepantel, implicating *Hco*-MPTL-1 as a likely target for AAD action against *H. contortus*. However, this hypothesis is difficult to test since *H. contortus* is not readily amenable to genetic manipulation [48]. The AADs are very well tolerated by sheep or cattle [14]. The absence of DEG-3 subfamily acetylcholine receptors in mammals might explain the selective toxicity of AADs to nematodes.

## Supporting Information

Figure S1 The full-length coding sequence of *Hco-mptl-1*. The N-terminal signal sequence is shown in blue, transmembrane domains (TMD) are shaded in grey and the hallmarks of nicotinic acetylcholine receptor α-subunits are highlighted in green. Exons 4 and 15 are underlined.
(1.22 MB TIF)

Figure S2 The full-length coding sequence of *Hco-des-2H*. The N-terminal signal sequence is shown in blue, transmembrane domains (TMD) are shaded in grey and the hallmarks of nicotinic acetylcholine receptor α-subunits are highlighted in green.
(1.17 MB TIF)

Figure S3 The full-length coding sequence of *Hco-deg-3H*. The N-terminal signal sequence is shown in blue, transmembrane domains (TMD) are shaded in grey and the hallmarks of nicotinic acetylcholine receptor α-subunits are highlighted in green.
(1.25 MB TIF)

Table S1 Primers used for PCR amplification of *deg-3* subfamily genes from *Haemonchus contortus*.
(0.07 MB DOC)

Text S1 All the sequences as submitted to GenBank.
(0.48 MB DOC)
