# Supplementary figures and images for: Haemonchus contortus Acetylcholine Receptors of the DEG-3 Subfamily and Their Role in Sensitivity to Monepantel
Source: PLoS Pathog. 2009 Apr 10;5(4):e1000380. doi: 10.1371/journal.ppat.1000380 (PMC2662886; doi:10.1371/journal.ppat.1000380)

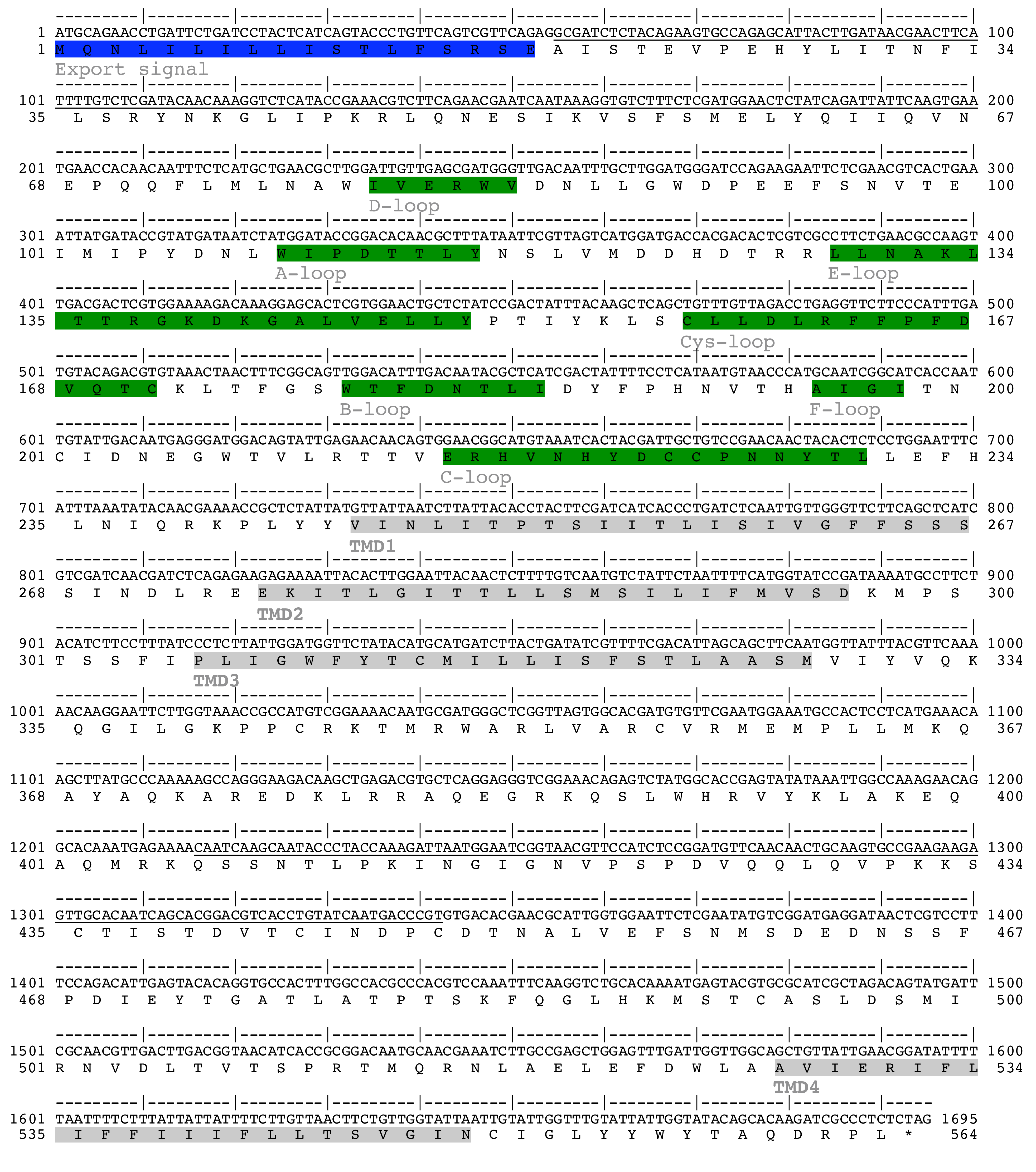

Supplement: Figure S1 — The full-length coding sequence of Hco-mptl-1. The N-terminal signal sequence is shown in blue, transmembrane domains (TMD) are shaded in grey and the hallmarks of nicotinic acetylcholine receptor α-subunits are highlighted in green. Exons 4 and 15 are underlined. (1.22 MB TIF) [file ppat.1000380.s001.tif]

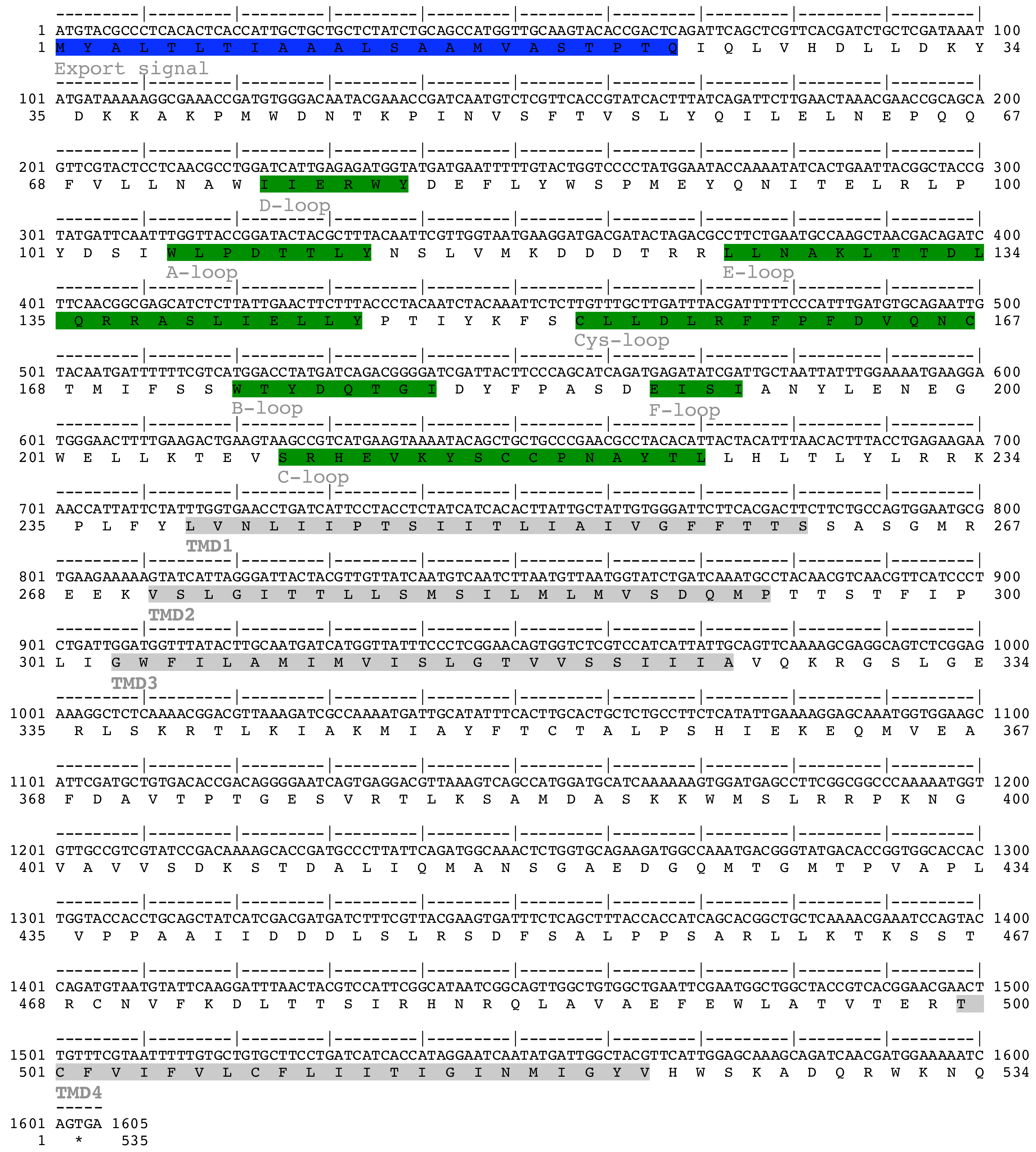

Supplement: Figure S2 — The full-length coding sequence of Hco-des-2H. The N-terminal signal sequence is shown in blue, transmembrane domains (TMD) are shaded in grey and the hallmarks of nicotinic acetylcholine receptor α-subunits are highlighted in green. (1.17 MB TIF) [file ppat.1000380.s002.tif]

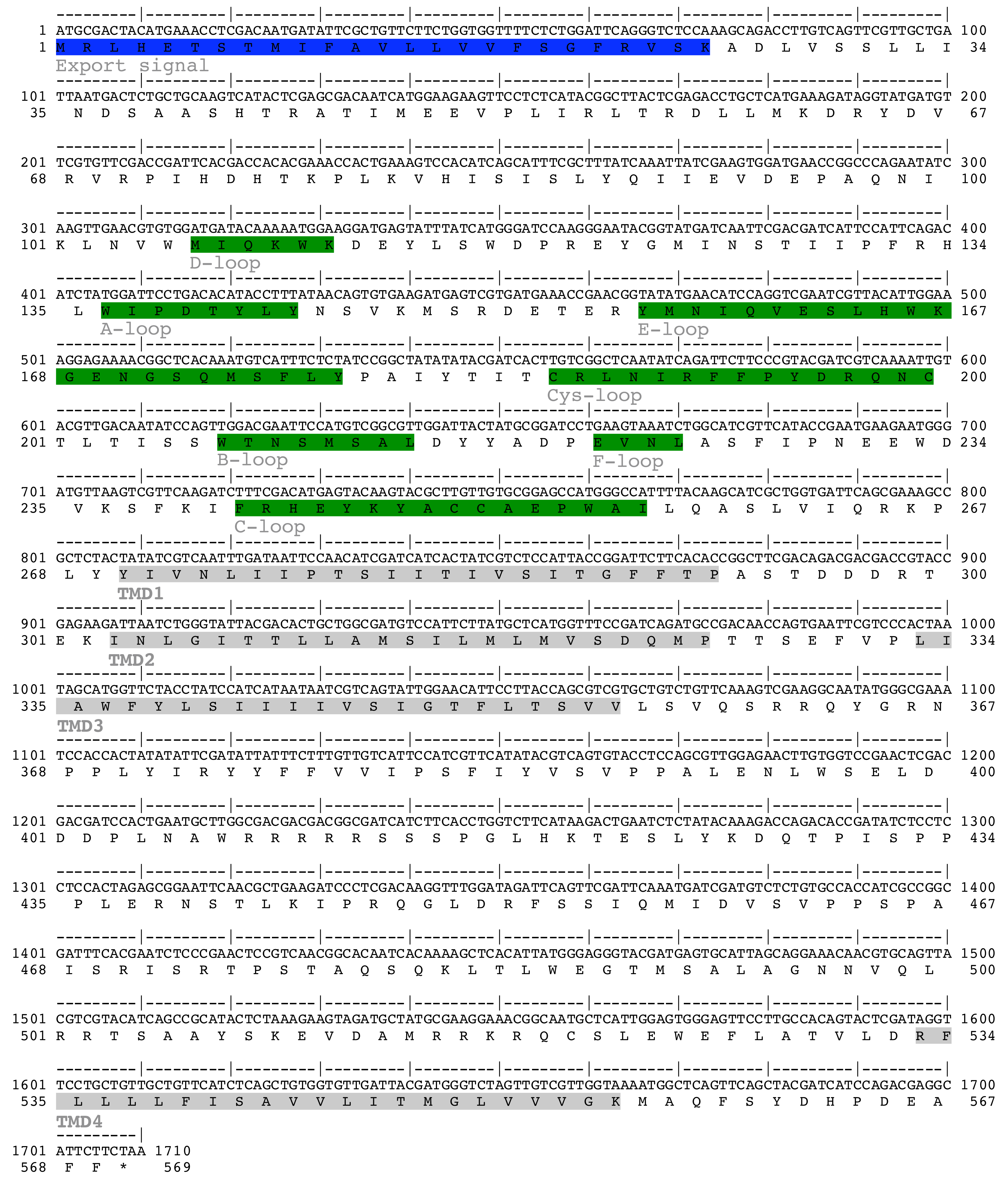

Supplement: Figure S3 — The full-length coding sequence of Hco-deg-3H. The N-terminal signal sequence is shown in blue, transmembrane domains (TMD) are shaded in grey and the hallmarks of nicotinic acetylcholine receptor α-subunits are highlighted in green. (1.25 MB TIF) [file ppat.1000380.s003.tif]
