# Supplementary material for: Haemonchus contortus Acetylcholine Receptors of the DEG-3 Subfamily and Their Role in Sensitivity to Monepantel
Source: PLoS Pathog. 2009 Apr 10;5(4):e1000380. doi: 10.1371/journal.ppat.1000380 (PMC2662886; doi:10.1371/journal.ppat.1000380)
Supplement: Table S1 — Primers used for PCR amplification of deg-3 subfamily genes from Haemonchus contortus. (0.07 MB DOC) [file ppat.1000380.s004.doc]

| **Primer name** | **Sequence 5’ → 3’** |
| --- | --- |
| *For PCR on gDNA or cDNA* |  |
| *Hco-mptl-1*_frw3 | CATGGATGACCACGACACTC |
| *Hco-mptl-1*_rev1 | AGATCGTTGATCGACGATGA |
| *Hco-mptl-1*_frw4 | TTTGTCTCGATACAACAAAGG |
| *Hco-mptl-1*-rev3 | CATTTGTGCCTGTTCTTTGG |
| *Hco-mptl-1*_frw6 | CTCGGTTAGTGGCACGATGT |
| *Hco-mptl-1*_rev6 | ATGCGCACGTACTCATTTTC |
| *Hco-des-2*_frw8 | CGTTCACGATCTGCTCGATA |
| *Hco-des-2*_rev8 | CAATTCTGCACATCGAATGG |
| *Hco-deg-3*_frw1 | AGTCGTGATGAGACCGAACG |
| *Hco-deg-3*_rev1 | ATTCACTGGTTGTCGGCATT |
| *Hco-mptl-1*_5’_frw1 | GTTTGAGGTTGTACCGCGATC |
| *Hco-mptl-1*_5’_frw3 | GAGGGCAACAATTTCCTTCA |
| *Hco-mptl-1*_3’end_rev1 | TGACTAGAGAGGGCGATCTTG |
| SL1 | GGTTTAATTACCCAAGTTTGAG |
| SL2 | GGTTTTAACCCAGTTACTCAAG |
| *Hco-mptl-1*_rev2 | ttccaagtgtaattttctcctctc |
| *Hco-mptl-1*_rev1 | AGATCGTTGATCGACGATGA |
| *Hco-mptl-1*_5’_frw2 | CAAATGGAAAATCCTAGCTCTGG |
| *Hco-mptl-1*_rev8 | GCGTTCAGCATGAGAAACTG |
| *Hco-mptl-1*_frw8 | AAGAGGGTCGGAAGCAGAGT |
| *Hco-mptl-1*_frw10/gDNA | CAATTGGGGACTCGAACCAG |
| *Hco-des-2*_frw11 | ATAATCGGCAATTGGCTGTG |
| *Hco-*AcRa_rev3 | CATTGATAACAACGTAGTAATACC |
| *Hco-*AcRa_rev2 | TCTCTTCACGCATTCCACTG |
| NheI_*des-2*_frw1 | GGCGGCTAGCCGCTCCTCCCCTACCTACTA |
| XhoI_*des-2*_rev1 | GGCGCTCGAGCATATCACTGATTTTTCCATCGTT |
| *Hco-deg-3*_2rev | ATTCTGACGATCGTACGGGA |
| *Hco-deg-3*_3rev | GCCATTTTACCAACCACCAC |
| NheI_*deg-3*_frw1 | GGCGGCTAGCATGCGACTACATGAAACCTCG |
| NotI_*deg-3*_rev1 | GGCGGCGGCCGCTTAGAAGAATGCCTCGTCTGG |

| *For real-time PCR* |  |
| --- | --- |
| *Hco-gpi*_RT_frw1 | GTACTTCACATTGCGCTTCG |
| *Hco-gpi*_RT_rev1 | CAGTCCAGGCTCCACTGATAA |
| *Hco-btub*_RT_frw1 | CCCGATGGAACATACAAAGG |
| *Hco-btub*_RT_rev1 | GTTCCAGGCTCGAGATCAAC |
| *Hco-mptl-1*_RT_frw1 | TGGTATCCGATAAAATGCCTTC |
| *Hco-mptl-1*_RT_rev1 | AACCATTGAAGCTGCTAACGTC |
| *Hco-des-2*_RT_frw2 | TCAAATGCCTACCACGTCAA |
| *Hco-des-2*_RT_rev2 | AAGACTGCCTCGCTTTTGAA |
| *Hco-deg-3*_RT_frw1 | TGGAATGCGTGAAGAGAAAG |
| *Hco-deg-3*_RT_rev1 | TGCAAGTATAAACCATCCAATCA |

**Supplementary Table 1.** Primers used for PCR amplification of *deg-3* subfamily genes from *Haemonchus contortus*.
