# Supplementary material for: Haemonchus contortus Acetylcholine Receptors of the DEG-3 Subfamily and Their Role in Sensitivity to Monepantel
Source: PLoS Pathog. 2009 Apr 10;5(4):e1000380. doi: 10.1371/journal.ppat.1000380 (PMC2662886; doi:10.1371/journal.ppat.1000380)
Supplement: Text S1 — All the sequences as submitted to GenBank. (0.48 MB DOC) [file ppat.1000380.s005.doc]

LOCUS Hco-monepantel-1;1 2105 bp mRNA linear INV 05-MAR-2009

DEFINITION [gene=Hco-mptl-1].

SOURCE Haemonchus contortus

ORGANISM Haemonchus contortus

Eukaryota; Metazoa; Nematoda; Chromadorea; Rhabditida; Strongylida;

Trichostrongyloidea; Haemonchidae; Haemonchinae; Haemonchus.

REFERENCE 1 (bases 1 to 2105)

AUTHORS Rufener,L., Maser,P., Roditi,I. and Kaminsky,R.

TITLE Haemonchus contortus Acetylcholine Receptors of the DEG-3 Subfamily

and their Role in Sensitivity to Monepantel

JOURNAL PLoS Pathogens, in press

REFERENCE 2 (bases 1 to 2105)

AUTHORS Rufener,L., Maser,P., Roditi,I. and Kaminsky,R.

TITLE Direct Submission

JOURNAL Submitted (05-MAR-2009) Institute of Cell Biology, University of

Bern, Baltzerstrasse 4, Bern, BE 3012, Switzerland

FEATURES Location/Qualifiers

source 1..2105

/organism="Haemonchus contortus"

/mol_type="mRNA"

/isolate="Hc-CRA"

/clone="wt-1"

/note="monepantel-sensitive isolate; wildtype gene"

CDS 348..2042

/note="Hco-MPTL-1"

/codon_start=1

/product="H. contortus Monepantel-1"

/translation="MQNLILILLISTLFSRSEAISTEVPEHYLITNFILSRYNKGLIP

KRLQNESIKVSFSMELYQIIQVNEPQQFLMLNAWIVERWVDNLLGWDPEEFSNVTEIM

IPYDNLWIPDTTLYNSLVMDDHDTRRLLNAKLTTRGKDKGALVELLYPTIYKLSCLLD

LRFFPFDVQTCKLTFGSWTFDNTLIDYFPHNVTHAIGITNCIDNEGWTVLRTTVERHV

NHYDCCPNNYTLLEFHLNIQRKPLYYVINLITPTSIITLISIVGFFSSSSINDLREEK

ITLGITTLLSMSILIFMVSDKMPSTSSFIPLIGWFYTCMILLISFSTLAASMVIYVQK

QGILGKPPCRKTMRWARLVARCVRMEMPLLMKQAYAQKAREDKLRRAQEGRKQSLWHR

VYKLAKEQAQMRKQSSNTLPKINGIGNVPSPDVQQLQVPKKSCTISTDVTCINDPCDT

NALVEFSNMSDEDNSSFPDIEYTGATLATPTSKFQGLHKMSTCASLDSMIRNVDLTVT

SPRTMQRNLAELEFDWLAAVIERIFLIFFIIIFLLTSVGINCIGLYYWYTAQDRPL"

BASE COUNT 640 a 474 c 418 g 573 t

ORIGIN

1 ggcacgaggc ggcacgaggc aagtttgagg ttgtaccgcg atcttcacaa atggaaaatc

61 ctagctctgg aacttgatat tcgcctgaaa atttgtggtt agaagacacg tcagttcact

121 caagcctacc cggtgtaaac aacaaaacct attcgtttca accagatcca aagccatttt

181 taggcatcgc aacgacatat ctcttctctc agtggttccc ttcgacaacc agaccaggag

241 gcggtccgag ggcaacaatt tccttcaccc caacggaaca gcgcctcaca aaccaacacc

301 catctgtgtc agcagcaatc agacgacgaa aacaccaaac gaccatcatg cagaacctga

361 ttctgatcct actcatcagt accctgttca gtcgttcaga ggcgatctct acagaagtgc

421 cagagcatta cttgataacg aacttcattt tgtctcgata caacaaaggt ctcataccga

481 aacgtcttca gaacgaatca ataaaggtgt ctttctcgat ggaactctat cagattattc

541 aagtgaatga accacaacaa tttctcatgc tgaacgcttg gattgttgag cgatgggttg

601 acaatttgct tggatgggat ccagaagaat tctcgaacgt cactgaaatt atgataccgt

661 atgataatct atggataccg gacacaacgc tttataattc gttagtcatg gatgaccacg

721 acactcgtcg ccttctgaac gccaagttga cgactcgtgg aaaagacaaa ggagcactcg

781 tggaactgct ctatccgact atttacaagc tcagctgttt gttagacctg aggttcttcc

841 catttgatgt acagacgtgt aaactaactt tcggcagttg gacatttgac aatacgctca

901 tcgactattt tcctcataat gtaacccatg caatcggcat caccaattgt attgacaatg

961 agggatggac agtattgaga acaacagtgg aacggcatgt aaatcactac gattgctgtc

1021 cgaacaacta cactctcctg gaatttcatt taaatataca acgaaaaccg ctctattatg

1081 ttattaatct tattacacct acttcgatca tcaccctgat ctcaattgtt gggttcttca

1141 gctcatcgtc gatcaacgat ctcagagaag agaaaattac acttggaatt acaactcttt

1201 tgtcaatgtc tattctaatt ttcatggtat ccgataaaat gccttctaca tcttccttta

1261 tccctcttat tggatggttc tatacatgca tgatcttact gatatcgttt tcgacattag

1321 cagcttcaat ggttatttac gttcaaaaac aaggaattct tggtaaaccg ccatgtcgga

1381 aaacaatgcg atgggctcgg ttagtggcac gatgtgttcg aatggaaatg ccactcctca

1441 tgaaacaagc ttatgcccaa aaagccaggg aagacaagct gagacgtgct caggagggtc

1501 ggaaacagag tctatggcac cgagtatata aattggccaa agaacaggca caaatgagaa

1561 aacaatcaag caatacccta ccaaagatta atggaatcgg taacgttcca tctccggatg

1621 ttcaacaact gcaagtgccg aagaagagtt gcacaatcag cacggacgtc acctgtatca

1681 atgacccgtg tgacacgaac gcattggtgg aattctcgaa tatgtcggat gaggataact

1741 cgtcctttcc agacattgag tacacaggtg ccactttggc cacgcccacg tccaaatttc

1801 aaggtctgca caaaatgagt acgtgcgcat cgctagacag tatgattcgc aacgttgact

1861 tgacggtaac atcaccgcgg acaatgcaac gaaatcttgc cgagctggag tttgattggt

1921 tggcagctgt tattgaacgg atatttttaa ttttctttat tattattttc ttgttaactt

1981 ctgttggtat taattgtatt ggtttgtatt attggtatac agcacaagat cgccctctct

2041 agtcaacttt aataaattgt acttagataa attaattgga gttcaaaaaa aaaaaaaaaa

2101 aaaaa

//

LOCUS Hco-monepantel-1;2 2105 bp mRNA linear INV 05-MAR-2009

DEFINITION [gene=Hco-mptl-1].

SOURCE Haemonchus contortus

ORGANISM Haemonchus contortus

Eukaryota; Metazoa; Nematoda; Chromadorea; Rhabditida; Strongylida;

Trichostrongyloidea; Haemonchidae; Haemonchinae; Haemonchus.

REFERENCE 1 (bases 1 to 2105)

AUTHORS Rufener,L., Maser,P., Roditi,I. and Kaminsky,R.

TITLE Haemonchus contortus Acetylcholine Receptors of the DEG-3 Subfamily

and their Role in Sensitivity to Monepantel

JOURNAL PLoS Pathogens, in press

REFERENCE 2 (bases 1 to 2105)

AUTHORS Rufener,L., Maser,P., Roditi,I. and Kaminsky,R.

TITLE Direct Submission

JOURNAL Submitted (05-MAR-2009) Institute of Cell Biology, University of

Bern, Baltzerstrasse 4, Bern, BE 3012, Switzerland

FEATURES Location/Qualifiers

source 1..2105

/organism="Haemonchus contortus"

/mol_type="mRNA"

/isolate="Hc-CRA"

/clone="wt-2"

/note="monepantel-sensitive isolate; wildtype gene"

CDS 348..2042

/note="Hco-MPTL-1"

/codon_start=1

/product="H. contortus Monepantel-1"

/translation="MQNLILILLISTLFSRSEAISTEVPEHYLITNFILSRYNKGLIP

KRLRNESIKVSFSMELYQIIQVNEPQQFLMLNAWIVERWVDNLLGWDPEEFSNVTEIM

IPYDNLWIPDTTLYNSLVMDDHDTRRLLNAKLTTRGKDKGALVELLYPTIYKLSCLLD

LRFFPFDVQTCKLTFGSWTFDNTLIDYFPHNVTHAIGITNCIDNEGWTVLRTTVERHV

NHYDCCPNNYTLLEFHLNIQRKPLYYVINLITPTSIITLISIVGFFSSSSINDLREEK

ITLGITTLLSMSILIFMVSDKMPSTSSFIPLIGWFYTCMILLISFSTLAASMVIYVQK

QGILGKPPCRKTMRWARLVARCVRMEMPLLMKQAYAQKAREDKLRRAQEGRKQSLWHR

VYKLAKEQAQMRKQSSNTLPKINGIGNVPSPDVQQLQVPKKSCTISTDVTCINDPCDT

NALVEFSNMSDEDNSSFPDIEYTGATLATPTSKFQGLHKMSTCASLDSMIRNVDLTVT

SPRTMQRNLAELEFDWLAAVIERIFLIFFIIIFLLTSVGINCIGLYYWYTAQDRPL"

BASE COUNT 640 a 474 c 418 g 573 t

ORIGIN

1 ggcacgaggc ggcacgaggc aagtttgagg ttgtaccgcg atcttcacaa atggaaaatc

61 ctagctctgg aacttgatat tcgcctgaaa atttgtggtt agaagacacg tcagttcact

121 caagcctacc cggtgtaaac aacaaaacct attcgtttca accagatcca aagccatttt

181 taggcatcgc aacgacatat ctcttctctc agtggttccc ttcgacaacc agaccaggag

241 gcggtccgag ggcaacaatt tccttcaccc caacggaaca gcgcctcaca aaccaacacc

301 catctgtgtc agcagcaatc agacgacgaa aacaccaaac gaccatcatg cagaacctga

361 ttctgatcct actcatcagt accctgttca gtcgttcaga ggcgatctct acagaagtgc

421 cagagcatta cttgataacg aacttcattt tgtctcgata caacaaaggt ctcataccga

481 aacgtcttcg aaacgaatca ataaaggtgt ctttctcgat ggaactctat cagattattc

541 aagtgaatga accacaacaa tttctcatgc tgaacgcttg gattgttgag cgatgggttg

601 acaatttgct tggatgggat ccagaagaat tctcgaacgt cactgaaatt atgataccgt

661 atgataatct atggataccg gacacaacgc tttataattc gttagtcatg gatgaccacg

721 acactcgtcg ccttctgaac gccaagttga cgactcgtgg aaaagacaaa ggagcactcg

781 tggaactgct ctatccgact atttacaagc tcagctgttt gttagacctg aggttcttcc

841 catttgatgt acagacgtgt aaactaactt tcggcagttg gacatttgac aatacgctca

901 tcgactattt tcctcataat gtaacccatg caatcggcat caccaattgt attgacaatg

961 agggatggac agtattgaga acaacagtgg aacggcatgt aaatcactac gattgctgtc

1021 cgaacaacta cactctcctg gaatttcatt taaatataca acgaaaaccg ctctattatg

1081 ttattaatct tattacacct acttcgatca tcaccctgat ctcaattgtt gggttcttca

1141 gctcatcgtc gatcaacgat ctcagagaag agaaaattac acttggaatt acaactcttt

1201 tgtcaatgtc tattctaatt ttcatggtat ccgataaaat gccttctaca tcttccttta

1261 tccctcttat tggatggttc tatacatgca tgatcttact gatatcgttt tcgacattag

1321 cagcttcaat ggttatttac gttcaaaaac aaggaattct tggtaaaccg ccatgtcgga

1381 aaacaatgcg atgggctcgg ttagtggcac gatgtgttcg aatggaaatg ccactcctca

1441 tgaaacaagc ttatgcccaa aaagccaggg aagacaagct gagacgtgct caggagggtc

1501 ggaaacagag tctatggcac cgagtatata aattggccaa agaacaggca caaatgagaa

1561 aacaatcaag caatacccta ccaaagatta atggaatcgg taacgttcca tctccggatg

1621 ttcaacaact gcaagtgccg aagaagagtt gcacaatcag cacggacgtc acctgtatca

1681 atgacccgtg tgacacgaac gcattggtgg aattctcgaa tatgtcggat gaggataact

1741 cgtcctttcc agacattgag tacacaggtg ccactttggc cacgcccacg tccaaatttc

1801 aaggtctgca caaaatgagt acgtgcgcat cgctagacag tatgattcgc aacgttgact

1861 tgacggtaac atcaccgcgg acaatgcaac gaaatcttgc cgagctggag tttgattggt

1921 tggcagctgt tattgaacgg atatttttaa ttttctttat tattattttc ttgttaactt

1981 ctgttggtat taattgtatt ggtttgtatt attggtatac agcacaagat cgccctctct

2041 agtcaacttt aataaattgt acttagataa attaattgga gttcaaaaaa aaaaaaaaaa

2101 aaaaa

//

LOCUS Hco-monepantel-1;3 2018 bp mRNA linear INV 05-MAR-2009

DEFINITION [gene=Hco-mptl-1].

SOURCE Haemonchus contortus

ORGANISM Haemonchus contortus

Eukaryota; Metazoa; Nematoda; Chromadorea; Rhabditida; Strongylida;

Trichostrongyloidea; Haemonchidae; Haemonchinae; Haemonchus.

REFERENCE 1 (bases 1 to 2018)

AUTHORS Rufener,L., Maser,P., Roditi,I. and Kaminsky,R.

TITLE Haemonchus contortus Acetylcholine Receptors of the DEG-3 Subfamily

and their Role in Sensitivity to Monepantel

JOURNAL PLoS Pathogens, in press

REFERENCE 2 (bases 1 to 2018)

AUTHORS Rufener,L., Maser,P., Roditi,I. and Kaminsky,R.

TITLE Direct Submission

JOURNAL Submitted (05-MAR-2009) Institute of Cell Biology, University of

Bern, Baltzerstrasse 4, Bern, BE 3012, Switzerland

FEATURES Location/Qualifiers

source 1..2018

/organism="Haemonchus contortus"

/mol_type="mRNA"

/isolate="Hc-CRA"

/clone="wt-3"

/note="monepantel-sensitive isolate; wildtype gene"

CDS 321..2015

/note="Hco-MPTL-1"

/codon_start=1

/product="H. contortus Monepantel-1"

/translation="MQNLILILLISTLFSRSEAISTEVPEHFLITNFILSRYNKGLIP

KRLQNESIKVSFSMELYQIIQVNEPQQFLMLNAWIVERWVDNLLGWDPEEFSNVTEIM

IPYDNLWIPDTTLYNSLVMDDHDTRRLLNAKLTTRGKDKGALVELLYPTIYKLSCLLD

LRFFPFDVQTCKLTFGSWTFDNTLIDYFPHNVTHAIGITNCIDNEGWTVLRTTVERHV

NHYDCCPNNYTLLEFHLNIQRKPLYYVINLITPTSIITLISIVGFCSSSSINDLREEK

ITLGITTLLSMSILIFMVSDKMPSTSSFIPLIGWFYACMILLISFSTLAASMVIYVQK

QGILGKPPCRKTMRWARLVARCVRMEMPLLMKQAYAQKAREDKLRRAQEGRKQSLWHR

VYKLAKEQAQMRKQSSNTLPKTNGIGNVPSPDVQQLQVPKKSCTISTDVTCINDPCDT

NALVEFSNMSDEDNSSFPDIEYTGATLATPTSKFQGLHKMSTCASLDSMIRNVDLTVT

SPRTMQRNLAELEFDWLAAVIERIFLIFFIIIFLLTSVGINCIGLYYWYTAQDRPL"

BASE COUNT 589 a 466 c 408 g 555 t

ORIGIN

1 gtttgcgatt gtaccgcgat cttcacaaat ggaaaatcct agctctggaa cttgatatag

61 cctgaaaatt tgtggttaga agacacgtca gttcactcaa gcctacccgg tgtaaacaac

121 aaaacttatt cgtttcaacc agatccaaag ccatttttag gcatcgcaac gacatatctc

181 ttctctcagt ggttcccttc gacaaccaga ccaggaggcg gtccgagggc aacaatttcc

241 ttcaccccaa cggaacaacg cctcaccaac acccatctgt gtcagcagtg aacagacgac

301 ggaaacacca aacgaccatc atgcagaacc tgattctgat cctactcatc agtaccctgt

361 tcagccgctc agaggcgatc tctacagaag tgccagagca tttcttgata acgaacttca

421 ttttgtctcg atacaacaaa ggtctcatac caaaacgtct tcagaacgaa tcaataaagg

481 tgtctttctc gatggaactc tatcagatta ttcaagtgaa tgaaccacag cagtttctca

541 tgctgaacgc ttggattgtt gagcgatggg ttgacaattt gcttggatgg gatccagaag

601 aattctcgaa cgtcactgaa attatgatac cgtatgataa tctatggata ccggacacaa

661 cgctttataa ttcgttagtc atggatgacc acgacactcg tcgccttctg aacgccaagt

721 tgacgactcg tggaaaagac aaaggagcac tcgtggaact gctctatccg actatttata

781 agctcagctg tttgttagac ctgaggttct tcccatttga tgtacagacg tgtaaactaa

841 ctttcggcag ttggacattt gacaatacgc tcatcgacta tttccctcat aatgtgactc

901 atgctatcgg cattaccaat tgtattgaca atgagggatg gaccgtattg agaacaacag

961 tggaacggca tgtaaatcac tacgattgct gtccgaacaa ctacactctc ctggaatttc

1021 atttgaatat acaacgaaaa ccgctctatt atgttattaa tcttattaca cctacttcga

1081 tcatcaccct gatctcaatt gttgggttct gcagctcatc gtcgatcaac gatctcagag

1141 aagagaaaat tacacttgga attacaactc ttttgtcaat gtctattcta attttcatgg

1201 tatccgataa aatgccttct acatcttcct ttatccctct tattggatgg ttctatgcat

1261 gcatgatctt actgatatcg ttttcgacat tagcagcttc aatggttatt tacgttcaaa

1321 aacaagggat tcttggtaaa ccgccatgtc ggaaaacaat gcgatgggct cggttagtgg

1381 cgcgatgtgt tcgaatggaa atgccactcc tcatgaaaca agcttatgcc caaaaagcca

1441 gggaagacaa actaagacgt gctcaagagg gtcgaaagca gagtctatgg caccgagtat

1501 acaaattggc caaagaacag gcacaaatga gaaaacagtc aagcaatacc ctgccaaaga

1561 ctaatggaat cgggaacgtt ccatctccgg atgttcaaca actacaagtg ccgaagaaga

1621 gttgcacaat cagcacggac gtcacctgca tcaatgaccc gtgtgacacg aacgcattgg

1681 tggaattctc aaatatgtcg gatgaggaca actcgtcctt tcccgacatc gagtacacag

1741 gagccacttt ggccacgccc acgtccaaat ttcaaggtct gcacaaaatg agtacgtgcg

1801 catcgttgga cagtatgatt cgcaatgttg atttgaccgt aacatcaccg cggacaatgc

1861 aacgaaatct tgccgaactg gagtttgatt ggttggcagc tgttattgaa cggatatttt

1921 tgattttctt tattattatt tttttgttaa cttctgttgg tattaattgt attggtttgt

1981 attattggta tacagcacaa gatcgccctc tctagtca

//

LOCUS Hco-monepantel-1;4 2000 bp mRNA linear INV 05-MAR-2009

DEFINITION [gene=Hco-mptl-1].

SOURCE Haemonchus contortus

ORGANISM Haemonchus contortus

Eukaryota; Metazoa; Nematoda; Chromadorea; Rhabditida; Strongylida;

Trichostrongyloidea; Haemonchidae; Haemonchinae; Haemonchus.

REFERENCE 1 (bases 1 to 2000)

AUTHORS Rufener,L., Maser,P., Roditi,I. and Kaminsky,R.

TITLE Haemonchus contortus Acetylcholine Receptors of the DEG-3 Subfamily

and their Role in Sensitivity to Monepantel

JOURNAL PLoS Pathogens, in press

REFERENCE 2 (bases 1 to 2000)

AUTHORS Rufener,L., Maser,P., Roditi,I. and Kaminsky,R.

TITLE Direct Submission

JOURNAL Submitted (05-MAR-2009) Institute of Cell Biology, University of

Bern, Baltzerstrasse 4, Bern, BE 3012, Switzerland

FEATURES Location/Qualifiers

source 1..2000

/organism="Haemonchus contortus"

/mol_type="mRNA"

/isolate="Hc-Howick"

/clone="wt-4"

/note="monepantel-sensitive isolate; wildtype gene"

CDS 303..1997

/note="Hco-MPTL-1"

/codon_start=1

/product="H. contortus Monepantel-1"

/translation="MQNLILILLISTLFSRSEAISTEVPEHYLITNFILSRYNKGLIP

KRLQNESIKVSFSMELYQIIQVNEPQQFLMLNAWIVERWVDNLLGWDPEEFSNVTEIM

IPYDNLWIPDTTLYNSLVMDDHDTRRLLNAKLTTRGKDKGALVGLLYPTIYKLSCLLD

LRFFPFDVQTCKLTFGSWTLDNTLIDYFPHNVTHAIGITNCIDNEGWTVLRTTVERHV

NHYDCCPNNYTLLEFHLNIQRKPLYYVINLITPTSIITLISIVGLFSSSSINDLREEK

ITLGITTLLSMSILIFMVSDKMPSTSSFIPLIGWFYTCMILLISFSTLAASMVIYVQK

QGILGKPPCRKTMRWARLVARCVRMEMPLLMKQAYAQKAREDKLRRAQEGRKQSLWHR

VYKLAKEQAQMRKQSSNTLPKINGIGNVPSPDVQQLQVPKKSCTISTDVTCINDPCDT

NALAEFSNMSDEDNSSFPDIEYTGATLATPTSKFQGLHKVSTCASLDSMIRNVDLTVT

SPRTMQRNLAELEFDWLAAVIERIFLIFFIIIFLLTSVGINCIGLYYWYTAQDRPL"

BASE COUNT 596 a 467 c 401 g 536 t

ORIGIN

1 caaatggaaa atcctagctc tggaacttga tattcgcctg aaaatttgtg gttagaagac

61 actccagttc gctacagcct acccggtgta aacaaagcga agcctatttg attcgaccgg

121 attcgaagtc atttttagac atcgcaacga catatctctt ctctcagtgg ttcccttcga

181 caaccagacc aggaggcggt ccgagggcaa caatttcctt catcccaacg gaacagcgcc

241 tcaccaacca acacccatct gtgtcagcag caatcagacg acgaaaacac caaacgacca

301 tcatgcagaa cctgattctg atcctactca tcagtaccct gttcagtcgt tcagaagcga

361 tctctacaga agtgccagag cattatttga taacgaactt cattttgtcc cgatacaaca

421 aaggtctcat accgaaacgt cttcaaaacg aatcaataaa ggtgtctttc tccatggaac

481 tctatcagat tattcaagtg aatgaaccac aacagtttct catgcttaac gcctggatcg

541 ttgagcgatg ggttgacaat ttgctcggat gggatcctga agaattctcg aacgtcactg

601 aaattatgat accgtacgat aatctatgga taccggatac gacgctttat aattcgttgg

661 tcatggatga ccacgacact cgtcgccttt tgaacgcaaa gttaacgact cgtggaaaag

721 acaaaggagc acttgtggga ttgctctatc cgactattta caagctcagc tgtttgctag

781 acctgaggtt cttcccattt gatgtacaga cgtgtaaact aactttcggc agttggacac

841 ttgacaatac gctcatcgac tattttcctc ataatgtaac acatgcaatc ggcatcacca

901 actgtattga caatgaggga tggaccgtat tgagaacaac agtagaacga catgtaaatc

961 actacgattg ctgtccgaac aactacactc ttctggaatt ccatttaaat atacaacgaa

1021 aaccgctcta ttatgtcatt aatcttatta cacctacttc gatcatcacc ctgatctcaa

1081 ttgttgggct tttcagctca tcgtcgatca acgatctcag agaggagaaa attacacttg

1141 gaattacaac actattgtca atgtctatat taatctttat ggtatccgat aaaatgcctt

1201 ctacatcctc cttcattcct cttattggat ggttttatac gtgcatgatt ttactgatat

1261 cgttttcgac gttagcagct tcaatggtta tttacgtaca gaaacaaggg attcttggta

1321 aaccaccctg tcggaaaaca atgcgatggg ctcggttagt ggcacgatgt gtccgaatgg

1381 aaatgccact tcttatgaaa caagcttatg cacagaaagc aagggaagac aagctaagac

1441 gtgcccaaga gggtcggaaa cagagcctat ggcaccgagt atacaaattg gccaaagaac

1501 aggcacaaat gagaaaacaa tcaagcaaca cactaccaaa gattaatgga atcggaaacg

1561 ttccatcgcc agatgttcag caactacaag tgccaaagaa gagttgcaca atcagcacgg

1621 acgtcacctg catcaatgac ccgtgtgaca cgaacgcatt ggcggaattc tcgaatatgt

1681 cggatgagga caactcgtcc tttcccgaca tcgagtacac aggagccacg ttggccacgc

1741 ccacgtccaa atttcaaggt ctgcacaaag tgagtacgtg cgcatcgcta gacagtatga

1801 ttcgcaacgt tgacttgacc gtaacatcac cgcggacaat gcaacgaaat cttgccgagc

1861 tggagtttga ttggttggca gctgttattg aacggatatt tttaattttc tttattatta

1921 ttttcttgtt aacttctgtt ggtattaatt gtattggttt gtattattgg tatacagcac

1981 aagatcgccc tctctagtca

//

LOCUS Hco-monepantel-1;5 1995 bp mRNA linear INV 05-MAR-2009

DEFINITION [gene=Hco-mptl-1].

SOURCE Haemonchus contortus

ORGANISM Haemonchus contortus

Eukaryota; Metazoa; Nematoda; Chromadorea; Rhabditida; Strongylida;

Trichostrongyloidea; Haemonchidae; Haemonchinae; Haemonchus.

REFERENCE 1 (bases 1 to 1995)

AUTHORS Rufener,L., Maser,P., Roditi,I. and Kaminsky,R.

TITLE Haemonchus contortus Acetylcholine Receptors of the DEG-3 Subfamily

and their Role in Sensitivity to Monepantel

JOURNAL PLoS Pathogens, in press

REFERENCE 2 (bases 1 to 1995)

AUTHORS Rufener,L., Maser,P., Roditi,I. and Kaminsky,R.

TITLE Direct Submission

JOURNAL Submitted (05-MAR-2009) Institute of Cell Biology, University of

Bern, Baltzerstrasse 4, Bern, BE 3012, Switzerland

FEATURES Location/Qualifiers

source 1..1995

/organism="Haemonchus contortus"

/mol_type="mRNA"

/isolate="Hc-Howick"

/clone="wt-5"

/note="monepantel-sensitive isolate; wildtype gene"

CDS 298..1992

/note="Hco-MPTL-1"

/codon_start=1

/product="H. contortus Monepantel-1"

/translation="MQNLILILLISTLFSRSEAISTEVPEHYLITNFILSRYNKGLIP

KRLQNESIKVSFSMELYQIIQVNEPQQFLMLNAWIVERWVDNLLGWDPEEFSNVTEIM

IPYDNLWIPDTTLYNSLVMDDHDTRRLLNAKLTTRGKDKGALVELLYPTIYKLSCLLD

LRFFPFDVQTCKLTFGSWTFDNTLIDYFPHNVTHAIGITNCIDNEGWTVLRTTVERHV

NHYDCCPNNYTLLEFHLNIQRKPLYYVINLITPTSIITLISIVGFFSSSSINDLREEK

ITLGITTLLSMSILIFMVSDKMPSTSSFIPLIGWFYTCMILLISFSTLAASMVIYVQK

QGILGKPPCRKTMRWARLVARCVRMEMPLLMKQAYAQKAREDKLRRAQEGRKQSLWHR

VYKLAKEQAQMRKQSSNTLPKINGIGNVPSPDVQQLQVPKKSCTISTDVTCINDPCGT

NALVEFSNMSDEDNSSFPDIEYTGATLTTPTSKFQGLHKMSTCASLDSMIRNVDLTVT

SPRTMQRNLAELEFDWLAAVIERIFLIFFIIIFLLTSVGINCIGLYYWYTAQDLPL"

BASE COUNT 594 a 459 c 395 g 547 t

ORIGIN

1 caaatggaaa atcctagctc tggaacttga tatcgcctga aaatttgtgg ttggaagaca

61 cgccagttcg tccaggtcta cccggtgtaa acaaagcaaa acctattcaa atcaaccaga

121 tccaaagcca tttttaggca tcgcaacgac atctctcttc tcacagtggt tcccttcgac

181 aaccagacca ggaggcggtc cgagggcaac aatttccttc accccaacgg aacaacgcct

241 caccaacacc catctgtgtc agcagtgatc agacgacgaa aacaccaaac gaccatcatg

301 caaaacctga ttctgatcct actcatcagt acactattca gtcgttcaga agcgatctct

361 acagaagtgc cagagcatta tttgataacg aacttcattt tgtcccgata caacaaaggt

421 ctcataccga aacgtcttca gaacgaatca ataaaggtgt ctttctcaat ggaactctac

481 cagattattc aagtgaatga accacaacag tttctcatgc ttaacgcctg gatcgttgag

541 cgatgggttg acaatttgct tggatgggat cctgaagaat tctcgaacgt cactgaaatt

601 atgataccgt atgataatct atggataccg gatacgacgc tttataattc gttagtcatg

661 gatgaccacg acactcgtcg ccttctgaac gccaagttga cgactcgtgg aaaagacaaa

721 ggagcactcg tggaactgct ctatccgact atttacaagc tcagctgttt gttagacctg

781 aggttcttcc catttgacgt acagacgtgt aaactaactt tcggcagttg gacatttgac

841 aatacgctca tcgactattt ccctcataat gtgactcatg ctatcggcat taccaattgt

901 attgacaatg agggatggac cgtattgaga acaacagtgg aacggcatgt aaatcactac

961 gattgctgtc cgaacaacta cactctcctg gaatttcatt tgaatataca gcgaaaaccg

1021 ctctattatg ttattaatct tattacaccc acttcgatca tcaccctgat ctcaattgtt

1081 ggtttcttca gctcatcgtc gatcaacgat ctcagagaag agaaaattac acttggaatt

1141 acaactcttt tgtcaatgtc tattctaatt ttcatggtat ccgataaaat gccttctaca

1201 tcttccttta tccctcttat tggatggttc tatacatgca tgatcttact gatatcgttt

1261 tcgacattag cagcttcaat ggttatttac gttcaaaaac aagggattct tggtaaaccg

1321 ccttgtcgaa aaacaatgcg atgggctcga ctagttgcac gatgtgtccg aatggaaatg

1381 ccacttctaa tgaaacaagc ttatgcacaa aaagccaggg aagacaagct gagacgtgct

1441 caggagggac ggaagcagag tctatggcat cgagtatata aattggccaa agaacaggca

1501 caaatgagaa aacaatcaag caatacccta ccaaagatta atggaatcgg gaacgttcca

1561 tctccggatg ttcaacaact acaagtgccg aagaaaagtt gcacaatcag cacggacgtc

1621 acctgtatca atgacccgtg tggcacgaac gcattggtgg aattctcgaa tatgtcggat

1681 gaggataact cgtcttttcc agacatcgag tacacaggag ccactctaac cacgcccacg

1741 tctaaatttc aagggctgca caaaatgagt acgtgcgcat cgttggacag tatgattcgc

1801 aatgttgact tgaccgtaac atcaccgcgg acaatgcaac ggaatcttgc cgaactggag

1861 tttgattggt tggcagctgt tattgaacgg atatttttaa ttttctttat tattattttc

1921 ttgttaactt ctgttggtat taattgtatt ggtttgtatt attggtatac agcacaagat

1981 ctccctctct agtca

//

LOCUS Hco-monepantel-1;6 1995 bp mRNA linear INV 05-MAR-2009

DEFINITION [gene=Hco-mptl-1].

SOURCE Haemonchus contortus

ORGANISM Haemonchus contortus

Eukaryota; Metazoa; Nematoda; Chromadorea; Rhabditida; Strongylida;

Trichostrongyloidea; Haemonchidae; Haemonchinae; Haemonchus.

REFERENCE 1 (bases 1 to 1995)

AUTHORS Rufener,L., Maser,P., Roditi,I. and Kaminsky,R.

TITLE Haemonchus contortus Acetylcholine Receptors of the DEG-3 Subfamily

and their Role in Sensitivity to Monepantel

JOURNAL PLoS Pathogens, in press

REFERENCE 2 (bases 1 to 1995)

AUTHORS Rufener,L., Maser,P., Roditi,I. and Kaminsky,R.

TITLE Direct Submission

JOURNAL Submitted (05-MAR-2009) Institute of Cell Biology, University of

Bern, Baltzerstrasse 4, Bern, BE 3012, Switzerland

FEATURES Location/Qualifiers

source 1..1995

/organism="Haemonchus contortus"

/mol_type="mRNA"

/isolate="Hc-Howick"

/clone="wt-6"

/note="monepantel-sensitive isolate; wildtype gene"

CDS 298..1992

/note="Hco-MPTL-1"

/codon_start=1

/product="H. contortus Monepantel-1"

/translation="MQNLILILLISTLFSRSEAISTEVPEHYLITNFILSRYNKGLIP

KRLQNESIKVSFSMELYQIIQVNEPQQFLMLNAWIVERWVDNLLGWDPEEFSNVTEIM

IPYDNLWIPDTTLYNSLVMDDHDTRRLLNAKLTTRGKDKGALVELLYPTIYKLSCLLD

LRFFPFDVQTCKLTFGSWTFDNTLIDYFPHNVTHAIGITNCIDNEGWTVLRTTVERHV

NHYDCCPNNYTLLEFHLNIQRKPLYYVINLITPTSIITLISIVGFFSSSSINDLREEK

ITLGITTLLSMSILIFMVSDKMPSTSSFIPLIGWFYTCMILLISFSTLAASMVIYVQK

QGILGKPPCRKTMRWARLVARCVRMEMPLLMKQAYAQKAREDKLRRAQEGRKQSLWHR

VYKLAKEQAQMRKQSSNTLPKINGIGNVPSPDVQQLQVPKKSCTISTDVTCINDPCDT

NALVEFSNMSDEDNSSFPDIEYTGATLATPTSKFQGLHKMSTCASLDSMIRNVDLTVT

SPRTMQRNLAELEFDWLAAVIERIFLIFFIIIFLLTSVGINCIGLYYWYTAQDRPL"

BASE COUNT 594 a 459 c 402 g 540 t

ORIGIN

1 caaatggaaa atcctagctc cggaacttga tatcgcctga aaatttgtgg ttagaagaca

61 cgccagttcg cccaaggcta cccggtgtaa acaaagcaaa acctattcga atcaaccaga

121 tccaaagccg tttttaggaa tcgcaacgac atatctcttc tctcagtggt tcccttcgac

181 aaccagacca ggaggcggtc cgagggcaac aatttccttc accccaacgg aacaacgcct

241 caccaacacc catctgtgtc agcagtgatc agacgacgaa aacaccaaac gaccatcatg

301 cagaacctga ttctgatcct actcatcagt accctgttca gccgctcaga ggcgatatct

361 acagaagtgc cagagcatta tctgataacg aacttcattt tgtctcgata caacaaaggt

421 ctcataccga aacgtcttca gaacgagtcg ataaaggtgt ctttctcaat ggaactttat

481 cagattattc aagtgaatga accacaacag tttctcatgc tgaacgcctg gatcgttgag

541 cgatgggttg acaatttgct tggatgggat ccagaagaat tctcgaacgt cactgaaatt

601 atgataccgt atgataatct atggataccg gatacgacgc tttataattc gttagtcatg

661 gatgaccacg acactcgtcg ccttctgaac gccaagttga cgactcgtgg aaaagacaaa

721 ggagcacttg tggaattgct ttatccgact atttacaaac tcagttgttt gctagacctg

781 aggttcttcc catttgatgt acagacgtgt aaactaactt tcggcagttg gacatttgac

841 aatacgctca tcgactattt tcctcataat gtaacccatg caatcggcat caccaattgt

901 attgacaatg agggatggac cgtattgaga acaacagtag aacgacatgt aaatcactac

961 gattgctgtc caaacaacta cacactcctg gaatttcatt taaatataca acgaaaaccc

1021 ctctattatg ttattaatct tattacgcct acttcgatca tcaccttgat ctcaattgtt

1081 gggttcttca gctcatcgtc gatcaacgat ctcagagaag agaaaattac acttggaatt

1141 acaacacttt tgtcaatgtc tatattaatc ttcatggtat ccgataaaat gccttctaca

1201 tcttccttca ttcctcttat tggatggttc tatacgtgca tgattttact gatatcgttt

1261 tcgacattag cagcttcaat ggttatttac gttcaaaaac aagggattct tggtaaaccg

1321 ccatgtcgga aaacaatgcg atgggctcgg ttggtggcac gatgtgttcg aatggaaatg

1381 ccactcctca tgaaacaagc ttatgcccaa aaagccaggg aagacaagct gagacgtgct

1441 caggagggac ggaaacagag tctatggcat cgagtatata aattggccaa agaacaggca

1501 caaatgagaa aacaatcaag caatacccta ccaaagatta atggaatcgg gaacgttcca

1561 tctccggatg ttcaacaact acaagtgccg aagaaaagtt gcacaatcag cacggacgtc

1621 acctgtatca atgacccgtg tgacacgaac gcattggtgg aattctcgaa tatgtcggat

1681 gaggataact cgtcctttcc agacattgag tacacaggtg ccactttggc cacgcccacg

1741 tccaaatttc aaggtctgca caaaatgagt acgtgcgcat cgctagacag tatgattcgc

1801 aacgttgact tgaccgtgac atcaccgcgg acaatgcaac ggaatcttgc cgagctggag

1861 tttgattggt tggcagctgt tattgaacgg atatttttaa ttttcttcat tattattttc

1921 ttgttaactt ctgttggtat taactgtatt ggtttgtatt attggtatac agcacaagat

1981 cgccctctct agtca

//

LOCUS Hco-monepantel-1;7 1999 bp mRNA linear INV 05-MAR-2009

DEFINITION [gene=Hco-mptl-1].

SOURCE Haemonchus contortus

ORGANISM Haemonchus contortus

Eukaryota; Metazoa; Nematoda; Chromadorea; Rhabditida; Strongylida;

Trichostrongyloidea; Haemonchidae; Haemonchinae; Haemonchus.

REFERENCE 1 (bases 1 to 1999)

AUTHORS Rufener,L., Maser,P., Roditi,I. and Kaminsky,R.

TITLE Haemonchus contortus Acetylcholine Receptors of the DEG-3 Subfamily

and their Role in Sensitivity to Monepantel

JOURNAL PLoS Pathogens, in press

REFERENCE 2 (bases 1 to 1999)

AUTHORS Rufener,L., Maser,P., Roditi,I. and Kaminsky,R.

TITLE Direct Submission

JOURNAL Submitted (05-MAR-2009) Institute of Cell Biology, University of

Bern, Baltzerstrasse 4, Bern, BE 3012, Switzerland

FEATURES Location/Qualifiers

source 1..1999

/organism="Haemonchus contortus"

/mol_type="mRNA"

/isolate="Hc-Howick"

/clone="wt-7"

/note="monepantel-sensitive isolate; wildtype gene"

CDS 302..1996

/note="Hco-MPTL-1"

/codon_start=1

/product="H. contortus Monepantel-1"

/translation="MQNLILILLISTLFSRSEAISTEVPEHYLITNFILSRYNKGLIP

KRLQNESIKVSFSMELYQIIQVNEPQQFLMLNAWIVERWVDNLLGWDPEEFSNVTEIM

IPYDNLWIPDTTLYNSLVMDDHDTRRLLNAKLTTRGKDEGALVELLYPTIYKLSCLLD

LRFFPFDVQTCKLTFGSWTFDNTLIDYFPHNVTHAIGITNCIDNEGWTVLRTTVERHV

NHYDCCPNNYTLLEFHLNIQRKPLYYVINLITPTSIITLISIVGFFSSSSINDLREEK

ITLGITTLLSMSILIFMVSDKMPSTSSFIPLIGWFYTCMILLISFSTLAASMVIYVQK

QGILGKPPCRKTMRWARLVARCVRMEMPLLMKQAYAQKAREDKLRRAQEGRKQSLWHR

VYKLAKEQAQMRKQSSNTLPKINGIGNVPSPDVQQLQVPKKSCTISTDVTCINDPCDT

NALVEFSNMSDEDNSSFPDIEYTGATLATPTSKFQGLHKMSTCASLDSMIRNVDLTVT

SPRTMQRNLAELEFDWLAAVIERIFLIFFIIIFLLTSVGINCIGLYYWYTAQDRPL"

BASE COUNT 597 a 466 c 402 g 534 t

ORIGIN

1 caaatggaaa atcctagctc tggaacttga tattgcctga aaatttgtgg ttagaagaca

61 cgccagttcg cccaggccta cccggtgtaa acaaagcaaa tcctattcga ttcaaccgga

121 tccaaaacca tttttaggca tcgcaacgac atatctcttc tctcagtggt tcccttcgac

181 aaccggacca ggaggcggtc cgagggcaac aatttccttc accccaacgg aacagcgcct

241 caccaaccaa cacccatcag tgtcagcagt gatcagacga ggaaaacacc aaacgaccat

301 catgcagaac ctgattctga tcctactcat cagtaccctg ttcagtcgtt cagaagcgat

361 ctctacagaa gtgccagagc attatttgat aacgaacttc attttgtccc gatacaacaa

421 aggtctcata ccgaaacgtc ttcaaaacga atcaataaag gtgtctttct ccatggaact

481 ctatcagatt attcaagtga atgaaccaca acagtttctc atgcttaacg cctggatcgt

541 tgagcgatgg gttgacaatt tgctcggatg ggatcctgaa gaattctcga acgtcactga

601 aattatgata ccgtacgata atctatggat accggatacg acgctttata attcgttggt

661 catggatgac cacgacactc gtcgcctttt gaacgcaaag ttaacgactc gtggaaaaga

721 cgaaggagca cttgtggaat tgctctatcc gactatttac aagctcagct gtttgctaga

781 cctgaggttc ttcccatttg atgtacagac gtgtaaacta actttcggca gttggacatt

841 tgacaatacg ctcatcgact attttcctca taatgtaaca catgcaatcg gcatcaccaa

901 ctgtattgac aatgagggat ggaccgtatt gagaacaaca gtagaacgac atgtaaatca

961 ctacgattgc tgtccgaaca actacactct tctggaattc catttaaata tacaacgaaa

1021 accgctctat tatgtcatta atcttattac acctacttcg atcatcaccc tgatctcaat

1081 tgttgggttt ttcagctcat cgtcgatcaa cgatctcaga gaggagaaaa ttacacttgg

1141 aattacaaca ctattgtcaa tgtctatatt aatctttatg gtatccgata aaatgccttc

1201 tacatcctcc ttcattcctc ttattggatg gttttatacg tgcatgattt tactgatatc

1261 gttttcgacg ttagcagctt caatggttat ttacgtacag aaacaaggga ttcttggtaa

1321 accaccctgt cggaaaacaa tgcgatgggc tcggttagtg gcacgatgtg tccgaatgga

1381 aatgccactt cttatgaaac aagcttatgc acagaaagca agggaagaca agctaagacg

1441 tgcccaagag ggtcggaaac agagcctatg gcaccgagta tacaaattgg ccaaagaaca

1501 ggcacaaatg agaaaacaat caagcaacac actaccaaag attaatggaa tcggaaacgt

1561 tccatcgcca gatgttcagc aactacaagt gccaaagaag agttgcacaa tcagcacgga

1621 cgtcacctgc atcaatgacc cgtgtgacac gaacgcattg gtggaattct cgaatatgtc

1681 ggatgaggac aactcgtcct ttcccgacat cgagtacaca ggagccacgt tggccacgcc

1741 cacgtccaag tttcaaggtc tgcacaaaat gagtacgtgc gcatcgctag acagtatgat

1801 tcgcaacgtt gacttgaccg taacatcacc gcggacaatg caacgaaatc ttgccgagct

1861 ggagtttgat tggttggcag ctgttattga acggatattt ttaattttct ttattattat

1921 tttcttgtta acttctgttg gtattaattg tattggtttg tattattggt atacagcaca

1981 agatcgccct ctctagtca

//

LOCUS Hco-monepantel-1;8 1156 bp mRNA linear INV 05-MAR-2009

DEFINITION [gene=Hco-mptl-1].

SOURCE Haemonchus contortus

ORGANISM Haemonchus contortus

Eukaryota; Metazoa; Nematoda; Chromadorea; Rhabditida; Strongylida;

Trichostrongyloidea; Haemonchidae; Haemonchinae; Haemonchus.

REFERENCE 1 (bases 1 to 1156)

AUTHORS Rufener,L., Maser,P., Roditi,I. and Kaminsky,R.

TITLE Haemonchus contortus Acetylcholine Receptors of the DEG-3 Subfamily

and their Role in Sensitivity to Monepantel

JOURNAL PLoS Pathogens, in press

REFERENCE 2 (bases 1 to 1156)

AUTHORS Rufener,L., Maser,P., Roditi,I. and Kaminsky,R.

TITLE Direct Submission

JOURNAL Submitted (05-MAR-2009) Institute of Cell Biology, University of

Bern, Baltzerstrasse 4, Bern, BE 3012, Switzerland

FEATURES Location/Qualifiers

source 1..1156

/organism="Haemonchus contortus"

/mol_type="mRNA"

/isolate="Hc-CRA"

/clone="wt-8"

/note="monepantel-sensitive isolate; wildtype gene"

CDS 342..>1154

/note="Hco-MPTL-1"

/codon_start=1

/product="H. contortus Monepantel-1"

/translation="MQNLILILLISTLFSRSEAISTEVPEHYLITNFILSRYNKGLIP

KRLQNESIKVSFSMELYQIIQVNEPQQFPMLNAWIVERWVDNLLGWDPEEFSNVTEIM

IPYDNLWIPDTTLYNSLVMDDHDTRRLLNAKLTTRGKDKGALVELLYPTIYKLSCLLD

LRFFPFDVQTCRLTFGSWTFDNTLIDYFPHNVTHAIGITNCIDNEGWTVLRTTVERHV

NHYDCCPNNYTLLEFHLNIQRKPLYYVINLITPTSIITLISIVGFFSSSSIND"

BASE COUNT 333 a 285 c 223 g 315 t

ORIGIN

1 ggtttaatta cccaagtttg agattgtacc gcgatcttca caaatggaaa atcctagctc

61 tggaacttga tattgcctga aaatttgtgg ttagaagaca cgccagttcg cccaggccta

121 cccggtgtaa acaaagcaaa tcctattcga ttcaacctga tccgaagtca tttttaggca

181 tcgcaacgac atatctcttc tctcagtggt tcccttcgac aaccagaatt ggaggcggtc

241 cgagggcaac aatttccttc accccaacgg aacagcgcct caccaaccaa cacccatcag

301 tgtcagcagt gatccgacga ggaaaacacc aaacgaccat catgcagaac ctgattctga

361 tcctactcat cagtaccctg ttcagtcgtt cagaagcgat ctctacagaa gtgccagagc

421 attatttgat aacgaacttc attttgtccc gatacaacaa aggtctcata ccgaaacgtc

481 ttcaaaacga atcaataaag gtgtctttct caatggaact ctaccagatt attcaagtga

541 atgaaccaca acagtttccc atgcttaacg cctggatcgt tgagcgatgg gttgacaatt

601 tgcttggatg ggatcctgaa gaattctcga acgtcactga aattatgata ccgtatgata

661 atctatggat accggatacg acgctttata attcgttagt catggatgac cacgacactc

721 gtcgccttct gaacgccaag ttgacgactc gtggaaaaga caaaggagca ctcgtggaac

781 tgctctatcc gactatttac aagctcagct gtttgttaga cctgaggttc ttcccatttg

841 atgtacagac gtgtagacta actttcggca gttggacatt tgacaatacg ctcatcgact

901 atttccctca taatgtgact catgctatcg gcattaccaa ttgtattgac aatgagggat

961 ggaccgtatt gagaacaaca gtggaacggc atgtaaatca ctacgattgc tgtccgaaca

1021 actacactct cctggaattt catttgaata tacagcgaaa accgctctat tatgttatta

1081 atcttattac acccacttcg atcatcaccc tgatctcaat tgttggtttc ttcagctcat

1141 cgtcgatcaa cgatct

//

LOCUS Hco-monepantel-1;9 1156 bp mRNA linear INV 05-MAR-2009

DEFINITION [gene=Hco-mptl-1].

SOURCE Haemonchus contortus

ORGANISM Haemonchus contortus

Eukaryota; Metazoa; Nematoda; Chromadorea; Rhabditida; Strongylida;

Trichostrongyloidea; Haemonchidae; Haemonchinae; Haemonchus.

REFERENCE 1 (bases 1 to 1156)

AUTHORS Rufener,L., Maser,P., Roditi,I. and Kaminsky,R.

TITLE Haemonchus contortus Acetylcholine Receptors of the DEG-3 Subfamily

and their Role in Sensitivity to Monepantel

JOURNAL PLoS Pathogens, in press

REFERENCE 2 (bases 1 to 1156)

AUTHORS Rufener,L., Maser,P., Roditi,I. and Kaminsky,R.

TITLE Direct Submission

JOURNAL Submitted (05-MAR-2009) Institute of Cell Biology, University of

Bern, Baltzerstrasse 4, Bern, BE 3012, Switzerland

FEATURES Location/Qualifiers

source 1..1156

/organism="Haemonchus contortus"

/mol_type="mRNA"

/isolate="Hc-CRA"

/clone="wt-9"

/note="monepantel-sensitive isolate; wildtype gene"

CDS 342..>1154

/note="Hco-MPTL-1"

/codon_start=1

/product="H. contortus Monepantel-1"

/translation="MQNLILILLISTLFSRSEAISTEVPEHYLITNFILSRYNKGLIP

KRLQNESIKVSFSMELYQIIQVNEPQQFLMLNAWIVERWVDNLLGWDPEEFSNVTEIM

IPYDNLWIPDTTLYNSLVMDDHDTRRLLNAKLTTRGKDKGALVELLYPTIYKLSCLLD

LRFFPFDVQTCKLTFGSWTFDNTLIDYFPHNVTHAIGITNCIDNEGWTVLRTTVERHV

NHYDCCPNNYTLLEFHLNIQRKPLYYVINLITPTSIITLISIVGFFSSSSIND"

BASE COUNT 343 a 285 c 218 g 310 t

ORIGIN

1 ggtttaatta cccaagtttg agattgtacc gcgatcttca caaatggaaa atcctagctc

61 tggaacttga tattgcctga aaatttgtgg ttagaagaca cgccagttcg cccaggccta

121 cccggtgtaa acaaagcaaa tcctattcga ttcaaccgga tccaaaacca tttttaggca

181 tcgcaacgac atatctcttc tctcagtggt tcccttcgac aaccggacca ggaggcggtc

241 cgagggcaac aatttccttc accccaacgg aacagcgcct caccaaccaa cacccatcag

301 tgtcagcagt gatcagacga ggaaaacacc aaacgaccat catgcagaac ctgattctga

361 tcctactcat cagtaccctg ttcagtcgtt cagaagcgat ctctacagaa gtgccagagc

421 attatttgat aacgaacttc attttgtccc gatacaacaa aggtctcata ccgaaacgtc

481 ttcaaaacga atcaataaag gtgtctttct ccatggaact ctatcagatt attcaagtga

541 atgaaccaca acagtttctc atgcttaacg cctggatcgt tgagcgatgg gttgacaatt

601 tgctcggatg ggatcctgaa gaattctcga acgtcactga aattatgata ccgtacgata

661 atctatggat accggatacg acgctttata attcgttggt catggatgac cacgacactc

721 gtcgcctttt gaacgcaaag ttaacgactc gtggaaaaga caaaggagca cttgtggaat

781 tgctctatcc gactatttac aagctcagct gtttgctaga cctgaggttc ttcccatttg

841 atgtacagac gtgtaaacta actttcggca gttggacatt tgacaatacg ctcatcgact

901 attttcctca taatgtaaca catgcaatcg gcatcaccaa ctgtattgac aatgagggat

961 ggaccgtatt gagaacaaca gtagaacgac atgtaaatca ctacgattgc tgtccgaaca

1021 actacactct tctggaattc catttaaata tacaacgaaa accgctctat tatgtcatta

1081 atcttattac acctacttcg atcatcaccc tgatctcaat tgttgggttt ttcagctcat

1141 cgtcgatcaa cgatct

//

LOCUS Hco-monepantel-1;m1-1 858 bp mRNA linear INV 05-MAR-2009

DEFINITION [gene=Hco-mptl-1].

SOURCE Haemonchus contortus

ORGANISM Haemonchus contortus

Eukaryota; Metazoa; Nematoda; Chromadorea; Rhabditida; Strongylida;

Trichostrongyloidea; Haemonchidae; Haemonchinae; Haemonchus.

REFERENCE 1 (bases 1 to 858)

AUTHORS Rufener,L., Maser,P., Roditi,I. and Kaminsky,R.

TITLE Haemonchus contortus Acetylcholine Receptors of the DEG-3 Subfamily

and their Role in Sensitivity to Monepantel

JOURNAL PLoS Pathogens, in press

REFERENCE 2 (bases 1 to 858)

AUTHORS Rufener,L., Maser,P., Roditi,I. and Kaminsky,R.

TITLE Direct Submission

JOURNAL Submitted (05-MAR-2009) Institute of Cell Biology, University of

Bern, Baltzerstrasse 4, Bern, BE 3012, Switzerland

FEATURES Location/Qualifiers

source 1..858

/organism="Haemonchus contortus"

/mol_type="mRNA"

/isolate="Hc-CRA AAD-mutant"

/clone="m1-1"

/note="monepantel-insensitive isolate; mutant gene"

CDS 44..70

/note="Hco-MPTL-1"

/codon_start=1

/product="H. contortus Monepantel-1"

/translation="MENPSSGT"

BASE COUNT 251 a 188 c 168 g 251 t

ORIGIN

1 ggtttaatta cccaagtttg agatagtact gcaatcttca caaatggaaa atcccagctc

61 tggtacttga ggttgatatc gcctgaaaat ttgtgaggcg atctctacag aagtgccaga

121 acattacctg ataacgaact tcattttgtc tcgatacaac aaaggtctca taccaaaacg

181 tcttcagaac gagtcgataa aggtgtcttt ctcgatggaa ctctatcaga ttattcaagt

241 gaatgaacca caacaatttc tcatgctaaa cgcctggatt gttgagcgat gggttgacaa

301 tttgcttgga tgggatccag aagaattctc gaacgtcact gaaattatga taccgtacga

361 taatctatgg ataccggata cgacgcttta taattcgtta gtcatggatg accacgacac

421 tcgtcgcctt ctgaacgcca agttgacggc tcgtggaaaa gacaaaggag cgcttgtgga

481 attgctctat ccgactattt acaagctcag ctgtttgtta gacctgaggt tcttcccact

541 tgatgtacaa acgtgtaaac taactttcgg cagttggaca tttgacaata cgctcatcga

601 ctatttccct cataatgtga ctcatgctat cggcattacc aattgtattg acaacgaggg

661 atggactgta ttgagaacga cagtagaacg acatgtaaat cactacgatt gctgtccgaa

721 caactacact ctcttggaat tccatttaaa tatacaacgg aagccgctct attatgttat

781 taatcttatt acacctactt caatcatcac cctgatctca attgttgggt tcttcagctc

841 atcgtcgatc aacgatct

//

LOCUS Hco-monepantel-1;m1-2 858 bp mRNA linear INV 05-MAR-2009

DEFINITION [gene=Hco-mptl-1].

SOURCE Haemonchus contortus

ORGANISM Haemonchus contortus

Eukaryota; Metazoa; Nematoda; Chromadorea; Rhabditida; Strongylida;

Trichostrongyloidea; Haemonchidae; Haemonchinae; Haemonchus.

REFERENCE 1 (bases 1 to 858)

AUTHORS Rufener,L., Maser,P., Roditi,I. and Kaminsky,R.

TITLE Haemonchus contortus Acetylcholine Receptors of the DEG-3 Subfamily

and their Role in Sensitivity to Monepantel

JOURNAL PLoS Pathogens, in press

REFERENCE 2 (bases 1 to 858)

AUTHORS Rufener,L., Maser,P., Roditi,I. and Kaminsky,R.

TITLE Direct Submission

JOURNAL Submitted (05-MAR-2009) Institute of Cell Biology, University of

Bern, Baltzerstrasse 4, Bern, BE 3012, Switzerland

FEATURES Location/Qualifiers

source 1..858

/organism="Haemonchus contortus"

/mol_type="mRNA"

/isolate="Hc-CRA AAD-mutant"

/clone="m1-2"

/note="monepantel-insensitive isolate; mutant gene"

CDS 44..70

/note="Hco-MPTL-1"

/codon_start=1

/product="H. contortus Monepantel-1"

/translation="MENPSSGT"

BASE COUNT 251 a 192 c 164 g 251 t

ORIGIN

1 ggtttaatta cccaagtttg agatagtact gcaatcttca caaatggaaa atcccagctc

61 tggtacttga ggttgatatc gcctgaaaat ttgtgaggcg atctctacag aagtgccaga

121 acattacctg ataacgaact tcattttgtc tcgatacaac aaaggtctca taccaaaacg

181 tcttcagaac gagtcgataa aggtgtcttt ctcgatggaa ctctatcaga ttattcaagt

241 gaatgaacca ctgcaatttc tcatgctaaa cgcctggatt gttgagcgat gggttgacaa

301 tttgcttgga tgggatccag aagaattctc gaacgtcact gaaattatga taccatacga

361 taatctgtgg ataccggata cgacacttta taattcgtta gtcatggatg accacgatac

421 tcgtcgcctt ctgaacgcca agttgacgac tcgtggaaaa gacaaaggag cacttgtgga

481 actgctctat ccgactattt acaaacttag ctgtttgtta gacctgcggt ccttcccatt

541 tgatgcacag acgtgtaaac tagctttcgg cagttggacc ttcgacaata cgctcatcga

601 ctattttcct cataatgtaa cccatgcaat cggcattact aactgtattg acaatgaagg

661 atggaccgta ttgagaacaa cagtagaacg acatgtaaat cactacgatt gctgtccgaa

721 caactacact cttctggagt ttcatttaaa tatccagcga aacccgctct attatgttat

781 taatcttatt acacctactt caatcatcac cctgatctca attgttgggt tcttcagctc

841 atcgtcgatc aacgatct

//

LOCUS Hco-monepantel-1;gDNA-1 956 bp DNA linear INV 05-MAR-2009

DEFINITION [gene=Hco-mptl-1].

SOURCE Haemonchus contortus

ORGANISM Haemonchus contortus

Eukaryota; Metazoa; Nematoda; Chromadorea; Rhabditida; Strongylida;

Trichostrongyloidea; Haemonchidae; Haemonchinae; Haemonchus.

REFERENCE 1 (bases 1 to 956)

AUTHORS Rufener,L., Maser,P., Roditi,I. and Kaminsky,R.

TITLE Haemonchus contortus Acetylcholine Receptors of the DEG-3 Subfamily

and their Role in Sensitivity to Monepantel

JOURNAL PLoS Pathogens, in press

REFERENCE 2 (bases 1 to 956)

AUTHORS Rufener,L., Maser,P., Roditi,I. and Kaminsky,R.

TITLE Direct Submission

JOURNAL Submitted (05-MAR-2009) Institute of Cell Biology, University of

Bern, Baltzerstrasse 4, Bern, BE 3012, Switzerland

FEATURES Location/Qualifiers

source 1..956

/organism="Haemonchus contortus"

/mol_type="genomic DNA"

/isolate="Hc-CRA"

/clone="gDNA-wt-1"

/note="monepantel-sensitive isolate; wildtype gene"

CDS join(<350..496,925..>954)

/note="Hco-MPTL-1"

/codon_start=1

/product="H. contortus Monepantel-1"

/translation="EAISTEVPEHYLITNFILSRYNKGLIPKRLQNESIKVSFSMELY

QIIQVNEPQQFLMLN"

BASE COUNT 295 a 203 c 193 g 265 t

ORIGIN

1 caattgggga ctcgaaccag caaccgtgcg tgttatatag tgcgcccttt accactacac

61 caccttcgtc ttttgatctg ccaaatacta ggaccaaata aaaatcaatt gtctatacat

121 atttcaagag agtatgacat aagaacaagt gcaaaagtga ctgactctac agttcaccat

181 attttcgatt ttagaacaag tcaggtagat tctactcgcg cgattatgta atgtactcac

241 tgtcttactt accactacct atagaaaccc actacaaaaa cttacttgag actattttca

301 aatattcatg tcgctcagaa tgaatgaatt taaatgcaga tattttatag aagcgatctc

361 tacagaagtg ccagagcatt atctaataac gaacttcatt ttgtcccgat acaacaaagg

421 tctcataccg aaacgtcttc agaacgaatc aataaaggtg tctttctcca tggaactcta

481 tcagattatt caagttgtga gtttgtgcaa gttttctgaa attgatggta ccaaaggccc

541 agaattgaaa gggagagcat ccagaatgta gagaaactcg gctgtaaggg tcccatgcga

601 tgctcccttg gacacagcaa atcgggcgat tcgatggtaa tcatcagatt acgatggtta

661 ttgatagagc cggattaaca gcagcatggc tgccatcctt acgatggcac cagcttatgc

721 gtgggaatct gtagttaagt aatcgtccag acttggagtt tgcttgttga agaactcttc

781 agctgaggtc cacctagaaa agcagtcaag ggtcttcagg gatggaacgg gaacgggtcg

841 aggagttcac tgatcatcgt tttcagccac ctctttgata gatgaattct tattccaact

901 caagtaagac gcatatcatt tcagaatgaa ccacaacagt ttctcatgct gaacgc

//

LOCUS Hco-monepantel-1;gDNA-2 963 bp DNA linear INV 05-MAR-2009

DEFINITION [gene=Hco-mptl-1].

SOURCE Haemonchus contortus

ORGANISM Haemonchus contortus

Eukaryota; Metazoa; Nematoda; Chromadorea; Rhabditida; Strongylida;

Trichostrongyloidea; Haemonchidae; Haemonchinae; Haemonchus.

REFERENCE 1 (bases 1 to 963)

AUTHORS Rufener,L., Maser,P., Roditi,I. and Kaminsky,R.

TITLE Haemonchus contortus Acetylcholine Receptors of the DEG-3 Subfamily

and their Role in Sensitivity to Monepantel

JOURNAL PLoS Pathogens, in press

REFERENCE 2 (bases 1 to 963)

AUTHORS Rufener,L., Maser,P., Roditi,I. and Kaminsky,R.

TITLE Direct Submission

JOURNAL Submitted (05-MAR-2009) Institute of Cell Biology, University of

Bern, Baltzerstrasse 4, Bern, BE 3012, Switzerland

FEATURES Location/Qualifiers

source 1..963

/organism="Haemonchus contortus"

/mol_type="genomic DNA"

/isolate="Hc-CRA"

/clone="gDNA-wt-2"

/note="monepantel-sensitive isolate; wildtype gene"

CDS join(<348..494,932..>961)

/note="Hco-MPTL-1"

/codon_start=1

/product="H. contortus Monepantel-1"

/translation="EAISTEVPEHYLITNFILSRYNKGLIPKRLQNESIKVSFSMELY

QIIQVNEPQQFLMLN"

BASE COUNT 307 a 200 c 196 g 259 t 1 others

ORIGIN

1 caattgggga ctcgaaccng caaccgtgcg tgtgatatag tgcgcccctt accactacac

61 cacctccgtt gatctgtgaa ataataggac gaaataaaaa aatcaattgt cattacatat

121 ttcaagaaag tatgacacaa gaacaagagc aaaagtgact gattttacaa gtctccatat

181 ttttgatttt agaacaagtc aggtagatac tactcgcgcg tttgtgaaat gtactcactg

241 tcttacttac cactacctat agaaacccat cacaaaaacc tacttgagac tattttcaaa

301 tattcatgac gctcagaatg aatgaattta aatgcagata ttttatagaa gcgatctcta

361 cagaagtgcc agagcattat ttgataacga acttcatttt gtcccgatac aacaaaggtc

421 tcataccgaa acgtcttcag aacgaatcaa taaaggtgtc tttctcaatg gaactctacc

481 agattattca agtggtgagt ttgtgaaaga tttctgaaat tgacgttacc aaaggcccag

541 aattgaaaga gagagcatcc agaatgtaga gaaactcggc tgtgagggtc ccatgcgatg

601 ctctcttgga cacagcaaat cgggcgattc gatgataatc atcagataac gatggttatt

661 gataggtgcg aatgaacaat agcatagttg ccatccttgc gatcgcatcc acgtatgcgt

721 gggaatctga agtatggtag tcgttcgaaa tggtggaact ttgaactgca cccttcagct

781 agtagttaag ctgaggtaca cctagggaat cctacaagac tccccaaggg aataagggaa

841 cgggttggag agttcacgat tcactgttct ctgctacctc tttgactgat gaatttttac

901 tccaactcaa gtacgacgcg tatcatttca gaatgaacca caacagtttc tcatgctgaa

961 cgc

//

LOCUS Hco-monepantel-1;gDNA-3 962 bp DNA linear INV 05-MAR-2009

DEFINITION [gene=Hco-mptl-1].

SOURCE Haemonchus contortus

ORGANISM Haemonchus contortus

Eukaryota; Metazoa; Nematoda; Chromadorea; Rhabditida; Strongylida;

Trichostrongyloidea; Haemonchidae; Haemonchinae; Haemonchus.

REFERENCE 1 (bases 1 to 962)

AUTHORS Rufener,L., Maser,P., Roditi,I. and Kaminsky,R.

TITLE Haemonchus contortus Acetylcholine Receptors of the DEG-3 Subfamily

and their Role in Sensitivity to Monepantel

JOURNAL PLoS Pathogens, in press

REFERENCE 2 (bases 1 to 962)

AUTHORS Rufener,L., Maser,P., Roditi,I. and Kaminsky,R.

TITLE Direct Submission

JOURNAL Submitted (05-MAR-2009) Institute of Cell Biology, University of

Bern, Baltzerstrasse 4, Bern, BE 3012, Switzerland

FEATURES Location/Qualifiers

source 1..962

/organism="Haemonchus contortus"

/mol_type="genomic DNA"

/isolate="Hc-CRA"

/clone="gDNA-wt-3"

/note="monepantel-sensitive isolate; wildtype gene"

CDS join(<348..494,932..>961)

/note="Hco-MPTL-1"

/codon_start=1

/product="H. contortus Monepantel-1"

/translation="EAISTEVPEHYLITNFILSRYNKGLIPKRLQNESIKVSFSMELY

QIIQVNEPQQFLMLN"

BASE COUNT 309 a 200 c 195 g 258 t

ORIGIN

1 caattgggga ctcgaaccag caaccgtgcg tgtgatatag tgcgcccctt accactacac

61 cacctccgtt gatctgtgaa ataataggac gaaataaaaa aatcaattgt cattacatat

121 ttcaagaaag tatgacacaa gaacaagagc aaaagtgact gattttacaa gtctccatat

181 ttttgatttt agaacaagtc aggtagatac tactcgcgcg tttgtgaaat gtactcacta

241 tcttacttac cactacctat agaaacccat cacaaaaacc tacttgagac tattttcaaa

301 tattcatgac gctcagaatg aatgaattta aatgcagata ttttatagaa gcgatctcta

361 cagaagtgcc agagcattat ttgataacga acttcatttt gtcccgatac aacaaaggtc

421 tcataccgaa acgtcttcag aacgaatcaa taaaggtgtc tttctcaatg gaactctacc

481 agattattca agtggtgagt ttgtgaaaga tttctgaaat tgacgttacc aaaggcccag

541 aattgaaaga gagagcatcc agaatgtaga gaaactcggc tgtgagggtc ccatgcgatg

601 ctctcttgga cacagcaaat cgggcgattc gatgataatc atcagataac gatggttatt

661 gataggtgcg aatgaacaat agcatagttg ccatccttgc gatcgcaccc acgtatgcgt

721 gggaatctga agtatggtag tcgttcgaaa tggtggaact ttgaactgca cccttcagct

781 agtagttaag ctgaggtaca cctagggaat cctacaagac tccccaaggg aataagggaa

841 cgggttggag agttcacgat tcactgttct ctgctacctc tttgactgat gaatttttac

901 tccaactcaa gtacgacgcg tatcatttca gaatgaacca caacagtttc tcatgctgaa

961 cg

//

LOCUS Hco-monepantel-1;gDNA-m2-1633 bp DNA linear INV 05-MAR-2009

DEFINITION [gene=Hco-mptl-1].

SOURCE Haemonchus contortus

ORGANISM Haemonchus contortus

Eukaryota; Metazoa; Nematoda; Chromadorea; Rhabditida; Strongylida;

Trichostrongyloidea; Haemonchidae; Haemonchinae; Haemonchus.

REFERENCE 1 (bases 1 to 633)

AUTHORS Rufener,L., Maser,P., Roditi,I. and Kaminsky,R.

TITLE Haemonchus contortus Acetylcholine Receptors of the DEG-3 Subfamily

and their Role in Sensitivity to Monepantel

JOURNAL PLoS Pathogens, in press

REFERENCE 2 (bases 1 to 633)

AUTHORS Rufener,L., Maser,P., Roditi,I. and Kaminsky,R.

TITLE Direct Submission

JOURNAL Submitted (05-MAR-2009) Institute of Cell Biology, University of

Bern, Baltzerstrasse 4, Bern, BE 3012, Switzerland

FEATURES Location/Qualifiers

source 1..633

/organism="Haemonchus contortus"

/mol_type="genomic DNA"

/isolate="Hc-CRA AAD-mutant"

/clone="gDNA-m2-1"

/note="monepantel-insensitive isolate; mutant gene"

CDS join(<153..173,602..>631)

/note="Hco-MPTL-1"

/codon_start=1

/product="H. contortus Monepantel-1"

/translation="LYQIIQVNEPQQFLMLN"

BASE COUNT 183 a 136 c 145 g 169 t

ORIGIN

1 caattgggga ctcgaaccag caaccgtgcg tgttatatag tgcgcccttt accactacac

61 caccttcgtc ttttgatctg ccaaatacta ggaccaaata aaaatcaatt gtctatacat

121 atttcaagag agtatgacat aagactctga cgctctatca gattattcaa gttgtgagtt

181 tgtgcaagtt ttctgaaatt gatggtacca aaggcccaga attgaaaggg agagcatcca

241 gaatgtagag aaactcggct gtaagggtcc catgcgatgc tcccttggac acagcaaatc

301 gggcgattcg atggtaatca tcagattacg atggttattg atagagccgg attaacagca

361 gcatggctgc catccttacg atggcaccag cttatgcgtg ggaatctgta gttaagtaat

421 cgtccagact tggagtttgc ttgttgaaga actcttcagc tgaggtccac ctagaaaagc

481 agtcaagggt cttcagggat ggaacgggaa cgggtcgagg agttcactga tcatcgtttt

541 cagccacctc tttgatagat gaattcttat tccaactcaa gtaagacgca tatcatttca

601 gaatgaacca caacagtttc tcatgctgaa cgc

//

LOCUS Hco-monepantel-1;gDNA-m2-2633 bp DNA linear INV 05-MAR-2009

DEFINITION [gene=Hco-mptl-1].

SOURCE Haemonchus contortus

ORGANISM Haemonchus contortus

Eukaryota; Metazoa; Nematoda; Chromadorea; Rhabditida; Strongylida;

Trichostrongyloidea; Haemonchidae; Haemonchinae; Haemonchus.

REFERENCE 1 (bases 1 to 633)

AUTHORS Rufener,L., Maser,P., Roditi,I. and Kaminsky,R.

TITLE Haemonchus contortus Acetylcholine Receptors of the DEG-3 Subfamily

and their Role in Sensitivity to Monepantel

JOURNAL PLoS Pathogens, in press

REFERENCE 2 (bases 1 to 633)

AUTHORS Rufener,L., Maser,P., Roditi,I. and Kaminsky,R.

TITLE Direct Submission

JOURNAL Submitted (05-MAR-2009) Institute of Cell Biology, University of

Bern, Baltzerstrasse 4, Bern, BE 3012, Switzerland

FEATURES Location/Qualifiers

source 1..633

/organism="Haemonchus contortus"

/mol_type="genomic DNA"

/isolate="Hc-CRA AAD-mutant"

/clone="gDNA-m2-2"

/note="monepantel-insensitive isolate; mutant gene"

CDS join(<153..173,602..>631)

/note="Hco-MPTL-1"

/codon_start=1

/product="H. contortus Monepantel-1"

/translation="LYQIIQVNEPQQFLMLN"

BASE COUNT 183 a 136 c 145 g 169 t

ORIGIN

1 caattgggga ctcgaaccag caaccgtgcg tgttatatag tgcgcccttt accactacac

61 caccttcgtc ttttgatctg ccaaatacta ggaccaaata aaaatcaatt gtctatacat

121 atttcaagag agtatgacat aagactctga cgctctatca gattattcaa gttgtgagtt

181 tgtgcaagtt ttctgaaatt gatggtacca aaggcccaga attgaaaggg agagcatcca

241 gaatgtagag aaactcggct gtaagggtcc catgcgatgc tcccttggac acagcaaatc

301 gggcgattcg atggtaatca tcagattacg atggttattg atagagccgg attaacagca

361 gcatggctgc catccttacg atggcaccag cttatgcgtg ggaatctgta gttaagtaat

421 cgtccagact tggagtttgc ttgttgaaga actcttcagc tgaggtccac ctagaaaagc

481 agtcaagggt cttcagggat ggaacgggaa cgggtcgagg agttcactga tcatcgtttt

541 cagccacctc tttgatagat gaattcttat tccaactcaa gtaagacgca tatcatttca

601 gaatgaacca caacagtttc tcatgctgaa cgc

//

LOCUS Hco-monepantel-1;gDNA-m2-3633 bp DNA linear INV 05-MAR-2009

DEFINITION [gene=Hco-mptl-1].

SOURCE Haemonchus contortus

ORGANISM Haemonchus contortus

Eukaryota; Metazoa; Nematoda; Chromadorea; Rhabditida; Strongylida;

Trichostrongyloidea; Haemonchidae; Haemonchinae; Haemonchus.

REFERENCE 1 (bases 1 to 633)

AUTHORS Rufener,L., Maser,P., Roditi,I. and Kaminsky,R.

TITLE Haemonchus contortus Acetylcholine Receptors of the DEG-3 Subfamily

and their Role in Sensitivity to Monepantel

JOURNAL PLoS Pathogens, in press

REFERENCE 2 (bases 1 to 633)

AUTHORS Rufener,L., Maser,P., Roditi,I. and Kaminsky,R.

TITLE Direct Submission

JOURNAL Submitted (05-MAR-2009) Institute of Cell Biology, University of

Bern, Baltzerstrasse 4, Bern, BE 3012, Switzerland

FEATURES Location/Qualifiers

source 1..633

/organism="Haemonchus contortus"

/mol_type="genomic DNA"

/isolate="Hc-CRA AAD-mutant"

/clone="gDNA-m2-3"

/note="monepantel-insensitive isolate; mutant gene"

CDS join(<153..173,602..>631)

/note="Hco-MPTL-1"

/codon_start=1

/product="H. contortus Monepantel-1"

/translation="LYQIIQVNEPQQFLMLN"

BASE COUNT 183 a 136 c 145 g 169 t

ORIGIN

1 caattgggga ctcgaaccag caaccgtgcg tgttatatag tgcgcccttt accactacac

61 caccttcgtc ttttgatctg ccaaatacta ggaccaaata aaaatcaatt gtctatacat

121 atttcaagag agtatgacat aagactctga cgctctatca gattattcaa gttgtgagtt

181 tgtgcaagtt ttctgaaatt gatggtacca aaggcccaga attgaaaggg agagcatcca

241 gaatgtagag aaactcggct gtaagggtcc catgcgatgc tcccttggac acagcaaatc

301 gggcgattcg atggtaatca tcagattacg atggttattg atagagccgg attaacagca

361 gcatggctgc catccttacg atggcaccag cttatgcgtg ggaatctgta gttaagtaat

421 cgtccagact tggagtttgc ttgttgaaga actcttcagc tgaggtccac ctagaaaagc

481 agtcaagggt cttcagggat ggaacgggaa cgggtcgagg agttcactga tcatcgtttt

541 cagccacctc tttgatagat gaattcttat tccaactcaa gtaagacgca tatcatttca

601 gaatgaacca caacagtttc tcatgctgaa cgc

//

LOCUS Hco-monepantel-1;m2-1 1877 bp mRNA linear INV 05-MAR-2009

DEFINITION [gene=Hco-mptl-1].

SOURCE Haemonchus contortus

ORGANISM Haemonchus contortus

Eukaryota; Metazoa; Nematoda; Chromadorea; Rhabditida; Strongylida;

Trichostrongyloidea; Haemonchidae; Haemonchinae; Haemonchus.

REFERENCE 1 (bases 1 to 1877)

AUTHORS Rufener,L., Maser,P., Roditi,I. and Kaminsky,R.

TITLE Haemonchus contortus Acetylcholine Receptors of the DEG-3 Subfamily

and their Role in Sensitivity to Monepantel

JOURNAL PLoS Pathogens, in press

REFERENCE 2 (bases 1 to 1877)

AUTHORS Rufener,L., Maser,P., Roditi,I. and Kaminsky,R.

TITLE Direct Submission

JOURNAL Submitted (05-MAR-2009) Institute of Cell Biology, University of

Bern, Baltzerstrasse 4, Bern, BE 3012, Switzerland

FEATURES Location/Qualifiers

source 1..1877

/organism="Haemonchus contortus"

/mol_type="mRNA"

/isolate="Hc-CRA AAD-mutant"

/clone="m2-1"

/note="monepantel-insensitive isolate; mutant gene"

CDS 326..382

/note="Hco-MPTL-1"

/codon_start=1

/product="H. contortus Monepantel-1"

/translation="MQNLILILLISTLFSRSE"

BASE COUNT 548 a 430 c 376 g 523 t

ORIGIN

1 gtttgagatt gtaccgcgat cttcacaaat ggaaaatcct agctctggaa cttgatattg

61 cctgaaaatt tgtggttaga ggactcacca gttcgcccaa gcctacccgg tgtaaacaaa

121 caaaacctat tcgattcaac ccgatccaaa gccattttta ggcatcgcaa cgacatatct

181 cttctctcag tggttccctt cgacaaccag gccaggaggc ggtccgaggg caacaatttc

241 cttcacctca acggaacaac gcctcaccaa ctaacaccca tctgtgtcag cagtaactag

301 acgacgaaaa catcaaacga ccatcatgca gaacctgatt ctgatcctac tcatcagtac

361 cctgttcagt cgttcagaat gaaccacaac agtttctcat gctgaacgcc tggatcgttg

421 agcgatgggt tgacaatttg cttggatggg atcctgaaga attttcgaac gtcaccgaaa

481 ttatggtacc gtacgataat ctatggatac cggatacgac gctttataat tcgttagtca

541 tggatgacca cgacactcgt cgccttctga acgccaagtt gacgactcgt ggaaaagaca

601 aaggagcgct tgtggaattg ctctatccga ctatttacaa gctcagctgt ttgttagacc

661 tgaggttctt cccatttgat gtacaaacgt gtaaactaac tttcggcagt tggacatttg

721 acaatacgct catcgactat ttccctcata atgtgactca tgctatcggc attaccaatt

781 gtattgacaa cgagggatgg actgtattga gaacgacagt agaacgacat gtaaatcact

841 acgattgctg tccgaacaac tacactctct tggaattcca tttaaatata caacggaaac

901 cgctctatta tgttattaat cttattacac ctacttcgat catcaccctg atctcaattg

961 ttgggttctt cagctcatca tcgatcaacg atctcagaga agagaaaatt acacttggaa

1021 ttacaactct tttgtcaatg tctattctaa ttttcatggt atccgataaa atgccttcta

1081 catcttcctt tatccctctt attggatggt tctatacatg catgatctta ctgatatcgt

1141 tttcgacatt agcagcttca atggttattt acgttcaaaa acaagggatt cttggtaaac

1201 cgccttgtcg aaaaacaatg cgatgggctc gattggttgc acgatgtgtc cgaatggaaa

1261 tgccacttct catgaaacaa gcttatgcac aaaaagccag ggaagataag ctaaggcgtg

1321 cacaggaggg tcggaaacag agtttatggc actgagtata caaattggcc aaagatcagg

1381 cacaaatgag aaaacaatca agcaataccc taccaaagat taatggaatc gggaacgttc

1441 catctccgga tgttcaacaa ctacaagtgc cgaagaagag ttgcacaatc agcacggacg

1501 tcacctgtat caatgacccg tgtgacacga acgcattggt ggaattctcg aatatgtcgg

1561 atgaggataa ctcgtccttt ccagacattg agtacacagg tgccactttg gccacgccca

1621 cgtcaaaatt tcaaggtctg cacaaaatga gtacgtgcgc atcgctagac agtatgattc

1681 gcaacgttga cttgaccgta acatcaccgc gaacaatgca acgaaatctt gccgagctgg

1741 agtttgattg gttggcagct gtaattgaac ggatattttt aattttcttt attattattt

1801 tcttgttaac ttctgttggt attaattgta ttggtttgta ttattggtat acagcacaag

1861 atcgccctct ctagtca

//

LOCUS Hco-monepantel-1;gDNA-4 787 bp DNA linear INV 05-MAR-2009

DEFINITION [gene=Hco-mptl-1].

SOURCE Haemonchus contortus

ORGANISM Haemonchus contortus

Eukaryota; Metazoa; Nematoda; Chromadorea; Rhabditida; Strongylida;

Trichostrongyloidea; Haemonchidae; Haemonchinae; Haemonchus.

REFERENCE 1 (bases 1 to 787)

AUTHORS Rufener,L., Maser,P., Roditi,I. and Kaminsky,R.

TITLE Haemonchus contortus Acetylcholine Receptors of the DEG-3 Subfamily

and their Role in Sensitivity to Monepantel

JOURNAL PLoS Pathogens, in press

REFERENCE 2 (bases 1 to 787)

AUTHORS Rufener,L., Maser,P., Roditi,I. and Kaminsky,R.

TITLE Direct Submission

JOURNAL Submitted (05-MAR-2009) Institute of Cell Biology, University of

Bern, Baltzerstrasse 4, Bern, BE 3012, Switzerland

FEATURES Location/Qualifiers

source 1..787

/organism="Haemonchus contortus"

/mol_type="genomic DNA"

/isolate="Hc-CRA"

/clone="gDNA-wt-4"

/note="monepantel-sensitive isolate; wildtype gene"

CDS join(<3..71,137..263,647..>786)

/note="Hco-MPTL-1"

/codon_start=1

/product="H. contortus Monepantel-1"

/translation="EGRKQSLWHRVCKLAKEQAQMRKQSSNTLPKINGIGNVPSPDVQ

QLQVPKKSCTISTDVTCINDQCDTNALVEFSNMSDEDNSSFPDIEYTGATLATPTSKF

QGLHKMSTCA"

BASE COUNT 250 a 164 c 173 g 200 t

ORIGIN

1 aagagggtcg gaagcagagt ctatggcatc gagtatgtaa attggccaaa gaacaggcac

61 aaatgagaaa agtaagtcaa ccagactata agatttttta tattgtactg tgatattatg

121 actatgcaac tttcagcaat caagcaatac cctaccaaag attaatggaa tcgggaacgt

181 tccatctccg gatgttcaac aactacaagt gccgaagaaa agttgcacaa taagcacgga

241 cgtcacctgt atcaatgacc agtgctagtt tttaaaattc atttaaaaat catttaaaag

301 tcgaaattgt tgaactgtga gcaagatgaa agtgaaagtg cgttgcccca ttgctctgag

361 actcccatgg tcgcatgggg aggaagggga gaggagggca aaagacgccg ttttcctgag

421 ccccctcgaa tgtgaacgtt cactcgtatc cgaaattccc taaaaataac tttcgcacta

481 ctccagtacc ctttcaatcg aatccgatac gctgtagact gtgtgagcat ggcgcactgt

541 agagaggtct agaagggtct agaagcaaat gatttacatc attcaattct atgtccctaa

601 taatatgaag atgtagatac aattctctaa ctcacgactg ttttaggtga cacgaacgca

661 ttggtggaat tctcgaatat gtcggatgag gataactcgt cctttccaga cattgagtac

721 acaggtgcca ctttggccac gcccacgtcc aaatttcaag gtctgcacaa aatgagtacg

781 tgcgcat

//

LOCUS Hco-monepantel-1;gDNA-5 801 bp DNA linear INV 05-MAR-2009

DEFINITION [gene=Hco-mptl-1].

SOURCE Haemonchus contortus

ORGANISM Haemonchus contortus

Eukaryota; Metazoa; Nematoda; Chromadorea; Rhabditida; Strongylida;

Trichostrongyloidea; Haemonchidae; Haemonchinae; Haemonchus.

REFERENCE 1 (bases 1 to 801)

AUTHORS Rufener,L., Maser,P., Roditi,I. and Kaminsky,R.

TITLE Haemonchus contortus Acetylcholine Receptors of the DEG-3 Subfamily

and their Role in Sensitivity to Monepantel

JOURNAL PLoS Pathogens, in press

REFERENCE 2 (bases 1 to 801)

AUTHORS Rufener,L., Maser,P., Roditi,I. and Kaminsky,R.

TITLE Direct Submission

JOURNAL Submitted (05-MAR-2009) Institute of Cell Biology, University of

Bern, Baltzerstrasse 4, Bern, BE 3012, Switzerland

FEATURES Location/Qualifiers

source 1..801

/organism="Haemonchus contortus"

/mol_type="genomic DNA"

/isolate="Hc-CRA"

/clone="gDNA-wt-5"

/note="monepantel-sensitive isolate; wildtype gene"

CDS join(<3..71,138..264,661..>800)

/note="Hco-MPTL-1"

/codon_start=1

/product="H. contortus Monepantel-1"

/translation="EGRKQSLWHRVYKLAKEQAQMRKQSSNTLPKINGIGNVPSPDVQ

QLQVPKKSCTISTDVTCINDPCDTNALVEFSNMSDEDNSSFPDIEYTGATLATPTSKF

QGLHKMSTCA"

BASE COUNT 244 a 159 c 187 g 211 t

ORIGIN

1 aagagggtcg gaagcagagt ctatggcacc gagtatataa attggccaaa gaacaggcac

61 aaatgagaaa agtaagtcaa ccagactaga agattttttg ataaaatacg gtaatattgt

121 gactgtgcaa ctttcagcaa tcaagcaata ccctaccaaa gattaatgga atcggtaacg

181 ttccatctcc ggatgttcaa caactgcaag tgccgaagaa gagttgcaca atcagcacgg

241 acgtcacctg tatcaatgac ccgtgctagt ttttaaaatt catttaaaaa tcatttaaaa

301 gtcgaggttg ttgaactgtg agcaagatgg aaacgagggt ataggtgaat cccgcgttgt

361 attcttagtt agttgcccta gttgcctcga tactcccatg gtgaatgggt agaagggaga

421 gaaggccaga agacgccgtt ttcctgagct ccctcgaatg tgaacgttcc aaggcttcca

481 aggaaatatc ttttccactc caccctttca atcgaattcg atacgcggta gactgtgtga

541 gcagggcgta ctgtggagag gtctagaagt ttctagaagc agtttgtttt gatcatttca

601 ctccatgtcg ataataatat aaagatggta gagaaaattt atcaactcat gttgttttag

661 gtgacacgaa cgcattggtg gaattctcga atatgtcgga tgaggataac tcgtcctttc

721 cagacattga gtacacaggt gccactttgg ccacgcccac gtccaaattt caaggtctgc

781 acaaaatgag tacgtgcgca t

//

LOCUS Hco-monepantel-1;gDNA-6 788 bp DNA linear INV 05-MAR-2009

DEFINITION [gene=Hco-mptl-1].

SOURCE Haemonchus contortus

ORGANISM Haemonchus contortus

Eukaryota; Metazoa; Nematoda; Chromadorea; Rhabditida; Strongylida;

Trichostrongyloidea; Haemonchidae; Haemonchinae; Haemonchus.

REFERENCE 1 (bases 1 to 788)

AUTHORS Rufener,L., Maser,P., Roditi,I. and Kaminsky,R.

TITLE Haemonchus contortus Acetylcholine Receptors of the DEG-3 Subfamily

and their Role in Sensitivity to Monepantel

JOURNAL PLoS Pathogens, in press

REFERENCE 2 (bases 1 to 788)

AUTHORS Rufener,L., Maser,P., Roditi,I. and Kaminsky,R.

TITLE Direct Submission

JOURNAL Submitted (05-MAR-2009) Institute of Cell Biology, University of

Bern, Baltzerstrasse 4, Bern, BE 3012, Switzerland

FEATURES Location/Qualifiers

source 1..788

/organism="Haemonchus contortus"

/mol_type="genomic DNA"

/isolate="Hc-CRA"

/clone="gDNA-wt-6"

/note="monepantel-sensitive isolate; wildtype gene"

CDS join(<3..71,138..264,648..>787)

/note="Hco-MPTL-1"

/codon_start=1

/product="H. contortus Monepantel-1"

/translation="EGRKQSLWHRVYKLAKEQAQMRKQSSNTLPKINGIGNVPSPDVQ

QLQVPKKSCTISTDVTCINDQCDTNALVEFSNMSDEDNSSFPDIEYTGATLATPTSKF

QGLHKMSTCA"

BASE COUNT 252 a 165 c 171 g 200 t

ORIGIN

1 aagagggtcg gaagcagagt ctatggcatc gagtatataa attggccaaa gaacaggcac

61 aaatgagaaa agtaagtcaa ccagactata agattttttt atattatact gtgatattat

121 gactatgcaa ctttcagcaa tcaagcaata ccctaccaaa gattaatgga atcgggaacg

181 ttccatctcc ggatgttcaa caactacaag tgccgaagaa aagttgcaca ataagcacgg

241 acgtcacctg tatcaatgac cagtgctagt ttttaaaatt catttaaaaa ccatttaaaa

301 gtcgaaattg ttgaactgtg agcaagatga aagtgaaagt gcgttgcccc attgctctga

361 gactcccatg gtcgcatggg gaggaagggg agaggagggc aaaagacgcc gttttcctga

421 gccccctcga atgtgaacgt tcactcgtat ccgaaattcc ctaaaaataa ctttcgcact

481 actccagtac cctttcaatc gaatccgata cgctgtagac tgtgtgagca tggcgcactg

541 tagagaggtc tagaagggtc tagaagcaaa tgatttacat cattcaattc tatgtcccta

601 ataatatgaa gatgtagata caattctcta actcacgact gttttaggtg acacgaacgc

661 attggtggaa ttctcgaata tgtcggatga ggataactcg tcctttccag acattgagta

721 cacaggtgcc actttggcca cgcccacgtc caaatttcaa ggtctgcaca aaatgagtac

781 gtgcgcat

//

LOCUS Hco-monepantel-1;gDNA-m3-1778 bp DNA linear INV 05-MAR-2009

DEFINITION [gene=Hco-mptl-1].

SOURCE Haemonchus contortus

ORGANISM Haemonchus contortus

Eukaryota; Metazoa; Nematoda; Chromadorea; Rhabditida; Strongylida;

Trichostrongyloidea; Haemonchidae; Haemonchinae; Haemonchus.

REFERENCE 1 (bases 1 to 778)

AUTHORS Rufener,L., Maser,P., Roditi,I. and Kaminsky,R.

TITLE Haemonchus contortus Acetylcholine Receptors of the DEG-3 Subfamily

and their Role in Sensitivity to Monepantel

JOURNAL PLoS Pathogens, in press

REFERENCE 2 (bases 1 to 778)

AUTHORS Rufener,L., Maser,P., Roditi,I. and Kaminsky,R.

TITLE Direct Submission

JOURNAL Submitted (05-MAR-2009) Institute of Cell Biology, University of

Bern, Baltzerstrasse 4, Bern, BE 3012, Switzerland

FEATURES Location/Qualifiers

source 1..778

/organism="Haemonchus contortus"

/mol_type="genomic DNA"

/isolate="Hc-CRA AAD-mutant"

/clone="gDNA-m3-1"

/note="monepantel-insensitive isolate; mutant gene"

CDS join(<3..71,128..254,638..>777)

/note="Hco-MPTL-1"

/codon_start=1

/product="H. contortus Monepantel-1"

/translation="EGRKQSLWHRVYKLAKDQAQMRKKSSNTLPKINGIGNVPSPDVQ

QLQVPKKSCTISTDVTCINDPCDTNALVEFSNMSDEDNSSFPDIEYTGATLATPTSKF

QGLHKMSTCA"

BASE COUNT 245 a 160 c 170 g 203 t

ORIGIN

1 aagagggtcg gaagcagagt ttatggcacc gagtatacaa attggccaaa gatcaggcac

61 aaatgagaaa agtaagtcaa ccagactata ggattttttt ataatatact gtaatattgt

121 gactgtgaaa tcaagcaata ccctaccaaa gattaatgga atcgggaacg ttccatctcc

181 ggatgttcaa caactacaag tgccgaagaa gagttgcaca atcagcacgg acgtcacctg

241 tatcaatgac ccgtgctagt ttttgaaatt catttaaaaa tcatttaaaa gtcgaaattg

301 ttgaactgtg agcaagatga aagtaaaagt tcgttgcccc attgctctga gactcccatg

361 gtcgcatggg gaggaacggg agaggaggtc aaaagacgcc gttttcctga gccccctcga

421 atgtgaatgt tcactcgtac ccaaatctcc ctaaaaataa cttttgcacc actttagtac

481 cctttcaatc gaatccgata tgctgtagac tgtgtgagca tggcgcactg tagagaggtc

541 tagaagggtc tagaagcaga tgatttacat cattcaattc tatgtcccta ataatatgaa

601 gatgtagata caattctcta actcacgatt gttttaggtg acacgaacgc attggtggaa

661 ttctcgaata tgtcggatga ggataactcg tcctttccag acattgagta cacaggtgcc

721 actttggcca cgcccacgtc aaaatttcaa ggtctgcaca aaatgagtac gtgcgcat

//

LOCUS Hco-monepantel-1;gDNA-m3-2778 bp DNA linear INV 05-MAR-2009

DEFINITION [gene=Hco-mptl-1].

SOURCE Haemonchus contortus

ORGANISM Haemonchus contortus

Eukaryota; Metazoa; Nematoda; Chromadorea; Rhabditida; Strongylida;

Trichostrongyloidea; Haemonchidae; Haemonchinae; Haemonchus.

REFERENCE 1 (bases 1 to 778)

AUTHORS Rufener,L., Maser,P., Roditi,I. and Kaminsky,R.

TITLE Haemonchus contortus Acetylcholine Receptors of the DEG-3 Subfamily

and their Role in Sensitivity to Monepantel

JOURNAL PLoS Pathogens, in press

REFERENCE 2 (bases 1 to 778)

AUTHORS Rufener,L., Maser,P., Roditi,I. and Kaminsky,R.

TITLE Direct Submission

JOURNAL Submitted (05-MAR-2009) Institute of Cell Biology, University of

Bern, Baltzerstrasse 4, Bern, BE 3012, Switzerland

FEATURES Location/Qualifiers

source 1..778

/organism="Haemonchus contortus"

/mol_type="genomic DNA"

/isolate="Hc-CRA AAD-mutant"

/clone="gDNA-m3-2"

/note="monepantel-insensitive isolate; mutant gene"

CDS join(<3..71,128..254,638..>777)

/note="Hco-MPTL-1"

/codon_start=1

/product="H. contortus Monepantel-1"

/translation="EGRKQSLWHRVYKLAKDQAQMRKKSSNTLPKINGIGNVPSPDVQ

QLQVPKKSCTISTDVTCINDPCDTNALVEFSNMSDEDNSSFPDIEYTGATLATPTSKF

QGLHKMSTCA"

BASE COUNT 245 a 160 c 170 g 203 t

ORIGIN

1 aagagggtcg gaagcagagt ttatggcacc gagtatacaa attggccaaa gatcaggcac

61 aaatgagaaa agtaagtcaa ccagactata ggattttttt ataatatact gtaatattgt

121 gactgtgaaa tcaagcaata ccctaccaaa gattaatgga atcgggaacg ttccatctcc

181 ggatgttcaa caactacaag tgccgaagaa gagttgcaca atcagcacgg acgtcacctg

241 tatcaatgac ccgtgctagt ttttgaaatt catttaaaaa tcatttaaaa gtcgaaattg

301 ttgaactgtg agcaagatga aagtaaaagt tcgttgcccc attgctctga gactcccatg

361 gtcgcatggg gaggaacggg agaggaggtc aaaagacgcc gttttcctga gccccctcga

421 atgtgaatgt tcactcgtac ccaaatctcc ctaaaaataa cttttgcacc actttagtac

481 cctttcaatc gaatccgata tgctgtagac tgtgtgagca tggcgcactg tagagaggtc

541 tagaagggtc tagaagcaga tgatttacat cattcaattc tatgtcccta ataatatgaa

601 gatgtagata caattctcta actcacgatt gttttaggtg acacgaacgc attggtggaa

661 ttctcgaata tgtcggatga ggataactcg tcctttccag acattgagta cacaggtgcc

721 actttggcca cgcccacgtc aaaatttcaa ggtctgcaca aaatgagtac gtgcgcat

//

LOCUS Hco-monepantel-1;gDNA-m3-3778 bp DNA linear INV 05-MAR-2009

DEFINITION [gene=Hco-mptl-1].

SOURCE Haemonchus contortus

ORGANISM Haemonchus contortus

Eukaryota; Metazoa; Nematoda; Chromadorea; Rhabditida; Strongylida;

Trichostrongyloidea; Haemonchidae; Haemonchinae; Haemonchus.

REFERENCE 1 (bases 1 to 778)

AUTHORS Rufener,L., Maser,P., Roditi,I. and Kaminsky,R.

TITLE Haemonchus contortus Acetylcholine Receptors of the DEG-3 Subfamily

and their Role in Sensitivity to Monepantel

JOURNAL PLoS Pathogens, in press

REFERENCE 2 (bases 1 to 778)

AUTHORS Rufener,L., Maser,P., Roditi,I. and Kaminsky,R.

TITLE Direct Submission

JOURNAL Submitted (05-MAR-2009) Institute of Cell Biology, University of

Bern, Baltzerstrasse 4, Bern, BE 3012, Switzerland

FEATURES Location/Qualifiers

source 1..778

/organism="Haemonchus contortus"

/mol_type="genomic DNA"

/isolate="Hc-CRA AAD-mutant"

/clone="gDNA-m3-3"

/note="monepantel-insensitive isolate; mutant gene"

CDS join(<3..71,128..254,638..>777)

/note="Hco-MPTL-1"

/codon_start=1

/product="H. contortus Monepantel-1"

/translation="EGRKQSLWHRVYKLAKDQAQMRKKSSNTLPKINGIGNVPSPDVQ

QLQVPKKSCTISTDVTCINDPCDTNALVEFSNMSDEDNSSFPDIEYTGATLATPTSKF

QGLHKMSTCA"

BASE COUNT 245 a 161 c 170 g 202 t

ORIGIN

1 aagagggtcg gaagcagagt ttatggcacc gagtatacaa attggccaaa gatcaggcac

61 aaatgagaaa agtaagtcaa ccagactata ggattttttt ataatatact gtaatattgt

121 gactgtgaaa tcaagcaata ccctaccaaa gattaatgga atcgggaacg ttccatctcc

181 ggatgttcaa caactacaag tgccgaagaa gagttgcaca atcagcacgg acgtcacctg

241 tatcaatgac ccgtgctagt ttttgaaatt catctaaaaa tcatttaaaa gtcgaaattg

301 ttgaactgtg agcaagatga aagtaaaagt tcgttgcccc attgctctga gactcccatg

361 gtcgcatggg gaggaacggg agaggaggtc aaaagacgcc gttttcctga gccccctcga

421 atgtgaatgt tcactcgtac ccaaatctcc ctaaaaataa cttttgcacc actttagtac

481 cctttcaatc gaatccgata tgctgtagac tgtgtgagca tggcgcactg tagagaggtc

541 tagaagggtc tagaagcaga tgatttacat cattcaattc tatgtcccta ataatatgaa

601 gatgtagata caattctcta actcacgatt gttttaggtg acacgaacgc attggtggaa

661 ttctcgaata tgtcggatga ggataactcg tcctttccag acattgagta cacaggtgcc

721 actttggcca cgcccacgtc aaaatttcaa ggtctgcaca aaatgagtac gtgcgcat

//

LOCUS Hco-monepantel-1;gDNA-7 788 bp DNA linear INV 05-MAR-2009

DEFINITION [gene=Hco-mptl-1].

SOURCE Haemonchus contortus

ORGANISM Haemonchus contortus

Eukaryota; Metazoa; Nematoda; Chromadorea; Rhabditida; Strongylida;

Trichostrongyloidea; Haemonchidae; Haemonchinae; Haemonchus.

REFERENCE 1 (bases 1 to 788)

AUTHORS Rufener,L., Maser,P., Roditi,I. and Kaminsky,R.

TITLE Haemonchus contortus Acetylcholine Receptors of the DEG-3 Subfamily

and their Role in Sensitivity to Monepantel

JOURNAL PLoS Pathogens, in press

REFERENCE 2 (bases 1 to 788)

AUTHORS Rufener,L., Maser,P., Roditi,I. and Kaminsky,R.

TITLE Direct Submission

JOURNAL Submitted (05-MAR-2009) Institute of Cell Biology, University of

Bern, Baltzerstrasse 4, Bern, BE 3012, Switzerland

FEATURES Location/Qualifiers

source 1..788

/organism="Haemonchus contortus"

/mol_type="genomic DNA"

/isolate="Hc-Howick"

/clone="gDNA-wt-7"

/note="monepantel-sensitive isolate; wildtype gene"

CDS join(<3..71,138..264,648..>787)

/note="Hco-MPTL-1"

/codon_start=1

/product="H. contortus Monepantel-1"

/translation="EGRKQSLWHRVYKLAKEQAQMRKQSSNTLPKINGIGNVPSPDVQ

QLQVPKKSCTISTDVTCINDPCDTNALVEFSNMSDEDNSSFPDIEYTGATLTTPTSKF

QGLHKMSTCA"

BASE COUNT 253 a 165 c 169 g 201 t

ORIGIN

1 aagagggtcg gaagcagagt ctatggcatc gagtatataa attggccaaa gaacaggcac

61 aaatgagaaa agtaagtcaa ccagactata agattttttt ataatatact gtaatattgt

121 gattatgcaa ctttcagcaa tcaagcaata ccctaccaaa gattaatgga atcgggaacg

181 ttccatctcc ggatgttcaa caactacaag tgccgaagaa aagttgcaca atcagcacgg

241 acgtcacctg tatcaatgac ccgtgctagt ttttaaaatt catttaaaaa tcatttaaaa

301 gtcgaagttg ttgaactgtg agcaagatga aagtgaaagt tcgttgcccc attgctctga

361 ggctcccatg gtcgcatggg gaggaacggg agaggaggtc aaaggacgcc gttttcctga

421 gccctctcga atgtgaacgt tcactcgtat ccaaaactcc ctaaaaataa cttttgcact

481 actttagtac cctttcaatc gaatccgata cgctgtagac tgtgtgagca tggcgcactg

541 tagagaggtc tagaagggtc tagaagcaaa tgatttacat catccaattc tatgtcccta

601 ataataagaa gatgtagata caattctcta actcacgatt gtttcaggtg acacgaacgc

661 actggtggaa ttctcgaata tgtcggatga ggataactcg tcttttccag acatcgagta

721 cacaggagcc actctaacca cgcccacgtc taaatttcaa gggctgcaca aaatgagtac

781 gtgcgcat

//

LOCUS Hco-monepantel-1;gDNA-8 787 bp DNA linear INV 05-MAR-2009

DEFINITION [gene=Hco-mptl-1].

SOURCE Haemonchus contortus

ORGANISM Haemonchus contortus

Eukaryota; Metazoa; Nematoda; Chromadorea; Rhabditida; Strongylida;

Trichostrongyloidea; Haemonchidae; Haemonchinae; Haemonchus.

REFERENCE 1 (bases 1 to 787)

AUTHORS Rufener,L., Maser,P., Roditi,I. and Kaminsky,R.

TITLE Haemonchus contortus Acetylcholine Receptors of the DEG-3 Subfamily

and their Role in Sensitivity to Monepantel

JOURNAL PLoS Pathogens, in press

REFERENCE 2 (bases 1 to 787)

AUTHORS Rufener,L., Maser,P., Roditi,I. and Kaminsky,R.

TITLE Direct Submission

JOURNAL Submitted (05-MAR-2009) Institute of Cell Biology, University of

Bern, Baltzerstrasse 4, Bern, BE 3012, Switzerland

FEATURES Location/Qualifiers

source 1..787

/organism="Haemonchus contortus"

/mol_type="genomic DNA"

/isolate="Hc-Howick"

/clone="gDNA-wt-8"

/note="monepantel-sensitive isolate; wildtype gene"

CDS join(<3..71,137..263,647..>786)

/note="Hco-MPTL-1"

/codon_start=1

/product="H. contortus Monepantel-1"

/translation="EGRKQSLWHRVYKLAKEQAQMRKQSSNTLPKINGIGNVPSPDVQ

QLQVPKKSCTISTDVTCINDPCDTNALVEFSNMSDEDNSSFPDIEYTGATLATPTSKF

QGLHKMSTCA"

BASE COUNT 245 a 165 c 174 g 203 t

ORIGIN

1 aagagggtcg gaagcagagt ctatggcatc gagtatataa attggccaaa gaacaggcac

61 aaatgagaaa agtaagtcaa ccagactata agatttttta tattatactg tgatattatg

121 actatgcaac tttcagcaat caagcaatac cctaccaaag attaatggaa tcgggaacgt

181 tccatctccg gatgttcaac aactacaagt gccgaagaaa agttgtacaa tcagcacgga

241 cgtcacctgt atcaatgacc cgtgctagtt tttaaaattc atttgaaaat catttaaaag

301 tcgaggttgt tgaactgtga gcaagatgaa agtgaaagtt cgttgcccca ttgctctgag

361 gctcccatgg tcgcatgggg aggaacggga gaggaggtca aaggacgccg ttttcctgag

421 ccccctcgaa tgtgaacgtt cactcgtatc caaagctccc taaaaataac ctttgcacta

481 ctttagtacc ctttcaatcg aatctaatac gctgtagact gtgtgagcat ggcgcactgt

541 agagaggtct agaggggtct agaagcagat gatttacatc atacaatcct atgtccctaa

601 taatatgaag atgtagatac aattctctaa ctcacgactg ttttaggtga cacgaacgca

661 ttggtggaat tctcgaatat gtcggatgag gataactcgt cctttccaga cattgagtac

721 acaggtgcca ctttggccac gcccacgtcc aaatttcaag gtctgcacaa aatgagtacg

781 tgcgcat

//

LOCUS Hco-monepantel-1;gDNA-9 787 bp DNA linear INV 05-MAR-2009

DEFINITION [gene=Hco-mptl-1].

SOURCE Haemonchus contortus

ORGANISM Haemonchus contortus

Eukaryota; Metazoa; Nematoda; Chromadorea; Rhabditida; Strongylida;

Trichostrongyloidea; Haemonchidae; Haemonchinae; Haemonchus.

REFERENCE 1 (bases 1 to 787)

AUTHORS Rufener,L., Maser,P., Roditi,I. and Kaminsky,R.

TITLE Haemonchus contortus Acetylcholine Receptors of the DEG-3 Subfamily

and their Role in Sensitivity to Monepantel

JOURNAL PLoS Pathogens, in press

REFERENCE 2 (bases 1 to 787)

AUTHORS Rufener,L., Maser,P., Roditi,I. and Kaminsky,R.

TITLE Direct Submission

JOURNAL Submitted (05-MAR-2009) Institute of Cell Biology, University of

Bern, Baltzerstrasse 4, Bern, BE 3012, Switzerland

FEATURES Location/Qualifiers

source 1..787

/organism="Haemonchus contortus"

/mol_type="genomic DNA"

/isolate="Hc-Howick"

/clone="gDNA-wt-9"

/note="monepantel-sensitive isolate; wildtype gene"

CDS join(<3..71,137..263,647..>786)

/note="Hco-MPTL-1"

/codon_start=1

/product="H. contortus Monepantel-1"

/translation="EGRKQSLWHRVYKLAKEQAQMRKQSSNTLPKINGIGNVPSPDVQ

QLQVPKKSCTISTDVTCINDPCDTNALVEFSNMSDEDNSSFPDIEYTGATLATPTSKF

QGLHKMSTCA"

BASE COUNT 248 a 167 c 173 g 199 t

ORIGIN

1 aagagggtcg gaagcagagt ctatggcatc gagtatataa attggccaaa gaacaggcac

61 aaatgagaaa agtaagtcaa ccagactata agatttttta tattatactg tgatattatg

121 actatgcaac tttcagcaat caagcaatac cctaccaaag attaatggaa tcgggaacgt

181 tccatctccg gatgttcaac aactacaagt gccgaagaaa agttgcacaa tcagcacgga

241 cgtcacctgt atcaatgacc cgtgctagtt tttaaaattc atttaaaaat catttgaaag

301 tcgaggttgt tgaactgtga gcaagatgaa agtgaaagtg cgttgcccca ttgctctgag

361 actcccatgg tcgcatgggg aggaagggga gaggagggca aaagacgccg ttttcctgag

421 ccccctcgaa tgtgaacgtt cactcgtatc cgaaattccc taaaaataac tttcgcacta

481 ctccaacacc ctttcaatcg aatccgatac gctgtagact gtgtgagcat ggcgcactgt

541 agagaggtct agaagggtct agaagcaaat gatttacatc attcaattct atgtccctaa

601 taatatgaag atgtagatac aattctctaa ctcacgactg ttttaggtga cacgaacgca

661 ttggtggaat tctcgaatat gtcggatgag gataactcgt cctttccaga cattgagtac

721 acaggtgcca ctttggccac gcccacgtcc aaatttcaag gtctgcacaa aatgagtacg

781 tgcgcat

//

LOCUS Hco-monepantel-1;gDNA-10 803 bp DNA linear INV 05-MAR-2009

DEFINITION [gene=Hco-mptl-1].

SOURCE Haemonchus contortus

ORGANISM Haemonchus contortus

Eukaryota; Metazoa; Nematoda; Chromadorea; Rhabditida; Strongylida;

Trichostrongyloidea; Haemonchidae; Haemonchinae; Haemonchus.

REFERENCE 1 (bases 1 to 803)

AUTHORS Rufener,L., Maser,P., Roditi,I. and Kaminsky,R.

TITLE Haemonchus contortus Acetylcholine Receptors of the DEG-3 Subfamily

and their Role in Sensitivity to Monepantel

JOURNAL PLoS Pathogens, in press

REFERENCE 2 (bases 1 to 803)

AUTHORS Rufener,L., Maser,P., Roditi,I. and Kaminsky,R.

TITLE Direct Submission

JOURNAL Submitted (05-MAR-2009) Institute of Cell Biology, University of

Bern, Baltzerstrasse 4, Bern, BE 3012, Switzerland

FEATURES Location/Qualifiers

source 1..803

/organism="Haemonchus contortus"

/mol_type="genomic DNA"

/isolate="Hc-Howick AAD-mutant"

/clone="gDNA-wt-10"

/note="monepantel-insensitive isolate; wildtype gene"

CDS join(<3..71,139..265,663..>802)

/note="Hco-MPTL-1"

/codon_start=1

/product="H. contortus Monepantel-1"

/translation="EGRKQSLWHRVYKLAKEQAQMRKQSSNTLPKINGIGNVPSPDVQ

QLQVPKKSCTINTDVTCINDPCDTNALVEFSNMSDEDNSSFPDIEYTGATLATPTSKF

QGLHKMSTCA"

BASE COUNT 249 a 162 c 188 g 204 t

ORIGIN

1 aagagggtcg gaagcagagt ctatggcacc gagtgtacaa attggccaaa gaacaggcac

61 aaatgagaaa agtaagttaa ccacactaga gcattttttt aataaaatac tgtaatattg

121 tgactgtgta actttcagca atcaagcaac accctaccaa agattaatgg gattgggaac

181 gttccatcgc cggatgttca gcaactacaa gtgccgaaaa agagttgtac aatcaacacg

241 gacgtcacct gtatcaatga cccgtgctag ttttaaattc atttgaaaat cattcaaaag

301 tctagattgc tgaactatgg gcaataagtg ggtgtatggg agaattctcg ggttgcaatt

361 cttagttagt tgcctcgatg ctctggggct cccatgggtg aatggggaag ggggagaaga

421 ggtcaaaaga cgctgtttct agctcgctcg attgtgaacg ttcactcgta tccaagaccc

481 caagaaaata tcttttgcgt tctacccttc caatcgaatt cgatacgcag tagactgtgt

541 gagcatggcg cactgtagag aggcctagaa agggctagaa gcagatgatt tacatcattc

601 aaatctatgt ccataataat ataaagacgt tagagacaat tctctaacga cgactgtttt

661 aggtgacacg aatgcattgg tggaattctc gaatatgtcg gatgaggata actcgtcctt

721 tcccgacatc gagtacacag gagccacttt ggccacgccc acgtcaaaat ttcaaggtct

781 gcacaaaatg agtacgtgcg cat

//

LOCUS Hco-monepantel-1;gDNA-11 803 bp DNA linear INV 05-MAR-2009

DEFINITION [gene=Hco-mptl-1].

SOURCE Haemonchus contortus

ORGANISM Haemonchus contortus

Eukaryota; Metazoa; Nematoda; Chromadorea; Rhabditida; Strongylida;

Trichostrongyloidea; Haemonchidae; Haemonchinae; Haemonchus.

REFERENCE 1 (bases 1 to 803)

AUTHORS Rufener,L., Maser,P., Roditi,I. and Kaminsky,R.

TITLE Haemonchus contortus Acetylcholine Receptors of the DEG-3 Subfamily

and their Role in Sensitivity to Monepantel

JOURNAL PLoS Pathogens, in press

REFERENCE 2 (bases 1 to 803)

AUTHORS Rufener,L., Maser,P., Roditi,I. and Kaminsky,R.

TITLE Direct Submission

JOURNAL Submitted (05-MAR-2009) Institute of Cell Biology, University of

Bern, Baltzerstrasse 4, Bern, BE 3012, Switzerland

FEATURES Location/Qualifiers

source 1..803

/organism="Haemonchus contortus"

/mol_type="genomic DNA"

/isolate="Hc-Howick AAD-mutant"

/clone="gDNA-wt-11"

/note="monepantel-insensitive isolate; wildtype gene"

CDS join(<3..71,139..265,663..>802)

/note="Hco-MPTL-1"

/codon_start=1

/product="H. contortus Monepantel-1"

/translation="EGRKQSLWHRVYKLAKEQAQMRKQSSNTLPKINGIGNVPSPDVQ

QLQVPKKSCTISTDVTCINDPCDTNALVEFSNMSDEDNSSFPDIEYTGATLATPTSKF

QGLHKMSTCA"

BASE COUNT 248 a 163 c 189 g 203 t

ORIGIN

1 aagagggtcg gaagcagagt ctatggcacc gagtgtacaa attggccaaa gaacaggcac

61 aaatgagaaa agtaagttaa ccacactaga gcattttttt aataaaatac tgtaatattg

121 tgactgtgta actttcagca atcaagcaac accctaccaa agattaatgg gattgggaac

181 gttccatcgc cggatgttca gcaactacaa gtgccgaaaa agagttgtac aatcagcacg

241 gacgtcacct gtatcaatga cccgtgctag ttttaaattc atttgaaaat cattcaaaag

301 tctagattgc tgaactatgg gcaataagtg ggtgtatggg agaattctcg ggttgcaatt

361 cttagttagt tgcctcgatg ctctggggct cccatgggtg aatggggaag ggggagaaga

421 ggtcaaaaga cgctgtttct agctcgctcg attgtgaacg ttcactcgta tccaagaccc

481 caagaaaata tcttttgcgt tctacccttc caatcgaatt cgatacgcag tagactgtgt

541 gagcatggcg cactgtagag aggcctagaa agggctagaa gcagatgatt tacatcattc

601 aaatctatgt ccataataat ataaagacgt tagagacaat tctctaacga cgactgtttt

661 aggtgacacg aatgcattgg tggaattctc gaatatgtcg gatgaggata actcgtcctt

721 tcccgacatc gagtacacag gagccacttt ggccacgccc acgtcaaaat ttcaaggtct

781 gcacaaaatg agcacgtgcg cat

//

LOCUS Hco-monepantel-1;gDNA-12 803 bp DNA linear INV 05-MAR-2009

DEFINITION [gene=Hco-mptl-1].

SOURCE Haemonchus contortus

ORGANISM Haemonchus contortus

Eukaryota; Metazoa; Nematoda; Chromadorea; Rhabditida; Strongylida;

Trichostrongyloidea; Haemonchidae; Haemonchinae; Haemonchus.

REFERENCE 1 (bases 1 to 803)

AUTHORS Rufener,L., Maser,P., Roditi,I. and Kaminsky,R.

TITLE Haemonchus contortus Acetylcholine Receptors of the DEG-3 Subfamily

and their Role in Sensitivity to Monepantel

JOURNAL PLoS Pathogens, in press

REFERENCE 2 (bases 1 to 803)

AUTHORS Rufener,L., Maser,P., Roditi,I. and Kaminsky,R.

TITLE Direct Submission

JOURNAL Submitted (05-MAR-2009) Institute of Cell Biology, University of

Bern, Baltzerstrasse 4, Bern, BE 3012, Switzerland

FEATURES Location/Qualifiers

source 1..803

/organism="Haemonchus contortus"

/mol_type="genomic DNA"

/isolate="Hc-Howick AAD-mutant"

/clone="gDNA-wt-12"

/note="monepantel-insensitive isolate; wildtype gene"

CDS join(<3..71,139..265,663..>802)

/note="Hco-MPTL-1"

/codon_start=1

/product="H. contortus Monepantel-1"

/translation="EGRKQSLWHRVYKLAKEQAQMRKQSSNTLPKINGIGNVPSPDVQ

QLQVPKKSCTIGTDVTCINDPCDTNALVEFSNMSDEDNSSFPDIEYTGATLATPTSKF

QGLHKMSTCA"

BASE COUNT 247 a 162 c 190 g 204 t

ORIGIN

1 aagagggtcg gaagcagagt ctatggcacc gagtgtacaa attggccaaa gaacaggcac

61 aaatgagaaa agtaagttaa ccacactaga gcattttttt aataaaatac tgtaatattg

121 tgactgtgta actttcagca atcaagcaac accctaccaa agattaatgg gattgggaac

181 gttccatcgc cggatgttca gcaactacaa gtgccgaaaa agagttgtac aatcggcacg

241 gacgtcacct gtatcaatga cccgtgctag ttttaaattc atttgaaaat cattcaaaag

301 tctagattgc tgaactatgg gcaataagtg ggtgtatggg agaattctcg ggttgcaatt

361 cttagttagt tgcctcgatg ctctggggct cccatgggtg aatggggaag ggggagaaga

421 ggtcaaaaga cgctgtttct agctcgctcg attgtgaacg ttcactcgta tccaagaccc

481 caagaaaata tcttttgcgt tctacccttc caatcgaatt cgatacgcag tagactgtgt

541 gagcatggcg cactgtagag aggcctagaa agggctagaa gcagatgatt tacatcattc

601 aaatctatgt ccataataat ataaagacgt tagagacaat tctctaacga cgactgtttt

661 aggtgacacg aatgcattgg tggaattctc gaatatgtcg gatgaggata actcgtcctt

721 tcccgacatc gagtacacag gagccacttt ggccacgccc acgtcaaaat ttcaaggtct

781 gcacaaaatg agtacgtgcg cat

//

LOCUS Hco-monepantel-1;m3-1 1896 bp mRNA linear INV 05-MAR-2009

DEFINITION [gene=Hco-mptl-1].

SOURCE Haemonchus contortus

ORGANISM Haemonchus contortus

Eukaryota; Metazoa; Nematoda; Chromadorea; Rhabditida; Strongylida;

Trichostrongyloidea; Haemonchidae; Haemonchinae; Haemonchus.

REFERENCE 1 (bases 1 to 1896)

AUTHORS Rufener,L., Maser,P., Roditi,I. and Kaminsky,R.

TITLE Haemonchus contortus Acetylcholine Receptors of the DEG-3 Subfamily

and their Role in Sensitivity to Monepantel

JOURNAL PLoS Pathogens, in press

REFERENCE 2 (bases 1 to 1896)

AUTHORS Rufener,L., Maser,P., Roditi,I. and Kaminsky,R.

TITLE Direct Submission

JOURNAL Submitted (05-MAR-2009) Institute of Cell Biology, University of

Bern, Baltzerstrasse 4, Bern, BE 3012, Switzerland

FEATURES Location/Qualifiers

source 1..1896

/organism="Haemonchus contortus"

/mol_type="mRNA"

/isolate="Hc-CRA AAD-mutant"

/clone="m3-1"

/note="monepantel-insensitive isolate; mutant gene"

CDS 326..1669

/note="Hco-MPTL-1"

/codon_start=1

/product="H. contortus Monepantel-1"

/translation="MQNLILILLISTLFSRSEAISTEVPEHYLITNFILSRYNKGLIP

KRLQNESIKVSFSMELYQIIQVNEPQQFLMLNAWIVERWVDNLLGWDPEEFSNVTETM

IPYDNLWIPDTTLYNSLVMDDHDTRRLLNAKLTTRGKDKGALVELLYPTIYKLSCLLD

LRFFPFDVQTCKLTFGSWTFDNTLIDYFPHNVTHAIGITNCIDNEGWTVLRTTVERHV

NHYDCCPNNYTLLEFHLNIQRKPLYYVINLITPTSIITLISIVGFFSSSSINDLREEK

ITLGITTLLSMSILIFMVSDKMPSTSSFIPLIGWFYTCMILLISFSTLAASMVIYVQK

QGILGKPPCRKTMRWARLVARCVRMEMPLLMKQAYAQKAREDKLRRAQEGRKQSLWHR

VYKLAKDQAQMRKVTRTHWWNSRICRMRITRPFQTLSTQVPLWPRPRQNFKVCTK"

BASE COUNT 555 a 432 c 373 g 536 t

ORIGIN

1 gtttgagatt gtaccgcgat cttcacaaat ggaaaatcct agctctggaa cttgatattg

61 cctgaaaatt tgtggttaga ggactcacca gttcgcccaa gcctacccgg tgtaaacaaa

121 caaaacctat tcgattcaac ccgatccaaa gccattttta ggcatcgcaa cgacatatct

181 cttctctcag tggttccctt cgacaaccag gccaggaggc ggtccgaggg caacaatttc

241 cttcacctca acggaacaac gcctcaccaa ctaacaccca tctgtgtcag cagtaactag

301 acgacgaaaa catcaaacga ccatcatgca gaacctgatt ctgatcctac tcatcagtac

361 cctgttcagt cgttcagaag cgatctctac agaagtgcca gagcattatc taataacgaa

421 cttcattttg tcccgataca acaaaggtct cataccgaaa cgtcttcaga acgaatcaat

481 aaaggtgtct ttctccatgg aactctatca gattattcaa gttaatgaac cacaacagtt

541 tctcatgctg aacgcctgga tcgttgagcg atgggttgac aatttgcttg gatgggatcc

601 tgaagaattt tcgaacgtca ccgaaactat gataccgtac gataatctat ggataccgga

661 tacgacgctt tataattcgt tagtcatgga tgaccacgac actcgtcgcc ttctgaacgc

721 caagttgacg actcgtggaa aagacaaagg agcgcttgtg gaattgctct atccgactat

781 ttacaagctc agctgtttgt tagacctgag gttcttccca tttgatgtac aaacgtgtaa

841 actaactttc ggcagttgga catttgacaa tacgctcatc gactatttcc ctcataatgt

901 gactcatgct atcggcatta ccaattgtat tgacaacgag ggatggactg tattgagaac

961 gacagtagaa cgacatgtaa atcactacga ttgctgtccg aacaactaca ctctcttgga

1021 attccattta aatatacaac ggaaaccgct ctattatgtt attaatctta ttacacctac

1081 ttcgatcatc accctgatct caattgttgg gttcttcagc tcatcatcga tcaacgatct

1141 cagagaagag aaaattacac ttggaattac aactcttttg tcaatgtcta ttctaatttt

1201 catggtatcc gataaaatgc cttctacatc ttcctttatc cctcttattg gatggttcta

1261 tacatgcatg atcttactga tatcgttttc gacattagca gcttcaatgg ttatttacgt

1321 tcaaaaacag gggattcttg gtaaaccgcc ttgtcgaaaa acaatgcgat gggctcgatt

1381 ggttgcacga tgtgtccgaa tggaaatgcc acttctcatg aaacaagctt atgcacaaaa

1441 agccagggaa gataagctaa gacgtgcaca ggagggtcgg aaacagagct tatggcaccg

1501 agtatacaaa ttggccaaag atcaggcaca aatgagaaaa gtgacacgaa cgcattggtg

1561 gaattctcga atatgtcgga tgaggataac tcgtcctttc cagacattga gtacacaggt

1621 gccactttgg ccacgcccac gtcaaaattt caaggtctgc acaaaatgag tacgtgcgca

1681 tcgctagaca gtatgattcg caacgttgac ttgaccgtaa catcaccgcg aacaatgcaa

1741 cgaaatcttg ccgagctgga gtttgattgg ttggcagctg taattgaacg gatattttta

1801 attttcttta ttattatttt cttgttaact tctgttggta ttaattgtat tggtttgtat

1861 tattggtata cagcacaaga tcgccctctc tagtca

//

LOCUS Hco-monepantel-1;m3-2 1655 bp mRNA linear INV 05-MAR-2009

DEFINITION [gene=Hco-mptl-1].

SOURCE Haemonchus contortus

ORGANISM Haemonchus contortus

Eukaryota; Metazoa; Nematoda; Chromadorea; Rhabditida; Strongylida;

Trichostrongyloidea; Haemonchidae; Haemonchinae; Haemonchus.

REFERENCE 1 (bases 1 to 1655)

AUTHORS Rufener,L., Maser,P., Roditi,I. and Kaminsky,R.

TITLE Haemonchus contortus Acetylcholine Receptors of the DEG-3 Subfamily

and their Role in Sensitivity to Monepantel

JOURNAL PLoS Pathogens, in press

REFERENCE 2 (bases 1 to 1655)

AUTHORS Rufener,L., Maser,P., Roditi,I. and Kaminsky,R.

TITLE Direct Submission

JOURNAL Submitted (05-MAR-2009) Institute of Cell Biology, University of

Bern, Baltzerstrasse 4, Bern, BE 3012, Switzerland

FEATURES Location/Qualifiers

source 1..1655

/organism="Haemonchus contortus"

/mol_type="mRNA"

/isolate="Hc-CRA AAD-mutant"

/clone="m3-2"

/note="monepantel-insensitive isolate; mutant gene"

CDS 301..>1653

/note="Hco-MPTL-1"

/codon_start=1

/product="H. contortus Monepantel-1"

/translation="MQNLILILLISTLFSRSEAISTEVPEHYLITNFILSRYNKGLIP

KRLQNESIKVSFSMELYQIIQVNEPQQFLMLNAWIVERWVDNLLGWDPEEFSNVTEIM

IPYDNLWIPDTTLYNSLVMDDHDTRRLLNAKLTTRGKDKGALVELLYPTIYKLSCLLD

LRFFPFDVQTCKLTFGSWTFDNTLIDYFPHNVTHAIGITNCIDNEGWTVLRTTVERHV

NHYDCCPNNYTLLEFHLNIQRKPLYYVINLTTPTSIITLISIVGFFSSSSINDLREEK

ITLGITTLLSMSILIFMVSDKMPSTSSFIPLIGWFYTCMILLISFSTLAASMVIYVQK

QGILGKPPCRKTMRWARLVARCVRMEMPLLMKQAYAQKAREDKLRRAQEGRKQSLWHR

VYKLAKDQAQMRKVTRTHWWNSRICRMRITRPFQTLSTQVPLWPRHVKISRSAQNEYV

R"

BASE COUNT 498 a 386 c 322 g 448 t 1 others

ORIGIN

1 caaatggaaa atcctagctc tggaacttga tattgcctga aaatttgtgg ttagaggact

61 caccagttcg cccaagccta cccggtgtaa acaaacaaaa cctattcgat tcaacccgan

121 ccaaagccat ttttaggcat cgcaacgaca tatctcttct ctcagtggtt cccttcgaca

181 accaggccag gaggcggtcc gagggcaaca atttccttca cctcaacgga acaacgcctc

241 accaactaac acccatctgt gtcagcagta actagacgac gaaaacatca aacgaccatc

301 atgcagaacc tgattctgat cctactcatc agtaccctgt ttagtcgttc agaagcgatc

361 tctacagaag tgccagagca ttatctaata acgaacttca ttttgtcccg atacaacaaa

421 ggtctcatac cgaaacgtct tcagaacgaa tcaataaagg tgtctttctc catggaactc

481 tatcagatta ttcaagttaa tgaaccacaa cagtttctca tgctaaacgc ctggatcgtt

541 gagcgatggg ttgacaattt gcttggatgg gatcctgaag aattttcgaa cgtcaccgaa

601 attatgatac cgtacgataa tctatggata ccggatacga cgctttataa ttcgttagtc

661 atggatgacc acgacactcg tcgccttctg aacgccaagt tgacgactcg tggaaaagac

721 aaaggagcgc ttgtggaatt gctctatccg actatttaca agctcagctg tttgttagac

781 ctgaggttct tcccatttga tgtacaaacg tgtaaactaa ctttcggcag ttggacattt

841 gacaatacgc tcatcgacta tttccctcat aatgtgactc atgctatcgg cattaccaat

901 tgtattgaca acgagggatg gactgtattg agaacgacag tagaacgaca tgtaaatcac

961 tacgattgct gtccgaacaa ctacactctc ttggaattcc atttaaatat acaacggaaa

1021 ccgctctatt atgttattaa tcttactaca cctacttcga tcatcaccct gatctcaatt

1081 gttgggttct tcagctcatc atcgatcaac gatctcagag aagagaaaat tacacttgga

1141 attacaactc ttttgtcaat gtctattcta attttcatgg tatccgataa aatgccttct

1201 acatcttcct ttatccctct tattggatgg ttctatacat gcatgatctt actgatatcg

1261 ttttcgacat tagcagcttc aatggttatt tacgttcaaa aacaagggat tcttggtaaa

1321 ccgccttgtc gaaaaacaat gcgatgggct cgattggttg cacgatgtgt ccgaatggaa

1381 atgccacttc tcatgaaaca agcttatgca caaaaagcca gggaagataa gctaagacgt

1441 gcacaggagg gtcggaaaca gagtttatgg caccgagtat acaaattggc caaagatcag

1501 gcacaaatga gaaaagtgac acgaacgcat tggtggaatt ctcgaatatg tcggatgagg

1561 ataactcgtc ctttccagac attgagtaca caggtgccac tttggccacg ccacgtcaaa

1621 atttcaaggt ctgcacaaaa tgagtacgtg cgcat

//

LOCUS Hco-monepantel-1;m3-3 1656 bp mRNA linear INV 05-MAR-2009

DEFINITION [gene=Hco-mptl-1].

SOURCE Haemonchus contortus

ORGANISM Haemonchus contortus

Eukaryota; Metazoa; Nematoda; Chromadorea; Rhabditida; Strongylida;

Trichostrongyloidea; Haemonchidae; Haemonchinae; Haemonchus.

REFERENCE 1 (bases 1 to 1656)

AUTHORS Rufener,L., Maser,P., Roditi,I. and Kaminsky,R.

TITLE Haemonchus contortus Acetylcholine Receptors of the DEG-3 Subfamily

and their Role in Sensitivity to Monepantel

JOURNAL PLoS Pathogens, in press

REFERENCE 2 (bases 1 to 1656)

AUTHORS Rufener,L., Maser,P., Roditi,I. and Kaminsky,R.

TITLE Direct Submission

JOURNAL Submitted (05-MAR-2009) Institute of Cell Biology, University of

Bern, Baltzerstrasse 4, Bern, BE 3012, Switzerland

FEATURES Location/Qualifiers

source 1..1656

/organism="Haemonchus contortus"

/mol_type="mRNA"

/isolate="Hc-CRA AAD-mutant"

/clone="m3-3"

/note="monepantel-insensitive isolate; mutant gene"

CDS 301..1644

/note="Hco-MPTL-1"

/codon_start=1

/product="H. contortus Monepantel-1"

/translation="MQNLILILLISTLFSRSEAISTEVPEHYLITNFILSRYNKGLIP

KRLQNESIKVSFSMELHQIIQVNEPQQFLMLNAWIVERWVDNLLGWDPEEFSNVTEIM

IPYDNLWIPDTTLYNSLVMDDHDTRRLLNAKLTTRGKDKGALVELLYPTIYKLSCLLD

LRFFPFDVQTCKLTFGSWTFDNTLIDYFPHNVTHAIGITNCIDNEGWTVLRTTVERHI

NHYDCCPNNYTLLEFHLNIQRKPLYYVINLITPTSIITLISIVGFFSSSSINDLREEK

ITLGITTLLSMSILIFMVSDKMPSTSSFIPLIGWLYTCMILLISFSTLAASMVIYVQK

QGILGKPPCRKTMRWARLVARCVRMEMPLLMKQAYAQKAREDKLRRAQEGRKQSLWHR

VYKLAKDQAQMRKVTRTHWWNSRICRMRITRPFQTLSTQVPLWPRPRQNFKVCTK"

BASE COUNT 498 a 389 c 322 g 446 t 1 others

ORIGIN

1 caaatggaaa atcctagctc tggaacttga tattgcctga aaatttgtgg ttagaggact

61 caccagttcg cccaagccta cccggtgtaa acaaacaaaa cctattcgat tcaacccgan

121 ccaaagccat ttttaggcat cgcaacgaca tatctcttct ctcagtggtt cccttcgaca

181 accaggccag gaggcggtcc gagggcaaca atttccttca cctcaacgga acaacgcctc

241 accaactaac acccatctgt gtcagcagta actagacgac gaaaacatca aacgaccatc

301 atgcagaacc tgattctgat cctactcatc agtaccctgt tcagtcgttc agaagcgatc

361 tctacagaag tgccagagca ttatctaata acgaacttca ttttgtcccg atacaacaaa

421 ggtctcatac cgaaacgtct tcagaacgaa tcaataaagg tgtctttctc catggaactc

481 catcagatta ttcaagttaa tgaaccacaa cagtttctca tgctgaacgc ctggatcgtt

541 gagcgatggg ttgacaattt gcttggatgg gatcctgaag aattttcgaa cgtcaccgaa

601 attatgatac cgtacgataa tctatggata ccggatacga cgctttataa ttcgttagtc

661 atggatgacc acgacactcg tcgccttctg aacgccaagt tgacgactcg tggaaaagac

721 aaaggagcgc ttgtggaatt gctctatccg actatttaca agctcagctg tttgttagac

781 ctgaggttct tcccatttga tgtacaaacg tgtaaactaa ctttcggcag ttggacattt

841 gacaatacgc tcatcgacta tttccctcat aatgtgactc atgctatcgg cattaccaat

901 tgtattgaca acgagggatg gactgtattg agaacgacag tagaacgaca tataaatcac

961 tacgattgct gtccgaacaa ctacactctc ttggaattcc atttaaatat acaacggaaa

1021 ccgctctatt atgttattaa tcttattaca cctacttcga tcatcaccct gatctcaatt

1081 gttgggttct tcagctcatc atcgatcaac gatctcagag aagagaaaat tacacttgga

1141 attacaactc ttttgtcaat gtctattcta attttcatgg tatccgataa aatgccttct

1201 acatcttcct ttatccctct tattggatgg ctctatacat gcatgatctt actgatatcg

1261 ttttcgacat tagcagcttc aatggttatt tacgttcaaa aacaagggat tcttggtaaa

1321 ccgccttgtc gaaaaacaat gcgatgggct cgattggttg cacgatgtgt ccgaatggaa

1381 atgccacttc tcatgaaaca agcttatgca caaaaagcca gggaagataa gctaagacgt

1441 gcacaggagg gtcggaaaca gagtttatgg caccgagtat acaaattggc caaagatcag

1501 gcacaaatga gaaaagtgac acgaacgcat tggtggaatt ctcgaatatg tcggatgagg

1561 ataactcgtc ctttccagac attgagtaca caggtgccac tttggccacg cccacgtcaa

1621 aatttcaagg tctgcacaaa atgagtacgt gcgcat

//

LOCUS Hco-monepantel-1;m4-1 1720 bp mRNA linear INV 05-MAR-2009

DEFINITION [gene=Hco-mptl-1].

SOURCE Haemonchus contortus

ORGANISM Haemonchus contortus

Eukaryota; Metazoa; Nematoda; Chromadorea; Rhabditida; Strongylida;

Trichostrongyloidea; Haemonchidae; Haemonchinae; Haemonchus.

REFERENCE 1 (bases 1 to 1720)

AUTHORS Rufener,L., Maser,P., Roditi,I. and Kaminsky,R.

TITLE Haemonchus contortus Acetylcholine Receptors of the DEG-3 Subfamily

and their Role in Sensitivity to Monepantel

JOURNAL PLoS Pathogens, in press

REFERENCE 2 (bases 1 to 1720)

AUTHORS Rufener,L., Maser,P., Roditi,I. and Kaminsky,R.

TITLE Direct Submission

JOURNAL Submitted (05-MAR-2009) Institute of Cell Biology, University of

Bern, Baltzerstrasse 4, Bern, BE 3012, Switzerland

FEATURES Location/Qualifiers

source 1..1720

/organism="Haemonchus contortus"

/mol_type="mRNA"

/isolate="Hc-CRA AAD-mutant"

/clone="m4-1"

/note="monepantel-insensitive isolate; mutant gene"

CDS 301..>1719

/note="Hco-MPTL-1"

/codon_start=1

/product="H. contortus Monepantel-1"

/translation="MQNLILILLISTLFSRSEAISTEVPEHYLITNFILSRYNKGLIP

KRLQNESIKNEPQQFLMLNAWIVERWVDNLLGWDPEEFSNVTEIMIPYDNLWIPDTTL

YNSLVMDDHDTRRLLNAKLTTRGKDKGALVELLYPTIYKLSCLLDLRFFPFDVQTCKL

TFGSWTFDNTLIDYFPHNVTHAIGITNCIDNEGWTVLRTTVERHVNHYDCCPNNYTLL

EFHLNIQRKPLYYVINLITPTSIITLISIVGFFSSSSINDLREEKITLGITTLLSMSI

LIFMVSDKMPSTSSFIPLIGWLYTCMILLISFSTLAASMVIYVQKQGILGKPPCRKTM

RWARLVARCVRMEMPLLMKQAYAQKAREDKLRRAQEGRKQSLWHRVYKLAKDQAQMRK

INGIGNVPSPDVQQLQVPKKSCTISTDVTCINDPCDTNALVEFSNMSDEDNSSFPDIE

YTGATLATPTSKFQGLHKMSTCA"

BASE COUNT 519 a 406 c 341 g 454 t

ORIGIN

1 caaatggaaa atcctagctc tggaacttga tattgcctga aaatttgtgg ttagaggact

61 caccagttcg cccaagccta cccggtgtaa acaaacaaaa cctattcgat tcaacccgat

121 ccaaagccat ttttaggcat cgcaacgaca tatctcttct ctcagtggtt cccttcgaca

181 accaggccag gaggcggtcc gagggcaaca atttccttca cctcaacgga acaacgcctc

241 accaactaac acccatctgt gtcagcagta actagacgac gaagacatca aacgaccatc

301 atgcagaacc tgattctgat cctactcatc agtaccctgt tcagtcgttc agaagcgatc

361 tctacagaag tgccagagca ttatctaata acgaacttca ttttgtcccg atacaacaaa

421 ggtctcatac cgaaacgtct tcagaacgaa tcaataaaga atgaaccaca acagtttctc

481 atgctgaacg cctggatcgt tgagcgatgg gttgacaatt tgcttggatg ggatcctgaa

541 gaattttcga acgtcaccga aattatgata ccgtacgata atctatggat accggatacg

601 acgctttata attcgttagt catggatgac cacgacactc gtcgccttct gaacgccaag

661 ttgacgactc gtggaaaaga caaaggagcg cttgtggaat tgctctatcc gactatttac

721 aagctcagct gtttgttaga cctgaggttc ttcccatttg atgtacaaac gtgtaaacta

781 actttcggca gttggacatt tgacaatacg ctcatcgact atttccctca taatgtgact

841 catgctatcg gcattaccaa ttgtattgac aacgagggat ggactgtatt gagaacgaca

901 gtagaacgac atgtaaatca ctacgattgc tgtccgaaca actacactct cttggaattc

961 catttaaata tacaacggaa accgctctat tatgttatta atcttattac acctacttcg

1021 atcatcaccc tgatctcaat tgttgggttc ttcagctcat catcgatcaa cgatctcaga

1081 gaagagaaaa ttacacttgg aattacaact cttttgtcaa tgtctattct aattttcatg

1141 gtatccgata aaatgccttc tacatcttcc tttatccctc ttattggatg gctctataca

1201 tgcatgatct tactgatatc gttttcgaca ttagcagctt caatggttat ttacgttcaa

1261 aaacaaggga ttcttggtaa accgccttgt cgaaaaacaa tgcgatgggc tcgattggtt

1321 gcacgatgtg tccgaatgga aatgccactt ctcatgaaac aagcttatgc acaaaaagcc

1381 agggaagata agctaagacg tgcacaggag ggtcggaaac agagtttatg gcaccgagta

1441 tacaaattgg ccaaagatca ggcacaaatg agaaaaatta atggaatcgg gaacgttcca

1501 tctccggatg ttcaacaact acaagtgccg aagaagagtt gcacaatcag cacggacgtc

1561 acctgtatca atgacccgtg tgacacgaac gcattggtgg aattctcgaa tatgtcggat

1621 gaggataact cgtcctttcc agacattgag tacacaggtg ccactttggc cacgcccacg

1681 tcaaaatttc aaggtctgca caaaatgagt acgtgcgcat

//

LOCUS Hco-monepantel-1;10 1829 bp mRNA linear INV 05-MAR-2009

DEFINITION [gene=Hco-mptl-1].

SOURCE Haemonchus contortus

ORGANISM Haemonchus contortus

Eukaryota; Metazoa; Nematoda; Chromadorea; Rhabditida; Strongylida;

Trichostrongyloidea; Haemonchidae; Haemonchinae; Haemonchus.

REFERENCE 1 (bases 1 to 1829)

AUTHORS Rufener,L., Maser,P., Roditi,I. and Kaminsky,R.

TITLE Haemonchus contortus Acetylcholine Receptors of the DEG-3 Subfamily

and their Role in Sensitivity to Monepantel

JOURNAL PLoS Pathogens, in press

REFERENCE 2 (bases 1 to 1829)

AUTHORS Rufener,L., Maser,P., Roditi,I. and Kaminsky,R.

TITLE Direct Submission

JOURNAL Submitted (05-MAR-2009) Institute of Cell Biology, University of

Bern, Baltzerstrasse 4, Bern, BE 3012, Switzerland

FEATURES Location/Qualifiers

source 1..1829

/organism="Haemonchus contortus"

/mol_type="mRNA"

/isolate="Hc-Howick"

/clone="wt-10"

/note="monepantel-sensitive isolate; wildtype gene"

CDS 347..>1828

/note="Hco-MPTL-1"

/codon_start=1

/product="H. contortus Monepantel-1"

/translation="MQNLILILLISTLFSRSEAISTEVPEHYLITNFILSRYNKDLIP

KRLQNESIKVSFSMELHQIIQVNEPQQFLMLNAWIVERWVDNLLGWDPEEFSNVTEIM

IPYDNLWIPDTTLYNSLVMDDHDTRRLLNAKLTTRGKDKGALVELLYPTIYKLSCLLD

LRFFPFDVQTCKLTFGSWTFDNTLIDYFPHNVTHAIGITNCIDNEGWTVLRTTVERHV

NHYDCCPNNYTLLESHLNIQRKPLYYVINLITPTSIITLISIVGFFSSSSINDLREEK

ITLGITTLLSMSILIFMVSDKMPSTSSFIPLIGWFYTCMILLISFSTLAASMVIYVQK

QGILGKPPCRKTMRWARLVARCVRMEMPLLMKQAYAQKAREDKLRRAQEGRKQSLWHR

VYKLAKEQAQMRKQSSNTLPKINGIGNVPSPDVQQLQVPKKSCTISTDVTCINDPCDT

NALVEFSNMSDEDNSSFPDIEYTGATLATPTFKFQGLHKMSTCA"

BASE COUNT 559 a 431 c 362 g 477 t

ORIGIN

1 ggtttaatta cccaagtttg agatagtact gcaatcttca caaatggaaa atcctagctc

61 tggaacttga ggttcgtatc gcctaaaaat ttgtggttag aagacacgcc agttcgtcca

121 ggcctacccg gtgtaaacaa acaaaatcta ttcgattcaa ccagatccaa agccattttt

181 aggcatcgca acgacatatc tcttctctca gtggttccct tcgacaacca gaccaggagg

241 cggtccgagg gcaacaattt ccttcacccc aacggaacag cgcctcacca accaacaccc

301 atctgtgtca gcagtgatca gacgacgaaa acaccaaacg accatcatgc agaacctgat

361 tctgatccta ctcatcagta ccctgttcag tcgttcagag gcgatatcta cagaagtgcc

421 agagcattac ttgataacga acttcatttt gtctcgatac aacaaagatc tcataccaaa

481 acgtcttcag aacgaatcaa taaaggtgtc tttctcgatg gaactccatc agattattca

541 agtgaatgaa ccacaacaat ttctcatgct gaacgcctgg attgttgagc gatgggttga

601 caatctgctt ggatgggatc cagaagaatt ttcgaacgtc actgaaatca tgataccgta

661 tgataatcta tggataccgg acacaacgct ttataattcg ttagtcatgg atgaccacga

721 cactcgtcgc cttctgaatg ccaagttgac gactcgtgga aaagacaaag gagcactcgt

781 ggaactgctc tatccgacta tttacaagct tagctgtttg ctagacctga ggttcttccc

841 atttgatgta cagacgtgta aactaacttt cggcagttgg acatttgaca atacgctcat

901 cgactatttt cctcataatg taacccatgc aatcggcatc accaattgta ttgacaatga

961 gggatggacc gtattgagaa caacagtgga acggcatgta aatcactacg attgctgtcc

1021 taacaactac actctcctgg aatctcattt gaatatacaa cgaaaaccgc tctattatgt

1081 tattaatctt attacaccta cttcgatcat caccctgatc tcaattgttg ggttcttcag

1141 ctcatcgtcg atcaacgatc tcagagagga gaaaattaca cttggaatta caacactatt

1201 gtcaatgtct atattaatct ttatggtatc cgataaaatg ccttctacat cctccttcat

1261 tcctcttatt ggatggttct atacgtgcat gattttactg atatcgtttt cgacgttagc

1321 agcttcaatg gttatttacg tacagaaaca agggattctt ggtaaaccac cctgtcggaa

1381 aacaatgcga tgggctcggt tagtggcacg atgtgtccga atggaaatgc cacttctcat

1441 gaaacaagct tatgcacaaa aagccaggga agataagcta agacgtgcac aggagggtcg

1501 gaaacagagt ttatggcacc gagtatacaa attggccaaa gaacaggcac aaatgagaaa

1561 acaatcaagc aataccctac caaagattaa tggaatcggg aacgttccat ctccggatgt

1621 tcaacaacta caagtgccga agaaaagttg cacaatcagc acggacgtca cctgtatcaa

1681 tgacccatgt gacacgaacg cattggtgga attctcgaat atgtcggatg aggataactc

1741 gtcctttcca gacattgagt acacaggtgc cactttggcc acgcccacgt tcaaatttca

1801 aggtctgcac aaaatgagta cgtgcgcat

//

LOCUS Hco-monepantel-1;m5-1 1830 bp mRNA linear INV 05-MAR-2009

DEFINITION [gene=Hco-mptl-1].

SOURCE Haemonchus contortus

ORGANISM Haemonchus contortus

Eukaryota; Metazoa; Nematoda; Chromadorea; Rhabditida; Strongylida;

Trichostrongyloidea; Haemonchidae; Haemonchinae; Haemonchus.

REFERENCE 1 (bases 1 to 1830)

AUTHORS Rufener,L., Maser,P., Roditi,I. and Kaminsky,R.

TITLE Haemonchus contortus Acetylcholine Receptors of the DEG-3 Subfamily

and their Role in Sensitivity to Monepantel

JOURNAL PLoS Pathogens, in press

REFERENCE 2 (bases 1 to 1830)

AUTHORS Rufener,L., Maser,P., Roditi,I. and Kaminsky,R.

TITLE Direct Submission

JOURNAL Submitted (05-MAR-2009) Institute of Cell Biology, University of

Bern, Baltzerstrasse 4, Bern, BE 3012, Switzerland

FEATURES Location/Qualifiers

source 1..1830

/organism="Haemonchus contortus"

/mol_type="mRNA"

/isolate="Hc-Howick AAD-mutant"

/clone="m5-1"

/note="monepantel-insensitive isolate; mutant gene"

CDS 348..626

/note="Hco-MPTL-1"

/codon_start=1

/product="H. contortus Monepantel-1"

/translation="MQNLILILLISTLFSRSEAISTEVPEHYLITNFILSRYNKGLIP

KRLQNESIKVSFSMELYQIIQVNEPQQFLMLNAWIVERWVDNLLGWDP"

BASE COUNT 545 a 432 c 368 g 485 t

ORIGIN

1 ggtttaatta cccaagtttg agatagtact gcaatcttca caaatggaaa atcccagctc

61 tgggacttga ggttggaatc gcctgaaaat ttgtggttag aagacacgcc agttcgctca

121 agtctacccg gtgtaaacaa agcaaaccta ttcgattcaa ccggatccaa agccattctt

181 aggcatcgtg aacgacatct ctcttctctc agtggttccc ttcgacaacc agaccaggag

241 gcggtccgag ggcaacaatt tccttcactc caacggaaca gctcctcacc aaccaactcc

301 catctgtgtc agcagtgatc agacgacgga aacaccaaac aaccatcatg cagaatctaa

361 ttctgatcct actcatcagt accctgttca gtcgttcaga agcgatctcg acagaagtgc

421 cagagcatta tttgataacg aacttcattt tgtcccgata caacaaaggt ctcataccga

481 aacgtcttca gaacgaatca ataaaggtgt ctttctccat ggaactctat cagattattc

541 aagtgaatga accacaacag tttctcatgc tgaacgcctg gatcgttgag cgatgggttg

601 acaatttgct tggatgggat ccttaagaat tttcgaacgt cactgaaata atgataccgt

661 acgataatct ctggatacca gatacgacac tttataattc gttagtcatg gatgaccacg

721 acactcgtcg ccttctgaac gccaagttga cgactcgtgg aaaagacaaa ggagcacttg

781 tggaattgct ttatccgact atttacaaac tcagttgttt gctagacctg aggttcttcc

841 catttgatgt acagacgtgt aaactaactt tcggcagttg gacatttgac aatacgctca

901 tcgactattt tcctcataat gtaacccatg caatcggcat caccaattgt attgacaatg

961 agggatggac cgtattgaga acaacagtgg aacggcatgt aaatcactac gattgctgtc

1021 cgaacaacta cactctcctg gaatttcatt tgaatataca acgaaaaccg ctctattatg

1081 ttattaatct tattacacct acttcgatca tcaccctgat ctcaattgtt gggttcttca

1141 gctcatcgtc gatcaacgat ctcagagaag agaaaattac acttggaatt acaactcttt

1201 tgtcaatgtc tattctaatt ttcatggtat ccgataaaat gccttctaca tcttccttta

1261 tccctcttat tggatggttc tatacatgca tgatcttact gatatcgttt tcgacattag

1321 cagcttcgat ggttatttac gttcaaaaac aagggattct cggtaaacca ccttgtcgaa

1381 aaacaatgcg atgggctcgg ttagtggcac gatgtgttcg aatggaaatg ccactcctca

1441 tgaaacaagc ttatgcccaa aaagccaggg aagataagct gagacgtgct caagagggtc

1501 ggaaacagag tctgtggcac cgagtgtaca aattggccaa agaacaggca caaatgcgaa

1561 aacaatcaag caatacccta ccgaagatta atggaattgg gaacgttcca tcgccggatg

1621 ttcaacaact acaagtgccg aagaagagtt gcacaatcag cacggacgtc acctgtatca

1681 atgacccgtg tgacacgaac gcattggtgg aattctcgaa tatgtcggat gaggataact

1741 cgtcctctcc ggacattgag tacacaggtg ctactttggc cacgcccacg tccaaatttc

1801 aaggtctgca caaaatgagt acgtgcgcat

//

LOCUS Hco-monepantel-1;m6-1 1981 bp mRNA linear INV 05-MAR-2009

DEFINITION [gene=Hco-mptl-1].

SOURCE Haemonchus contortus

ORGANISM Haemonchus contortus

Eukaryota; Metazoa; Nematoda; Chromadorea; Rhabditida; Strongylida;

Trichostrongyloidea; Haemonchidae; Haemonchinae; Haemonchus.

REFERENCE 1 (bases 1 to 1981)

AUTHORS Rufener,L., Maser,P., Roditi,I. and Kaminsky,R.

TITLE Haemonchus contortus Acetylcholine Receptors of the DEG-3 Subfamily

and their Role in Sensitivity to Monepantel

JOURNAL PLoS Pathogens, in press

REFERENCE 2 (bases 1 to 1981)

AUTHORS Rufener,L., Maser,P., Roditi,I. and Kaminsky,R.

TITLE Direct Submission

JOURNAL Submitted (05-MAR-2009) Institute of Cell Biology, University of

Bern, Baltzerstrasse 4, Bern, BE 3012, Switzerland

FEATURES Location/Qualifiers

source 1..1981

/organism="Haemonchus contortus"

/mol_type="mRNA"

/isolate="Hc-Howick AAD-mutant"

/clone="m6-1"

/note="monepantel-insensitive isolate; mutant gene"

CDS 306..803

/note="Hco-MPTL-1"

/codon_start=1

/product="H. contortus Monepantel-1"

/translation="MHNLILILLISTLFSRSEAISTEVPEHYLITNFILSRYNKGLIP

KRLQNESIKVSFSMELYQIIQVNEPQQFLMLNAWSVERWVDNLLGWDPEEFSNVTEIM

IPYDNLWIPDTTLYNSLVMDDHDTRRLLNAKLTTRGKDKGALVELLYPTIYKLSCLLD

LRRVN"

BASE COUNT 580 a 452 c 402 g 547 t

ORIGIN

1 caaatggaaa atcctagctc tggtacttga ggttgatatc gcctgaaaat ttgtggttag

61 aagacacgcc agttcgtcca ggtctacccg gtgtaaacaa agcaaatcct attcgattca

121 acctgatccg aagtcatttt taggcatcgc aacgacatat ctcttctctc agtggttccc

181 ttcgacaacc agaattggag gcggtccgag ggcaacaatt tccttcaccc aacggaacag

241 cgcctcacca accaacaccc atctgtgtca gcagtaatca gacggcgaaa caccaaacga

301 ccatcatgca taacctgatt ctgatcctac tcatcagtac cctgttcagt cgctcagagg

361 cgatctctac agaagtgcca gaacattacc tgataacgaa cttcattttg tctcgataca

421 acaaaggtct cataccaaaa cgtcttcaga acgagtcgat aaaggtgtcc ttctcgatgg

481 aactctatca gattattcaa gtgaatgaac cacaacaatt tctcatgcta aacgcctgga

541 gtgttgagcg atgggttgac aatttgcttg gatgggatcc agaagaattc tcgaacgtca

601 ctgaaattat gataccatac gataatctgt ggataccgga tacgacactt tataattcgt

661 tagtcatgga tgaccacgat actcgtcgcc ttctgaacgc caagttgacg actcgtggaa

721 aagacaaagg agcacttgtg gaactgctct atccgactat ttacaaactt agctgtttgt

781 tagacctgcg acgtgtaaac taactttcgg cagttggacc ttcgacaata cgctcatcga

841 ctattttcct cataatgtaa cccatgcaat cggcattact aactgtattg acaatgaagg

901 atggaccgta ttgagaacaa cagtagaacg acatgtaaat cactacgatt gctgtccgaa

961 caactacact cttctggagt ttcatttaaa tatccagcga aaaccgctct attatgttat

1021 taatcttatt acacctactt caatcatcac cccgatctca attgttgggt tcttcagctc

1081 atcgtcgatt aacgatctca gagaggagaa aattacactt ggaattacaa cacttttgtc

1141 aatgtctata ctaattttta tggtatccga taaaatgcct tccacatctt catttattcc

1201 tcttatcgga tggttctata cgtgcatgat tttactgata tcgttttcga cattagcagc

1261 ttcgatggtt atttacgttc aaaaacaagg cattcttggt aaaccacctt gtcgaaaaac

1321 aatgcgatgg gctcggttag tggcacgatg tgtccgaatg gaaatgccac ttctcatgaa

1381 acaagcttat gcacaaaaag ccagggaaga taagctgaga cgtgcccaag agggtcggaa

1441 gcagagtcta tggcaccgag tgtacaaatt ggccaaagaa cgggcacaaa tgagaaaaca

1501 atcaagcaac accctaccaa agattaatgg gattgggaac gttccatcgc tggatgttca

1561 gcaactacaa gtgccgaaaa agagttgtac aatcagcacg gtcgtcacct gtatcaatga

1621 cccgtgtgac acgaatgcat tggtggaatt ctcgaatatg tcggatgagg ataactcgtc

1681 ctttcccgac atcgagtaca caggagccac tttggccacg cccacgtcaa aatttcaagg

1741 tctgcacaaa atgagtacgt gcgcatcgtt ggacagtatg attcgcaatg ttgacttgac

1801 cgtcacatca ccgcggacaa tgcaacggaa tcttgccgag ctggaatttg actggttggc

1861 agctgttatt gaacggatat ttttgatttt ctttattatt atttttttgt taacttctgt

1921 tggtattaat tgtattggtt tgtattattg gtatacagca caagatcgcc ctctctagtc

1981 a

//

LOCUS Hco-des-2H;1 1605 bp mRNA linear INV 05-MAR-2009

DEFINITION [gene=Hco-des-2H].

SOURCE Haemonchus contortus

ORGANISM Haemonchus contortus

Eukaryota; Metazoa; Nematoda; Chromadorea; Rhabditida; Strongylida;

Trichostrongyloidea; Haemonchidae; Haemonchinae; Haemonchus.

REFERENCE 1 (bases 1 to 1605)

AUTHORS Rufener,L., Maser,P., Roditi,I. and Kaminsky,R.

TITLE Haemonchus contortus Acetylcholine Receptors of the DEG-3 Subfamily

and their Role in Sensitivity to Monepantel

JOURNAL PLoS Pathogens, in press

REFERENCE 2 (bases 1 to 1605)

AUTHORS Rufener,L., Maser,P., Roditi,I. and Kaminsky,R.

TITLE Direct Submission

JOURNAL Submitted (05-MAR-2009) Institute of Cell Biology, University of

Bern, Baltzerstrasse 4, Bern, BE 3012, Switzerland

FEATURES Location/Qualifiers

source 1..1605

/organism="Haemonchus contortus"

/mol_type="mRNA"

/isolate="Hc-CRA"

/clone="wt-3"

/note="monepantel-sensitive isolate; wildtype gene, long

transcript"

CDS 1..1605

/note="Hco-DES-2H"

/codon_start=1

/product="H. contortus DES-2 homologue"

/translation="MYALTLTIAAALSAAMVASTPTQIQLVHDLLDKYDKKAKPMWDN

TKPINVSFTVSLYQILELNEPQQFVLLNAWIIERWYDEFLYWSPMEYQNITELRLPYD

SIWLPDTTLYNSLVMKDDDTRRLLNAKLTTDLQRRASLIELLYPTIYKFSCLLDLRFF

PFDVQNCTMIFSSWTYDQTGIDYFPASDEISIANYLENEGWELLKTEVSRHEVKYSCC

PNAYTLLHLTLYLRRKPLFYLVNLIIPTSIITLIAIVGFFTTSSASGMREEKVSLGIT

TLLSMSILMLMVSDQMPTTSTFIPLIGWFILAMIMVISLGTVVSSIIIAVQKRGSLGE

RLSKRTLKIAKMIAYFTCTALPSHIEKEQMMEAFDAVTPTGESVRTLKSAMDASKKWM

SLRRPKNGVAVVSDKSTDALIQVANTGAEDGQMTGMTPVAPLVPPAAILDDDLSLRSD

FSALPPSARLLKTKSSTRCNVFKDLTTSIRHNRQLAVAEFEWLATVTERTCFVIFVLC

FLIITIGINMIGYVHWSKADQRWKNQ"

BASE COUNT 451 a 355 c 341 g 458 t

ORIGIN

1 atgtacgccc tcacactcac cattgctgct gctctatctg cagccatggt tgcaagtaca

61 ccgactcaga ttcagctcgt tcacgatctg ctcgataaat atgacaagaa agcaaaaccg

121 atgtgggaca atacgaaacc gatcaatgtc tcgttcaccg tatcacttta ccagattctt

181 gaactaaacg aaccacagca gttcgtactc cttaacgcct ggatcattga gagatggtat

241 gatgaatttt tgtactggtc ccctatggaa taccaaaata tcactgaatt acggctaccg

301 tatgattcaa tttggttacc ggatactacg ctttacaatt cgttggtaat gaaggatgac

361 gatactagac gccttctgaa tgccaagcta acgacagatc ttcaacggcg agcatctctt

421 attgaacttc tttaccctac aatctacaaa ttctcttgtt tgcttgattt acgatttttc

481 ccatttgatg tgcagaattg tacaatgatt ttttcgtcat ggacctatga tcagacgggg

541 atcgattact tcccagcatc agatgagata tcgattgcta attatttgga aaatgaagga

601 tgggaacttt tgaagactga agtgagccgt catgaagtaa aatacagctg ctgcccgaat

661 gcctacacat tactacattt aacactttat ttgaggagga aaccattatt ctatttggtg

721 aacctgatca ttcctacctc tatcatcaca cttattgcta ttgtgggatt cttcacgacc

781 tcttctgcca gtggaatgcg tgaagagaaa gtatcattag gtattactac gttgttatca

841 atgtcgatct tgatgttaat ggtatctgat caaatgccta ccacgtcaac gttcatcccc

901 ctgattggat ggtttatact tgcaatgatc atggtgatct ccctcggaac agttgtctcg

961 tccattatta ttgctgttca aaaacgaggc agtcttggag aaaggctttc aaaacggacg

1021 ttaaagatcg ccaaaatgat tgcgtatttc acttgtacag ctttgccatc tcatattgag

1081 aaggagcaaa tgatggaagc attcgatgct gtgacaccaa caggggaatc agtgaggacc

1141 ttaaagtcag ccatggatgc gtcgaaaaag tggatgagcc ttcgacggcc caaaaacggc

1201 gtcgccgtcg tatccgacaa aagcaccgat gccctgattc aggtggcgaa cactggtgca

1261 gaagatggcc aaatgacggg tatgacaccg gtggcacctc tggtaccacc tgcagctatc

1321 ctcgatgatg atctttcgtt acggagtgat ttctcagctt taccaccatc ggcacggctg

1381 ctcaaaacga aatccagtac cagatgtaat gtattcaagg atttaactac gtccattcga

1441 cataatcggc aattggctgt ggctgaattc gaatggctgg ctaccgtcac ggaacgaact

1501 tgctttgtaa tttttgtact gtgtttcctg attatcacca tagggatcaa tatgattggc

1561 tacgttcatt ggagcaaagc agatcaacga tggaaaaatc agtga

//

LOCUS Hco-des-2H;2 1605 bp mRNA linear INV 05-MAR-2009

DEFINITION [gene=Hco-des-2H].

SOURCE Haemonchus contortus

ORGANISM Haemonchus contortus

Eukaryota; Metazoa; Nematoda; Chromadorea; Rhabditida; Strongylida;

Trichostrongyloidea; Haemonchidae; Haemonchinae; Haemonchus.

REFERENCE 1 (bases 1 to 1605)

AUTHORS Rufener,L., Maser,P., Roditi,I. and Kaminsky,R.

TITLE Haemonchus contortus Acetylcholine Receptors of the DEG-3 Subfamily

and their Role in Sensitivity to Monepantel

JOURNAL PLoS Pathogens, in press

REFERENCE 2 (bases 1 to 1605)

AUTHORS Rufener,L., Maser,P., Roditi,I. and Kaminsky,R.

TITLE Direct Submission

JOURNAL Submitted (05-MAR-2009) Institute of Cell Biology, University of

Bern, Baltzerstrasse 4, Bern, BE 3012, Switzerland

FEATURES Location/Qualifiers

source 1..1605

/organism="Haemonchus contortus"

/mol_type="mRNA"

/isolate="Hc-CRA"

/clone="wt-4"

/note="monepantel-sensitive isolate; wildtype gene, long

transcript"

CDS 1..1605

/note="Hco-DES-2H"

/codon_start=1

/product="H. contortus DES-2 homologue"

/translation="MYALTLTIAAALSAAMVASTPTQIQLVHDLLDKYDKKAKPMWDN

TKPINVSFTVSLYQILELNEPQQFVPLNAWIIERWYDEFLYWSPMEYQNITELRLPYD

SIWLPDTTLYNSLVMKDDDTRRLLNAKLTTDLQRRASLIELLYPTIYKFSCLLDLRFF

PFDVQNCTMIFSSWTYDQTGIDYFPASDEISIANYLENEGWELLKTEVSRHEVKYSCC

PNAYTLLHLTLYLRRKPLFYLVNLIIPTSIITLIAIVGFFTTSSASGMREEKVSLGIT

TLLSMSILMLMVSDQMPTTSTFIPLIGWFILAMIMVISLGTVVSSIIIAVQKRGSLGE

RLSKRTLKIAKMIAYFTCTALPSHIEKEQMVEAFDAVTPTGESVRTLKSAMDASKKWM

SLRRPKNGVAVVSDKSTDALIQMANSGAEDGQMTGMTPVAPLVPPAAIIDDDLSLRSD

FSALPPSARLLKTKSSTRCNVFKDLTTSIRHNRQLAVAEFEWLATVTERTCFVIFVLC

FLIITIGINMIGYVHWSKADQRWKNQ"

BASE COUNT 458 a 356 c 336 g 455 t

ORIGIN

1 atgtacgccc tcacactcac cattgctgct gctctatctg cagccatggt tgcaagtaca

61 ccgactcaga ttcagctcgt tcacgatctg ctcgataaat atgataaaaa ggcgaaaccg

121 atgtgggaca atacgaaacc gatcaatgtc tcgttcaccg tatcacttta tcagattctt

181 gaactaaacg aaccgcagca gttcgtacct ctcaacgcct ggatcattga gagatggtat

241 gatgaatttt tgtactggtc ccctatggaa taccaaaata tcactgaatt acggctaccg

301 tatgattcaa tttggttacc ggatactacg ctttacaatt cgttggtaat gaaggatgac

361 gatactagac gccttctgaa tgccaagcta acgacagatc ttcaacggcg agcatctctt

421 attgaacttc tttaccctac aatctacaaa ttctcttgtt tgcttgattt acgatttttc

481 ccatttgatg tgcagaattg tacaatgatt ttttcgtcat ggacctatga tcagacgggg

541 atcgattact tcccagcatc agatgagata tcgattgcta attatttgga aaatgaagga

601 tgggaacttt tgaagactga agtaagccgt catgaagtaa aatacagctg ctgcccgaac

661 gcctacacat tactacattt aacactttac ctgagaagaa aaccattatt ctatttggtg

721 aacctgatca ttcctacctc tatcatcaca cttattgcta ttgtgggatt cttcacgact

781 tcttctgcca gtggaatgcg tgaagaaaaa gtatcattag ggattactac gttgttatca

841 atgtcaatct taatgttaat ggtatctgat caaatgccta caacgtcaac gttcatccct

901 ctgattggat ggtttatact tgcaatgatc atggttattt ccctcggaac agtggtctcg

961 tccatcatta ttgcagttca aaagcgaggc agtctcggag aaaggctctc aaaacggacg

1021 ttaaagatcg ccaaaatgat tgcatatttc acttgcactg ctctgccttc tcatattgaa

1081 aaggagcaaa tggtggaagc attcgatgct gtgacaccga caggggaatc agtgaggacg

1141 ttaaagtcag ccatggatgc atcaaaaaag tggatgagcc ttcggcggcc caaaaatggt

1201 gttgccgtcg tatccgacaa aagcaccgat gcccttattc agatggcaaa ctctggtgca

1261 gaagatggcc aaatgacggg tatgacaccg gtggcaccac tggtaccacc tgcagctatc

1321 atcgacgatg atctttcgtt acgaagtgat ttctcagctt taccaccatc agcacggctg

1381 ctcaaaacga aatccagtac cagatgtaat gtattcaagg atttaactac gtccattcgg

1441 cataatcggc agttggctgt ggctgaattc gaatggctgg ctaccgtcac ggaacgaact

1501 tgtttcgtaa tttttgtgct gtgcttcctg atcatcacca taggaatcaa tatgattggc

1561 tacgttcatt ggagcaaagc agatcaacga tggaaaaatc agtga

//

LOCUS Hco-des-2H;3 1605 bp mRNA linear INV 05-MAR-2009

DEFINITION [gene=Hco-des-2H].

SOURCE Haemonchus contortus

ORGANISM Haemonchus contortus

Eukaryota; Metazoa; Nematoda; Chromadorea; Rhabditida; Strongylida;

Trichostrongyloidea; Haemonchidae; Haemonchinae; Haemonchus.

REFERENCE 1 (bases 1 to 1605)

AUTHORS Rufener,L., Maser,P., Roditi,I. and Kaminsky,R.

TITLE Haemonchus contortus Acetylcholine Receptors of the DEG-3 Subfamily

and their Role in Sensitivity to Monepantel

JOURNAL PLoS Pathogens, in press

REFERENCE 2 (bases 1 to 1605)

AUTHORS Rufener,L., Maser,P., Roditi,I. and Kaminsky,R.

TITLE Direct Submission

JOURNAL Submitted (05-MAR-2009) Institute of Cell Biology, University of

Bern, Baltzerstrasse 4, Bern, BE 3012, Switzerland

FEATURES Location/Qualifiers

source 1..1605

/organism="Haemonchus contortus"

/mol_type="mRNA"

/isolate="Hc-CRA"

/clone="wt-5"

/note="monepantel-sensitive isolate; wildtype gene, long

transcript"

CDS 1..1605

/note="Hco-DES-2H"

/codon_start=1

/product="H. contortus DES-2 homologue"

/translation="MYALTLTIAAALSAAMVASTPTQIQLVHDLLDKYDKKAKPMWDN

TKPINVSFTVSLYQILELNEPQQFVLLNAWIIERWYDEFLYWSPMEYQNITELWLPYD

SIWLPDTTLYNSLVMKDDDTRRLLNAKLTTDLQRRASLIELLYPTIYKFSCLLDLRFF

PFDVQNCTMIFSSWTYDQTGIDYFPASDEISIANYLENEGWELLKTEVSRHEVKYSCC

PNAYTLLHLTLYLRRKPLFYLVNLIIPTSIITLIAIVGFFTTSSASGMREEKVSLGIT

TLLSMSILMLMVSDQMPTTSTFIPLIGWFILAMIMVISLGTVVSSIIIAVQKRGSLGE

RLSKRTLKIAKMIAYFTCTALPSHIEKGQMMEAFDAVTPTGESVRTLKSAMDASKKWM

SLRRPKNGVAVVSDKSTDALIQMANTGAEDGQMTGMTPVAPLVPPAAILDDDLSLRSD

FSALPPSARLLKTKSSTRCNVFKDLTTSIRHNRQLAVAEFEWLATVTERTCFVIFVLC

FLIITIGINMIGYVHWSKADQRWKNQ"

BASE COUNT 451 a 354 c 341 g 459 t

ORIGIN

1 atgtacgccc tcacactcac cattgctgct gctctatctg cagccatggt tgcaagtaca

61 ccgactcaga ttcagctcgt tcacgatctg ctcgataaat atgacaagaa agcaaaaccg

121 atgtgggaca atacgaaacc gatcaatgtc tcgttcaccg tatcacttta ccagattctt

181 gaactaaacg aaccacagca gttcgtactc cttaacgcct ggatcattga gagatggtat

241 gatgaatttt tgtactggtc ccctatggaa taccaaaata tcactgaatt atggctaccg

301 tatgattcaa tttggttacc ggatactacg ctttacaatt cgttggtaat gaaggatgac

361 gatactagac gccttctgaa tgccaagcta acgacagatc ttcaacggcg agcatctctt

421 attgaacttc tttaccctac aatctacaaa ttctcttgtt tgcttgattt acgatttttc

481 ccatttgatg tgcagaattg tacaatgatt ttttcgtcat ggacctatga tcagacgggg

541 atcgattact tcccagcatc agatgagata tcgattgcta attatttgga aaatgaagga

601 tgggaacttt tgaagactga agtgagccgt catgaagtaa aatacagctg ctgcccgaat

661 gcctacacat tactacattt aacactttat ttgaggagga aaccattatt ctatttggtg

721 aacctgatca ttcctacctc tatcatcaca cttattgcta ttgtgggatt cttcacgact

781 tcttccgcca gtggaatgcg tgaagagaaa gtatcattag gtattactac gttgttatca

841 atgtcgatct tgatgttaat ggtatctgat caaatgccta ccacgtcaac gttcatcccc

901 ctgattggat ggtttatact tgcaatgatc atggtgatct ccctcggaac agttgtctcg

961 tccattatta ttgctgttca aaaacgaggc agtcttggag aaaggctttc aaaacggacg

1021 ttaaagatcg ccaaaatgat tgcgtatttc acttgtacag ctttgccatc tcatattgag

1081 aaggggcaaa tgatggaagc attcgatgct gtgacaccaa caggggaatc agtgaggacc

1141 ttaaagtcag ccatggatgc gtcgaaaaag tggatgagcc ttcgacggcc caaaaacggc

1201 gtcgccgtcg tatccgacaa aagcaccgat gccctgattc agatggcgaa cactggtgca

1261 gaagatggcc aaatgacggg tatgacaccg gtggcacctc tggtaccacc tgcagctatc

1321 ctcgatgatg atctttcgtt acggagtgat ttctcagctt taccaccatc ggcacggctg

1381 ctcaaaacga aatccagtac cagatgtaat gtattcaagg atttaactac gtccattcga

1441 cataatcggc aattggctgt ggctgaattc gaatggctgg ctaccgtcac ggaacgaact

1501 tgctttgtaa tttttgtact gtgtttcctg attatcacca tagggatcaa tatgattggc

1561 tacgttcatt ggagcaaagc agatcaacga tggaaaaatc agtga

//

LOCUS Hco-des-2H;4 1605 bp mRNA linear INV 05-MAR-2009

DEFINITION [gene=Hco-des-2H].

SOURCE Haemonchus contortus

ORGANISM Haemonchus contortus

Eukaryota; Metazoa; Nematoda; Chromadorea; Rhabditida; Strongylida;

Trichostrongyloidea; Haemonchidae; Haemonchinae; Haemonchus.

REFERENCE 1 (bases 1 to 1605)

AUTHORS Rufener,L., Maser,P., Roditi,I. and Kaminsky,R.

TITLE Haemonchus contortus Acetylcholine Receptors of the DEG-3 Subfamily

and their Role in Sensitivity to Monepantel

JOURNAL PLoS Pathogens, in press

REFERENCE 2 (bases 1 to 1605)

AUTHORS Rufener,L., Maser,P., Roditi,I. and Kaminsky,R.

TITLE Direct Submission

JOURNAL Submitted (05-MAR-2009) Institute of Cell Biology, University of

Bern, Baltzerstrasse 4, Bern, BE 3012, Switzerland

FEATURES Location/Qualifiers

source 1..1605

/organism="Haemonchus contortus"

/mol_type="mRNA"

/isolate="Hc-CRA"

/clone="wt-6"

/note="monepantel-sensitive isolate; wildtype gene, long

transcript"

CDS 1..1605

/note="Hco-DES-2H"

/codon_start=1

/product="H. contortus DES-2 homologue"

/translation="MYALTLTIAAALSAAMVASTPTQIQLVHDLLDKYDKKAKPMWDN

TKPINVSFTVSLYQILELNEPQQFVLLNAWIIERWYDEFLYWSPMEYQNITELRLPYD

SIWLPDTTLYNSLVMKDDDTRRLLNAELTTDLQRRASLIELLYPTIYKFSCLLDLRFF

PFDVQNCTMIFSSWTYDQTGIDYFPASDEISIANYLENEGWELLKTEVSRHEVKYSCC

PNAYTLLHLTLYLKRKPLFYLVNLIIPTSIITLIAIVGFFTTSSASGMREEKVSLGIT

TLLSMSILMLMVSDQMPTTSTFIPLIGWFILAMIMVISLGTVVSSIIIAVQKRGSLGE

RLSKRTLKIAKMIAYFTCTALPSHIEKEQMMEAFDAVTPTGESVRTLKSAMDASKKWM

SLRRPKNGVAVVSDKSTDALIQMANSGAEDGQMTGMTPVAPLVPPAAIIDDDLSLRSD

FSALPPSARLLKTKSSTRCNVFKDLTTSIRHNRQLAVAEFEWLATVTERTCFVIFVLC

FLIITIGINMIGYVHWSKADQRWKNQ"

BASE COUNT 453 a 350 c 340 g 462 t

ORIGIN

1 atgtacgccc tcacactcac cattgctgct gctctatctg cagctatggt tgcaagtacg

61 ccgactcaga ttcagctcgt tcacgatctg ctcgataaat atgacaagaa ggcgaaaccg

121 atgtgggaca atacgaaacc gatcaatgtc tcgttcaccg tatcacttta ccagattctt

181 gaactaaacg aaccacagca gttcgtactc ctcaacgcct ggatcattga gagatggtat

241 gatgaattcc tatactggtc ccctatggag taccaaaata tcactgaatt acggctaccg

301 tatgattcaa tttggttgcc ggatactacg ctttacaatt cgttggtaat gaaggatgac

361 gatactagac gccttctgaa tgccgagcta acgacagatc ttcaacggcg agcatctctt

421 attgaacttc tttatcctac aatctacaaa ttctcttgtt tgcttgattt acgatttttc

481 ccatttgatg tgcagaattg tacaatgatt ttttcgtcat ggacctatga tcagacgggg

541 atcgattact tcccagcatc agatgagata tcgattgcta attatttgga aaatgaagga

601 tgggaacttt tgaagactga agtgagccgt catgaagtga aatacagctg ctgcccgaat

661 gcctacacat tactacattt aacactttat ttgaaaagga aaccattgtt ttatttggtg

721 aatctgataa tacctacctc tatcattaca cttattgcta ttgtgggatt cttcacgact

781 tcttctgcca gtggaatgcg tgaagaaaaa gtatcattag ggattacaac gttgttatca

841 atgtcaatct tgatgttaat ggtatctgat caaatgccta ccacgtcaac gttcatcccc

901 ctgattggat ggttcatact tgcaatgatc atggtaatct ccctcggaac agttgtctcg

961 tccattatta ttgctgttca aaaacgagga agtcttggag aaaggctttc aaaacggacg

1021 ttaaagatcg ccaaaatgat tgcatatttc acttgcactg ctctgccttc tcatattgaa

1081 aaggagcaaa tgatggaagc attcgatgct gtgacaccca caggggaatc tgtgaggacg

1141 ttaaagtcgg ctatggatgc gtcgaaaaag tggatgagcc ttcgacggcc taaaaacggc

1201 gtcgccgtcg tatccgacaa aagcaccgat gcccttattc agatggcgaa ctctggtgca

1261 gaagatggcc aaatgacggg tatgacaccg gtggcacctc tggtaccacc tgcagctatc

1321 atcgacgatg atctttcgtt acgaagtgat ttctcagctt taccaccatc agcacggctg

1381 ctcaaaacga aatccagtac cagatgtaat gtattcaagg atttaactac atccattcgg

1441 cataatcggc agttggctgt ggctgaattc gaatggttgg ctaccgtcac ggaacgaact

1501 tgttttgtaa tttttgtact gtgcttcctg attatcacca taggaatcaa tatgattggc

1561 tacgttcatt ggagcaaagc agatcaacga tggaaaaatc agtga

//

LOCUS Hco-des-2H;s1 1450 bp mRNA linear INV 05-MAR-2009

DEFINITION [gene=Hco-des-2H].

SOURCE Haemonchus contortus

ORGANISM Haemonchus contortus

Eukaryota; Metazoa; Nematoda; Chromadorea; Rhabditida; Strongylida;

Trichostrongyloidea; Haemonchidae; Haemonchinae; Haemonchus.

REFERENCE 1 (bases 1 to 1450)

AUTHORS Rufener,L., Maser,P., Roditi,I. and Kaminsky,R.

TITLE Haemonchus contortus Acetylcholine Receptors of the DEG-3 Subfamily

and their Role in Sensitivity to Monepantel

JOURNAL PLoS Pathogens, in press

REFERENCE 2 (bases 1 to 1450)

AUTHORS Rufener,L., Maser,P., Roditi,I. and Kaminsky,R.

TITLE Direct Submission

JOURNAL Submitted (05-MAR-2009) Institute of Cell Biology, University of

Bern, Baltzerstrasse 4, Bern, BE 3012, Switzerland

FEATURES Location/Qualifiers

source 1..1450

/organism="Haemonchus contortus"

/mol_type="mRNA"

/isolate="Hc-CRA"

/clone="wt-s1"

/note="monepantel-sensitive isolate; wildtype gene, short

transcript"

CDS 1..1437

/note="Hco-DES-2H"

/codon_start=1

/product="H. contortus DES-2 homologue"

/translation="MYALTLTIAAALSAAMVASTPTQIQLVHDLLDKYDKKAKPMWDN

TKPINVSFTVSLYQILELNEPQQFILLNAWIIERWYDEFLYWSPMEYQNITELRLPYD

SIWLPDTTLYNSLVMKDDDTRRLLNAKLTTDLQRRASLIELLYPTIYKFSCLLDLRFF

PFDVQNCTMIFSSWTYDQTGIDYFPASDEISIANYLENEGWELLKTEVSRHEVKYSCC

PNAYTLLHLTLYLRRKPLFYLVNLIIPTSIITLIAIVGFFTTSSASGMREEKVSLGIT

TLLSMSILMLMVSDQMPTTSTFIPLIGWFILAMIMVISLGTVVSSIIIAVQKRGSLGE

RLSKRTLKIAKMIAYFTCTALPSHIEKEQMMEAFDAVTPTGESVRTLKSAMDASKKWM

SLRRPKNGVAVVSDKSTDALIQMANSGAASIRHNRQLAVAEFEWLATVTERTCFVIFV

LCFLIITIGINMIGYVHWSKADQRWKNQ"

BASE COUNT 414 a 316 c 301 g 419 t

ORIGIN

1 atgtacgccc tcacactcac cattgctgct gctctatctg cagccatggt tgcaagtaca

61 ccgactcaga ttcagctcgt tcacgatctg ctcgataaat atgataaaaa ggcgaaaccg

121 atgtgggaca atacgaaacc gatcaatgtc tcgttcaccg tatcacttta tcagattctt

181 gaactaaacg aaccgcagca gttcatactt ctcaacgcct ggatcattga gagatggtat

241 gatgaatttt tgtactggtc ccctatggaa taccaaaata tcactgaatt acggctaccg

301 tatgattcaa tttggttacc ggatactacg ctttacaatt cgttggtaat gaaggatgac

361 gatactagac gccttctgaa tgccaagcta acgacagatc ttcaacggcg agcatctctt

421 attgaacttc tttaccctac aatctacaaa ttctcttgtt tgcttgattt acgatttttc

481 ccatttgatg tgcagaattg tacaatgatt ttttcgtcat ggacctatga tcagacgggg

541 atcgattact tcccagcatc agatgagata tcgattgcta attatttgga aaatgaagga

601 tgggaacttt tgaagactga agtaagccgt catgaagtaa aatacagctg ctgcccgaac

661 gcctacacat tactacattt aacactttac ctgagaagaa aaccattatt ctatttggtg

721 aacctgatca ttcctacctc tatcatcaca cttattgcta ttgtgggatt cttcacgact

781 tcttctgcca gtggaatgcg tgaagaaaaa gtatcattag ggattactac gttgttatca

841 atgtcaattt taatgttaat ggtatctgat caaatgccta caacgtcaac gttcatccct

901 ctgattggat ggtttatact tgcaatgatc atggttattt ccctcggaac agtggtctcg

961 tccatcatta ttgcagttca aaagcgaggc agtcttggag aaaggctctc aaaacggacg

1021 ttaaagatcg ccaaaatgat tgcatatttc acttgcactg ctctgccttc tcatattgaa

1081 aaggagcaaa tgatggaagc attcgatgct gtgacaccga caggggaatc agtgaggacg

1141 ttaaagtcag ccatggatgc atcaaaaaag tggatgagcc ttcggcggcc caaaaatggt

1201 gttgccgtcg tatccgacaa aagcaccgat gcccttattc agatggcaaa ctctggtgca

1261 gcgtccattc ggcataatcg gcagttggct gtggctgaat tcgaatggct ggctaccgtc

1321 acggaacgaa cttgtttcgt aatttttgtg ctatgcttcc taatcatcac cataggaatc

1381 aatatgattg gctacgttca ttggagcaaa gcagatcaac gatggaaaaa tcagtgatag

1441 ctcgagcgcc

//

LOCUS Hco-des-2H;s2 1451 bp mRNA linear INV 05-MAR-2009

DEFINITION [gene=Hco-des-2H].

SOURCE Haemonchus contortus

ORGANISM Haemonchus contortus

Eukaryota; Metazoa; Nematoda; Chromadorea; Rhabditida; Strongylida;

Trichostrongyloidea; Haemonchidae; Haemonchinae; Haemonchus.

REFERENCE 1 (bases 1 to 1451)

AUTHORS Rufener,L., Maser,P., Roditi,I. and Kaminsky,R.

TITLE Haemonchus contortus Acetylcholine Receptors of the DEG-3 Subfamily

and their Role in Sensitivity to Monepantel

JOURNAL PLoS Pathogens, in press

REFERENCE 2 (bases 1 to 1451)

AUTHORS Rufener,L., Maser,P., Roditi,I. and Kaminsky,R.

TITLE Direct Submission

JOURNAL Submitted (05-MAR-2009) Institute of Cell Biology, University of

Bern, Baltzerstrasse 4, Bern, BE 3012, Switzerland

FEATURES Location/Qualifiers

source 1..1451

/organism="Haemonchus contortus"

/mol_type="mRNA"

/isolate="Hc-CRA"

/clone="wt-s2"

/note="monepantel-sensitive isolate; wildtype gene, short

transcript"

CDS 1..1437

/note="Hco-DES-2H"

/codon_start=1

/product="H. contortus DES-2 homologue"

/translation="MYALTLTIAAALSAAMVASTPTQIQLVHDLLDKYDKKAKPMWDN

TKPINVSFTVSLYQILELNEPQQFVLLNAWIIERWYDEFLYWSPMEYQNITELRLPYD

SIWLPDTTLYNSLVMKDDDTRRLLNAKLTTDLQRRASLIELLYPTIYKFSCLLDLRFF

PFDVQNCTMIFSSWTYDQTGIDYFPASDEISIANYLENEGWELLKTEVSRHEVKYSCC

PNAYTLLHLTLYLRRKPLFYLVNLIIPTSIITLIAIVGFFTTSSASGMREEKVSLGIT

TLLSMSILMLMVSDQMPTTSTFIPLIGWFILAMIMVISLGTVVSSIIIAVQKRGSLGE

RLSKRTLKIAKMIAYFTCTALPSHIEKEQMMEAFDAVTPTGESVRTLKSAMDASKKWM

SLRRPKNGVAVVSDKSTDALIQMANSGAASIRHNRQLAVAEFEWLATVTERTCFVIFV

LCFLIITIGINMIGYVHWSKADQRWKNQ"

BASE COUNT 412 a 324 c 302 g 413 t

ORIGIN

1 atgtacgccc tcacgctcac aatcgctgct gctctatctg cagccatggt tgcaagtaca

61 ccgactcaga tccagctcgt tcacgatctg ctcgataagt atgacaaaaa ggcgaaaccg

121 atgtgggaca atacaaaacc gatcaatgtc tcgttcaccg tatcacttta ccagattctt

181 gaactaaacg aaccacagca attcgtactc ctcaacgcct ggatcattga gagatggtat

241 gatgaattcc tatattggtc ccctatggag taccaaaata tcactgaatt acggctaccg

301 tatgattcaa tttggttgcc ggatactacg ctttacaatt cgttggtaat gaaggatgac

361 gatactagac gccttctgaa tgccaagcta acgacagatc ttcaacggcg agcatctctt

421 attgaacttc tttaccctac aatctacaaa ttctcttgtt tgcttgattt acgatttttc

481 ccatttgatg tgcagaattg tacaatgatt ttttcgtcat ggacctatga tcagacaggg

541 atcgattact tcccagcatc agatgagata tcgattgcta attatttgga aaatgaagga

601 tgggaacttt tgaagactga agtgagccgc catgaagtaa aatacagctg ctgcccgaat

661 gcctacacat tactacattt aacactttat ttgagaagaa aaccgttatt ctatctggtg

721 aatctgatca ttcctacctc tatcatcaca cttattgcta ttgtgggatt cttcacgaca

781 tcctctgcca gtggaatgcg tgaagagaaa gtatcattag ggattactac gttgttatca

841 atgtcaatct taatgttaat ggtatctgat caaatgccta ccacgtcaac gttcatcccc

901 ctgattggat ggtttatact tgcaatgatc atggtaatct ccctcggaac agtggtctcg

961 tccattatta ttgcagttca aaagcgaggc agtcttggag agagactttc aaaacggacg

1021 ttaaagatcg ccaaaatgat tgcatatttc acttgcactg ctcttccttc tcatattgaa

1081 aaggagcaaa tgatggaagc attcgatgcc gtgacaccaa caggggaatc tgtaaggacc

1141 ttaaagtcgg ccatggatgc atcgaaaaag tggatgagcc ttcgacggcc caaaaatggc

1201 gtcgccgtcg tatccgacaa aagcaccgat gcccttattc aaatggcgaa ctctggtgca

1261 gcgtccattc ggcataatcg gcagctggct gtggctgaat tcgaatggtt ggctaccgtc

1321 acggaacgaa cttgttttgt aatttttgta ctgtgcttcc tgattatcac cataggaatc

1381 aatatgattg gctacgttca ttggagcaaa gcagatcaac gatggaaaaa tcagtgatat

1441 gctcgagcgc c

//

LOCUS Hco-des-2H;s3 1451 bp mRNA linear INV 05-MAR-2009

DEFINITION [gene=Hco-des-2H].

SOURCE Haemonchus contortus

ORGANISM Haemonchus contortus

Eukaryota; Metazoa; Nematoda; Chromadorea; Rhabditida; Strongylida;

Trichostrongyloidea; Haemonchidae; Haemonchinae; Haemonchus.

REFERENCE 1 (bases 1 to 1451)

AUTHORS Rufener,L., Maser,P., Roditi,I. and Kaminsky,R.

TITLE Haemonchus contortus Acetylcholine Receptors of the DEG-3 Subfamily

and their Role in Sensitivity to Monepantel

JOURNAL PLoS Pathogens, in press

REFERENCE 2 (bases 1 to 1451)

AUTHORS Rufener,L., Maser,P., Roditi,I. and Kaminsky,R.

TITLE Direct Submission

JOURNAL Submitted (05-MAR-2009) Institute of Cell Biology, University of

Bern, Baltzerstrasse 4, Bern, BE 3012, Switzerland

FEATURES Location/Qualifiers

source 1..1451

/organism="Haemonchus contortus"

/mol_type="mRNA"

/isolate="Hc-CRA"

/clone="wt-s3"

/note="monepantel-sensitive isolate; wildtype gene, short

transcript"

CDS 1..1437

/note="Hco-DES-2H"

/codon_start=1

/product="H. contortus DES-2 homologue"

/translation="MYALTLTIAAALSAAMVASTPTQIQLVHDLLDKYDKKAKPMWDN

TKPINVSFTVSLYQILELNEPQQFVLLNAWIIERWYDEFLYWSPMEYQNITELRLPYD

SIWLPDTTLYNSLVMKDDDTRRLLNAKLTTDLQRRASLIELLYPTIYKFSCLLDLRFF

PFDVQNCTMIFSSWTYDQTGIDYFPASDEISIANYLENEGWELLKTEVSRHEVKYSCC

PNAYTLLHLTLYLRRKPLFYLVNLIIPTSIITLIAIVGFFTTSSASGMREEKVSLGIT

TLLSMSILMLMVSDQMPTTSTFIPLIGWFILAMIMVISLGTVVSSIIIAVQKRGSLGE

RLSKRTLRIAKMIAYFTCTALPSHIEKEQMMEAFDAVTPTGESVRTLKSAMDASKKWV

SLRRPKNGVAVVSDKSTDALIQMANSGAASIRHNRQLAVAEFEWLATVTERTCFVIFV

LCFLIITIGINMIGYVHWSKADQRWKNQ"

BASE COUNT 409 a 322 c 305 g 415 t

ORIGIN

1 atgtacgccc tcacactcac cattgctgct gctctatctg cagctatggt tgcaagtacg

61 ccgactcaga tccagcttgt tcacgatctg ctcgataaat atgacaaaaa ggcgaaaccg

121 atgtgggaca atacgaaacc gatcaatgtc tcgttcaccg tatcacttta ccagattctt

181 gaactaaacg aaccacagca attcgtactc ctcaacgcct ggatcattga gagatggtat

241 gatgaattcc tatactggtc ccctatggag taccaaaata tcactgaatt acggctaccg

301 tatgattcaa tttggttgcc ggatactacg ctttacaatt cgttggtaat gaaggatgac

361 gatactagac gccttctgaa tgccaagcta acgacagatc ttcaacggcg agcatctctt

421 attgaacttc tttatcctac aatctacaaa ttctcttgtt tgcttgattt acgatttttc

481 ccatttgatg tgcagaattg tacaatgatt ttttcgtcat ggacctatga tcagacgggg

541 atcgattact tcccagcatc agatgagata tcgattgcta attatttgga aaatgaagga

601 tgggaacttt tgaagactga agtgagccgc catgaagtaa aatacagctg ctgcccgaat

661 gcctacacat tactacattt aacactttat ttgagaagaa aaccgttatt ctatctggtg

721 aatctgatca ttcctacctc tatcatcaca cttatcgcta ttgtgggatt cttcacgaca

781 tcctctgcca gtggaatgcg tgaagagaaa gtatcattag ggattactac gttgttatca

841 atgtcaatct taatgttaat ggtatctgat caaatgccta ccacgtcaac gttcatcccc

901 ctgattggat ggttcatact tgcaatgatc atggtaatct ccctcggaac agttgtctcg

961 tccattatta ttgctgttca aaaacgagga agtcttggag aaaggctttc aaaacggacg

1021 ttaaggatcg ccaaaatgat tgcatatttc acttgcactg ctctgccttc tcatattgaa

1081 aaggagcaaa tgatggaagc attcgatgcc gtgacaccaa caggggaatc tgtgaggacg

1141 ttaaagtcgg ccatggatgc atcaaaaaag tgggtgagcc ttcgacggcc caaaaacggc

1201 gtcgccgtcg tatccgacaa aagcaccgat gcccttattc agatggcgaa ctctggtgca

1261 gcatccattc ggcataatcg gcagttggct gtggctgaat tcgaatggtt ggctaccgtc

1321 acggaacgaa cttgttttgt aatttttgta ctgtgcttcc tgattatcac cataggaatc

1381 aatatgattg gctacgttca ttggagcaaa gcagatcaac gatggaaaaa tcagtgatat

1441 gctcgagcgc c

//

LOCUS Hco-des-2H;7 1605 bp mRNA linear INV 05-MAR-2009

DEFINITION [gene=Hco-des-2H].

SOURCE Haemonchus contortus

ORGANISM Haemonchus contortus

Eukaryota; Metazoa; Nematoda; Chromadorea; Rhabditida; Strongylida;

Trichostrongyloidea; Haemonchidae; Haemonchinae; Haemonchus.

REFERENCE 1 (bases 1 to 1605)

AUTHORS Rufener,L., Maser,P., Roditi,I. and Kaminsky,R.

TITLE Haemonchus contortus Acetylcholine Receptors of the DEG-3 Subfamily

and their Role in Sensitivity to Monepantel

JOURNAL PLoS Pathogens, in press

REFERENCE 2 (bases 1 to 1605)

AUTHORS Rufener,L., Maser,P., Roditi,I. and Kaminsky,R.

TITLE Direct Submission

JOURNAL Submitted (05-MAR-2009) Institute of Cell Biology, University of

Bern, Baltzerstrasse 4, Bern, BE 3012, Switzerland

FEATURES Location/Qualifiers

source 1..1605

/organism="Haemonchus contortus"

/mol_type="mRNA"

/isolate="Hc-CRA AAD-mutant"

/clone="wt-7"

/note="monepantel-insensitive isolate; wildtype gene, long

transcript"

CDS 1..1605

/note="Hco-DES-2H"

/codon_start=1

/product="H. contortus DES-2 homologue"

/translation="MYALTLTIAAALSAAMVASTPTQIQLVHDLLDKYDKKAKPMWDN

TKPINVSFTVSLYQILELNEPQQFVLLNAWIIERWYDEFLYWSPMEYQNITELRLPYD

SIWLPDTTLYNSLVMKDDDTRRLLNAKLTTDLQRRASLIELLYPTIYKFSCLLDLRFF

PFDVQNCTMIFSSWTYDQTGIDYFPASDEISIANYLENEGWELLKTEVSRHEVKYSCC

PNAYTLLHLTLYLRRKPLFYLVNLIIPTSIITLIAIVGFFTTSSASGMREEKVSLGIT

TLLSMSILMLMVSDQMPTTSTFIPLIGWFILAMIMVISLGTVVSSIIIAVQKRGSLGE

RLSKRTLRIAKMIAYFTCTALPSHIEKEQMMEAFDAVTPTGESVRTLKSAMDASKKWM

SLRRPKNGVAVVSDKSTDALIQMANSGAEDGQMTGMTPVAPLVPPAAIIDDDLSLRSD

FSALPPSARLLKTKSSTRCNVFKDLTTSIRHNRQLAVAEFEWLATVTERTCFVIFVLC

FLIITIGINMIGYVHWSKADQRWKNQ"

BASE COUNT 451 a 355 c 338 g 461 t

ORIGIN

1 atgtacgccc tcacactcac cattgctgct gctctatctg cagccatggt tgcaagtaca

61 ccaactcaga tccagcttgt tcacgatctg cttgataaat acgacaagaa ggcgaaacca

121 atgtgggaca atacgaaacc gatcaatgtc tcgttcaccg tatcacttta ccagattctt

181 gaactaaacg aaccacagca gttcgtactc ctcaacgcct ggattattga gagatggtat

241 gatgaatttt tgtactggtc ccctatggag taccaaaata tcactgaatt acggctaccg

301 tatgattcaa tttggttacc ggatactacg ctttacaatt cgttggtaat gaaggatgac

361 gatactagac gccttctgaa tgccaagcta acgacagatc ttcaacggcg agcatctctt

421 attgaacttc tttatcctac aatctacaaa ttctcttgtc tacttgacct acgatttttc

481 ccatttgatg tacagaattg tacaatgatt ttttcgtcat ggacctatga tcagacaggg

541 atcgattact ttcctgcttc agatgaaata tcgattgcta attatttgga aaatgaagga

601 tgggaacttt tgaagactga agtgagccgt catgaagtaa aatacagctg ctgtccgaat

661 gcctacacat tactacattt aacactttat ttgagaagga agccattatt ctatttggtg

721 aacctgatca ttcctacctc tatcatcaca cttattgcta ttgtgggatt cttcacgact

781 tcttctgcca gtggaatgcg tgaagagaaa gtatcattag gtattactac gttgttatca

841 atgtcgatct tgatgttaat ggtatctgat caaatgccta ccacgtcaac gttcatcccc

901 ctgattggat ggtttatact tgcaatgatc atggtaatct ctctcggaac agtggtctcg

961 tccatcatta ttgcagttca aaagcgaggc agtcttggag agagactttc aaagcggacg

1021 ttaaggatcg ccaaaatgat tgcatatttc acttgcactg ctctgccttc tcatattgaa

1081 aaggagcaaa tgatggaagc attcgatgct gtgacaccca caggggaatc agtgaggacg

1141 ttaaagtcgg ccatggatgc atcaaaaaag tggatgagcc ttcgacggcc caaaaacggc

1201 gtcgccgtcg tatccgacaa aagcaccgat gcccttattc agatggcgaa ctctggtgca

1261 gaagatggcc aaatgacggg tatgacaccg gtggcacctc tggtaccacc tgcagctatc

1321 atcgacgatg atctttcgtt acgaagtgat ttctcagctt taccaccatc agcacggctg

1381 ctcaaaacga aatccagtac cagatgtaat gtattcaagg atttaaccac gtccattcgg

1441 cataatcggc agttggctgt ggctgaattc gaatggctgg ctaccgtcac ggaacggact

1501 tgttttgtaa tttttgtgct gtgtttcctg attatcacta taggaatcaa tatgattggc

1561 tacgttcatt ggagcaaagc agatcaacga tggaaaaatc agtga

//

LOCUS Hco-des-2H;8 1605 bp mRNA linear INV 05-MAR-2009

DEFINITION [gene=Hco-des-2H].

SOURCE Haemonchus contortus

ORGANISM Haemonchus contortus

Eukaryota; Metazoa; Nematoda; Chromadorea; Rhabditida; Strongylida;

Trichostrongyloidea; Haemonchidae; Haemonchinae; Haemonchus.

REFERENCE 1 (bases 1 to 1605)

AUTHORS Rufener,L., Maser,P., Roditi,I. and Kaminsky,R.

TITLE Haemonchus contortus Acetylcholine Receptors of the DEG-3 Subfamily

and their Role in Sensitivity to Monepantel

JOURNAL PLoS Pathogens, in press

REFERENCE 2 (bases 1 to 1605)

AUTHORS Rufener,L., Maser,P., Roditi,I. and Kaminsky,R.

TITLE Direct Submission

JOURNAL Submitted (05-MAR-2009) Institute of Cell Biology, University of

Bern, Baltzerstrasse 4, Bern, BE 3012, Switzerland

FEATURES Location/Qualifiers

source 1..1605

/organism="Haemonchus contortus"

/mol_type="mRNA"

/isolate="Hc-CRA AAD-mutant"

/clone="wt-8"

/note="monepantel-insensitive isolate; wildtype gene, long

transcript"

CDS 1..1605

/note="Hco-DES-2H"

/codon_start=1

/product="H. contortus DES-2 homologue"

/translation="MYALTLTIAAALSAAMVASTPTQIQLVHDLLDKYDKKAKPMWDN

TKPINVSFTVSLYQILELNEPQQFVLLNAWIIERWYDEFLYWSPMEYQNITELRLPYD

SIWLPDTTLYNSLVMKDDDTRRLLNAKLTTDLQRRASLIELLYPTIYKFSCLLDLRFF

PFDVQNCTMIFSSWTYDQTGIDYFPASDEISIANYLENEGWELLKTEVSRHEVKYSCC

PNAYTLLHLTLYLRRKPLFYLVNLIIPTSIITLIAIVGFFTTSSASGMREEKVSLGIT

TLLSMSILMLMVSDQMPTTSTFIPLIGWFILAMIMVISLGTVVSSIIIAVQKRGSLGE

RLSKRTLRIAKMIAYFTCTALPSHIEKEQMMEAFDAVTPTGESVRTLKSAMDASKKWM

SLRRSKNGVAVVSDKSTDALIQMANSGAEDGQMTGMTPVAPLVPPAAIIDDDLSLRSD

FSALPPSARLLKTKSSTRCNVFKDLTTSIRHNRQLAVAEFEWLATVTERTCFVIFVLC

FLIITIGINMIGYVHWSKADQRWKNQ"

BASE COUNT 451 a 354 c 338 g 462 t

ORIGIN

1 atgtacgccc tcacactcac cattgctgct gctctatctg cagccatggt tgcaagtaca

61 ccaactcaga tccagcttgt tcacgatctg cttgataaat acgacaagaa ggcgaaacca

121 atgtgggaca atacgaaacc gatcaatgtc tcgttcaccg tatcacttta ccagattctt

181 gaactaaacg aaccacagca gttcgtactc ctcaacgcct ggattattga gagatggtat

241 gatgaatttt tgtactggtc ccctatggag taccaaaata tcactgaatt acggctaccg

301 tatgattcaa tttggttacc ggatactacg ctttacaatt cgttggtaat gaaggatgac

361 gatactagac gccttctgaa tgccaagcta acgacagatc ttcaacggcg agcatctctt

421 attgaacttc tttatcctac aatctacaaa ttctcttgtc tacttgacct acgatttttc

481 ccatttgatg tacagaattg tacaatgatt ttttcgtcat ggacctatga tcagacaggg

541 atcgattact ttcctgcttc agatgaaata tcgattgcta attatttgga aaatgaagga

601 tgggaacttt tgaagactga agtgagccgt catgaagtaa aatacagctg ctgtccgaat

661 gcctacacat tactacattt aacactttat ttgagaagga agccattatt ctatttggtg

721 aacctgatca ttcctacctc tatcatcaca cttattgcta ttgtgggatt cttcacgact

781 tcttctgcca gtggaatgcg tgaagagaaa gtatcattag gtattactac gttgttatca

841 atgtcgatct tgatgttaat ggtatctgat caaatgccta ccacgtcaac gttcatcccc

901 ctgattggat ggtttatact tgcaatgatc atggtaatct ctctcggaac agtggtctcg

961 tccatcatta ttgcagttca aaagcgaggc agtcttggag agagactttc aaagcggacg

1021 ttaaggatcg ccaaaatgat tgcatatttc acttgcactg ctctgccttc tcatattgaa

1081 aaggagcaaa tgatggaagc attcgatgct gtgacaccca caggggaatc agtgaggacg

1141 ttaaagtcgg ccatggatgc atcaaaaaag tggatgagcc ttcgacggtc caaaaacggc

1201 gtcgccgtcg tatccgacaa aagcaccgat gcccttattc agatggcgaa ctctggtgca

1261 gaagatggcc aaatgacggg tatgacaccg gtggcacctc tggtaccacc tgcagctatc

1321 atcgacgatg atctttcgtt acgaagtgat ttctcagctt taccaccatc agcacggctg

1381 ctcaaaacga aatccagtac cagatgtaat gtattcaagg atttaaccac gtccattcgg

1441 cataatcggc agttggctgt ggctgaattc gaatggctgg ctaccgtcac ggaacggact

1501 tgttttgtaa tttttgtgct gtgtttcctg attatcacta taggaatcaa tatgattggc

1561 tacgttcatt ggagcaaagc agatcaacga tggaaaaatc agtga

//

LOCUS Hco-des-2H;9 1605 bp mRNA linear INV 05-MAR-2009

DEFINITION [gene=Hco-des-2H].

SOURCE Haemonchus contortus

ORGANISM Haemonchus contortus

Eukaryota; Metazoa; Nematoda; Chromadorea; Rhabditida; Strongylida;

Trichostrongyloidea; Haemonchidae; Haemonchinae; Haemonchus.

REFERENCE 1 (bases 1 to 1605)

AUTHORS Rufener,L., Maser,P., Roditi,I. and Kaminsky,R.

TITLE Haemonchus contortus Acetylcholine Receptors of the DEG-3 Subfamily

and their Role in Sensitivity to Monepantel

JOURNAL PLoS Pathogens, in press

REFERENCE 2 (bases 1 to 1605)

AUTHORS Rufener,L., Maser,P., Roditi,I. and Kaminsky,R.

TITLE Direct Submission

JOURNAL Submitted (05-MAR-2009) Institute of Cell Biology, University of

Bern, Baltzerstrasse 4, Bern, BE 3012, Switzerland

FEATURES Location/Qualifiers

source 1..1605

/organism="Haemonchus contortus"

/mol_type="mRNA"

/isolate="Hc-CRA AAD-mutant"

/clone="wt-9"

/note="monepantel-insensitive isolate; wildtype gene, long

transcript"

CDS 1..1605

/note="Hco-DES-2H"

/codon_start=1

/product="H. contortus DES-2 homologue"

/translation="MYALTLTIAAALSAAMVASTPTQIQLVHDLLDKYDKKAKPMWDN

TKPINVSFTVSLYQILELNEPQQFVLLNAWIIERWYDEFLYWSPMEYQNITELRLPYD

SIWLPDTTLYNSLVMKDDDTRRLLNAKLTTDLQRRASLIELLYPTIYKFSCLLDLRFF

PFDVQNCTMIFSSWTYDQTGIDYLPASDEISIANYLENEGWELLKTEVSRHEVKYSCC

PNAYTLLHLTLYLRRKPLFYLVNLIIPTSIITLIAIVGFFTTSSASGMREEKVSLGIT

TLLSMSILMLMVSDQMPTTSTFIPLIGWCILAMIMVISLGTVVSSIIIAVQKRGSLGE

RLSKRTLRIAKMIAYFTCTALPSHIEKEQMMEAFDAVTPTGESVRTLKSAMDASKKWM

SLRRPKNGVAVVSDKSTDALIQMANSGAEGGQMTGMTPVAPLVPPAAIIDDDLSLRSD

FSALPPSARLLKTKSSTRCNVFKDLTTSIRHNRQLAVAEFEWLATVTERTCFVIFVLC

FLIITIGINMIGYVHWSKADQRWKNQ"

BASE COUNT 450 a 356 c 340 g 459 t

ORIGIN

1 atgtacgccc tcacactcac cattgctgct gctctatctg cagccatggt tgcaagtaca

61 ccaactcaga tccagcttgt tcacgatctg cttgataaat acgacaagaa ggcgaaacca

121 atgtgggaca atacgaaacc gatcaatgtc tcgttcaccg tatcacttta ccagattctt

181 gaactaaacg aaccacagca gttcgtactc ctcaacgcct ggattattga gagatggtat

241 gatgaatttt tgtactggtc ccctatggag taccaaaata tcactgaatt acggctaccg

301 tatgattcaa tttggttacc ggatactacg ctttacaatt cgttggtaat gaaggatgac

361 gatactagac gccttctgaa tgccaagcta acgacagatc ttcaacggcg agcatctctt

421 attgaacttc tttatcctac aatctacaaa ttctcttgtc tacttgacct acgatttttc

481 ccatttgatg tacagaattg tacaatgatt ttttcgtcat ggacctatga tcagacaggg

541 atcgattacc ttcctgcttc agatgaaata tcgattgcta attatttgga aaatgaagga

601 tgggaacttt tgaagactga agtgagccgt catgaagtaa aatacagctg ctgtccgaat

661 gcctacacat tactacattt aacactttat ttgagaagga agccattatt ctatttggtg

721 aacctgatca ttcctacctc tatcatcaca cttattgcta ttgtgggatt cttcacgact

781 tcttctgcca gtggaatgcg tgaagagaaa gtatcattag gtattactac gttgttatca

841 atgtcgatct tgatgttaat ggtatctgat caaatgccta ccacgtcaac gttcatcccc

901 ctgattggat ggtgtatact tgcaatgatc atggtaatct ctctcggaac agtggtctcg

961 tccatcatta ttgcagttca aaagcgaggc agtcttggag agagactttc aaagcggacg

1021 ttaaggatcg ccaaaatgat tgcatatttc acttgcactg ctctgccttc tcatattgaa

1081 aaggagcaaa tgatggaagc attcgatgct gtgacaccca caggggaatc agtgaggacg

1141 ttaaagtcgg ccatggatgc atcaaaaaag tggatgagcc ttcgacggcc caaaaacggc

1201 gtcgccgtcg tatccgacaa aagcaccgat gcccttattc agatggcgaa ctctggtgca

1261 gaaggtggcc aaatgacggg tatgacaccg gtggcacctc tggtaccacc tgcagctatc

1321 atcgacgatg atctttcgtt acgaagtgat ttctcagctt taccaccatc agcacggctg

1381 ctcaaaacga aatccagtac cagatgtaat gtattcaagg atttaaccac gtccattcgg

1441 cataatcggc agttggctgt ggctgaattc gaatggctgg ctaccgtcac ggaacggact

1501 tgttttgtaa tttttgtgct gtgtttcctg attatcacta taggaatcaa tatgattggc

1561 tacgttcatt ggagcaaagc agatcaacga tggaaaaatc agtga

//

LOCUS Hco-des-2H;10 1605 bp mRNA linear INV 05-MAR-2009

DEFINITION [gene=Hco-des-2H].

SOURCE Haemonchus contortus

ORGANISM Haemonchus contortus

Eukaryota; Metazoa; Nematoda; Chromadorea; Rhabditida; Strongylida;

Trichostrongyloidea; Haemonchidae; Haemonchinae; Haemonchus.

REFERENCE 1 (bases 1 to 1605)

AUTHORS Rufener,L., Maser,P., Roditi,I. and Kaminsky,R.

TITLE Haemonchus contortus Acetylcholine Receptors of the DEG-3 Subfamily

and their Role in Sensitivity to Monepantel

JOURNAL PLoS Pathogens, in press

REFERENCE 2 (bases 1 to 1605)

AUTHORS Rufener,L., Maser,P., Roditi,I. and Kaminsky,R.

TITLE Direct Submission

JOURNAL Submitted (05-MAR-2009) Institute of Cell Biology, University of

Bern, Baltzerstrasse 4, Bern, BE 3012, Switzerland

FEATURES Location/Qualifiers

source 1..1605

/organism="Haemonchus contortus"

/mol_type="mRNA"

/isolate="Hc-CRA AAD-mutant"

/clone="wt-10"

/note="monepantel-insensitive isolate; wildtype gene, long

transcript"

CDS 1..1605

/note="Hco-DES-2H"

/codon_start=1

/product="H. contortus DES-2 homologue"

/translation="MYALTLTIAAALSAAMVASTPTQIQLVHDLLDKYDKKAKPMWDN

TKPINVSFTVSLYQILELNEPQQFVLLNAWIIERWYDEFLYWSPMEYQNITELRLPYD

SIWLPDTTLYNSLVMKDDDTRRLLNAKLTTDLQRRASLIELLYPTIYKFSCLLDLRFF

PFDVQNCTMIFSSWTYDQTGIDYFPASDEISIANYLENEGWELLKTEVSRHEVKYSCC

PNAYTLLHLTLYLRRKPLFYLVNLIIPTSIITLIAIVGFFTTSSASGMREEKVSLGIT

TLLSMSILMLVVSDQMPTTSTFIPLIGWFILAMIMVISLGTVVSSIIIAVQKRGSLGE

RLSKRTLRIAKMIAYFTCTALPSHIEKEQMMEAFDAVTPTGESVRTLKSAMDASKKWM

SLRRPKNGVAVVSDKSTDALIQMANSGAEDGQMTGMTPVAPLVPPAATIDDDLSLRSD

FSALPPSARLLKTKSSTRCNVFKDLTTSIRHNRQLAVAEFEWLATVTERTCFVIFVLC

FLIITIGINMIGYVHWSKADQRWKNQ"

BASE COUNT 450 a 356 c 339 g 460 t

ORIGIN

1 atgtacgccc tcacactcac cattgctgct gctctatctg cagccatggt tgcaagtaca

61 ccaactcaga tccagcttgt tcacgatctg cttgataaat acgacaagaa ggcgaaacca

121 atgtgggaca atacgaaacc gatcaatgtc tcgttcaccg tatcacttta ccagattctt

181 gaactaaacg aaccacagca gttcgtactc ctcaacgcct ggattattga gagatggtat

241 gatgaatttt tgtactggtc ccctatggag taccaaaata tcactgaatt acggctaccg

301 tatgattcaa tttggttacc ggatactacg ctttacaatt cgttggtaat gaaggatgac

361 gatactagac gccttctgaa tgccaagcta acgacagatc ttcaacggcg agcatctctt

421 attgaacttc tttatcctac aatctacaaa ttctcttgtc tacttgacct acgatttttc

481 ccatttgatg tacagaattg tacaatgatt ttttcgtcat ggacctatga tcagacaggg

541 atcgattact ttcctgcttc agatgaaata tcgattgcta attatttgga aaatgaagga

601 tgggaacttt tgaagactga agtgagccgt catgaagtaa aatacagctg ctgtccgaat

661 gcctacacat tactacattt aacactttat ttgagaagga agccattatt ctatttggtg

721 aacctgatca ttcctacctc tatcatcaca cttattgcta ttgtgggatt cttcacgact

781 tcttctgcca gtggaatgcg tgaagagaaa gtatcattag gtattactac gttgttatca

841 atgtcgatct tgatgttagt ggtatctgat caaatgccta ccacgtcaac gttcatcccc

901 ctgattggat ggtttatact tgcaatgatc atggtaatct ctctcggaac agtggtctcg

961 tccatcatta ttgcagttca aaagcgaggc agtcttggag agagactttc aaagcggacg

1021 ttaaggatcg ccaaaatgat tgcatatttc acttgcactg ctctgccttc tcatattgaa

1081 aaggagcaaa tgatggaagc attcgatgct gtgacaccca caggggaatc agtgaggacg

1141 ttaaagtcgg ccatggatgc atcaaaaaag tggatgagcc ttcgacggcc caaaaacggc

1201 gtcgccgtcg tatccgacaa aagcaccgat gcccttattc agatggcgaa ctctggtgca

1261 gaagatggcc aaatgacggg tatgacaccg gtggcacctc tggtaccacc tgcagctacc

1321 atcgacgatg atctttcgtt acgaagtgat ttctcagctt taccaccatc agcacggctg

1381 ctcaaaacga aatccagtac cagatgtaat gtattcaagg atttaaccac gtccattcgg

1441 cataatcggc agttggctgt ggctgaattc gaatggctgg ctaccgtcac ggaacggact

1501 tgttttgtaa tttttgtgct gtgtttcctg attatcacta taggaatcaa tatgattggc

1561 tacgttcatt ggagcaaagc agatcaacga tggaaaaatc agtga

//

LOCUS Hco-des-2H;11 1602 bp mRNA linear INV 05-MAR-2009

DEFINITION [gene=Hco-des-2H].

SOURCE Haemonchus contortus

ORGANISM Haemonchus contortus

Eukaryota; Metazoa; Nematoda; Chromadorea; Rhabditida; Strongylida;

Trichostrongyloidea; Haemonchidae; Haemonchinae; Haemonchus.

REFERENCE 1 (bases 1 to 1602)

AUTHORS Rufener,L., Maser,P., Roditi,I. and Kaminsky,R.

TITLE Haemonchus contortus Acetylcholine Receptors of the DEG-3 Subfamily

and their Role in Sensitivity to Monepantel

JOURNAL PLoS Pathogens, in press

REFERENCE 2 (bases 1 to 1602)

AUTHORS Rufener,L., Maser,P., Roditi,I. and Kaminsky,R.

TITLE Direct Submission

JOURNAL Submitted (05-MAR-2009) Institute of Cell Biology, University of

Bern, Baltzerstrasse 4, Bern, BE 3012, Switzerland

FEATURES Location/Qualifiers

source 1..1602

/organism="Haemonchus contortus"

/mol_type="mRNA"

/isolate="Hc-CRA AAD-mutant"

/clone="wt-11"

/note="monepantel-insensitive isolate; wildtype gene, long

transcript"

CDS 1..1602

/note="Hco-DES-2H"

/codon_start=1

/product="H. contortus DES-2 homologue"

/translation="MYALTLTIAAALSAAMVASTPTQIQLVHDLLDKYDKKAKPMWDN

TKPINVSFTVSLYQILELNEPQQFVLLNAWIIERWYDEFLYWSPMEYQNITELRLPYD

SIWLPDTTLYNSLVMKDDDTRRLLNAKLTTDLQRRASLIELLYPTIYKFSCLLDLRFF

PFDVQNCTMIFSSWTYDQTGIDYFPASDEISIANYLENEGWELLKTEVSRHEVKYSCC

PNAYTLLHLTLYLRRKPLFYLVNLIIPTSIITLIAIVGFFTTSSASGMREEKVSLGIT

TLLSMSILMLMVSDQMPTTSTFIPLIGWFILAMIMVISLGTVVSSIIIAVQKRGSLGE

RLSKRTLRIAKMIAYFTCTALPSHIEKEQMMEAFDAVTPTGESVRTLKSAMDASKKWM

SLRRPKNGVAVVSDKSTDALIQMANSGADGQMTGMTPVAPLVPPAAIIDDDLSLRSDF

SALPPSARLLKTKSSTRCNVFKDLTTSIRHNRQLAVAEFEWLATVTERTCFVIFVLCF

LIITIGINMIGYVHWSKADQRWKNQ"

BASE COUNT 449 a 355 c 337 g 461 t

ORIGIN

1 atgtacgccc tcacactcac cattgctgct gctctatctg cagccatggt tgcaagtaca

61 ccaactcaga tccagcttgt tcacgatctg cttgataaat acgacaagaa ggcgaaacca

121 atgtgggaca atacgaaacc gatcaatgtc tcgttcaccg tatcacttta ccagattctt

181 gaactaaacg aaccacagca gttcgtactc ctcaacgcct ggattattga gagatggtat

241 gatgaatttt tgtactggtc ccctatggag taccaaaata tcactgaatt acggctaccg

301 tatgattcaa tttggttacc ggatactacg ctttacaatt cgttggtaat gaaggatgac

361 gatactagac gccttctgaa tgccaagcta acgacagatc ttcaacggcg agcatctctt

421 attgaacttc tttatcctac aatctacaaa ttctcttgtc tacttgacct acgatttttc

481 ccatttgatg tacagaattg tacaatgatt ttttcgtcat ggacctatga tcagacaggg

541 atcgattact ttcctgcttc agatgaaata tcgattgcta attatttgga aaatgaagga

601 tgggaacttt tgaagactga agtgagccgt catgaagtaa aatacagctg ctgtccgaat

661 gcctacacat tactacattt aacactttat ttgagaagga agccattatt ctatttggtg

721 aacctgatca ttcctacctc tatcatcaca cttattgcta ttgtgggatt cttcacgact

781 tcttctgcca gtggaatgcg tgaagagaaa gtatcattag gtattactac gttgttatca

841 atgtcgatct tgatgttaat ggtatctgat caaatgccta ccacgtcaac gttcatcccc

901 ctgattggat ggtttatact tgcaatgatc atggtaatct ctctcggaac agtggtctcg

961 tccatcatta ttgcagttca aaagcgaggc agtcttggag agagactttc aaagcggacg

1021 ttaaggatcg ccaaaatgat tgcatatttc acttgcactg ctctgccttc tcatattgaa

1081 aaggagcaaa tgatggaagc attcgatgct gtgacaccca caggggaatc agtgaggacg

1141 ttaaagtcgg ccatggatgc atcaaaaaag tggatgagcc ttcgacggcc caaaaacggc

1201 gtcgccgtcg tatccgacaa aagcaccgat gcccttattc agatggcgaa ctctggtgca

1261 gatggccaaa tgacgggtat gacaccggtg gcacctctgg taccacctgc agctatcatc

1321 gacgatgatc tttcgttacg aagtgatttc tcagctttac caccatcagc acggctgctc

1381 aaaacgaaat ccagtaccag atgtaatgta ttcaaggatt taaccacgtc cattcggcat

1441 aatcggcagt tggctgtggc tgaattcgaa tggctggcta ccgtcacgga acggacttgt

1501 tttgtaattt ttgtgctgtg tttcctgatt atcactatag gaatcaatat gattggctac

1561 gttcattgga gcaaagcaga tcaacgatgg aaaaatcagt ga

//

LOCUS Hco-des-2H;s4 1443 bp mRNA linear INV 05-MAR-2009

DEFINITION [gene=Hco-des-2H].

SOURCE Haemonchus contortus

ORGANISM Haemonchus contortus

Eukaryota; Metazoa; Nematoda; Chromadorea; Rhabditida; Strongylida;

Trichostrongyloidea; Haemonchidae; Haemonchinae; Haemonchus.

REFERENCE 1 (bases 1 to 1443)

AUTHORS Rufener,L., Maser,P., Roditi,I. and Kaminsky,R.

TITLE Haemonchus contortus Acetylcholine Receptors of the DEG-3 Subfamily

and their Role in Sensitivity to Monepantel

JOURNAL PLoS Pathogens, in press

REFERENCE 2 (bases 1 to 1443)

AUTHORS Rufener,L., Maser,P., Roditi,I. and Kaminsky,R.

TITLE Direct Submission

JOURNAL Submitted (05-MAR-2009) Institute of Cell Biology, University of

Bern, Baltzerstrasse 4, Bern, BE 3012, Switzerland

FEATURES Location/Qualifiers

source 1..1443

/organism="Haemonchus contortus"

/mol_type="mRNA"

/isolate="Hc-CRA AAD-mutant"

/clone="wt-s4"

/note="monepantel-insensitive isolate; wildtype gene,

short transcript"

CDS 1..1437

/note="Hco-DES-2H"

/codon_start=1

/product="H. contortus DES-2 homologue"

/translation="MYALTLTIAAALSAAMVASTPTQIQLVHDLLDKYDKKAKPMWDN

TKPINVSFTVSLYQILELNEPQQFVLLNAWIIERWYDEFLYWSPMEYQNITELRLPYD

SIWLPDTTLYNSLVMKDDDTRRLLNAKLTTDLQRRASLIELLYPTIYKFSCLLDLRFF

PFDVQNCTMIFSSWTYDQTGIDYFPASDEISIANYLENEGWELLKTEVSRHEVKYSCC

PNAYTLLHLTLYLRRKPLFYLVNLIIPTSIITLIAIVGFFTTSSASGMREEKVSLGIT

TLLSMSILMLMVSDQMPTTSTFIPLIGWFILAMIMVISLGTVVSSIIIAVQKWGSLGE

RLSKRTLRIAKMIAYFTCTALPSHIEKEQMMEAFDAVTPTGESVRTLKSAMDASKKWM

SLRRPKNGVAVVSDKSTDALIQMANSGAASIRHNRQLAVAEFEWLATVTERTCFVIFV

LCFLIITIGINMIGYVHWSKADQRWKNQ"

BASE COUNT 402 a 316 c 312 g 413 t

ORIGIN

1 atgtacgccc tcacgctcac cattgctgct gctctatctg cagctatggt tgcaagtaca

61 ccgactcaga ttcagctcgt tcacgatctg ctcgataagt atgacaaaaa ggcgaaacca

121 atgtgggaca atacgaaacc gatcaatgtc tcgttcaccg tatcactcta tcagattctt

181 gaactaaacg aaccacagca gttcgtactc cttaacgcct ggatcattga gagatggtat

241 gatgaatttt tgtactggtc ccctatggaa taccaaaata tcactgaatt acggctaccg

301 tatgattcaa tttggttgcc ggatactacg ctttacaatt cgttggtaat gaaggatgac

361 gatactagac gccttctgaa tgccaagcta acgacagatc ttcaacggcg agcatctctc

421 attgaacttc tttatcctac aatctacaaa ttctcttgtt tgcttgattt acgatttttc

481 ccatttgatg tgcagaattg tacaatgatt ttttcgtcgt ggacctatga tcagacgggg

541 atcgattact tcccagcatc agatgagata tcgattgcta actatttgga aaatgaagga

601 tgggaacttt tgaagactga agtgagtcgt catgaagtaa aatatagctg ctgcccgaat

661 gcctacacat tactgcattt aacgctttac ctgagaagaa aaccattgtt ctatttggtg

721 aatctgataa tacctacctc gatcatcaca cttattgcta ttgtgggatt cttcacgaca

781 tcctctgcca gtggaatgcg tgaagagaaa gtatcactag gaattactac gttgttatca

841 atgtcaatct taatgttaat ggtatctgat caaatgccta ccacgtcaac gttcatcccc

901 ctgattggat ggtttatact tgcaatgatc atggtaatct ccctcggaac agtggtctcg

961 tccattatta ttgcagttca aaaatggggc agtcttggag aaaggctttc gaaacggaca

1021 ttgaggatcg ccaaaatgat tgcatatttc acttgcactg ctctgccttc tcatattgaa

1081 aaggagcaga tgatggaagc attcgatgcc gtgacaccaa caggggaatc agtgaggacg

1141 ttaaagtcgg ccatggatgc gtcgaaaaag tggatgagcc ttcgacggcc caaaaacggc

1201 gtcgccgtcg tatccgacaa aagcaccgat gcccttattc agatggcgaa ctctggtgca

1261 gcgtccattc ggcataatcg gcagctggct gtggctgaat tcgaatggct ggctaccgtc

1321 acggaacgta cttgctttgt aatttttgta ctgtgcttcc tgattatcac cataggaatc

1381 aatatgattg gctacgttca ttggagcaaa gcagatcaac gatggaaaaa tcagtgatat

1441 gct

//

LOCUS Hco-des-2H;s5 1451 bp mRNA linear INV 05-MAR-2009

DEFINITION [gene=Hco-des-2H].

SOURCE Haemonchus contortus

ORGANISM Haemonchus contortus

Eukaryota; Metazoa; Nematoda; Chromadorea; Rhabditida; Strongylida;

Trichostrongyloidea; Haemonchidae; Haemonchinae; Haemonchus.

REFERENCE 1 (bases 1 to 1451)

AUTHORS Rufener,L., Maser,P., Roditi,I. and Kaminsky,R.

TITLE Haemonchus contortus Acetylcholine Receptors of the DEG-3 Subfamily

and their Role in Sensitivity to Monepantel

JOURNAL PLoS Pathogens, in press

REFERENCE 2 (bases 1 to 1451)

AUTHORS Rufener,L., Maser,P., Roditi,I. and Kaminsky,R.

TITLE Direct Submission

JOURNAL Submitted (05-MAR-2009) Institute of Cell Biology, University of

Bern, Baltzerstrasse 4, Bern, BE 3012, Switzerland

FEATURES Location/Qualifiers

source 1..1451

/organism="Haemonchus contortus"

/mol_type="mRNA"

/isolate="Hc-CRA AAD-mutant"

/clone="wt-s5"

/note="monepantel-insensitive isolate; wildtype gene,

short transcript"

CDS join(1..518,522..1437)

/note="Hco-DES-2H"

/codon_start=1

/product="H. contortus DES-2 homologue"

/translation="MYALTLTIAAALSAAMVASTPTQIQLVHDLLDKYDKKAKPMWDN

TKPINVSFTVSLYQILELNEPQQFVLLNAWIIERWYDEFLYWSPMEYQNITELRLPYD

SIWLPDTTLYNSLVMKDDDTRRLLNAKLTTDLQRRASLIELLYPTIYKFSCLLDLRFF

PFDVQNCTMIFSSTYDQTGIDYFPASDEISIANYLENEGWELLKTEVSRHEVKYSCCP

NAYTLLHLTLYLRRKPLFYLVNLIIPTSIITLIAIVGFFTTSSASGMREEKVSLGITT

LLSMSILMLMVSDQMPTTSTFIPLIGWFILAMIMVISLGTVVSSIIIAVQKRGSLGER

LSKRTLRIAKMIAYFTCTALPSHIEKEQMMEAFDAVTPTGESVRTLKSAMDASKKWMS

LRRPKNGVAVVSDKSTDALIQMANSGAASIRHNRQLAVAEFEWLATVTERTCFVIFVL

CFLIITIGINMIGYVHWSKADQRWKNQ"

BASE COUNT 404 a 321 c 314 g 412 t

ORIGIN

1 atgtacgccc tcacgctcac cattgctgct gctctatctg cagctatggt tgcaagtaca

61 ccgactcaga ttcagctcgt tcacgatctg ctcgataagt atgacaaaaa ggcgaaacca

121 atgtgggaca atacgaaacc gatcaatgtc tcgttcaccg tatcactcta tcagattctt

181 gaactaaacg aaccacagca gttcgtactc cttaacgcct ggatcattga gagatggtat

241 gatgaatttt tgtactggtc ccctatggaa taccaaaata tcactgaatt acggctaccg

301 tatgattcaa tttggttgcc ggatactacg ctttacaatt cgttggtaat gaaggatgac

361 gatactagac gccttctgaa tgccaagcta acgacagatc ttcaacggcg agcatctctc

421 attgaacttc tttatcctac aatctacaaa ttctcttgtt tgcttgattt acgatttttc

481 ccatttgatg tgcagaattg tacaatgatt ttttcgtcgt agacctatga tcagacgggg

541 atcgattact tcccagcatc agatgagata tcgattgcta actatttgga aaatgaagga

601 tgggaacttt tgaagactga agtgagtcgt catgaagtaa aatatagctg ctgcccgaat

661 gcctacacat tactgcattt aacgctttac ctgagaagaa aaccattgtt ctatttggtg

721 aatctgataa tacctacctc gatcatcaca cttattgcta ttgtgggatt cttcacgaca

781 tcctctgcca gtggaatgcg tgaagagaaa gtatcactag gaattactac gttgttatca

841 atgtcaatct taatgttaat ggtatctgat caaatgccta ccacgtcaac gttcatcccc

901 ctgattggat ggtttatact tgcaatgatc atggtaatct ccctcggaac agtggtctcg

961 tccattatta ttgcagttca aaaacggggc agtcttggag aaaggctttc gaaacggaca

1021 ttgaggatcg ccaaaatgat tgcatatttc acttgcactg ctctgccttc tcatattgaa

1081 aaggagcaga tgatggaagc attcgatgcc gtgacaccaa caggggaatc agtgaggacg

1141 ttaaagtcgg ccatggatgc gtcgaaaaag tggatgagcc ttcgacggcc caaaaacggc

1201 gtcgccgtcg tatccgacaa aagcaccgat gcccttattc agatggcgaa ctctggtgca

1261 gcgtccattc ggcataatcg gcagctggct gtggctgaat tcgaatggct ggctaccgtc

1321 acggaacgta cttgctttgt aatttttgta ctgtgcttcc tgattatcac cataggaatc

1381 aatatgattg gctacgttca ttggagcaaa gcagatcaac gatggaaaaa tcagtgatat

1441 gctcgagcgc c

//

LOCUS Hco-des-2H;s6 1451 bp mRNA linear INV 05-MAR-2009

DEFINITION [gene=Hco-des-2H].

SOURCE Haemonchus contortus

ORGANISM Haemonchus contortus

Eukaryota; Metazoa; Nematoda; Chromadorea; Rhabditida; Strongylida;

Trichostrongyloidea; Haemonchidae; Haemonchinae; Haemonchus.

REFERENCE 1 (bases 1 to 1451)

AUTHORS Rufener,L., Maser,P., Roditi,I. and Kaminsky,R.

TITLE Haemonchus contortus Acetylcholine Receptors of the DEG-3 Subfamily

and their Role in Sensitivity to Monepantel

JOURNAL PLoS Pathogens, in press

REFERENCE 2 (bases 1 to 1451)

AUTHORS Rufener,L., Maser,P., Roditi,I. and Kaminsky,R.

TITLE Direct Submission

JOURNAL Submitted (05-MAR-2009) Institute of Cell Biology, University of

Bern, Baltzerstrasse 4, Bern, BE 3012, Switzerland

FEATURES Location/Qualifiers

source 1..1451

/organism="Haemonchus contortus"

/mol_type="mRNA"

/isolate="Hc-CRA AAD-mutant"

/clone="wt-s6"

/note="monepantel-insensitive isolate; wildtype gene,

short transcript"

CDS 1..1437

/note="Hco-DES-2H"

/codon_start=1

/product="H. contortus DES-2 homologue"

/translation="MYALTLTIAAALSAAMVASTPTQIQLVHDLLDKYDKKAKPMWDN

TKPVNVSFTVSLYQILELNEPQQFVLLNAWIIERWYDEFLYWSPMEYQNITELRLPYD

SIWLPDTTLYNSLVMKDDDTRRLLNAKLTTDLQRRASLIELLYPTIYKFSCLLDLRFF

PFDVQNCTMIFSSWTYDQTGIDYFPASDEISIANYLENEGWELLKTEVSRHEVKYSCC

PNAYTLLHLTLYLRRKPLFYLVNLIIPTSIITLIAIVGFFTTSSASGMREEKVSLGIT

TLLSMSILMLMVSDQMPTTSTFIPLIGWFILAMIMVISLGTVVSSIIIAVQKRGSLGE

RLSKRTLRIAKMIAYFTCTALPSHIEKEQMMEAFDAVTPTGESVRTLKSAMDASKKWM

SLRRPKNGVAVVSDKSTDALIQMANSGAASIRHNRQLAVAEFEWLATVTERTCFVIFV

LCFLIITIGINMIGYVHWSKADQRWKNQ"

BASE COUNT 402 a 321 c 316 g 412 t

ORIGIN

1 atgtacgccc tcacgctcac cattgctgct gctctatctg cagctatggt tgcaagtaca

61 ccgactcaga ttcagctcgt tcacgatctg ctcgataagt atgacaaaaa ggcgaaacca

121 atgtgggaca atacgaaacc ggtcaatgtc tcgttcaccg tatcactcta tcagattctt

181 gaactaaacg aaccacagca gttcgtactc cttaacgcct ggatcattga gagatggtat

241 gatgaatttt tgtactggtc ccctatggaa taccaaaata tcactgaatt acggctaccg

301 tatgattcaa tttggttgcc ggatactacg ctttacaatt cgttggtaat gaaggatgac

361 gatactagac gccttctgaa tgccaagcta acgacagatc ttcaacggcg agcatctctc

421 attgaacttc tttatcctac aatctacaaa ttctcttgtt tgcttgattt acgatttttc

481 ccatttgatg tgcagaattg tacaatgatt ttttcgtcgt ggacctatga tcagacgggg

541 atcgattact tcccagcatc agatgagata tcgattgcta actatttgga aaatgaagga

601 tgggaacttt tgaagactga agtgagtcgt catgaagtaa aatatagctg ctgcccgaat

661 gcctacacat tactgcattt aacgctttac ctgagaagaa aaccattgtt ctatttggtg

721 aatctgataa tacctacctc gatcatcaca cttattgcta ttgtgggatt cttcacgaca

781 tcctctgcca gtggaatgcg tgaagagaaa gtatcactag gaattactac gttgttatca

841 atgtcaatct taatgttaat ggtatctgat caaatgccta ccacgtcaac gttcatcccc

901 ctgattggat ggtttatact tgcaatgatc atggtaatct ccctcggaac agtggtctcg

961 tccattatta ttgcagttca aaaacggggc agtcttggag aaaggctttc gaaacggaca

1021 ttgaggatcg ccaaaatgat tgcatatttc acttgcactg ctctgccttc tcatattgaa

1081 aaggagcaga tgatggaagc attcgatgcc gtgacaccaa caggggaatc agtgaggacg

1141 ttaaagtcgg ccatggatgc gtcgaaaaag tggatgagcc ttcgacggcc caaaaacggc

1201 gtcgccgtcg tatccgacaa aagcaccgat gcccttattc agatggcgaa ctctggtgca

1261 gcgtccattc ggcataatcg gcagctggct gtggctgaat tcgaatggct ggctaccgtc

1321 acggaacgta cttgctttgt aatttttgta ctgtgcttcc tgattatcac cataggaatc

1381 aatatgattg gctacgttca ttggagcaaa gcagatcaac gatggaaaaa tcagtgatat

1441 gctcgagcgc c

//

LOCUS Hco-des-2H;12 1605 bp mRNA linear INV 05-MAR-2009

DEFINITION [gene=Hco-des-2H].

SOURCE Haemonchus contortus

ORGANISM Haemonchus contortus

Eukaryota; Metazoa; Nematoda; Chromadorea; Rhabditida; Strongylida;

Trichostrongyloidea; Haemonchidae; Haemonchinae; Haemonchus.

REFERENCE 1 (bases 1 to 1605)

AUTHORS Rufener,L., Maser,P., Roditi,I. and Kaminsky,R.

TITLE Haemonchus contortus Acetylcholine Receptors of the DEG-3 Subfamily

and their Role in Sensitivity to Monepantel

JOURNAL PLoS Pathogens, in press

REFERENCE 2 (bases 1 to 1605)

AUTHORS Rufener,L., Maser,P., Roditi,I. and Kaminsky,R.

TITLE Direct Submission

JOURNAL Submitted (05-MAR-2009) Institute of Cell Biology, University of

Bern, Baltzerstrasse 4, Bern, BE 3012, Switzerland

FEATURES Location/Qualifiers

source 1..1605

/organism="Haemonchus contortus"

/mol_type="mRNA"

/isolate="Hc-Howick"

/clone="wt-12"

/note="monepantel-sensitive isolate; wildtype gene, long

transcript"

CDS 1..1605

/note="Hco-DES-2H"

/codon_start=1

/product="H. contortus DES-2 homologue"

/translation="MYALTLTIAAALSAAMVASTPTQIQLVHDLLDKYDKKAKPMWDN

TKPINVSFTVSLYQILELNEPQQFVLLNAWIIERWYDEFLYWSPMEYQNITELRLPYD

SIWLPDTTLYNSLVMKDDDTRRLLNAKLTTDLQRRASLIELLYPTIYKFSCLLDLRFF

PFDVQNCTMIFSSWTYDQTGIDYFPASDEISIANYLENEGWELLKTEVSRHEVKYSCC

PNAYTLLHLTLYLRRKPLFYLVNLIIPTSIITLIAIVGFFTTSSASGMREEKVSLGIT

TLLSMSILMLMVSDQMPTTSTFIPLIGWFILAMIMVISLGTVVSSIIIAVQKRGSLGE

RLSKRTLRIAKMIAYFTCTALPSHIEKEQMMEAFDAVTPTGESVRTLKSAMDASKKWM

SLRRPKNGVAVVSDKSTDALIQMANSGAEDGQMTGMTPVAPLVPPAAIIDDDLSLRSD

LSALPPSARLLKTKSSTRCNVFKDLTTSIRHNRQLAVAEFEWLATVTERTCFVIFVLC

FLIITIGINMIGYVHWSKADQRWKNQ"

BASE COUNT 453 a 357 c 340 g 455 t

ORIGIN

1 atgtacgccc tcacactcac cattgctgct gctctatctg cagccatggt tgcaagtacg

61 ccgactcaga ttcagctcgt tcacgatctg ctcgataaat atgacaaaaa ggcgaaacca

121 atgtgggaca atacgaaacc gatcaatgtc tcgttcaccg tatcacttta ccagattctt

181 gaactaaacg aaccacagca gttcgtactc ctcaacgcct ggatcattga gagatggtat

241 gatgaatttt tgtactggtc ccctatggag taccaaaata tcactgaatt acggctaccg

301 tatgattcaa tttggttgcc ggatactacg ctttacaatt cgttggtaat gaaggatgac

361 gatactagac gccttctgaa tgccaagcta acgacagacc ttcaacggcg agcatctctt

421 attgaacttc tttatcctac aatctacaaa ttctcttgtt tgcttgattt acgatttttc

481 ccatttgatg tgcagaattg tacaatgatt ttttcgtcat ggacctatga tcagacgggg

541 atcgattact tcccagcatc agatgagata tcgattgcta attatttgga aaatgaagga

601 tgggaacttt tgaagactga agtgagccgt catgaagtta agtacagctg ctgcccgaat

661 gcctacacat tactacattt aacactttac ctgagaagaa aaccattgtt ctatttggtg

721 aatctgataa tacctacctc tatcatcaca cttattgcta ttgtgggatt cttcacgact

781 tcttctgcca gtggaatgcg tgaagagaaa gtatcattag ggattactac gttgttatca

841 atgtcaatct taatgttaat ggtatctgat caaatgccta ccacgtcaac gttcatcccc

901 ctgattggat ggttcatact tgcaatgatc atggtaatct ccctcggaac agttgtctcg

961 tccattatta ttgctgttca aaaacgaggc agccttggag aaaggctttc aaaacggacg

1021 ttaaggatcg ccaaaatgat tgcatatttc acttgcactg ctctgccttc tcatattgaa

1081 aaggagcaaa tgatggaagc attcgatgcc gtgacaccaa caggggaatc tgtgaggacg

1141 ttaaagtcgg ccatggatgc atcaaaaaag tggatgagcc ttcgacggcc caaaaacggc

1201 gtcgccgtcg tatccgacaa aagcaccgat gcccttattc agatggcgaa ctctggtgca

1261 gaagatggac aaatgacggg tatgacaccg gtggcacctc tggtaccacc tgcagctatc

1321 atcgacgatg atctttcgtt acgaagtgat ttatcagctt taccaccatc agcacggctg

1381 ctcaaaacga aatccagtac cagatgtaat gtattcaagg atttaaccac gtccattcgg

1441 cataatcggc agttggctgt ggctgaattc gaatggctgg ctaccgtcac ggaacggact

1501 tgttttgtaa tttttgtgct gtgcttcctg attatcacta taggaatcaa tatgattggc

1561 tacgttcatt ggagcaaagc agatcaacga tggaaaaatc agtga

//

LOCUS Hco-des-2H;13 1605 bp mRNA linear INV 05-MAR-2009

DEFINITION [gene=Hco-des-2H].

SOURCE Haemonchus contortus

ORGANISM Haemonchus contortus

Eukaryota; Metazoa; Nematoda; Chromadorea; Rhabditida; Strongylida;

Trichostrongyloidea; Haemonchidae; Haemonchinae; Haemonchus.

REFERENCE 1 (bases 1 to 1605)

AUTHORS Rufener,L., Maser,P., Roditi,I. and Kaminsky,R.

TITLE Haemonchus contortus Acetylcholine Receptors of the DEG-3 Subfamily

and their Role in Sensitivity to Monepantel

JOURNAL PLoS Pathogens, in press

REFERENCE 2 (bases 1 to 1605)

AUTHORS Rufener,L., Maser,P., Roditi,I. and Kaminsky,R.

TITLE Direct Submission

JOURNAL Submitted (05-MAR-2009) Institute of Cell Biology, University of

Bern, Baltzerstrasse 4, Bern, BE 3012, Switzerland

FEATURES Location/Qualifiers

source 1..1605

/organism="Haemonchus contortus"

/mol_type="mRNA"

/isolate="Hc-Howick"

/clone="wt-13"

/note="monepantel-sensitive isolate; wildtype gene, long

transcript"

CDS 1..1605

/note="Hco-DES-2H"

/codon_start=1

/product="H. contortus DES-2 homologue"

/translation="MYALTLTIAAALSAAMVASTPTQIQLVHDLLDKYDKKAKPMWDN

TKPINVSFTVSLYQILELNEPQQFVLLNAWIIERWYDEFLYWSPMEYQNITELRLPYD

SIWLPDTTLYNSLVMKDDDTRRLLNAKLTTDLQRRASLIELLYPTIYKFSCLLDLRFF

PFDVQNCTMIFSSWTYDQTGIDYFPASDEISIANYLENEGWELLKTEVSRHEVKYSCC

PNAYTLLHLTLYLRRKPLFYLVNLIIPTSIITLIAIVGFFTTSSASGMREEKVSLGIT

TLLSMSILMLMVSDQMPTTSTFIPLIGWFILAMIMVISLGTVVSSIIIAVQKRGSLGE

RLSKRTLRIAKMIAYFTCTALPSHIEKEQMMEAFDAVTPTGESVRTLKSAMDASKKWM

SLRRPKNGVAVVSDKSTDALIQMANSGAEDGQMTGMTPAAPLVPPAAIIDDDLSLRSD

LSALPPSARLLKTKSSTRCNVFKDLTTSIRHNRQLAVAEFEWLATVTERTCFVIFVLC

FLIITIGINMIGYVHWSKADQRWKNQ"

BASE COUNT 453 a 355 c 338 g 459 t

ORIGIN

1 atgtacgccc tcacactcac cattgctgct gctctatctg cagccatggt tgcaagtaca

61 ccgactcaga tccagctcgt tcacgatctg ctcgataagt atgacaaaaa ggcgaaacca

121 atgtgggaca atacgaaacc gatcaatgtc tcgttcaccg tatcacttta ccagattctt

181 gaactaaacg aaccgcagca gttcgtactt ctcaacgcct ggatcattga gagatggtat

241 gatgaatttt tgtactggtc ccctatggaa taccaaaata tcactgaatt acggctaccg

301 tatgattcaa tttggttacc ggatactacg ctttacaatt cgttggtaat gaaggatgac

361 gatactagac gccttctgaa tgccaagcta acgacagatc ttcaacggcg agcatctctt

421 attgaacttc tttatcctac aatctacaaa ttctcttgtt tgcttgattt acgatttttc

481 ccatttgatg tgcagaattg tacaatgatt ttttcgtcat ggacctatga tcagacgggg

541 atcgattact tcccagcatc agatgagata tcgattgcta attatttgga aaatgaagga

601 tgggaacttt tgaagactga agtgagccgt catgaagtaa aatacagctg ctgccctaac

661 gcctacacat tactacattt aaccctttat ttgagaagga agccattatt ctatttggtt

721 aatctgataa tacctacctc tatcatcaca cttattgcta ttgtgggatt cttcacgact

781 tcttctgcca gtggaatgcg tgaagagaaa gtatcattag ggattactac gttgttatca

841 atgtcaatct tgatgttaat ggtatctgat caaatgccta ccacgtcaac gttcatccct

901 ttgattggat ggttcatact tgcaatgatc atggtaatct ccctcggaac agttgtctcg

961 tccattatta ttgctgttca aaaacgaggc agccttggag aaaggctttc aaaacggacg

1021 ttaaggatcg ccaaaatgat tgcatatttc acttgcactg ctctgccttc tcatattgaa

1081 aaggagcaaa tgatggaagc attcgatgcc gtgacaccaa caggggaatc tgtgaggacg

1141 ttaaagtcgg ccatggatgc atcaaaaaag tggatgagcc ttcgacggcc caaaaacggc

1201 gtcgccgtcg tatccgacaa aagcaccgat gcccttattc agatggcgaa ctctggtgca

1261 gaagatggac aaatgacggg tatgacaccg gcggcacctc tggtaccacc tgcagctatc

1321 atcgacgatg atctttcgtt acgaagtgat ttatcagctt taccaccatc agcacggctg

1381 ctcaaaacga aatccagtac cagatgtaat gtattcaagg atttaaccac gtccattcgg

1441 cataatcggc agttggctgt ggctgaattc gaatggctgg ctaccgtcac ggaacggact

1501 tgttttgtaa tttttgtgct gtgcttcctg attatcacta taggaatcaa tatgattggc

1561 tacgttcatt ggagcaaagc agatcaacga tggaaaaatc agtga

//

LOCUS Hco-des-2H;14 1602 bp mRNA linear INV 05-MAR-2009

DEFINITION [gene=Hco-des-2H].

SOURCE Haemonchus contortus

ORGANISM Haemonchus contortus

Eukaryota; Metazoa; Nematoda; Chromadorea; Rhabditida; Strongylida;

Trichostrongyloidea; Haemonchidae; Haemonchinae; Haemonchus.

REFERENCE 1 (bases 1 to 1602)

AUTHORS Rufener,L., Maser,P., Roditi,I. and Kaminsky,R.

TITLE Haemonchus contortus Acetylcholine Receptors of the DEG-3 Subfamily

and their Role in Sensitivity to Monepantel

JOURNAL PLoS Pathogens, in press

REFERENCE 2 (bases 1 to 1602)

AUTHORS Rufener,L., Maser,P., Roditi,I. and Kaminsky,R.

TITLE Direct Submission

JOURNAL Submitted (05-MAR-2009) Institute of Cell Biology, University of

Bern, Baltzerstrasse 4, Bern, BE 3012, Switzerland

FEATURES Location/Qualifiers

source 1..1602

/organism="Haemonchus contortus"

/mol_type="mRNA"

/isolate="Hc-Howick AAD-mutant"

/clone="wt-14"

/note="monepantel-insensitive isolate; wildtype gene, long

transcript"

CDS 1..1602

/note="Hco-DES-2H"

/codon_start=1

/product="H. contortus DES-2 homologue"

/translation="MYALTLTIAAALSAAMVASTPTQIQLVHDLLDKYDKKAKPMWDN

TKPINVSFTVSLYQILELNEPQQFVLLNAWIIERWYDEFLYWSPMEYQNITELRLPYD

SIWLPDTTLYNSLVMKDDDTRRLLNAKLTTDLQRRASLIELLYPTIYKFSCLLDLRFF

PFDVQNCTMIFSSWTYDQTGIDYFPASDEISIANYLENEGWELLKTEVSRHEVKYSCC

PNAYTLLHLTLYLRRKPLFYLVNLIIPTSIITLIAIVGLFTTSSASGMREEKVSLGIT

TLLSMSLMLMVSDQMPTTSTFIPLIGWFILAMIMVISLGTVVSSIIIAVQKRGSLGER

LSKRTLRIAKMIAYFTCTALPSHIEKEQMMEAFDAATPTGESVRTLKSAMDASKKWMS

LRRPKNGVAVVSDKSTDALIQMANSGAEDGQMTGMTPVAPLVPPAAIIDDDLSLRSDL

SALPPSARLLKTKSSTRCNVFKDLTTSIRHNRQLAVAEFEWLATVTERTCFVIFVLCF

LIITIGINMIGYVHWSKADQRWKNQ"

BASE COUNT 452 a 358 c 340 g 452 t

ORIGIN

1 atgtacgccc tcacactcac cattgctgct gctctatctg cagccatggt tgcaagtacg

61 ccgactcaga ttcagctcgt tcacgatctg ctcgataaat atgacaaaaa ggcgaaacca

121 atgtgggaca atacgaaacc gatcaatgtc tcgttcaccg tatcacttta ccagattctt

181 gaactaaacg aaccacagca gttcgtactc ctcaacgcct ggatcattga gagatggtat

241 gatgaatttt tgtactggtc ccctatggag taccaaaata tcactgaatt acggctaccg

301 tatgattcaa tttggttgcc ggatactacg ctttacaatt cgttggtaat gaaggatgac

361 gatactagac gccttctgaa tgccaagcta acgacagacc ttcaacggcg agcatctctt

421 attgaacttc tttatcctac aatctacaaa ttctcttgtt tgcttgattt acgatttttc

481 ccatttgatg tgcagaattg tacaatgatt ttttcgtcat ggacctatga tcagacgggg

541 atcgattact tcccagcatc agatgagata tcgattgcta attatttgga aaatgaagga

601 tgggaacttt tgaagactga agtgagccgt catgaagtta agtacagctg ctgcccgaat

661 gcctacacat tactacattt aacactttac ctgagaagaa aaccattgtt ctatttggtg

721 aatctgataa tacctacctc tatcatcaca cttattgcta ttgtgggact cttcacgact

781 tcttctgcca gtggaatgcg tgaagagaaa gtatcattag ggattactac gttgttatca

841 atgtcattaa tgttaatggt atctgatcaa atgcctacca cgtcaacgtt catccccctg

901 attggatggt tcatacttgc aatgatcatg gtaatctccc tcggaacagt tgtctcgtcc

961 attattattg ctgttcaaaa acgaggcagc cttggagaaa ggctttcaaa acggacgtta

1021 aggatcgcca aaatgattgc atatttcact tgcactgctc tgccttctca tattgaaaag

1081 gagcaaatga tggaagcatt cgatgccgcg acaccaacag gggaatctgt gaggacgtta

1141 aagtcggcca tggatgcatc aaaaaagtgg atgagccttc gacggcccaa aaacggcgtc

1201 gccgtcgtat ccgacaaaag caccgatgcc cttattcaga tggcgaactc tggtgcagaa

1261 gatggacaaa tgacgggtat gacaccggtg gcacctctgg taccacctgc agctatcatc

1321 gacgatgatc tttcgttacg aagtgattta tcagctttac caccatcagc acggctgctc

1381 aaaacgaaat ccagtaccag atgtaatgta ttcaaggatt taaccacgtc cattcggcat

1441 aatcggcagt tggctgtggc tgaattcgaa tggctggcta ccgtcacgga acggacttgt

1501 tttgtaattt ttgtgctgtg cttcctgatt atcactatag gaatcaatat gattggctac

1561 gttcattgga gcaaagcaga tcaacgatgg aaaaatcagt ga

//

LOCUS Hco-des-2H;15 1605 bp mRNA linear INV 05-MAR-2009

DEFINITION [gene=Hco-des-2H].

SOURCE Haemonchus contortus

ORGANISM Haemonchus contortus

Eukaryota; Metazoa; Nematoda; Chromadorea; Rhabditida; Strongylida;

Trichostrongyloidea; Haemonchidae; Haemonchinae; Haemonchus.

REFERENCE 1 (bases 1 to 1605)

AUTHORS Rufener,L., Maser,P., Roditi,I. and Kaminsky,R.

TITLE Haemonchus contortus Acetylcholine Receptors of the DEG-3 Subfamily

and their Role in Sensitivity to Monepantel

JOURNAL PLoS Pathogens, in press

REFERENCE 2 (bases 1 to 1605)

AUTHORS Rufener,L., Maser,P., Roditi,I. and Kaminsky,R.

TITLE Direct Submission

JOURNAL Submitted (05-MAR-2009) Institute of Cell Biology, University of

Bern, Baltzerstrasse 4, Bern, BE 3012, Switzerland

FEATURES Location/Qualifiers

source 1..1605

/organism="Haemonchus contortus"

/mol_type="mRNA"

/isolate="Hc-Howick AAD-mutant"

/clone="wt-15"

/note="monepantel-insensitive isolate; wildtype gene, long

transcript"

CDS 1..1605

/note="Hco-DES-2H"

/codon_start=1

/product="H. contortus DES-2 homologue"

/translation="MYALTLTIAAALSAAMVASTPTQIQLVHDLLDKYDKKAKPMWDN

TKPINVSFTVSLYQILELNEPQQFVLLNAWIIERWYDEFLYWSPMEYQNITELRLPYD

SIWLPDTTLYNSLVMKDDDTRRLLNAKLTTDLQRRASLIELLYPTIYKFSCLLDLRFF

PFDVQNCTMIFSSWTYDQTGIDYFPASDEISIANYLENEGWELLKTEVSRHEVKYSCC

PNAYTLLHLTLYLRRKPLFYLVNLIIPTSIITLIAIVGFFTTSSASGMREEKVSLGIT

TLLSMSILMLMVSDQMPTTSTFIPLIGWFILAMIMVISLGTVVSSIIIAVQKRGSLGE

RLSKRTLRIAKMTAYFTCTALPSHIEKEQMMEAFDAVTPTGESVRTLKSAMDASKKWM

SLRRPKNGVAVVSDKSTDALIQMANSGAEDGQMTGMTPVAPLGPPTAIIDDDLSFRSD

FSALPPSARLLKTKSSTRCNVFKDLTTSIRHNRQLAVAEFEWLATVTERTCFVIFVLC

FLIITIGINMIGYVHWSKADQRWKNQ"

BASE COUNT 450 a 359 c 346 g 450 t

ORIGIN

1 atgtacgccc tcacgctcac cattgctgct gctctatctg cagctatggt tgcaagtaca

61 ccgactcaga ttcagctcgt tcacgatctg ctcgataagt atgacaaaaa ggcgaaacca

121 atgtgggaca atacgaaacc gatcaatgtc tcgttcaccg tatcactcta tcagattctt

181 gaactaaacg aaccacagca gttcgtactc cttaacgcct ggatcattga gagatggtat

241 gatgaatttt tgtactggtc ccctatggaa taccaaaata tcactgaatt acggctaccg

301 tatgattcaa tttggttgcc ggatactacg ctttacaatt cgttggtaat gaaggatgac

361 gatactagac gccttctgaa tgccaagcta acgacagatc ttcaacggcg agcatctctc

421 attgaacttc tttatcctac aatctacaaa ttctcttgtt tgcttgattt acgatttttc

481 ccatttgatg tgcagaattg tacaatgatt ttttcgtcgt ggacctatga tcagacgggg

541 atcgattact tcccagcatc agatgagata tcgattgcta actatttgga aaatgaagga

601 tgggaacttt tgaagactga agtgagtcgt catgaagtaa aatatagctg ctgcccgaat

661 gcctacacat tactgcattt aacgctttac ctgagaagaa aaccattgtt ctatttggtg

721 aatctgataa tacctacctc gatcatcaca cttattgcta ttgtgggatt cttcacgaca

781 tcctctgcca gtggaatgcg tgaagagaaa gtatcactag gaattactac gttgttatca

841 atgtcaatct taatgttaat ggtatctgat caaatgccta ccacgtcaac gttcatcccc

901 ctgattggat ggtttatact tgcaatgatc atggtaatct ccctcggaac agtggtctcg

961 tccattatta ttgcagttca aaaacggggc agtcttggag aaaggctttc gaaacggaca

1021 ttgaggatcg ccaaaatgac tgcatatttc acttgcactg ctctgccttc tcatattgaa

1081 aaggagcaga tgatggaagc attcgatgcc gtgacaccaa caggggaatc agtgaggacg

1141 ttaaagtcgg ccatggatgc gtcgaaaaag tggatgagcc ttcgacggcc caaaaacggc

1201 gtcgccgtcg tatccgacaa aagcaccgat gcccttattc agatggcgaa ctctggtgca

1261 gaagatggcc aaatgacggg tatgacaccg gtggcacctc tgggaccacc tacagctatc

1321 atcgacgatg atctttcgtt tcgaagtgat ttctcagctt taccaccatc agcacggctg

1381 ctcaaaacga aatccagtac cagatgtaat gtattcaagg atttaactac gtccattcgg

1441 cataatcggc agctggctgt ggctgaattc gaatggctgg ctaccgtcac ggaacgtact

1501 tgctttgtaa tttttgtact gtgcttcctg attatcacca taggaatcaa tatgattggc

1561 tacgttcatt ggagcaaagc agatcaacga tggaaaaatc agtga

//

LOCUS Hco-des-2H;16 954 bp mRNA linear INV 05-MAR-2009

DEFINITION [gene=Hco-des-2H].

SOURCE Haemonchus contortus

ORGANISM Haemonchus contortus

Eukaryota; Metazoa; Nematoda; Chromadorea; Rhabditida; Strongylida;

Trichostrongyloidea; Haemonchidae; Haemonchinae; Haemonchus.

REFERENCE 1 (bases 1 to 954)

AUTHORS Rufener,L., Maser,P., Roditi,I. and Kaminsky,R.

TITLE Haemonchus contortus Acetylcholine Receptors of the DEG-3 Subfamily

and their Role in Sensitivity to Monepantel

JOURNAL PLoS Pathogens, in press

REFERENCE 2 (bases 1 to 954)

AUTHORS Rufener,L., Maser,P., Roditi,I. and Kaminsky,R.

TITLE Direct Submission

JOURNAL Submitted (05-MAR-2009) Institute of Cell Biology, University of

Bern, Baltzerstrasse 4, Bern, BE 3012, Switzerland

FEATURES Location/Qualifiers

source 1..954

/organism="Haemonchus contortus"

/mol_type="mRNA"

/isolate="Hc-CRA"

/clone="wt-16"

/note="monepantel-sensitive isolate; wildtype gene"

CDS 147..>953

/note="Hco-DES-2H"

/codon_start=1

/product="H. contortus DES-2 homologue"

/translation="MYALTLTIAAALSAAMVASTPTQIQLVHDLLDKYDKKAKPMWDN

TKPINVSFTVSLYQILELNEPQQFVLLNAWIIERWYDEFLYWSPMEYQNITELRLPYD

SIWLPDTTLYNSLVMKDDDTRRLLNAKLTTDLQRRASLIELLYPTIYKFSCLLDLRFF

PFDVQNCTMIFSSWTYDQTGIDYFPASDEISIANYLENEGWELLKTEVSRHEVKYSCC

PNAYTLLHLTLYLRRKPLFYLVKLIIPTSIITLIAIVGFFTTSSASGMREE"

BASE COUNT 262 a 232 c 184 g 276 t

ORIGIN

1 ggtttaatta cccaagtttg aggatacgct tgacagcatc gtggtctaac accattgccg

61 ttcaatctcg agattgaacc acctaacaga gtcgccacag ccatcgttgt cgctgccgtc

121 accgctcctc ccctacctac tattcaatgt acgccctcac actcaccatt gctgctgctc

181 tatctgcagc catggttgca agtacaccga ctcagattca gctcgttcac gatctgctcg

241 ataaatatga caagaaagca aaaccgatgt gggacaatac gaaaccgatc aatgtctcgt

301 tcaccgtatc actttaccag attcttgaac taaacgaacc acagcagttc gtactcctca

361 acgcctggat cattgagaga tggtatgatg aatttttgta ctggtcccct atggaatacc

421 aaaatatcac cgaattacgg ctaccgtatg attcaatttg gttgccggat actacgcttt

481 acaattcgtt ggtaatgaag gatgacgata ctagacgcct tctgaatgcc aagctaacga

541 cagatcttca acggcgagca tctctcattg aacttcttta tcctacaatc tacaaattct

601 cttgtttgct tgatttacga tttttcccat tcgatgtgca gaattgtaca atgatttttt

661 cgtcatggac ctatgatcag acggggatcg attacttccc agcatcagat gagatatcga

721 ttgctaatta tttggaaaat gaaggatggg aacttttgaa gactgaagtg agccgtcatg

781 aagtaaaata cagctgctgc ccgaatgcct acacattact gcatttaact ctttacctga

841 gaaggaaacc attgttctat ttggtgaagc tgatcattcc tacctctatc atcacactta

901 ttgctattgt gggattcttc acgacatcct ctgccagtgg aatgcgtgaa gaga

//

LOCUS Hco-des-2H;17 963 bp mRNA linear INV 05-MAR-2009

DEFINITION [gene=Hco-des-2H].

SOURCE Haemonchus contortus

ORGANISM Haemonchus contortus

Eukaryota; Metazoa; Nematoda; Chromadorea; Rhabditida; Strongylida;

Trichostrongyloidea; Haemonchidae; Haemonchinae; Haemonchus.

REFERENCE 1 (bases 1 to 963)

AUTHORS Rufener,L., Maser,P., Roditi,I. and Kaminsky,R.

TITLE Haemonchus contortus Acetylcholine Receptors of the DEG-3 Subfamily

and their Role in Sensitivity to Monepantel

JOURNAL PLoS Pathogens, in press

REFERENCE 2 (bases 1 to 963)

AUTHORS Rufener,L., Maser,P., Roditi,I. and Kaminsky,R.

TITLE Direct Submission

JOURNAL Submitted (05-MAR-2009) Institute of Cell Biology, University of

Bern, Baltzerstrasse 4, Bern, BE 3012, Switzerland

FEATURES Location/Qualifiers

source 1..963

/organism="Haemonchus contortus"

/mol_type="mRNA"

/isolate="Hc-CRA"

/clone="wt-17"

/note="monepantel-sensitive isolate; wildtype gene"

CDS 156..>962

/note="Hco-DES-2H"

/codon_start=1

/product="H. contortus DES-2 homologue"

/translation="MYALTLTIAAALSAAMVASTPTQIQLVHDLLDKYGKKAKPMWDN

TKPINVSFTVSLYQILELNEPQQFVLLNAWIIERWYDEFLYWSPMEYQNITELRLPYD

SIWLPDTTLYNSLVMKDDDTRRLLNAKLTTDLQRRASLIELLYPTIYKFSCLLDLRFF

PFDVQNCTMIFSSWTYDQTGIDYFPASDEISIANYLGNEGWELLKTEVSRHEVKYSCC

PNAYTLLHLTLYLRRKPLFYLVNLIIPTSIITLIAIVGFFTTSSASGMREE"

BASE COUNT 266 a 232 c 181 g 284 t

ORIGIN

1 ggtttaatta cccaagtttg aggatacgct tgacagcatc gtggtctaac accattaccg

61 tccaatctcg agattgaacc acctaacaga gtcgtcaccg ccgaaaccgc catcgttgtc

121 gctgccgtca ccgctcctcc cctacctact attcaatgta cgccctcaca ctcaccattg

181 ctgctgctct atctgcagcc atggttgcaa gtacaccaac tcagatccag cttgttcacg

241 atctgcttga taaatacggc aagaaggcga aaccaatgtg ggacaatacg aaaccgatca

301 atgtctcgtt caccgtatca ctttaccaga ttcttgaact aaacgaacca cagcagttcg

361 tactcctcaa cgcctggatt attgagagat ggtatgatga atttttgtac tggtccccta

421 tggagtacca aaatatcact gaattacggc taccgtatga ttcaatttgg ttaccggata

481 ctacgcttta caattcgttg gtaatgaagg atgacgatac tagacgcctt ctgaatgcca

541 agctaacgac agatcttcaa cggcgagcat ctcttattga acttctttat cctacaatct

601 acaaattctc ttgtctactt gacctacgat ttttcccatt tgatgtacag aattgtacaa

661 tgattttttc gtcatggacc tatgatcaga cagggatcga ttactttcct gcttcagatg

721 aaatatcgat tgctaattat ttgggaaatg aaggatggga acttttgaag actgaagtga

781 gccgtcatga agtaaaatac agctgctgtc cgaatgccta cacattacta catttaacac

841 tttatttgag aaggaagcca ttattctatt tggtgaacct gatcattcct acctctatca

901 tcacacttat tgctattgtg ggattcttca cgacttcttc tgccagtgga atgcgtgaag

961 aga

//

LOCUS Hco-des-2H;18 954 bp mRNA linear INV 05-MAR-2009

DEFINITION [gene=Hco-des-2H].

SOURCE Haemonchus contortus

ORGANISM Haemonchus contortus

Eukaryota; Metazoa; Nematoda; Chromadorea; Rhabditida; Strongylida;

Trichostrongyloidea; Haemonchidae; Haemonchinae; Haemonchus.

REFERENCE 1 (bases 1 to 954)

AUTHORS Rufener,L., Maser,P., Roditi,I. and Kaminsky,R.

TITLE Haemonchus contortus Acetylcholine Receptors of the DEG-3 Subfamily

and their Role in Sensitivity to Monepantel

JOURNAL PLoS Pathogens, in press

REFERENCE 2 (bases 1 to 954)

AUTHORS Rufener,L., Maser,P., Roditi,I. and Kaminsky,R.

TITLE Direct Submission

JOURNAL Submitted (05-MAR-2009) Institute of Cell Biology, University of

Bern, Baltzerstrasse 4, Bern, BE 3012, Switzerland

FEATURES Location/Qualifiers

source 1..954

/organism="Haemonchus contortus"

/mol_type="mRNA"

/isolate="Hc-CRA"

/clone="wt-18"

/note="monepantel-sensitive isolate; wildtype gene"

CDS 147..>953

/note="Hco-DES-2H"

/codon_start=1

/product="H. contortus DES-2 homologue"

/translation="MYALTLTIAAALSAAMVASTPTQIQLVHDLLDKYDKKAKPMWDN

TKPINVSFTVSLYQILELNEPQQFVLLNAWIIERWYDEFLYWSPMEYQNITELRLPYD

SIWLPDTTLYNSLVMKDDDTRRLLNAKLTTDLQRRASLIELLYPTIHKFSCLLDLRFF

PFDVQNCTMIFSSWTYDQTRIDYFPASDEISIANYLENEGWELLKTEVSRHEVKYSCC

PNAYTLLHLTLYLRRKPLFYLVNLIIPTSIITLIAIVGFFTTSSASGMREE"

BASE COUNT 264 a 235 c 181 g 274 t

ORIGIN

1 ggtttaatta cccaagtttg aggatacgct tgacagcatc gtggtctaac accattgccg

61 tccaatctcg agattgaacc acctaacaga gtcgccacag ccatcgttgt cgctgccgtc

121 accgctcctc ccctacctac tattcaatgt acgccctcac actcaccatt gctgctgctc

181 tatctgcagc catggttgca agtacaccga ctcagattca gctcgttcac gatctgctcg

241 ataaatatga caagaaagca aaaccgatgt gggacaatac gaaaccgatc aatgtctcgt

301 tcaccgtatc actttaccag attcttgaac taaacgaacc acagcagttc gtactcctca

361 acgcctggat cattgagaga tggtatgatg aatttttgta ctggtcccct atggaatacc

421 aaaatatcac tgaattacgg ctaccgtatg attcaatttg gttgccggat actacgcttt

481 acaattcgtt ggtaatgaag gatgacgata ctagacgcct tctgaatgcc aagctaacga

541 cagatcttca acggcgagca tctctcattg aacttcttta tcctacaatc cacaaattct

601 cttgtttgct tgatctacga tttttcccat tcgatgtgca gaattgtaca atgatttttt

661 cgtcatggac ctatgatcag acgaggatcg attacttccc agcatcagat gagatatcga

721 ttgctaatta tttggaaaat gaaggatggg aacttttgaa gactgaagtg agccgtcatg

781 aagtaaaata cagctgctgc ccgaatgcct acacattact gcatttaact ctttacctga

841 gaagaaaacc attgttctat ttggtgaacc tgatcattcc tacctctatc atcacactta

901 ttgctattgt gggattcttc acgacatcct ctgccagtgg aatgcgtgaa gaga

//

LOCUS Hco-des-2H;19 954 bp mRNA linear INV 05-MAR-2009

DEFINITION [gene=Hco-des-2H].

SOURCE Haemonchus contortus

ORGANISM Haemonchus contortus

Eukaryota; Metazoa; Nematoda; Chromadorea; Rhabditida; Strongylida;

Trichostrongyloidea; Haemonchidae; Haemonchinae; Haemonchus.

REFERENCE 1 (bases 1 to 954)

AUTHORS Rufener,L., Maser,P., Roditi,I. and Kaminsky,R.

TITLE Haemonchus contortus Acetylcholine Receptors of the DEG-3 Subfamily

and their Role in Sensitivity to Monepantel

JOURNAL PLoS Pathogens, in press

REFERENCE 2 (bases 1 to 954)

AUTHORS Rufener,L., Maser,P., Roditi,I. and Kaminsky,R.

TITLE Direct Submission

JOURNAL Submitted (05-MAR-2009) Institute of Cell Biology, University of

Bern, Baltzerstrasse 4, Bern, BE 3012, Switzerland

FEATURES Location/Qualifiers

source 1..954

/organism="Haemonchus contortus"

/mol_type="mRNA"

/isolate="Hc-CRA"

/clone="wt-19"

/note="monepantel-sensitive isolate; wildtype gene"

CDS 147..>953

/note="Hco-DES-2H"

/codon_start=1

/product="H. contortus DES-2 homologue"

/translation="MYALTLTIAAALSAAMVASTPTQIQLVHDLLDKYDKEAKPMWDN

TKPINVSFTVSLYQILELNEPQQFVLLNAWIIERWYDEFLYWSPMEYQNITELRLPYD

SIWLPDTTLYNSLVMKDDDTRRLLNAKLTTDLQRRASLIELLYPTIYKFSCLLDLRFF

PFDVQNCTMIFSSWTYDQTGIDYFPASDEISIANYLENEGWELLKTEVSRHEVKYSCC

PNAYTLLHLTLYLRRKPLFYLVNLIIPTSIITLIAIVGFFTTSSASGMREE"

BASE COUNT 262 a 233 c 183 g 276 t

ORIGIN

1 ggtttaatta cccaagtttg aggatacgct tgacagcatc gtggtctaac accattgccg

61 tccaatctcg agattgaacc acctaacaga gtcgccacag ccatcgttgt cgctgccgtc

121 accgctcctc ccctacctac tattcaatgt acgccctcac actcaccatt gctgctgctc

181 tatctgcagc catggttgca agtacaccga ctcagattca gctcgttcac gatctgctcg

241 ataaatatga caaggaagca aaaccgatgt gggacaatac gaaaccgatc aatgtctcgt

301 tcaccgtatc actttaccag attcttgaac taaacgaacc acagcagttc gtactcctca

361 acgcctggat cattgagaga tggtatgatg aatttttgta ctggtcccct atggaatacc

421 aaaatatcac tgaattacgg ctaccgtatg attcaatttg gttgccggat actacgcttt

481 acaattcgtt ggtaatgaag gatgacgata ctagacgcct tctgaatgcc aagctaacga

541 cagatcttca acggcgagca tctctcattg aacttcttta tcctacaatc tacaaattct

601 cttgtttgct tgatttacga tttttcccat tcgatgtgca gaattgtaca atgatttttt

661 cgtcatggac ctatgatcag acggggatcg attacttccc agcatcagat gagatatcga

721 ttgctaatta tttggaaaat gaaggatggg aacttttgaa gactgaagtg agccgtcatg

781 aagtaaaata cagctgctgc ccgaatgcct acacattact gcatttaact ctttacctga

841 gaagaaaacc attgttctat ttggtgaacc tgatcattcc tacctctatc atcacactta

901 ttgctattgt gggattcttc acgacatcct ctgccagtgg aatgcgtgaa gaga

//

LOCUS Hco-des-2H;20 954 bp mRNA linear INV 05-MAR-2009

DEFINITION [gene=Hco-des-2H].

SOURCE Haemonchus contortus

ORGANISM Haemonchus contortus

Eukaryota; Metazoa; Nematoda; Chromadorea; Rhabditida; Strongylida;

Trichostrongyloidea; Haemonchidae; Haemonchinae; Haemonchus.

REFERENCE 1 (bases 1 to 954)

AUTHORS Rufener,L., Maser,P., Roditi,I. and Kaminsky,R.

TITLE Haemonchus contortus Acetylcholine Receptors of the DEG-3 Subfamily

and their Role in Sensitivity to Monepantel

JOURNAL PLoS Pathogens, in press

REFERENCE 2 (bases 1 to 954)

AUTHORS Rufener,L., Maser,P., Roditi,I. and Kaminsky,R.

TITLE Direct Submission

JOURNAL Submitted (05-MAR-2009) Institute of Cell Biology, University of

Bern, Baltzerstrasse 4, Bern, BE 3012, Switzerland

FEATURES Location/Qualifiers

source 1..954

/organism="Haemonchus contortus"

/mol_type="mRNA"

/isolate="Hc-CRA"

/clone="wt-20"

/note="monepantel-sensitive isolate; wildtype gene"

CDS 147..>953

/note="Hco-DES-2H"

/codon_start=1

/product="H. contortus DES-2 homologue"

/translation="MYALTLTIAAALSAAMVASTPTQIQLVHDLLDKYDEKAKPMWDN

TKPINVSFTVSLYQILELNEPQQFVLLNAWIIERWYDEFLYWSPMEYQNITELRLPYD

SIWLPDTTLYNSLVMKDDDTRRLLNAKLTTDLQRRASLIELLYPTIYKFSCLLDLRFF

PFDVQNCTMIFSSWTYDQTGIDYFPASDEISIANYLENEGWELLKTEVSRHEVKYSCC

PNAYTLLHLTLYLRRKPLFYLVNLIIPTSIITLIAIVGFFTTSSASGMREE"

BASE COUNT 262 a 230 c 181 g 281 t

ORIGIN

1 ggtttaatta cccaagtttg aggatacgcc tgacagcatc gtggtctaac accattgccg

61 tccaatctcg agattgaacc acctaacaga gtcgccacag ccatcgttgt cgctgccgtc

121 accgctcctc ccctacctac tattcaatgt acgccctcac actcaccatt gctgctgctc

181 tatctgcagc catggttgca agtacaccga ctcagattca gctcgttcac gatctgctcg

241 ataaatatga cgagaaggca aaaccgatgt gggacaatac gaaaccgatc aatgtctcgt

301 tcaccgtatc actttaccag attcttgaac taaacgaacc acagcagttc gtactcctca

361 acgcctggat tattgagaga tggtatgatg aatttttgta ctggtcccct atggagtacc

421 aaaatatcac tgaattacgg ctaccgtatg attcaatttg gttaccggat actacgcttt

481 acaattcgtt ggtaatgaag gatgacgata ctagacgcct tctgaatgcc aagctaacga

541 cagatcttca acggcgagca tctcttattg aacttcttta tcctacaatc tacaaattct

601 cttgtctact tgacctacga tttttcccat ttgatgtaca gaattgtaca atgatttttt

661 cgtcatggac ctatgatcag acagggatcg attactttcc tgcttcggat gaaatatcga

721 ttgctaatta tttggaaaat gaaggatggg aacttttgaa gactgaagtg agccgtcatg

781 aagtaaaata cagctgctgt ccgaatgcct acacattact acacttaaca ctttatttga

841 gaaggaagcc attattctat ttggtgaacc tgatcattcc tacctctatc atcacactta

901 ttgctattgt gggattcttc acgacttctt ctgccagtgg aatgcgtgaa gaga

//

LOCUS Hco-des-2H;m1-1 1098 bp mRNA linear INV 05-MAR-2009

DEFINITION [gene=Hco-des-2H].

SOURCE Haemonchus contortus

ORGANISM Haemonchus contortus

Eukaryota; Metazoa; Nematoda; Chromadorea; Rhabditida; Strongylida;

Trichostrongyloidea; Haemonchidae; Haemonchinae; Haemonchus.

REFERENCE 1 (bases 1 to 1098)

AUTHORS Rufener,L., Maser,P., Roditi,I. and Kaminsky,R.

TITLE Haemonchus contortus Acetylcholine Receptors of the DEG-3 Subfamily

and their Role in Sensitivity to Monepantel

JOURNAL PLoS Pathogens, in press

REFERENCE 2 (bases 1 to 1098)

AUTHORS Rufener,L., Maser,P., Roditi,I. and Kaminsky,R.

TITLE Direct Submission

JOURNAL Submitted (05-MAR-2009) Institute of Cell Biology, University of

Bern, Baltzerstrasse 4, Bern, BE 3012, Switzerland

FEATURES Location/Qualifiers

source 1..1098

/organism="Haemonchus contortus"

/mol_type="mRNA"

/isolate="Hc-CRA AAD-mutant"

/clone="m1-1"

/note="monepantel-insensitive isolate; mutant gene"

CDS 78..212

/note="Hco-DES-2H"

/codon_start=1

/product="H. contortus DES-2 homologue"

/translation="MTRVGRGTITGTPLAPDEQVLRAVCKWIRLTASWSNTITVQSRD"

BASE COUNT 302 a 269 c 211 g 316 t

ORIGIN

1 ggtttaatta cccaagtttg aggttccaat accggaaagc taaagcaaag tctcaactgc

61 gacgatttga tgagcttatg acacgtgttg gtcgcggtac catcaccggt actccactcg

121 ctcccgacga acaagttcta cgtgctgtct gcaagtggat acgcttgaca gcatcgtggt

181 ctaacaccat taccgtccaa tctcgagatt gaaccaccta acagagtcgt caccgccgaa

241 accgccatcg ttgtcgctgc cgtcaccgct cctcccctac ctactattca atgtacgccc

301 tcacactcac cattgctgct gctctatctg cagccatggt tgcaagtaca ccaactcaga

361 tccagcttgt tcacgatctg cttgataaat acgacaagaa ggcgaaacca atgtgggaca

421 atacgaaacc gatcaatgtc tcgttcaccg tatcacttta ccagattctt gaactaaacg

481 aaccacagca gttcgtactc ctcaacgcct ggattattga gagatggtat gatgaatttt

541 tgtactggtc ccctatggag taccaaaata tcactgaatt acggctaccg tatgattcaa

601 tttggttacc ggatactacg ctttacaatt cgttggtaat gaaggatgac gatactagac

661 gccttctgaa tgccaagcta acgacagatc ttcaacggcg agcatctctt attgaacttc

721 tttatcctac aatctacaaa ttctcttgtc tacttgacct acgatttttc ccatttgatg

781 tacagaattg tacaatgatt ttttcgtcat ggacctatga tcagacaggg atcgattact

841 ttcctgcttc agatgaaata tcgattgcta attatttgga aaatgaagga tgggaacttt

901 tgaagactga agtgagccgt catgaagtaa aatacagctg ctgtccgaat gcctacacat

961 tactacattt aacactttat ttgagaagga agccattatt ctatttggtg aacctgatca

1021 ttcctacctc tatcatcaca cttattgcta ttgtgggatt cttcacgact tcttctgcca

1081 gtggaatgcg tgaagaga

//

LOCUS Hco-des-2H;m1-2 1086 bp mRNA linear INV 05-MAR-2009

DEFINITION [gene=Hco-des-2H].

SOURCE Haemonchus contortus

ORGANISM Haemonchus contortus

Eukaryota; Metazoa; Nematoda; Chromadorea; Rhabditida; Strongylida;

Trichostrongyloidea; Haemonchidae; Haemonchinae; Haemonchus.

REFERENCE 1 (bases 1 to 1086)

AUTHORS Rufener,L., Maser,P., Roditi,I. and Kaminsky,R.

TITLE Haemonchus contortus Acetylcholine Receptors of the DEG-3 Subfamily

and their Role in Sensitivity to Monepantel

JOURNAL PLoS Pathogens, in press

REFERENCE 2 (bases 1 to 1086)

AUTHORS Rufener,L., Maser,P., Roditi,I. and Kaminsky,R.

TITLE Direct Submission

JOURNAL Submitted (05-MAR-2009) Institute of Cell Biology, University of

Bern, Baltzerstrasse 4, Bern, BE 3012, Switzerland

FEATURES Location/Qualifiers

source 1..1086

/organism="Haemonchus contortus"

/mol_type="mRNA"

/isolate="Hc-CRA AAD-mutant"

/clone="m1-2"

/note="monepantel-insensitive isolate; mutant gene"

CDS 78..212

/note="Hco-DES-2H"

/codon_start=1

/product="H. contortus DES-2 homologue"

/translation="MTRVGRGTITGTPLAPDEQVLRAVCKWIRLTASWSNTITVQSRD"

BASE COUNT 299 a 266 c 209 g 312 t

ORIGIN

1 ggtttaatta cccaagtttg aggttccaat accggaaagc taaagcaaag tctcaactgc

61 gacgatttga tgagcttatg acacgtgttg gtcgcggtac catcaccggt actccactcg

121 ctcccgacga acaagttcta cgtgctgtct gcaagtggat acgcttgaca gcatcgtggt

181 ctaacaccat taccgtccaa tctcgagatt gaaccaccta acagagtcgt caccgccgaa

241 accgccatcg ttgtcgctgc cgtcaccgct cctcccctac ctactattca atgtacgccc

301 tcacactcac cattgctgct gccatggttg caagtacacc aactcagatc cagcttgttc

361 acgatctgct tgataaatac gacaagaagg cgaaaccaat gtgggacaat acgaaaccga

421 tcaatgtctc gttcaccgta tcactttacc agattcttga actaaacgaa ccacagcagt

481 tcgtactcct caacgcctgg attattgaga gatggtatga tgaatttttg tactggtccc

541 ctatggagta ccaaaatatc actgaattac ggctaccgta tgattcaatt tggttaccgg

601 atactacgct ttacaattcg ttggtaatga aggatgacga tactagacgc cttctgaatg

661 ccaagctaac gacagatctt caacggcgag catctcttat tgaacttctt tatcctacaa

721 tctacaaatt ctcttgtcta cttgacctac gatttttccc atttgatgta cagaattgta

781 caatgatttt ttcgtcatgg acctatgatc agacagggat cgattacttt cctgcttcag

841 atgaaatatc gattgctaat tatttggaaa atgaaggatg ggaacttttg aagactgaag

901 tgagccgtca tgaagtaaaa tacagctgct gtccgtatgc ctacacatta ctacatttaa

961 cactttattt gagaaggaag ccattattct atttggtgaa cctgatcatt cctacctcca

1021 tcatcacact tattgctatt gtgggattct tcacgacttc ttctgccagt ggaatgcgtg

1081 aagaga

//

LOCUS Hco-des-2H;m1-3 1098 bp mRNA linear INV 05-MAR-2009

DEFINITION [gene=Hco-des-2H].

SOURCE Haemonchus contortus

ORGANISM Haemonchus contortus

Eukaryota; Metazoa; Nematoda; Chromadorea; Rhabditida; Strongylida;

Trichostrongyloidea; Haemonchidae; Haemonchinae; Haemonchus.

REFERENCE 1 (bases 1 to 1098)

AUTHORS Rufener,L., Maser,P., Roditi,I. and Kaminsky,R.

TITLE Haemonchus contortus Acetylcholine Receptors of the DEG-3 Subfamily

and their Role in Sensitivity to Monepantel

JOURNAL PLoS Pathogens, in press

REFERENCE 2 (bases 1 to 1098)

AUTHORS Rufener,L., Maser,P., Roditi,I. and Kaminsky,R.

TITLE Direct Submission

JOURNAL Submitted (05-MAR-2009) Institute of Cell Biology, University of

Bern, Baltzerstrasse 4, Bern, BE 3012, Switzerland

FEATURES Location/Qualifiers

source 1..1098

/organism="Haemonchus contortus"

/mol_type="mRNA"

/isolate="Hc-CRA AAD-mutant"

/clone="m1-3"

/note="monepantel-insensitive isolate; mutant gene"

CDS 78..212

/note="Hco-DES-2H"

/codon_start=1

/product="H. contortus DES-2 homologue"

/translation="MTRVGRGTITGTPLAPDEQVLRAVCKWIRLTASWSNTITVQSRD"

BASE COUNT 302 a 271 c 211 g 314 t

ORIGIN

1 ggtttaatta cccaagtttg agattccaat accggaaagc taaagcaagg tctcaactgc

61 gacgatttga tgagcttatg acacgtgttg gtcgcggtac catcaccggt actccactcg

121 ctcccgacga acaagttcta cgtgctgtct gcaagtggat acgcttgaca gcatcgtggt

181 ctaacaccat taccgtccaa tctcgagatt gaaccaccta acagagtcgt caccgccgaa

241 accgccatcg ttgtcgctgc cgtcaccgct cctcccctac ctactattca atgtacgccc

301 tcacactcac cattgctgct gctctatctg cagccatggt tgcaagtaca ccaactcaga

361 tccagcttgt tcacgatctg cttgataaat acgacaagaa ggcgaaacca atgtgggaca

421 atacgaaacc gatcaatgtc tcgttcaccg tatcacttta ccagattctt gaactaaacg

481 aaccacagca gttcgtactc ctcaacgcct ggattattga gagatggtat gatgaatttt

541 tgtactggtc ccctatggag taccaaaata tcactgaatt acggctaccg tatgattcaa

601 tttggttacc ggatactacg ctttacaatt cgttggtaat gaaggatgac gatactagac

661 gccttctgaa tgccaagcca acgacagatc ttcaacggcg agcatctctt attgaacttc

721 tttatcctac aatctacaaa ttctcttgtc tacttgacct acgatttttc ccatttgatg

781 tacagaattg tacaatgatt ttttcgtcat ggacctatga tcagacaggg atcgattacc

841 ttcctgcttc agatgaaata tcgattgcta attatttgga aaatgaagga tgggaacttt

901 tgaagactga agtgagccgt catgaagtaa aatacagctg ctgtccgaat gcctacacat

961 tactacattt aacactttat ttgagaagga agccattatt ctatttggtg aacctgatca

1021 ttcctacctc tatcatcaca cttattgcta ttgtgggatt cttcacgact tcttctgcca

1081 gtggaatgcg tgaagaga

//

LOCUS Hco-des-2H;m1-4 1098 bp mRNA linear INV 05-MAR-2009

DEFINITION [gene=Hco-des-2H].

SOURCE Haemonchus contortus

ORGANISM Haemonchus contortus

Eukaryota; Metazoa; Nematoda; Chromadorea; Rhabditida; Strongylida;

Trichostrongyloidea; Haemonchidae; Haemonchinae; Haemonchus.

REFERENCE 1 (bases 1 to 1098)

AUTHORS Rufener,L., Maser,P., Roditi,I. and Kaminsky,R.

TITLE Haemonchus contortus Acetylcholine Receptors of the DEG-3 Subfamily

and their Role in Sensitivity to Monepantel

JOURNAL PLoS Pathogens, in press

REFERENCE 2 (bases 1 to 1098)

AUTHORS Rufener,L., Maser,P., Roditi,I. and Kaminsky,R.

TITLE Direct Submission

JOURNAL Submitted (05-MAR-2009) Institute of Cell Biology, University of

Bern, Baltzerstrasse 4, Bern, BE 3012, Switzerland

FEATURES Location/Qualifiers

source 1..1098

/organism="Haemonchus contortus"

/mol_type="mRNA"

/isolate="Hc-CRA AAD-mutant"

/clone="m1-4"

/note="monepantel-insensitive isolate; mutant gene"

CDS 78..212

/note="Hco-DES-2H"

/codon_start=1

/product="H. contortus DES-2 homologue"

/translation="MTRVGRGTITGTPLAPDEQVLRAVCKWIRLTASWSNTITVQSRD"

BASE COUNT 299 a 269 c 213 g 317 t

ORIGIN

1 ggtttaatta cccaagtttg aggttccaat accggaaagc taaagcaaag tctcaactgc

61 gacgatttga tgagcttatg acacgtgttg gtcgcggtac catcaccggt actccactcg

121 ctcccgacga acaagttcta cgtgctgtct gcaagtggat acgcttgaca gcatcgtggt

181 ctaacaccat taccgtccaa tctcgagatt gaaccaccta acagagtcgt caccgccgaa

241 accgccatcg ttgtcgctgc cgtcaccgct cctcccctac ctactattca atgtacgccc

301 tcgcactcac cattgctgct gctctatctg cagccatggt tgcaagtaca ccaactcaga

361 tccagcttgt tcacgatctg cttgataaat acgacaagaa ggcgaaacca atgtgggaca

421 atacgaaacc gatcaatgtc tcgttcaccg tatcacttta ccagattctt gaactaaacg

481 aaccacagca gttcgtactc ctcaacgcct ggattattga gagatggtat gatgaatttt

541 tgtactggtc ccctatggag taccaaaata tcactgaatt acggctaccg tatgattcaa

601 tttggttacc ggatactacg ctttacaatt cgttggtaat gaaggatgac gatactaggc

661 gccttctgaa tgccaagcta acgacagatc ttcaacggcg agcatctctt attgaacttc

721 tttatcctac aatctacaaa ttctcttgtc tacttgacct acgatttttc ccatttgatg

781 tacagaattg tacaatgatt ttttcgtcat ggacctatga tcagacaggg atcgattact

841 ttcctgcttc agatgaaata tcgattgcta attatttgga aaatgaagga tgggaacttt

901 tgaagactga agtgagccgt catgaagtaa aatacagctg ctgtccgaat gcctacacat

961 tactacattt aacactttat ttgagaagga agccattatt ctatttggtg aacctgatca

1021 ttcctacctc tatcttcaca cttattgcta ttgtgggatt cttcacgact tcttctgcca

1081 gtggaatgcg tgaagaga

//

LOCUS Hco-des-2H;m1-5 1086 bp mRNA linear INV 05-MAR-2009

DEFINITION [gene=Hco-des-2H].

SOURCE Haemonchus contortus

ORGANISM Haemonchus contortus

Eukaryota; Metazoa; Nematoda; Chromadorea; Rhabditida; Strongylida;

Trichostrongyloidea; Haemonchidae; Haemonchinae; Haemonchus.

REFERENCE 1 (bases 1 to 1086)

AUTHORS Rufener,L., Maser,P., Roditi,I. and Kaminsky,R.

TITLE Haemonchus contortus Acetylcholine Receptors of the DEG-3 Subfamily

and their Role in Sensitivity to Monepantel

JOURNAL PLoS Pathogens, in press

REFERENCE 2 (bases 1 to 1086)

AUTHORS Rufener,L., Maser,P., Roditi,I. and Kaminsky,R.

TITLE Direct Submission

JOURNAL Submitted (05-MAR-2009) Institute of Cell Biology, University of

Bern, Baltzerstrasse 4, Bern, BE 3012, Switzerland

FEATURES Location/Qualifiers

source 1..1086

/organism="Haemonchus contortus"

/mol_type="mRNA"

/isolate="Hc-CRA AAD-mutant"

/clone="m1-5"

/note="monepantel-insensitive isolate; mutant gene"

CDS 78..212

/note="Hco-DES-2H"

/codon_start=1

/product="H. contortus DES-2 homologue"

/translation="MTRVGRGTITGTPLAPDEQVLRAVCKWIRLTASWSNTITVQSRD"

BASE COUNT 300 a 264 c 209 g 312 t 1 others

ORIGIN

1 ggtttaatta cccaagtttg aggttccaat accggaaagc taaagcaaag tctcaactgc

61 gacgatttga tgagcttatg acacgtgttg gtcgcggtac catcaccggt actccactcg

121 ctcccgacga acaagttcta cgtgctgtct gcaagtggat acgcttgaca gcatcgtggt

181 ctaacaccat taccgtccaa tctcgagatt gaaccaccta acggagtcgt caccaccgaa

241 accgccatcg ttgtcgctgc cgtcaccgct cctcccctac ctactattca atgtacgccc

301 tcacactcac cattgctgct gccatggttg caagtacacc aactcagatc cagcttgttc

361 acgatctgct tgataaatac gacaagaagg cgaaaccaat gtgggacaat acgaaaccga

421 tcaatgtctc gttcaccgta tcactttacc agattcttga actaaacgaa ccacagcagt

481 tcgtactcct caacgcctgg attattgaga gatggtatga tgaatttttg tactggtccc

541 ctatggagta ccaaaatatc actgaattac ggctaccgta tgattcaatt tggttaccgg

601 atactacgct ttacaactcg ttggtaatga aggatgacga tactagacgc nttctgaatg

661 ccaagctaac gacagatctt caacggcgag catctcttat tgaacttctt tatcctacaa

721 tctacaaatt ctcttgtcta cttgacctat gatttttccc atttgatgta cagaattgta

781 caatgatttt ttcgtcatgg acctatgatc agacagggat cgattacttt cctgcttcag

841 atgaaatatc gattgctaat tatttggaaa atgaaggatg ggaacttttg aagactgaag

901 tgagccgtca tgaagtaaaa tacagctgct gtccgaatgc ctacacatta ctacatttaa

961 cactttattt gagaaggaag ccattattct atttggtgaa cctgatcatt cctacctcta

1021 tcatcacact tattgctatt gtgggattct tcacgacttc ttctgccagt ggaatgcgtg

1081 aagaga

//

LOCUS Hco-des-2H;21 963 bp mRNA linear INV 05-MAR-2009

DEFINITION [gene=Hco-des-2H].

SOURCE Haemonchus contortus

ORGANISM Haemonchus contortus

Eukaryota; Metazoa; Nematoda; Chromadorea; Rhabditida; Strongylida;

Trichostrongyloidea; Haemonchidae; Haemonchinae; Haemonchus.

REFERENCE 1 (bases 1 to 963)

AUTHORS Rufener,L., Maser,P., Roditi,I. and Kaminsky,R.

TITLE Haemonchus contortus Acetylcholine Receptors of the DEG-3 Subfamily

and their Role in Sensitivity to Monepantel

JOURNAL PLoS Pathogens, in press

REFERENCE 2 (bases 1 to 963)

AUTHORS Rufener,L., Maser,P., Roditi,I. and Kaminsky,R.

TITLE Direct Submission

JOURNAL Submitted (05-MAR-2009) Institute of Cell Biology, University of

Bern, Baltzerstrasse 4, Bern, BE 3012, Switzerland

FEATURES Location/Qualifiers

source 1..963

/organism="Haemonchus contortus"

/mol_type="mRNA"

/isolate="Hc-Howick"

/clone="wt-21"

/note="monepantel-sensitive isolate; wildtype gene"

CDS 156..>962

/note="Hco-DES-2H"

/codon_start=1

/product="H. contortus DES-2 homologue"

/translation="MYALTLTIAAALSAAMVASTPTQIQLVHDLLDKYDKKAKPMWDN

TKPINVSFTVSLYQILELNEPQQFVLLNAWIIERWYDEFLYWSPMEYQNITELRLPYD

SIWLPDTTLYNSLVMKDDDTRRLLNAKLTTDLQRRASLIELLYPTIYKFSCLLDLRFF

PFDVQNCTMIFSSWTYDQTGIDYFPASDEISIANYLENEGWELLKTEVSRHEVKYSCC

PNAYTLLHLTLYLRRKPLFYLVNLIIPTSIITLIAIVGFFTTSSASGMREE"

BASE COUNT 265 a 232 c 183 g 283 t

ORIGIN

1 ggtttaatta cccaagtttg aggatacgct tgacagcatc gtggtctaac accattgccg

61 tccaatctcg agattgaacc acctaacaga gtcgtcaccg ccgaaaccgc catcgttgtc

121 gctgccgtca ccgctcctcc cctacctact attcaatgta cgccctcaca ctcaccattg

181 ctgctgctct atctgcagcc atggttgcaa gtacaccgac tcagatccag ctcgttcacg

241 atctgctcga taagtatgac aaaaaggcga aaccaatgtg ggacaatacg aaaccgatca

301 atgtctcgtt caccgtatca ctttaccaga ttcttgaact aaacgaaccg cagcagttcg

361 tacttctcaa cgcctggatc attgagagat ggtatgatga atttttgtac tggtccccta

421 tggaatacca aaatatcact gaattacggc taccgtatga ttcaatttgg ttaccggata

481 ctacgcttta caattcgttg gtaatgaagg atgacgatac tagacgcctt ctgaatgcca

541 agctaacgac agatcttcaa cggcgagcat ctcttattga acttctttat cctacaatct

601 acaaattctc ttgtttgctt gatttacgat ttttcccatt tgatgtgcag aattgtacaa

661 tgattttttc gtcatggacc tatgatcaga cggggatcga ttacttccca gcatcagatg

721 agatatcgat tgctaattat ttggaaaatg aaggatggga acttttgaag actgaagtga

781 gccgtcatga agtaaaatac agctgctgcc ctaacgccta cacattacta catttaaccc

841 tttatttgag aaggaagcca ttattctatt tggttaatct gataatacct acctctatca

901 tcacacttat tgctattgtg ggattcttca cgacttcttc tgccagtgga atgcgtgaag

961 aga

//

LOCUS Hco-des-2H;22 963 bp mRNA linear INV 05-MAR-2009

DEFINITION [gene=Hco-des-2H].

SOURCE Haemonchus contortus

ORGANISM Haemonchus contortus

Eukaryota; Metazoa; Nematoda; Chromadorea; Rhabditida; Strongylida;

Trichostrongyloidea; Haemonchidae; Haemonchinae; Haemonchus.

REFERENCE 1 (bases 1 to 963)

AUTHORS Rufener,L., Maser,P., Roditi,I. and Kaminsky,R.

TITLE Haemonchus contortus Acetylcholine Receptors of the DEG-3 Subfamily

and their Role in Sensitivity to Monepantel

JOURNAL PLoS Pathogens, in press

REFERENCE 2 (bases 1 to 963)

AUTHORS Rufener,L., Maser,P., Roditi,I. and Kaminsky,R.

TITLE Direct Submission

JOURNAL Submitted (05-MAR-2009) Institute of Cell Biology, University of

Bern, Baltzerstrasse 4, Bern, BE 3012, Switzerland

FEATURES Location/Qualifiers

source 1..963

/organism="Haemonchus contortus"

/mol_type="mRNA"

/isolate="Hc-Howick"

/clone="wt-22"

/note="monepantel-sensitive isolate; wildtype gene"

CDS 156..>962

/note="Hco-DES-2H"

/codon_start=1

/product="H. contortus DES-2 homologue"

/translation="MYALTLTIAAALSAAMVASTPTQIQLVHDLLDKYDKKAKPMWDN

TKPINVSFTVSLYQILELNEPQQFVLLNAWIIERWYDEFLYWSPMEYQNITELRLPYD

SIWLPDTTLYNSLVMKDDDTRRLLNAKLTTDLQRRASLIELLYPTIYKFSCLLDLRFF

PFDVQNCTMIFSSWTYDQTGIDYFPASDEISIANYLENEGWELLKTEVSRHEVKYSCC

PNAYTLLHLTLYLRRKPLFYLVNLIIPTSIITLIAIVGFFTTSSASGMREE"

BASE COUNT 265 a 232 c 183 g 283 t

ORIGIN

1 ggtttaatta cccaagtttg aggatacgct tgacagcatc gtggtctaac accattgccg

61 tccaatctcg agattgaacc acctaacaga gtcgtcaccg ccgaaaccgc catcgttgtc

121 gctgccgtca ccgctcctcc cctacctact attcaatgta cgccctcaca ctcaccattg

181 ctgctgctct atctgcagcc atggttgcaa gtacaccgac tcagatccag ctcgttcacg

241 atctgctcga taagtatgac aaaaaggcga aaccaatgtg ggacaatacg aaaccgatca

301 atgtctcgtt caccgtatca ctttaccaga ttcttgaact aaacgaaccg cagcagttcg

361 tacttctcaa cgcctggatc attgagagat ggtatgatga atttttgtac tggtccccta

421 tggaatacca aaatatcact gaattacggc taccgtatga ttcaatttgg ttaccggata

481 ctacgcttta caattcgttg gtaatgaagg atgacgatac tagacgcctt ctgaatgcca

541 agctaacgac agatcttcaa cggcgagcat ctcttattga acttctttat cctacaatct

601 acaaattctc ttgtttgctt gatttacgat ttttcccatt tgatgtgcag aattgtacaa

661 tgattttttc gtcatggacc tatgatcaga cggggatcga ttacttccca gcatcagatg

721 agatatcgat tgctaattat ttggaaaatg aaggatggga acttttgaag actgaagtga

781 gccgtcatga agtaaaatac agctgctgcc ctaacgccta cacattacta catttaaccc

841 tttatttgag aaggaagcca ttattctatt tggttaatct gataatacct acctctatca

901 tcacacttat tgctattgtg ggattcttca cgacttcttc tgccagtgga atgcgtgaag

961 aga

//

LOCUS Hco-des-2H;m1-6 1098 bp mRNA linear INV 05-MAR-2009

DEFINITION [gene=Hco-des-2H].

SOURCE Haemonchus contortus

ORGANISM Haemonchus contortus

Eukaryota; Metazoa; Nematoda; Chromadorea; Rhabditida; Strongylida;

Trichostrongyloidea; Haemonchidae; Haemonchinae; Haemonchus.

REFERENCE 1 (bases 1 to 1098)

AUTHORS Rufener,L., Maser,P., Roditi,I. and Kaminsky,R.

TITLE Haemonchus contortus Acetylcholine Receptors of the DEG-3 Subfamily

and their Role in Sensitivity to Monepantel

JOURNAL PLoS Pathogens, in press

REFERENCE 2 (bases 1 to 1098)

AUTHORS Rufener,L., Maser,P., Roditi,I. and Kaminsky,R.

TITLE Direct Submission

JOURNAL Submitted (05-MAR-2009) Institute of Cell Biology, University of

Bern, Baltzerstrasse 4, Bern, BE 3012, Switzerland

FEATURES Location/Qualifiers

source 1..1098

/organism="Haemonchus contortus"

/mol_type="mRNA"

/isolate="Hc-Howick AAD-mutant"

/clone="m1-6"

/note="monepantel-insensitive isolate; mutant gene"

CDS 78..212

/note="Hco-DES-2H"

/codon_start=1

/product="H. contortus DES-2 homologue"

/translation="MTRVGRGTITGTPLAPDEQVLRAVCKWIRLTASWSNTIAVQSRD"

BASE COUNT 298 a 270 c 221 g 309 t

ORIGIN

1 ggtttaatta cccaagtttg aggttccaat accggaaagc taaagcaaag tctcaactgc

61 gacgatttga tgagcttatg acacgtgttg gtcgcggtac catcaccggt actccactcg

121 ctcccgacga acaagttcta cgtgctgtct gcaagtggat acgcttgaca gcatcgtggt

181 ctaacaccat tgccgtccaa tctcgagatt gaaccaccta acagagtcgt caccgccgaa

241 accgccatcg ttgtcgctgc cgtcaccgct cctcccctac ctaccattca atgtacgccc

301 tcacgctcac cattgctgcc gctctatctg cagctatggt tgcaagtaca ccgactcaga

361 ttcagctcgt tcacgatctg ctcgataagt atgacaaaaa ggcgaaacca atgtgggaca

421 atacgaaacc gatcaatgtc tcgttcaccg tatcactcta tcagattctt gaactaaacg

481 aaccacagca gttcgtactc cttaacgcct ggatcattga gagatggtat gatgaatttt

541 tgtactggtc ccctatggaa taccaaaata tcactgaatt acggctaccg tatgattcaa

601 tttggttgcc ggatactacg ctttacaatt cgttggtaat gaaggatgac gatactagac

661 gccttctgaa tgccaagcta acgacagatc ttcaacggcg agcatctctc attgaacttc

721 tttatcctac aatctacaaa ttctcttgtt tgcttgattt acgatttttc ccatttgatg

781 tgcagaattg tacaatgatt ttttcgtcgt ggacctatga tcagacgggg atcgattact

841 tcccagcatc agatgagata tcgattgcta actatttgga aaatgaagga tgggaacttt

901 tgaagactga agtgagtcgt catgaagtaa aatatagctg ctgcccgaat gcctacacat

961 tactgcattt aacgctttac ctgagaagaa aaccattgtt ctatttggtg aatctgataa

1021 tacctacctc gatcatcaca cttattgcta ttgtgggatt cttcacgaca tcctctgcca

1081 gtggaatgcg tgaagaga

//

LOCUS Hco-des-2H;m1-7 1098 bp mRNA linear INV 05-MAR-2009

DEFINITION [gene=Hco-des-2H].

SOURCE Haemonchus contortus

ORGANISM Haemonchus contortus

Eukaryota; Metazoa; Nematoda; Chromadorea; Rhabditida; Strongylida;

Trichostrongyloidea; Haemonchidae; Haemonchinae; Haemonchus.

REFERENCE 1 (bases 1 to 1098)

AUTHORS Rufener,L., Maser,P., Roditi,I. and Kaminsky,R.

TITLE Haemonchus contortus Acetylcholine Receptors of the DEG-3 Subfamily

and their Role in Sensitivity to Monepantel

JOURNAL PLoS Pathogens, in press

REFERENCE 2 (bases 1 to 1098)

AUTHORS Rufener,L., Maser,P., Roditi,I. and Kaminsky,R.

TITLE Direct Submission

JOURNAL Submitted (05-MAR-2009) Institute of Cell Biology, University of

Bern, Baltzerstrasse 4, Bern, BE 3012, Switzerland

FEATURES Location/Qualifiers

source 1..1098

/organism="Haemonchus contortus"

/mol_type="mRNA"

/isolate="Hc-Howick AAD-mutant"

/clone="m1-7"

/note="monepantel-insensitive isolate; mutant gene"

CDS 78..212

/note="Hco-DES-2H"

/codon_start=1

/product="H. contortus DES-2 homologue"

/translation="MTRVGRGTITGTPLAPDEQVLRAVCKWIRLTASWSNTIAVQSRD"

BASE COUNT 298 a 268 c 221 g 311 t

ORIGIN

1 ggtttaatta cccaagtttg aggttccaat accggaaagc taaagcaaag tctcaactgc

61 gacgatttga tgagcttatg acacgtgttg gtcgcggtac catcaccggt actccactcg

121 ctcccgacga acaagttcta cgtgctgtct gcaagtggat acgcttgaca gcatcgtggt

181 ctaacaccat tgccgtccaa tctcgagatt gaaccaccta acagagtcgt caccgccgaa

241 accgccatcg ttgtcgctgc cgtcaccgct cctcccctac ctactattca atgtacgccc

301 tcacgctcac cattgctgct gctctatctg cagctatggt tgcaagtaca ccgactcaga

361 ttcagctcgt tcacgatctg ctcgataagt atgacaaaaa ggcgaaacca atgtgggaca

421 atacgaaacc gatcaatgtc tcgttcaccg tatcactcta tcagattctt gaactaaacg

481 aaccacagca gttcgtactc cttaacgcct ggatcattga gagatggtat gatgaatttt

541 tgtactggtc ccctatggaa taccaaaata tcactgaatt acggctaccg tatgattcaa

601 tttggttgcc ggatactacg ctttacaatt cgttggtaat gaagggtgac gatactagac

661 gccttctgaa tgccaagcta acgacagatc ttcaacggcg agcatctctc attgaacttc

721 tttatcctac aatctacaaa ttctcttgtt tgcttgattt acgatttttc ccatttgatg

781 tgcagaattg tacaatgatt ttttcgtcgt ggacctatga tcagacgggg atcgattact

841 tcccagcatc agatgagata tcgattgcta actatttgga aaatgaagga tgggaacttt

901 tgaagactga agtgagtcgt catgaagtaa aatatagctg ctgcccgaat gcctacacat

961 tactacattt aacgctttac ctgagaagaa aaccattgtt ctatttggtg aatctgataa

1021 tacctacctc gatcatcaca cttattgcta ttgtgggatt cttcacgaca tcctctgcca

1081 gtggaatgcg tgaagaga

//

LOCUS Hco-deg-3H;1 1710 bp mRNA linear INV 05-MAR-2009

DEFINITION [gene=Hco-deg-3H].

SOURCE Haemonchus contortus

ORGANISM Haemonchus contortus

Eukaryota; Metazoa; Nematoda; Chromadorea; Rhabditida; Strongylida;

Trichostrongyloidea; Haemonchidae; Haemonchinae; Haemonchus.

REFERENCE 1 (bases 1 to 1710)

AUTHORS Rufener,L., Maser,P., Roditi,I. and Kaminsky,R.

TITLE Haemonchus contortus Acetylcholine Receptors of the DEG-3 Subfamily

and their Role in Sensitivity to Monepantel

JOURNAL PLoS Pathogens, in press

REFERENCE 2 (bases 1 to 1710)

AUTHORS Rufener,L., Maser,P., Roditi,I. and Kaminsky,R.

TITLE Direct Submission

JOURNAL Submitted (05-MAR-2009) Institute of Cell Biology, University of

Bern, Baltzerstrasse 4, Bern, BE 3012, Switzerland

FEATURES Location/Qualifiers

source 1..1710

/organism="Haemonchus contortus"

/mol_type="mRNA"

/isolate="Hc-CRA"

/clone="1"

/note="monepantel-sensitive isolate"

CDS 1..1710

/note="Hco-DEG-3H"

/codon_start=1

/product="H. contortus DEG-3 homologue"

/translation="MRLHETSTMIFAVLLVVFSGFGVSKADLVSSLLINDSAASHTRA

TIMEEVPLIRLTRDLLMKDRYDVRVRPIHDHTKPLKVHISISLYQIIEVDEPAQNIKL

NVWMIQKWKDEYLSWDPREYGMINSTIIPFRHLWIPDTYLYNSVKMSRDETERYMNIQ

VESLHWKGENGSQMSFLYPAIYTITCRLNIRFFPYDRQNCTLTISSWTNSMSALDYYA

DPEVNLASFIPNEEWDVKSFKIFRHEYKYACCAEPWAILQASLVIQRKPLYYIVNLII

PTSIITIVSITGFFTPASTDDDRTEKINLGITTLLAMSILMLMVSDQMPTTSEFVPLI

AWFYLSIIIIVSIGTFLTSVVLSVQSRRQYGRNPPLYIRYYFFVVIPSFIYVSVPPAL

ENLWSELDDDPLNAWRRRRRSSSPGLHKTESLYKDQTPISPPPLERNSTLKIPRQGLD

RFSSIQMIDVSVPPSPAISRISRTPSTAQSQKLTLWEGTMSALAGNNVQLRRTSAAYS

KEVDAMRRKRQCSLEWEFLATVLDRFLLLLFISAVVLITMGLVVVGKMAQFSYDHPDE

AFF"

BASE COUNT 479 a 399 c 363 g 468 t 1 others

ORIGIN

1 atgcgactac atgaaacctc gacaatgata ttcgctgttc ttctagtggt tttctctgga

61 ttcggggtct ctaaagcaga ccttgttagt tcgttgctga ttaatgactc tgctgcaagt

121 catactcgag cgacaatcat ggaagaagtt cctctcatac ggcttacgcg agaccttctc

181 atgaaagata ggtatgatgt tcgtgttcga cctattcacg accacacgaa gccactgaaa

241 gtccacatca gcatttcgct ctaccaaatc atcgaagtgg atgaaccggc acagaatatc

301 aagttaaacg tgtggatgat tcaaaaatgg aaagatgagt atctatcgtg ggatccaagg

361 gaatacggta tgatcaattc gacgatcatc ccattcagac atctatggat acccgacaca

421 tacctttata acagtgtgaa gatgagtcgt gatgagactg aacggtatat gaatatccaa

481 gtcgaatcgt tacattggaa aggagaaaac ggatcacaaa tgtcatttct ctatcccgct

541 atatacacga tcacctgtcg actcaacatc agattcttcc cgtacgatcg tcagaattgt

601 acattgacaa tatcaagttg gacgaattcg atgtcggcat tggactatta tgcggatcct

661 gaagtaaatc tggcatcgtt cataccgaat gaagaatggg atgtgaaatc gttcaagatc

721 tttagacatg agtacaagta cgcgtgctgt gcagaaccat gggccatttt acaagcatca

781 cttgtgattc aacgaaagcc actctattac atagttaatc tgataattcc aacatcgatc

841 atcactatcg tctccatcac cggattcttc acaccggctt cgacagacga cgaccgtacc

901 gaaaagatca atctgggtat tacgacgttg ctggcgatgt ccatcctaat gcttatggtt

961 tccgatcaaa tgccgacnac cagtgaattc gtcccattga tagcatggtt ctacctatcc

1021 atcataatta tcgtcagtat tggaacattc cttaccagcg tcgtgctgtc tgttcaaagt

1081 cgaaggcaat acgggcgaaa tccacccctt tatattcgtt attacttctt tgttgttatt

1141 ccatcgttca tatacgtcag tgtaccacca gcgctggaaa atttgtggtc cgaactcgat

1201 gatgacccat tgaatgcttg gcgacgacga cggcgatcat cttctcctgg tcttcataag

1261 actgaatctc tatacaaaga ccagacaccg atatctcctc ctccactaga gcggaattca

1321 acgctgaaga tccctcgaca aggtttggat cgattcagtt ctattcaaat gatcgatgtc

1381 tctgtgccac catcgccggc gatttcacga atctcaagaa ctccgtcaac ggcacaatca

1441 caaaagctca cattatggga gggtacgatg agtgcattag caggaaacaa cgtgcagtta

1501 cgtcgtacat cagccgcata ctctaaagaa gtagatgcta tgcgaaggaa acggcaatgc

1561 tcattggagt gggagttcct cgccacagtc cttgataggt tcctgctgtt gctgttcatc

1621 tcagctgtgg tgttgattac gatgggtcta gtggtcgttg gtaaaatggc tcaattcagc

1681 tacgatcatc cagacgaggc attcttctaa

//

LOCUS Hco-deg-3H;2 1707 bp mRNA linear INV 05-MAR-2009

DEFINITION [gene=Hco-deg-3H].

SOURCE Haemonchus contortus

ORGANISM Haemonchus contortus

Eukaryota; Metazoa; Nematoda; Chromadorea; Rhabditida; Strongylida;

Trichostrongyloidea; Haemonchidae; Haemonchinae; Haemonchus.

REFERENCE 1 (bases 1 to 1707)

AUTHORS Rufener,L., Maser,P., Roditi,I. and Kaminsky,R.

TITLE Haemonchus contortus Acetylcholine Receptors of the DEG-3 Subfamily

and their Role in Sensitivity to Monepantel

JOURNAL PLoS Pathogens, in press

REFERENCE 2 (bases 1 to 1707)

AUTHORS Rufener,L., Maser,P., Roditi,I. and Kaminsky,R.

TITLE Direct Submission

JOURNAL Submitted (05-MAR-2009) Institute of Cell Biology, University of

Bern, Baltzerstrasse 4, Bern, BE 3012, Switzerland

FEATURES Location/Qualifiers

source 1..1707

/organism="Haemonchus contortus"

/mol_type="mRNA"

/isolate="Hc-CRA"

/clone="2"

/note="monepantel-sensitive isolate"

CDS 1..1707

/note="Hco-DEG-3H"

/codon_start=1

/product="H. contortus DEG-3 homologue"

/translation="MRLHETSTMIFAVLLVVFSGFRVSKADLVSSLLINDSAASHTRA

TIMEEVPLIRLTRDLLMKDRYDVRVRPIHDHTKPLKVHISISLYQIIEVDEPAQNIKL

NVWMIQKWKDEYLSWDPREYGMINSTIIPFRHLWIPDTYLYNSVKMSRDETERYMNIQ

VESLHWKGENGSQMSFLYPAIYTITCRLNIRFFPYDRQNCTLTISSWTNSMSALDYYA

DPEVNLASFIPNEEWDVKSFKIFRHEYKYACCAEPWAILQASLVIQRKPLYYIVNLII

PTSIITIVSITGFFTPASTDDDRTEKINLGITTLLAMSILMLMVSDQMPTTSEFVPLI

AWFYLSIIIIVSIGTFLTSVVLSVQSRRQYGRNPPLYIRYYFFVVIPSFIYVSVPPAL

ENLWSELDDDPLNAWRRRRRSSSPGLHKTESLYKDQTPISPPLERNSTLKIPRQGLDR

FSSIQMIDVSVPPSPAISRISRTPSTAQSQKLTLWEGTMSALAGNNVQLRRTSAAYSK

EVDAMRRKRQCSLEWEFLATVLDRFLLLLFISAVVLITMGLVVVGKMAQFSYDHPDEA

FF"

BASE COUNT 475 a 395 c 372 g 465 t

ORIGIN

1 atgcgactac atgaaacctc gacaatgata ttcgctgttc ttctggtggt cttctctgga

61 ttcagggtct ccaaagcaga ccttgttagt tcgttgctga ttaatgactc tgctgcaagt

121 catactcgag cgacaatcat ggaagaagtt cctctgatac ggcttactcg ggacctgctc

181 atgaaagata ggtatgatgt tcgtgttcga ccgattcacg accacacaaa gccactgaaa

241 gtccacatca gcatttcgct ttaccaaatt atcgaagtgg atgaaccggc ccagaatatc

301 aagttgaacg tgtggatgat acaaaaatgg aaagatgagt atttatcatg ggatccaagg

361 gaatacggta tgatcaattc gacgatcatc ccattcagac atctatggat tcctgacaca

421 tacctttata acagtgtgaa gatgagtcgt gatgaaaccg aacggtatat gaacatccag

481 gtcgaatcgt tacattggaa aggagaaaac ggctcacaaa tgtcatttct ctatccggct

541 atatatacga tcacttgtcg gctcaatatc agattcttcc cgtacgatcg tcagaattgt

601 acgttgacaa tatccagttg gacaaattcg atgtcggcgt tggactatta tgcggatcct

661 gaagtaaatc tagcatcatt cataccgaat gaagaatggg atgttaaatc gttcaagatc

721 tttcgacatg agtacaagta cgcttgctgt gcagaaccat gggccatttt acaagcatca

781 ctggtgattc aacgaaagcc gctctactat atagtcaatt tgataattcc aacatcgatc

841 atcacgatcg tctccatcac cggattcttt acaccagcat cgacagacga cgaccgtacc

901 gagaagatta atctgggtat tacgacgttg ctggcgatgt ccatcctaat gcttatggtt

961 tccgatcaaa tgccgacaac cagtgaattc gtcccactaa tagcatggtt ctacttatcc

1021 atcataatta tcgtcagtat tggaacattc cttacgagcg tcgtgctctc tgttcaaagt

1081 cgaaggcaat acgggcgaaa tccaccgctt tatattcgtt attacttctt tgttgttatt

1141 ccatcattca tatacgtcag tgtacctcca gcgttggaga acttgtggtc tgaactcgac

1201 gatgacccat tgaatgcttg gcgacgacga cggcgatcat cttctcctgg tcttcataag

1261 actgaatctc tatacaaaga ccagacaccg atatctcctc cactagagcg gaattcaacg

1321 ctgaagatcc ctcgacaagg tttggatcga ttcagctcaa ttcaaatgat cgatgtctcc

1381 gtgccaccat cgccggcgat ttcacgaatc tcaagaactc cgtcgacggc gcaatcacaa

1441 aaactcacgt tatgggaggg tacgatgagt gcgctggcag gaaacaacgt gcagttacgt

1501 cgtacatcag ccgcatactc taaagaagta gatgctatgc gaaggaaacg acaatgctca

1561 ttggagtggg agttccttgc cacagtactc gataggttcc tgctgttgct gttcatctca

1621 gcggtggtgt tgatcactat gggtctagtg gtggttggaa aaatggctca gttcagctac

1681 gatcatccag acgaggcatt cttctaa

//

LOCUS Hco-deg-3H;3 1710 bp mRNA linear INV 05-MAR-2009

DEFINITION [gene=Hco-deg-3H].

SOURCE Haemonchus contortus

ORGANISM Haemonchus contortus

Eukaryota; Metazoa; Nematoda; Chromadorea; Rhabditida; Strongylida;

Trichostrongyloidea; Haemonchidae; Haemonchinae; Haemonchus.

REFERENCE 1 (bases 1 to 1710)

AUTHORS Rufener,L., Maser,P., Roditi,I. and Kaminsky,R.

TITLE Haemonchus contortus Acetylcholine Receptors of the DEG-3 Subfamily

and their Role in Sensitivity to Monepantel

JOURNAL PLoS Pathogens, in press

REFERENCE 2 (bases 1 to 1710)

AUTHORS Rufener,L., Maser,P., Roditi,I. and Kaminsky,R.

TITLE Direct Submission

JOURNAL Submitted (05-MAR-2009) Institute of Cell Biology, University of

Bern, Baltzerstrasse 4, Bern, BE 3012, Switzerland

FEATURES Location/Qualifiers

source 1..1710

/organism="Haemonchus contortus"

/mol_type="mRNA"

/isolate="Hc-CRA"

/clone="3"

/note="monepantel-sensitive isolate"

CDS 1..1710

/note="Hco-DEG-3H"

/codon_start=1

/product="H. contortus DEG-3 homologue"

/translation="MRLHETSTMIFAVLLVVFSGCRVSKADLVSSLLINDSAASHTRA

TIMEEVPLIRLTRDLLMKDRYDVRVRPIHDHTKPLKVHISISLYQIIEVDEPAQNIKL

NVWMIQKWKDEYLSWDPREYGMINSTIIPFRHLWIPDTYLYNSVKMSRDETERYMNIQ

VESLRWKGENGSQMSFLYPAIYTITCRLNIRFFPYDRQNCTLTISSWTNSMSALDYYA

DPEVNLASFIPNEEWDVKSFKIFRHEYKYACCAEPWAILQASLVIQRKPLYYIVNLII

PTSIITIVSITGFFTPASTDDDRTEKINLGITTLLAMSILMLMVSDQMPTTSEFVPLI

AWFYLSIIIIVSIGTFLTSVVLSVQSRRQYGRNPPLYIRYYFFVVIPSFIYVSVPPAL

ENLWSELDDDPLDAWRRRRRSPSPGLHKTESLYKDQTPISPPPLERNSTLKIPRQGLD

RFSSIQMIDVSVPPSPAISRISRTPSTAQSQKLTLWEGTMSALAGNNVQLRRTSAAYS

KEVDAMRRKRQCSLEWEFLATVLDRFLLLLFISAVVLITMGLVVVGKMAQFSYDHPDE

AFF"

BASE COUNT 465 a 398 c 375 g 472 t

ORIGIN

1 atgcgactac atgaaacctc gacaatgata ttcgctgttc ttctggtggt tttctctggt

61 tgcagggtct ccaaagcaga ccttgttagt tcgttgctga ttaatgactc tgctgcaagt

121 catactcgag cgacaatcat ggaagaagtt cctctgatac ggcttactcg agaccttctc

181 atgaaagata ggtatgatgt tcgtgttcga ccgattcacg accatacaaa gccactgaaa

241 gtccacatca gcatttcgct ttaccaaatt atcgaagtgg atgaaccggc ccagaatatc

301 aagttgaacg tgtggatgat tcaaaaatgg aaagatgagt atttatcatg ggatccaagg

361 gaatacggta tgatcaattc gacgatcatt ccattcagac atctatggat tcccgacaca

421 tacctttata acagcgtgaa gatgagtcgc gatgagactg aacggtatat gaacatccag

481 gttgaatcgt tacgttggaa aggagaaaac ggttcacaaa tgtcatttct ctatccggcc

541 atatacacga tcacttgtcg actcaatatc agatttttcc cgtacgatcg tcagaattgt

601 acgttgacaa tatccagctg gacgaattca atgtcggcgt tggactatta tgcggatcct

661 gaagtaaatc tggcatcgtt tataccaaat gaagaatggg atgtgaaatc gttcaagatc

721 tttagacatg agtacaagta cgcttgctgt gcggaaccat gggccatttt acaagcctcg

781 ctggtgattc aacgaaagcc actctactac atagtcaatt tgataattcc aacatcaatc

841 atcactatag tctctatcac cggatttttc acaccggcat cgacagacga cgaccgtacc

901 gagaagatta atctcggtat tacgacgttg ctggcgatgt ccattcttat gcttatggtt

961 tccgatcaaa tgccaacaac cagtgaattc gtcccgctca tagcatggtt ctacctatcc

1021 atcataatta tcgtcagtat tggaacattc cttacgagcg tcgtgctctc tgtccaaagt

1081 cgaaggcaat acgggcgaaa tccaccgctt tatattcgtt attacttctt tgttgttatc

1141 ccatcattca tatacgtcag tgtacctcca gcgttggaga acttgtggtc cgaactcgac

1201 gatgacccat tggatgcttg gcgacgacga cgacgatcac cttctcctgg tcttcataag

1261 actgaatctc tatacaaaga ccagacaccg atatctcctc ctccactgga gcggaattca

1321 acgctgaaga tccctcgaca aggtttggat cgattcagtt cgattcaaat gatcgatgtc

1381 tctgtgccac catcgccggc gatttcacga atctcaagaa ctccgtcgac ggcgcaatca

1441 caaaagctca cgttatggga gggtacgatg agtgcgttgg caggaaacaa cgtgcagtta

1501 cgtcgtacat cagctgcata ctccaaagag gtagatgcta tgcgaaggaa acgacaatgc

1561 tcattggaat gggagttcct tgccacagtc ctcgataggt tcctgctgtt gctgttcatc

1621 tcagctgtgg tgttgatcac tatgggtcta gtggtggttg gtaaaatggc tcagttcagc

1681 tacgatcatc cagacgaggc attcttctaa

//

LOCUS Hco-deg-3H;4 1710 bp mRNA linear INV 05-MAR-2009

DEFINITION [gene=Hco-deg-3H].

SOURCE Haemonchus contortus

ORGANISM Haemonchus contortus

Eukaryota; Metazoa; Nematoda; Chromadorea; Rhabditida; Strongylida;

Trichostrongyloidea; Haemonchidae; Haemonchinae; Haemonchus.

REFERENCE 1 (bases 1 to 1710)

AUTHORS Rufener,L., Maser,P., Roditi,I. and Kaminsky,R.

TITLE Haemonchus contortus Acetylcholine Receptors of the DEG-3 Subfamily

and their Role in Sensitivity to Monepantel

JOURNAL PLoS Pathogens, in press

REFERENCE 2 (bases 1 to 1710)

AUTHORS Rufener,L., Maser,P., Roditi,I. and Kaminsky,R.

TITLE Direct Submission

JOURNAL Submitted (05-MAR-2009) Institute of Cell Biology, University of

Bern, Baltzerstrasse 4, Bern, BE 3012, Switzerland

FEATURES Location/Qualifiers

source 1..1710

/organism="Haemonchus contortus"

/mol_type="mRNA"

/isolate="Hc-CRA"

/clone="4"

/note="monepantel-sensitive isolate"

CDS 1..1710

/note="Hco-DEG-3H"

/codon_start=1

/product="H. contortus DEG-3 homologue"

/translation="MRLHETSTMIFAVLLVVFSGFRVSKADLVSSLLINDSAASHTRA

TIMEEVPLIRLTRDLLMKDRYDVRVRPIHDHTKPLKVHISISLYQIIEVDEPAQNIKL

NVWMIQKWKDEYLSWDPREYGMINSTIIPFRHLWIPDTYLYNSVKMSRDETERYMNIQ

VESLHWKGENGSQMSFLYPAIYTITCRLNIRFFPYDRQNCTLTISSWTNSMSALDYYA

DPEVNLASFIPNEEWDVKSFKIFRHEYKYACCAEPWAILQASLVIQRKPLYYIVNLII

PTSIITIVSITGFFTPASTDDDRTEKINLGITTLLAMSILMLMVSDQMPTTSEFVPLI

AWFYLSIIIIVSIGTFLTSVVLSVQSRRQYGRNPPLYIRYYFFVVIPSFIYVSVPPAL

ENLWSELDDDPLNAWRRRRRSSSPGLHKTESLYKDQTPISPPPLERNSTLKIPRQGLD

RFSSIQMIDVSVPPSPAISRISRTPSTAQSQKLTLWEGTMSALAGNNVQLRRTSAAYS

KEVDAMRRKRQCSLEWEFLATVLDRFLLLLFISAVVLITMGLVVVGKMAQFSYDHPDE

AFF"

BASE COUNT 468 a 398 c 373 g 471 t

ORIGIN

1 atgcgactac atgaaacctc gacaatgata ttcgctgttc ttctggtggt tttctctgga

61 ttcagggtct ccaaagcaga ccttgtcagt tcgttgctga ttaatgactc tgctgcaagt

121 catactcgag cgacaatcat ggaagaagtt cctctcatac ggcttactcg agacctgctc

181 atgaaagata ggtatgatgt tcgtgttcga ccgattcacg accacacgaa accactgaaa

241 gtccacatca gcatttcgct ttatcaaatt atcgaagtgg atgaaccggc ccagaatatc

301 aagttgaacg tgtggatgat acaaaaatgg aaggatgagt atttatcatg ggatccaagg

361 gaatacggta tgatcaattc gacgatcatt ccattcagac atctatggat tcctgacaca

421 tacctttata acagtgtgaa gatgagtcgt gatgaaaccg aacggtatat gaacatccag

481 gtcgaatcgt tacattggaa aggagaaaac ggctcacaaa tgtcatttct ctatccggct

541 atatatacga tcacttgtcg gctcaatatc agattcttcc cgtacgatcg tcaaaattgt

601 acgttgacaa tatccagttg gacgaattcc atgtcggcgt tggattacta tgcggatcct

661 gaagtaaatc tggcatcgtt cataccgaat gaagaatggg atgttaagtc gttcaagatc

721 tttcgacatg agtacaagta cgcttgttgt gcggagccat gggccatttt acaagcatcg

781 ctggtgattc agcgaaagcc gctctactat atcgtcaatt tgataattcc aacatcgatc

841 atcactatcg tctccattac cggattcttc acaccggctt cgacagacga cgaccgtacc

901 gagaagatta atctgggtat tacgacactg ctggcgatgt ccattcttat gctcatggtt

961 tccgatcaga tgccgacaac cagtgaattc gtcccactaa tagcatggtt ctacctatcc

1021 atcataataa tcgtcagtat tggaacattc cttaccagcg tcgtgctgtc tgttcaaagt

1081 cgaaggcaat atgggcgaaa tccaccacta tatattcgat attatttctt tgttgtcatt

1141 ccatcgttca tatacgtcag tgtacctcca gcgttggaga acttgtggtc cgaactcgac

1201 gacgatccac tgaatgcttg gcgacgacga cggcgatcat cttcacctgg tcttcataag

1261 actgaatctc tatacaaaga ccagacaccg atatctcctc ctccactaga gcggaattca

1321 acgctgaaga tccctcgaca aggtttggat agattcagtt cgattcaaat gatcgatgtc

1381 tctgtgccac catcgccggc gatttcacga atctcccgaa ctccgtcaac ggcacaatca

1441 caaaagctca cattatggga gggtacgatg agtgcattag caggaaacaa cgtgcagtta

1501 cgtcgtacat cagccgcata ctctaaagaa gtagatgcta tgcgaaggaa acggcaatgc

1561 tcattggagt gggagttcct tgccacagta ctcgataggt tcctgctgtt gctgttcatc

1621 tcagctgtgg tgttgattac gatgggtcta gttgtcgttg gtaaaatggc tcagttcagc

1681 tacgatcatc cagacgaggc attcttctaa

//

LOCUS Hco-deg-3H;5 1710 bp mRNA linear INV 05-MAR-2009

DEFINITION [gene=Hco-deg-3H].

SOURCE Haemonchus contortus

ORGANISM Haemonchus contortus

Eukaryota; Metazoa; Nematoda; Chromadorea; Rhabditida; Strongylida;

Trichostrongyloidea; Haemonchidae; Haemonchinae; Haemonchus.

REFERENCE 1 (bases 1 to 1710)

AUTHORS Rufener,L., Maser,P., Roditi,I. and Kaminsky,R.

TITLE Haemonchus contortus Acetylcholine Receptors of the DEG-3 Subfamily

and their Role in Sensitivity to Monepantel

JOURNAL PLoS Pathogens, in press

REFERENCE 2 (bases 1 to 1710)

AUTHORS Rufener,L., Maser,P., Roditi,I. and Kaminsky,R.

TITLE Direct Submission

JOURNAL Submitted (05-MAR-2009) Institute of Cell Biology, University of

Bern, Baltzerstrasse 4, Bern, BE 3012, Switzerland

FEATURES Location/Qualifiers

source 1..1710

/organism="Haemonchus contortus"

/mol_type="mRNA"

/isolate="Hc-CRA"

/clone="5"

/note="monepantel-sensitive isolate"

CDS 1..1710

/note="Hco-DEG-3H"

/codon_start=1

/product="H. contortus DEG-3 homologue"

/translation="MRLHETSTMIFAVLLVVFSGCRVSKADLVSSLLINDSAASHTRA

TIMEEVPLIRLTRDLLMKDRYDVRVRPIHDHTKPLKVHISISLYQIIEVDEPAQNIKL

NVWMIQKWKDEYLSWDPREYGMINSTIIPFRHLWIPDTYLYNSVKMSRDETERYMNIQ

VESLHWKGENGSQMSFLYPAIYTITCRLNIRFFPYDRQNCTLTISSWTNSMSALDYYA

DPEVNLASFIPNEEWDVKSFKIFRHEYKYACCAEPWAILQASLVIQRKPLYYIVNLII

PTSIITIVSITGFFTPASTDDDRTEKINLGITTLLAMSILMLMVSDQMPTTSEFVPLI

AWFYLSIIIIVSIGTFLTSVVLSVQSRRQYGRNPPLYIRYYFFVVIPSFIYVSVPPAL

ENLWSELDDDPLNAWRRRRRSPSPGLHKTESLYKDQTPISPPPLERNSTLKIPRQGLD

RFSSIQMIDVSVPPSPAISRISRTPSTAQSQKLTLWEGTMSALAGNNVQLRRTSAAYS

KEVDAMRRKRQCSLEWEFLATVLDRFLLLLFISAVVLITMGLVVVGKMAQFSYDHSDE

AFF"

BASE COUNT 467 a 395 c 372 g 476 t

ORIGIN

1 atgcgactac atgaaacctc gacaatgata ttcgctgttc ttctggtggt tttctctggt

61 tgcagggtct ccaaagcaga ccttgttagt tcgttgctga ttaatgactc tgctgcaagt

121 catactcgag cgacaatcat ggaagaagtt cctctgatac ggcttactcg agaccttctc

181 atgaaagata ggtatgatgt tcgtgttcga ccgattcacg accatacaaa gccactgaaa

241 gtccacatca gcatttcgct ttaccaaatt atcgaagtgg atgaaccggc ccagaatatc

301 aagttgaacg tgtggatgat tcaaaaatgg aaagatgagt atttatcatg ggatccaagg

361 gaatacggta tgatcaattc gacgatcatt ccattcagac atctatggat tcccgacaca

421 tacctttata acagcgtgaa gatgagtcgt gatgagactg aacggtatat gaacatccag

481 gttgaatcgt tacattggaa aggagaaaac ggttcacaaa tgtcatttct ctatccggcc

541 atttacacga tcacttgtcg actcaatatc agatttttcc cgtacgatcg tcagaattgt

601 acgttgacaa tatccagctg gacgaattca atgtcggcgt tggactatta tgcggatcct

661 gaagtaaatc tggcatcgtt tataccaaat gaagaatggg atgtgaaatc gttcaagatc

721 tttagacatg agtacaagta cgcttgctgt gcggaaccat gggccatttt acaagcctcg

781 ctggtgattc aacgaaagcc actctattac atagtcaatt tgataattcc aacatcaatc

841 atcactatag tctctatcac cggatttttc acaccggcat cgacagacga cgaccgtacc

901 gagaagatta atctcggtat tacgacgttg ctggcgatgt ccattcttat gcttatggtt

961 tccgatcaaa tgccaacaac cagtgaattc gtcccgctca tagcatggtt ctacctatcc

1021 atcataatta tcgtcagtat tggaacattc cttacgagcg tcgtgctctc tgtccaaagt

1081 cgaaggcaat acgggcgaaa tccaccgctt tatattcgtt attacttctt tgttgttatc

1141 ccatcattca tatacgtcag tgtacctcca gcgttggaga acttgtggtc cgaactcgac

1201 gatgacccat tgaatgcttg gcgacgacga cgacgatcac cttctcctgg tcttcataag

1261 actgaatctc tatacaaaga ccagacaccg atatctcctc ctccactgga gcggaattca

1321 acgctgaaga tccctcgaca aggtttggat cgattcagtt cgattcaaat gatcgatgtc

1381 tctgtgccac catcgccggc gatttcacga atctcaagaa ctccgtcgac ggcgcaatca

1441 caaaaactca cgttatggga gggtacgatg agtgcgttgg caggaaacaa cgtgcagtta

1501 cgtcgtacat cagctgcata ctccaaagag gtagatgcta tgcgaaggaa acgacaatgc

1561 tcattggaat gggagttcct tgccacagtc ctcgataggt tcctgctgtt gctgttcatc

1621 tcagctgtgg tgttgatcac tatgggtcta gtggtggttg gtaaaatggc tcagttcagc

1681 tacgatcatt cagacgaggc attcttctaa

//

LOCUS Hco-deg-3H;6 1710 bp mRNA linear INV 05-MAR-2009

DEFINITION [gene=Hco-deg-3H].

SOURCE Haemonchus contortus

ORGANISM Haemonchus contortus

Eukaryota; Metazoa; Nematoda; Chromadorea; Rhabditida; Strongylida;

Trichostrongyloidea; Haemonchidae; Haemonchinae; Haemonchus.

REFERENCE 1 (bases 1 to 1710)

AUTHORS Rufener,L., Maser,P., Roditi,I. and Kaminsky,R.

TITLE Haemonchus contortus Acetylcholine Receptors of the DEG-3 Subfamily

and their Role in Sensitivity to Monepantel

JOURNAL PLoS Pathogens, in press

REFERENCE 2 (bases 1 to 1710)

AUTHORS Rufener,L., Maser,P., Roditi,I. and Kaminsky,R.

TITLE Direct Submission

JOURNAL Submitted (05-MAR-2009) Institute of Cell Biology, University of

Bern, Baltzerstrasse 4, Bern, BE 3012, Switzerland

FEATURES Location/Qualifiers

source 1..1710

/organism="Haemonchus contortus"

/mol_type="mRNA"

/isolate="Hc-CRA AAD-mutant"

/clone="6"

/note="monepantel-insensitive isolate"

CDS 1..1710

/note="Hco-DEG-3H"

/codon_start=1

/product="H. contortus DEG-3 homologue"

/translation="MRLHETSTMIFAVLLVVFSGCRVSKADLVSSLLINDSAASHTRA

TIMEEVPLIRLTRDLLMKDRYDVRVRPIHDHTKPLKVHISISLYQIIEVDEPAQNIKL

NVWMIQKWKDEYLSWDPREYGMINSTIIPFRHLWIPDTYLYNSVKMSRDETERYMNIQ

VESLHWKGENGSQMSFLYPAIYTITCRLNIRFFPYDRQNCTLTISSWTNSMSALDYYA

DPEVNLASFIPNEEWDVKSFKIFRHEYKYACCAEPWAILQASLVIQRKPLYYIVNLII

PTSIITIVSITGFFTPASTDDDRTEKINLGITTLLAMSILMLMVSDQMPTTSEFVPLI

AWFYLSIIIIVSIGTFLTSVVLSVQSRRQYGRNPPLYIRYYFFVVIPSFIYVSVPPAL

ENLWSELDDDPLNAWRRRRRSPSPGLHKTESLYKDQTPISPPPLERNSTLKIPRQGLD

RFSSIQMIDVSVPPSPAISRISRTPSTAQSQKLTLWEGTMSALAGNNVQLRRTSAAYS

KEVDAMRRKRQCSLEWEFLATVLDRFLLLLFISAVVLITMGLVVVGKMAQFSYDHPDE

AFF"

BASE COUNT 468 a 396 c 372 g 474 t

ORIGIN

1 atgcgactac atgaaacctc gacaatgata ttcgctgttc ttctggtggt tttctctggt

61 tgcagggtct ccaaagcaga ccttgttagt tcgttgctga ttaatgactc tgctgcaagt

121 catactcgag cgacaatcat ggaagaagtt cctctgatac ggcttactcg agaccttctc

181 atgaaagata ggtatgatgt tcgtgttcga ccgattcacg accatacaaa gccactgaaa

241 gtccacatca gcatttcgct ttaccaaatt atcgaagtgg atgaaccggc ccagaatatc

301 aagttgaacg tgtggatgat tcaaaaatgg aaagatgagt atttatcatg ggatccaagg

361 gaatacggta tgatcaattc gacgatcatt ccattcagac atctatggat tcccgacaca

421 tacctttata acagcgtgaa gatgagtcgt gatgagactg aacggtatat gaacatccag

481 gttgaatcgt tacattggaa aggagaaaac ggttcacaaa tgtcatttct ctatccggcc

541 atatacacga tcacttgtcg actcaatatc agatttttcc cgtacgatcg tcagaattgt

601 acgttgacaa tatccagctg gacgaattca atgtcggcgt tggactatta tgcggatcct

661 gaagtaaatc tggcatcgtt tataccaaat gaagaatggg atgtgaaatc gttcaagatc

721 tttagacatg agtacaagta cgcttgctgt gcggaaccat gggccatttt acaagcctcg

781 ctggtgattc aacgaaagcc actctattac atagtcaatt tgataattcc aacatcaatc

841 atcactatag tctctatcac cggatttttc acaccggcat cgacagacga cgaccgtacc

901 gagaagatta atctcggtat tacgacgttg ctggcgatgt ccattcttat gcttatggtt

961 tccgatcaaa tgccaacaac cagtgaattc gtcccgctca tagcatggtt ctacctatcc

1021 atcataatta tcgtcagtat tggaacattc cttacgagcg tcgtgctctc tgtccaaagt

1081 cgaaggcaat acgggcgaaa tccaccgctt tatattcgtt attacttctt tgttgttatc

1141 ccatcattca tatacgtcag tgtacctcca gcgttggaga acttgtggtc cgaactcgac

1201 gatgacccat tgaatgcttg gcgacgacga cgacgatcac cttctcctgg tcttcataag

1261 actgaatctc tatacaaaga ccagacaccg atatctcctc ctccactgga gcggaattca

1321 acgctgaaga tccctcgaca aggtttggat cgattcagtt cgattcaaat gatcgatgtc

1381 tctgtgccac catcgccggc gatttcacga atctcaagaa ctccgtcgac ggcgcaatca

1441 caaaaactca cgttatggga gggtacgatg agtgcgttgg caggaaacaa cgtgcagtta

1501 cgtcgtacat cagctgcata ctccaaagag gtagatgcta tgcgaaggaa acgacaatgc

1561 tcattggaat gggagttcct tgccacagtc ctcgataggt tcctgctgtt gctgttcatc

1621 tcagctgtgg tgttgatcac tatgggtcta gtggtggttg gtaaaatggc tcagttcagc

1681 tacgatcatc cagacgaggc attcttctaa

//

LOCUS Hco-deg-3H;7 1710 bp mRNA linear INV 05-MAR-2009

DEFINITION [gene=Hco-deg-3H].

SOURCE Haemonchus contortus

ORGANISM Haemonchus contortus

Eukaryota; Metazoa; Nematoda; Chromadorea; Rhabditida; Strongylida;

Trichostrongyloidea; Haemonchidae; Haemonchinae; Haemonchus.

REFERENCE 1 (bases 1 to 1710)

AUTHORS Rufener,L., Maser,P., Roditi,I. and Kaminsky,R.

TITLE Haemonchus contortus Acetylcholine Receptors of the DEG-3 Subfamily

and their Role in Sensitivity to Monepantel

JOURNAL PLoS Pathogens, in press

REFERENCE 2 (bases 1 to 1710)

AUTHORS Rufener,L., Maser,P., Roditi,I. and Kaminsky,R.

TITLE Direct Submission

JOURNAL Submitted (05-MAR-2009) Institute of Cell Biology, University of

Bern, Baltzerstrasse 4, Bern, BE 3012, Switzerland

FEATURES Location/Qualifiers

source 1..1710

/organism="Haemonchus contortus"

/mol_type="mRNA"

/isolate="Hc-CRA AAD-mutant"

/clone="7"

/note="monepantel-insensitive isolate"

CDS 1..1710

/note="Hco-DEG-3H"

/codon_start=1

/product="H. contortus DEG-3 homologue"

/translation="MRLHETSTMIFAVLLVVFSGCRVSKADLVSSLLINDSAASHTRA

TIMEEVPLIRLTRDLLMKDRYDVRARPIHDHTKPLKVHISISLYQIIEVDEPAQNIKL

NVWMIQKWKDEYLSWDPREYGMINSTIIPFRHLWIPDTYLYNSVKMSRDETERYMNIQ

VESLHWKGENGSQMSFLYPAIYTITCRLNIRFFPYDRQNCTLTISSWTNSMSALDYYA

DPEVNLASFIPNEEWDVKSFKIFRHEYKYACCAEPWAILQASLVIQRKPLYYIVNLII

PTSIITIVSITGFFTPASTDDDRTEKINLGITTLLAMSILMLMVSDQMPTTSEFVPLI

AWFYLSIIIIVSIGTFLTSVVLSVQSRRQYGRNPPLYIRYYFFVVIPSFIYVSVPPAL

ENLWSELDDDPLNAWRRRRRSPSPGLHKTESLYKDQTPISPPPLERNSTLKIPRQGLD

RFSSIQMIDVSVPPSPAISRISRTPSTAQSQKLTLWEGTMSALAGNNVQLRRTSAAYS

KEVDAMRRKRQCSLEWEFLATVLDRFLLLLFISAVVLITMGLVVVGKMAQFSYDHPDE

AFF"

BASE COUNT 468 a 397 c 372 g 473 t

ORIGIN

1 atgcgactac atgaaacctc gacaatgata ttcgctgttc ttctggtggt tttctctggt

61 tgcagggtct ccaaagcaga ccttgttagt tcgttgctga ttaatgactc tgctgcaagt

121 catactcgag cgacaatcat ggaagaagtt cctctgatac ggcttactcg agaccttctc

181 atgaaagata ggtatgatgt tcgtgctcga ccgattcacg accatacaaa gccactgaaa

241 gtccacatca gcatttcgct ttaccaaatt atcgaagtgg atgaaccggc ccagaatatc

301 aagttgaacg tgtggatgat tcaaaaatgg aaagatgagt atttatcatg ggatccaagg

361 gaatacggta tgatcaattc gacgatcatt ccattcagac atctatggat tcccgacaca

421 tacctttata acagcgtgaa gatgagtcgt gatgagactg aacggtatat gaacatccag

481 gttgaatcgt tacattggaa aggagaaaac ggttcacaaa tgtcatttct ctatccggcc

541 atatacacga tcacttgtcg actcaatatc agatttttcc cgtacgatcg tcagaattgt

601 acgttgacaa tatccagctg gacgaattca atgtcggcgt tggactatta tgcggatcct

661 gaagtaaatc tggcatcgtt tataccaaat gaagaatggg atgtgaaatc gttcaagatc

721 tttagacatg agtacaagta cgcttgctgt gcggaaccat gggccatttt acaagcctcg

781 ctggtgattc aacgaaagcc actctattac atagtcaatt tgataattcc aacatcaatc

841 atcactatag tctctatcac cggatttttc acaccggcat cgacagacga cgaccgtacc

901 gagaagatta atctcggtat tacgacgttg ctggcgatgt ccattcttat gcttatggtt

961 tccgatcaaa tgccaacaac cagtgaattc gtcccgctca tagcatggtt ctacctatcc

1021 atcataatta tcgtcagtat tggaacattc cttacgagcg tcgtgctctc tgtccaaagt

1081 cgaaggcaat acgggcgaaa tccaccgctt tatattcgtt attacttctt tgttgttatc

1141 ccatcattca tatacgtcag tgtacctcca gcgttggaga acttgtggtc cgaactcgac

1201 gatgacccat tgaatgcttg gcgacgacga cgacgatcac cttctcctgg tcttcataag

1261 actgaatctc tatacaaaga ccagacaccg atatctcctc ctccactgga gcggaattca

1321 acgctgaaga tccctcgaca aggtttggat cgattcagtt cgattcaaat gatcgatgtc

1381 tctgtgccac catcgccggc gatttcacga atctcaagaa ctccgtcgac ggcgcaatca

1441 caaaaactca cgttatggga gggtacgatg agtgcgttgg caggaaacaa cgtgcagtta

1501 cgtcgtacat cagctgcata ctccaaagag gtagatgcta tgcgaaggaa acgacaatgc

1561 tcattggaat gggagttcct tgccacagtc ctcgataggt tcctgctgtt gctgttcatc

1621 tcagctgtgg tgttgatcac tatgggtcta gtggtggttg gtaaaatggc tcagttcagc

1681 tacgatcatc cagacgaggc attcttctaa

//

LOCUS Hco-deg-3H;8 1710 bp mRNA linear INV 05-MAR-2009

DEFINITION [gene=Hco-deg-3H].

SOURCE Haemonchus contortus

ORGANISM Haemonchus contortus

Eukaryota; Metazoa; Nematoda; Chromadorea; Rhabditida; Strongylida;

Trichostrongyloidea; Haemonchidae; Haemonchinae; Haemonchus.

REFERENCE 1 (bases 1 to 1710)

AUTHORS Rufener,L., Maser,P., Roditi,I. and Kaminsky,R.

TITLE Haemonchus contortus Acetylcholine Receptors of the DEG-3 Subfamily

and their Role in Sensitivity to Monepantel

JOURNAL PLoS Pathogens, in press

REFERENCE 2 (bases 1 to 1710)

AUTHORS Rufener,L., Maser,P., Roditi,I. and Kaminsky,R.

TITLE Direct Submission

JOURNAL Submitted (05-MAR-2009) Institute of Cell Biology, University of

Bern, Baltzerstrasse 4, Bern, BE 3012, Switzerland

FEATURES Location/Qualifiers

source 1..1710

/organism="Haemonchus contortus"

/mol_type="mRNA"

/isolate="Hc-Howick"

/clone="8"

/note="monepantel-sensitive isolate"

CDS 1..1710

/note="Hco-DEG-3H"

/codon_start=1

/product="H. contortus DEG-3 homologue"

/translation="MRLHETSTMIFAVLLVVFSGFGVSKADLVSSLLINDSAASHTRA

TIMEEVPLIRLTRDLLMKDRYDVRVRPIHDHTKPLKVHISISLYQIIEVDEPAQNIKL

NVWMIQKWKDEYLSWDPREYGMINSTIIPFRHLWIPDTYLYNSVKMSRDETERYMNIQ

VESLHWKGENGSQMSFLHPAIYTITCRLNIRFFPYDRQNCTLTISSWTNSMSALDYYA

DPEVNLASFIPNEEWDVKSFKIFRHEYKYACCAEPWAILQASLVIQRKPLYYIVNLII

PTSTITIVSITGFFTPASTDDDRTEKINLGITTLLAMSILMLMVSDQMPTTSEFVPLI

AWFYLSIIIIVSIGTFLTSVVLSVQSRRQYGRNPPLYIRYYFFVVIPSFIYVSVPPAL

ENLWSELDDDPLNAWRRRRRSSSPGLHKTESLYKDQTPISPLPLERNSTLKIPRQGLD

RFSSIQMIDVSVPPSPAISRISRTPSTAQSQKLTLWEGTMSALAGNNVQLRHTSAAYS

KEVDAMRRKRQCSLEWEFLATVLDRFLLLLFISAVVLITMGLVVVGKMAQFSYDHPDE

AFF"

BASE COUNT 477 a 396 c 369 g 468 t

ORIGIN

1 atgcgactac atgaaacctc gacaatgata ttcgctgttc ttctggtcgt tttctctgga

61 ttcggggtct ccaaagcaga tcttgtcagt tcgttgctga ttaatgactc tgctgcaagt

121 catactcgag cgacaatcat ggaagaagtc cctctgatac ggcttactcg ggaccttctc

181 atgaaagata ggtatgatgt tcgtgttcga ccgattcacg accacacaaa gccgctgaaa

241 gtccacatca gcatttcgct ttaccaaatt atcgaagtgg atgaaccggc ccagaatatc

301 aagctgaacg tgtggatgat acaaaaatgg aaagatgagt atttatcatg ggatccaagg

361 gaatacggta tgatcaattc gacgatcatt ccattcagac atctatggat tcctgacaca

421 tacctttata acagtgtgaa gatgagtcgt gatgaaaccg aacggtatat gaacatccag

481 gtcgaatcgt tacattggaa aggagaaaac ggctcacaaa tgtcatttct ccatccagct

541 atatatacga tcacttgtcg gctcaatatc agatttttcc cgtacgatcg tcaaaattgc

601 acgttgacaa tatccagttg gacaaattcg atgtcggcgt tggactatta tgcggatcct

661 gaagtaaatc tagcatcatt cataccgaat gaagagtggg atgtgaaatc gttcaagatc

721 ttccgacatg agtacaagta cgcttgctgt gcggaaccat gggccatttt acaagcatca

781 ctggtgattc aacgaaagcc gctctactat atagtcaatt tgataattcc aacatcgacc

841 atcacgatcg tctccatcac cggattcttt acaccagcat cgacggacga cgaccgtacc

901 gagaagatta atctgggtat tacgacgctg ctggcgatgt ccattcttat gcttatggtt

961 tccgatcaaa tgccgacaac cagtgaattc gtcccattga tagcatggtt ctacctatcc

1021 atcataatta tcgtcagtat tggaacattc ctcaccagcg tcgtgctgtc tgttcaaagt

1081 cgaaggcaat acgggcgaaa tccaccacta tatattcgat attatttctt tgttgttatt

1141 ccatcgttca tatacgtcag tgtaccacca gcattggaga acttgtggtc cgaactcgac

1201 gatgatccat tgaatgcttg gcgacgacga cggcgatcat cttctcctgg tcttcataag

1261 actgaatctc tatacaaaga ccagacaccg atatctcctc ttccactaga gcggaattca

1321 acgctaaaga tccctcgaca aggtttggat cgattcagtt cgattcaaat gatcgatgtc

1381 tctgtgccac catcgccggc gatttcacga atctcaagaa ctccgtcgac ggcgcaatca

1441 caaaaactca cgttatggga gggtacgatg agtgcattag caggaaacaa cgtgcagtta

1501 cgtcatacat cagccgcata ctctaaagaa gtagatgcta tgcgaaggaa acggcaatgc

1561 tcattggagt gggaattcct tgccacagta ctcgataggt tcctgctgtt gctgttcatc

1621 tcagctgtgg tgttgattac gatgggtcta gttgtggttg gtaaaatggc tcaattcagc

1681 tacgatcatc cagacgaggc attcttctaa

//

LOCUS Hco-deg-3H;9 1710 bp mRNA linear INV 05-MAR-2009

DEFINITION [gene=Hco-deg-3H].

SOURCE Haemonchus contortus

ORGANISM Haemonchus contortus

Eukaryota; Metazoa; Nematoda; Chromadorea; Rhabditida; Strongylida;

Trichostrongyloidea; Haemonchidae; Haemonchinae; Haemonchus.

REFERENCE 1 (bases 1 to 1710)

AUTHORS Rufener,L., Maser,P., Roditi,I. and Kaminsky,R.

TITLE Haemonchus contortus Acetylcholine Receptors of the DEG-3 Subfamily

and their Role in Sensitivity to Monepantel

JOURNAL PLoS Pathogens, in press

REFERENCE 2 (bases 1 to 1710)

AUTHORS Rufener,L., Maser,P., Roditi,I. and Kaminsky,R.

TITLE Direct Submission

JOURNAL Submitted (05-MAR-2009) Institute of Cell Biology, University of

Bern, Baltzerstrasse 4, Bern, BE 3012, Switzerland

FEATURES Location/Qualifiers

source 1..1710

/organism="Haemonchus contortus"

/mol_type="mRNA"

/isolate="Hc-Howick"

/clone="9"

/note="monepantel-sensitive isolate"

CDS 1..1710

/note="Hco-DEG-3H"

/codon_start=1

/product="H. contortus DEG-3 homologue"

/translation="MRLHETSTMIFAVLLVVFSGFGVSKADLVSSLLINDSAASHTRA

TIMEEVPLIRLTRDLLMKDRYDVRVRPIHDHTKPLKVHISISLYQIIEVDEPAQNIKL

NVWMIQKWKDEYLSWDPREYGMINSTIIPFRHLWIPDTYLYNSVKMSRDETERYMNIQ

VESLHWKGENGSQMSFLYPAIYTITCRLNIRFFPYDRQNCTLTISSWTNSMSALDYYA

DPEVNLASFIPNEEWDVKSFKIFRHEYKYACCAEPWAILQASLVIQRKPLYYIVNLII

PTSIITIVSITGFFTPASTDDDRTEKINLGITTLLAMSILMLMVSDQMPTTSEFVPLI

AWFYLSIIIIVSIGTFLTSVVLSVQSRRQYGRNPPLYIRYYFFVVIPSFIYVSVPPAL

ENLWSELDDDPLNAWRRRRRSSSPGLHKTESLYKDQTPISPLPLERNSTLKIPRQGLD

RFSSIQMIDVSVPPSPAISRISRTPSTAQSQKLTLWEGTMSALAGNNVQLRRTSAAYS

KEVDAMRRKRQCSLEWEFLATVLDRFLLLLFISAVVLITMGLVVVGKMAQFSYDHPDE

AFF"

BASE COUNT 477 a 394 c 369 g 470 t

ORIGIN

1 atgcgactac atgaaacctc gacaatgata ttcgctgttc ttctggtcgt tttctctgga

61 ttcggggtct ccaaagcaga tcttgtcagt tcgttgctga ttaatgactc tgctgcaagt

121 catactcgag cgacaatcat ggaagaagtc cctctgatac ggcttactcg ggaccttctc

181 atgaaagata ggtatgatgt tcgtgttcga ccgattcacg accacacaaa gccgctgaaa

241 gtccacatca gcatttcgct ttaccaaatt atcgaagtgg atgaaccggc ccagaatatc

301 aagctgaacg tgtggatgat acaaaaatgg aaagatgagt atttatcatg ggatccaagg

361 gaatacggta tgatcaattc gacgatcatt ccattcagac atctatggat tcctgacaca

421 tacctttata acagtgtgaa gatgagtcgt gatgaaaccg aacggtatat gaacatccag

481 gtcgaatcgt tacattggaa aggagaaaac ggctcacaaa tgtcatttct ctatccagct

541 atatatacga tcacttgtcg gctcaatatc agatttttcc cgtacgatcg tcaaaattgc

601 acgttgacaa tatccagttg gacaaattcg atgtcggcgt tggactatta tgcggatcct

661 gaagtaaatc tagcatcatt cataccgaat gaagagtggg atgtgaaatc gttcaagatc

721 ttccgacatg agtacaagta cgcttgctgt gcggaaccat gggccatttt acaagcatca

781 ctggtgattc aacgaaagcc gctctactat atagtcaatt tgataattcc aacatcgatc

841 atcacgatcg tctccatcac cggattcttt acaccagcat cgacagacga cgaccgtacc

901 gagaagatta atctgggtat tacgacgctg ctggcgatgt ccattcttat gcttatggtt

961 tccgatcaaa tgccgacaac cagtgaattc gtcccattga tagcatggtt ctacctatcc

1021 atcataatta tcgtcagtat tggaacattc ctcaccagcg tcgtgctgtc tgttcaaagt

1081 cgaaggcaat acgggcgaaa tccaccacta tatattcgat attatttctt tgttgttatt

1141 ccatcgttca tatacgtcag tgtaccacca gcattggaga acttgtggtc cgaactcgac

1201 gatgatccat tgaatgcttg gcgacgacga cggcgatcat cttctcctgg tcttcataag

1261 actgaatctc tatacaaaga ccagacaccg atatctcctc ttccactaga gcggaattca

1321 acgctaaaga tccctcgaca aggtttggat cgattcagtt cgattcaaat gatcgatgtc

1381 tctgtgccac catcgccggc gatttcacga atctcaagaa ctccgtcgac ggcgcaatca

1441 caaaaactca cgttatggga gggtacgatg agtgcattag caggaaacaa cgtgcagtta

1501 cgtcgtacat cagccgcata ctctaaagaa gtagatgcta tgcgaaggaa acggcaatgc

1561 tcattggagt gggaattcct tgccacagta ctcgataggt tcctgctgtt gctgttcatc

1621 tcagctgtgg tgttgattac gatgggtcta gttgtggttg gtaaaatggc tcaattcagc

1681 tacgatcatc cagacgaggc attcttctaa

//

LOCUS Hco-deg-3H;10 1710 bp mRNA linear INV 05-MAR-2009

DEFINITION [gene=Hco-deg-3H].

SOURCE Haemonchus contortus

ORGANISM Haemonchus contortus

Eukaryota; Metazoa; Nematoda; Chromadorea; Rhabditida; Strongylida;

Trichostrongyloidea; Haemonchidae; Haemonchinae; Haemonchus.

REFERENCE 1 (bases 1 to 1710)

AUTHORS Rufener,L., Maser,P., Roditi,I. and Kaminsky,R.

TITLE Haemonchus contortus Acetylcholine Receptors of the DEG-3 Subfamily

and their Role in Sensitivity to Monepantel

JOURNAL PLoS Pathogens, in press

REFERENCE 2 (bases 1 to 1710)

AUTHORS Rufener,L., Maser,P., Roditi,I. and Kaminsky,R.

TITLE Direct Submission

JOURNAL Submitted (05-MAR-2009) Institute of Cell Biology, University of

Bern, Baltzerstrasse 4, Bern, BE 3012, Switzerland

FEATURES Location/Qualifiers

source 1..1710

/organism="Haemonchus contortus"

/mol_type="mRNA"

/isolate="Hc-Howick AAD-mutant"

/clone="10"

/note="monepantel-insensitive isolate"

CDS 1..1710

/note="Hco-DEG-3H"

/codon_start=1

/product="H. contortus DEG-3 homologue"

/translation="MRLHETSTMIFAVLLVVFFGCTGSKADLVSSLLINDSAASHTRA

TIMEEVPLIRLTRDLLMKDRYDVRVRPIHDHTKPLKVHISISLYQIIEVDEPAQNIKL

NVWMIQKWKDEYLSWDPREYGMINSTIIPFRHLWIPDTYLYNSVKMSRDETERYMNIQ

VESLHWKGENGSQMSFLYPAIYTITCRLNIRFFPYDRQNCTLTISSWTNSMSALDYYA

DPEVNLASFIPNEEWDVKSFKIFRHEYKYACCAEPWAILQASLVIQRKPLYYIVNLII

PTSIITIVSITGFFTPASTDDDRTEKINLGITTLLAMSILMLMVSDQMPTTSEFVPLI

AWFNLSIIIIVSIGTFLTSVVLSVQSRRQYGRNPPLYIRYYFFVVIPSFIYVSVPPAL

ENLWSELDDDPLNAWRRRRRSSSPGLRKTESICKEQTPISPPPLERNSTLRIPRQGLD

RFSSIQMIDVSVPPSPAISRISRTPSTAQSQKLTLWEGTMSALAGNNVQLRRTSAAYS

KEVDAMRRKRQCSLEWEFLATVLDRFLLLLFISAVVLITMGLVVVGKMAQFSYDHPDE

AFF"

BASE COUNT 475 a 405 c 372 g 458 t

ORIGIN

1 atgcgactac atgaaacctc gacaatgata ttcgctgttc ttctggtggt tttctttgga

61 tgcacgggct ccaaagcaga tctcgtcagt tcgctgctga ttaatgactc tgctgcaagt

121 catactcgag cgacaatcat ggaagaagtt cctctgatac ggcttactcg ggaccttctc

181 atgaaagata ggtatgatgt tcgtgttcga ccgattcacg accacacaaa gccactgaaa

241 gtccacatca gcatttcgct ttaccaaatt atcgaagtgg atgaaccggc ccagaatatc

301 aagttgaacg tgtggatgat acaaaaatgg aaagatgagt atttatcatg ggatccaagg

361 gaatacggta tgatcaattc gacgatcatt ccattcagac atctatggat acccgacaca

421 tacctttata acagtgtgaa gatgagtcgt gatgagaccg aacggtatat gaacatccag

481 gtcgaatcgt tacattggaa aggagaaaac ggttcacaaa tgtcatttct ctatccagct

541 atatacacaa tcacttgtcg actaaacatc agattcttcc cgtacgatcg tcagaattgt

601 acgttgacaa tatccagttg gacgaattca atgtcggcat tggactatta tgcggatccc

661 gaagtaaatc tggcatcgtt cataccgaac gaagaatggg atgtgaaatc gttcaagatc

721 ttccgacatg agtacaaata cgcttgctgt gcagaaccat gggccatttt acaagcatca

781 ttggtgattc aacgaaagcc gctctattac atagttaatc tgataattcc aacgtcgatc

841 atcactatcg tctccattac cggattcttc acaccggctt cgacagacga tgatcgtacc

901 gaaaagatta atctgggtat tacgacgctg ctggccatgt ccattcttat gctcatggtt

961 tccgatcaaa tgccgacaac cagtgaattc gttccgctca tagcatggtt caacctgtct

1021 atcataatta tcgtcagtat tggaacattc cttaccagcg tcgtgctctc tgttcaaagt

1081 cgaaggcaat acgggcgaaa tccaccactt tatatccgat actatttctt tgttgtcatt

1141 ccatcgttca tatacgtcag tgtaccacct gcgttggaga acttgtggtc cgaacttgat

1201 gatgatccac tgaatgcatg gcgacgacga cggcgatcat cttctcctgg tcttcgaaaa

1261 actgaatcta tatgcaaaga gcagacaccg atatctcctc ctccattgga gcggaattca

1321 acgctacgga tccctcgaca agggctggat cgattcagct caattcaaat gatcgacgtc

1381 tccgtgccgc catcgccggc gatatcacga atctcacgaa ccccgtcaac ggcacaatca

1441 caaaagctca cattatggga gggtacgatg agtgcgttag caggaaacaa cgtgcaatta

1501 cgtcgtacat cagccgcata ctctaaagaa gtagatgcta tgcgaaggaa acggcagtgc

1561 tcattggaat gggagttcct tgccacagtc cttgacaggt ttctgctgct gctgttcatc

1621 tcagctgtgg tgttgatcac tatgggtcta gtggtggttg gtaaaatggc tcagttcagc

1681 tacgaccatc cagacgaggc attcttctaa

//

LOCUS Hco-deg-3H;11 1710 bp mRNA linear INV 05-MAR-2009

DEFINITION [gene=Hco-deg-3H].

SOURCE Haemonchus contortus

ORGANISM Haemonchus contortus

Eukaryota; Metazoa; Nematoda; Chromadorea; Rhabditida; Strongylida;

Trichostrongyloidea; Haemonchidae; Haemonchinae; Haemonchus.

REFERENCE 1 (bases 1 to 1710)

AUTHORS Rufener,L., Maser,P., Roditi,I. and Kaminsky,R.

TITLE Haemonchus contortus Acetylcholine Receptors of the DEG-3 Subfamily

and their Role in Sensitivity to Monepantel

JOURNAL PLoS Pathogens, in press

REFERENCE 2 (bases 1 to 1710)

AUTHORS Rufener,L., Maser,P., Roditi,I. and Kaminsky,R.

TITLE Direct Submission

JOURNAL Submitted (05-MAR-2009) Institute of Cell Biology, University of

Bern, Baltzerstrasse 4, Bern, BE 3012, Switzerland

FEATURES Location/Qualifiers

source 1..1710

/organism="Haemonchus contortus"

/mol_type="mRNA"

/isolate="Hc-Howick AAD-mutant"

/clone="11"

/note="monepantel-insensitive isolate"

CDS 1..1710

/note="Hco-DEG-3H"

/codon_start=1

/product="H. contortus DEG-3 homologue"

/translation="MRLHETSTMIFAVLLVVFSGSRVSKADLVSSLLINDSAASHTRA

TIMEEVPLIRLTRDLLMKDRYDVRVRPIHDHTKPLKVHISISLYQIIEVDEPAQNIKL

NVWMIQKWKDEYLSWDPREYGMINSTIIPFRHLWIPDTYLYNSVKMSRDETERYMNIQ

VESLHWKGENGSQMSFLYPAIYTITCRLNIRFFPYDRQNCTLTISSWTNSMSALDYYA

DPEVNLASFIPNEEWDVKSFKIFRHEYKYACCAEPWAILQASLVIQRKPLYYIVNLII

PTSIITIVSITGFFTPASTDDDRTEKINLGITTLLAMSILMLMVSDQMPTTSEFVPLI

AWFYLSIIIIVSIGTFLTSVVLSVQSRRQYGRNPPLYIRYYFFVVIPSFIYVSVPPAL

ENLWSELDDDPLNAWRRRRRSSSPGLHKTESLYKDQTPISPPPLERNSTLKIPRQGLD

RFSSIQMIDVSVPPSPAISRISRTPSTAQSQKLTLWEGTMSALAGNNVQLRRTSAAYS

KEVDAMRRKRQCSLEWEFLATVLDRFLLLLFISAVVLITMGLVVVGKMAQFSYDHPDE

AFF"

BASE COUNT 472 a 404 c 372 g 462 t

ORIGIN

1 atgcgactac atgaaacctc gacaatgata ttcgctgttc ttctggtggt tttctctgga

61 tccagggtct ccaaagcaga ccttgtcagt tcgctgctga ttaatgactc tgctgcaagt

121 catactcgag cgacaatcat ggaagaagtt cctctgatac ggcttactcg ggaccttctc

181 atgaaagata ggtatgacgt tcgtgttcgg ccgatccacg accacacgaa gccactgaaa

241 gtccacatca gtatttcgct ctatcaaatt atcgaagtgg atgaaccggc acagaatatc

301 aagttgaatg tgtggatgat acaaaaatgg aaagatgagt atttatcatg ggatccaagg

361 gaatacggta tgatcaattc gacgatcatt ccattcagac atctatggat tcctgacaca

421 tacctttata acagtgtgaa gatgagtcgt gatgagaccg aacggtatat gaacatccag

481 gtcgaatcgt tacattggaa aggagaaaac ggctcacaaa tgtcatttct ttatcccgct

541 atatatacga tcacttgtcg gctcaatatc agattcttcc cgtacgatcg tcagaattgc

601 acgttgacaa tatccagttg gacgaattcc atgtcggcgt tggattacta tgcggatcct

661 gaagtaaatc tagcatcatt cataccgaat gaagagtggg atgtaaaatc gttcaagatc

721 ttccgacatg agtacaagta cgcttgctgt gcggaaccat gggccatttt acaagcatca

781 ctggtgattc aacgaaagcc gctctactat atagtcaatt tgataattcc aacatcgatc

841 atcactatcg tctccatcac aggattcttc acacccgctt cgacagacga tgaccgtacc

901 gagaagatta atctgggtat tacgacgttg ctggcgatgt ccatcctaat gcttatggtt

961 tccgatcaaa tgccgacaac cagtgaattc gtcccattaa tagcatggtt ctacctatcc

1021 atcataataa tcgtcagtat tgggacattc cttaccagcg tcgtgctctc tgttcaaagt

1081 agaaggcaat acgggcgaaa tccaccactt tatattcgat attatttctt tgttgtcatt

1141 ccatcgttca tatacgtcag tgtaccacca gcgttggaga acttgtggtc cgaactcgac

1201 gatgacccat tgaatgcttg gcgacgacga cggcgatcat cttctcctgg tcttcataag

1261 actgaatctc tatacaaaga ccagacaccg atatctcctc ctccactaga gcggaattca

1321 acgctgaaga tccctcgaca aggattggat cgattcagtt cgattcaaat gatcgacgtc

1381 tccgtgccac catcgccggc gatatcacga atctcccgaa ctccgtcaac ggcacaatca

1441 caaaagctca cattatggga gggtacaatg agtgcattag caggaaacaa cgtgcagtta

1501 cgtcgtacat cagccgcata ctccaaagag gtagatgcta tgcgaaggaa gcggcaatgc

1561 tcattggaat gggagttcct cgccacagtc cttgataggt tcctgctgtt gctgttcatc

1621 tcagctgtgg tgctgattac gatgggtctt gtggtggttg gtaaaatggc tcagttcagc

1681 tacgatcatc cagacgaggc attcttctaa

//
